# Lifitegrast Degradation: Products and Pathways

**DOI:** 10.3390/pharmaceutics17101299

**Published:** 2025-10-04

**Authors:** Leo Štefan, Ivan Sušanj, Jadranka Buljević, Marin Roje, Mladenka Jurin, Anđela Buljan, Tamara Rinkovec, Robert Vianello, Marijana Pocrnić, Nives Galić, Ana Čikoš

**Affiliations:** 1 JGL d.d. Jadran Galenski Laboratorij, 51000 Rijeka, Croatia; 2 Laboratory for Chiral Technologies, Division of Organic Chemistry and Biochemistry, Ruđer Bošković Institute, 10000 Zagreb, Croatia; marin.roje@irb.hr (M.R.); mladenka.jurin@inantro.hr (M.J.); andjela.buljan@irb.hr (A.B.); 3 Laboratory for the Computational Design and Synthesis of Functional Materials, Division of Organic Chemistry and Biochemistry, Ruđer Bošković Institute, 10000 Zagreb, Croatia; tamara.rinkovec@irb.hr (T.R.); robert.vianello@irb.hr (R.V.); 4 Department of Chemistry, Faculty of Science, University of Zagreb, 10000 Zagreb, Croatia; mpocrnic@chem.pmf.hr (M.P.); ngalic@chem.pmf.hr (N.G.); 5 NMR Centre, Ruđer Bošković Institute, 10000 Zagreb, Croatia

**Keywords:** lifitegrast, degradation products, NMR, HRMS, DFT calculations

## Abstract

**Background/Objectives**: Lifitegrast is a recent therapeutic agent provoking scientific and regulatory interest due to its outstanding safety profile and high efficacy in the treatment of dry eye disease. **Methods**: Herein we employ NMR spectroscopy and mass spectrometry to investigate the weak spots of lifitegrast under standard to extreme stress conditions, resulting in the characterization of three known and nine new degradation products (of which DP7 presented the greatest structural challenge, but was eventually determined as C10 hydroxy derivative, warranting a revision of its previously suggested structure). **Results**: The first weak spot is identified as a N1–C40 amide bond, and its high susceptibility to hydrolysis is explained through computational DFT analysis. The second and third weak spots are elucidated through bond dissociation energy (BDE) calculations which highlighted the oxidative vulnerabilities of both the piperidine and benzofuran ring. **Conclusions**: Additionally, two degradation products, observed in initial, extended, and targeted oxidative forced degradation studies, were selected for in silico toxicity assessment and were predicted to have toxicity profiles comparable to or lower than lifitegrast.

## 1. Introduction

Lifitegrast (LIF) represents a significant advancement in the treatment of dry eye syndrome (keratoconjunctivitis sicca) through a unique mechanism of action that targets the specific immunological pathways involved in ocular surface inflammation.

According to the Report of the Definition and Classification Subcommittee of the International Dry Eye Workshop [1], dry eye syndrome is “a multifactorial disease of the tears and ocular surface that results in symptoms of discomfort, visual disturbance, and tear film instability with potential damage to the ocular surface.” Unlike conventional therapies that primarily manage the symptoms, LIF is designed to inhibit interaction between lymphocyte function-associated antigen 1 (LFA-1) and intercellular adhesion molecule 1 (ICAM-1). LFA-1 is a heterodimeric integrin expressed on the surface of T and B lymphocytes. ICAM-1 mediates processes such as T-cell adhesion to endothelial and epithelial cells, cellular proliferation, and the release of inflammatory cytokines through its interaction with LFA-1. By inhibiting that interaction, LIF reduces inflammation and immune activation associated with dry eye syndrome [2].

LIF has been available on the market less than a decade, following the U.S. Food and Drug Administration (FDA) approval in 2016. Due to its high aqueous solubility, it is formulated as an ophthalmic solution, enabling direct delivery to the ocular surface. This formulation maximizes local therapeutic efficacy while minimizing systemic exposure and, consequently, reduces the likelihood of systemic adverse effects [3]. Its excellent safety profile [4] has been demonstrated in multiple clinical studies [5,6,7,8,9,10,11,12].

**Figure 1:**
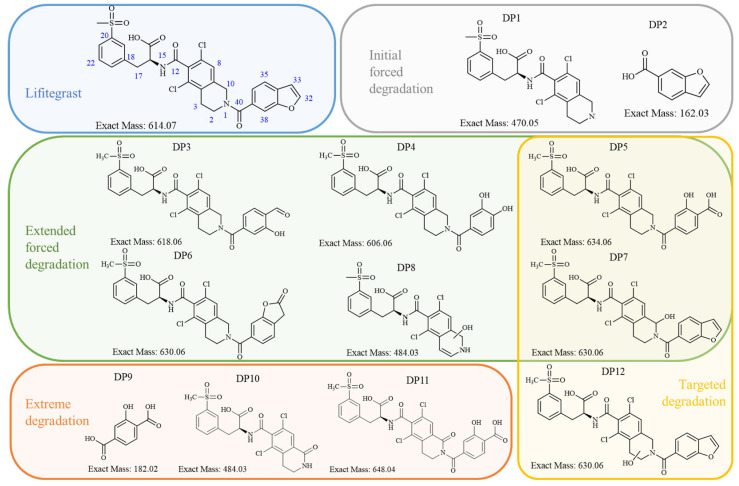
Lifitegrast, its two most common degradation products (DP1 and DP2), and overview of all degradation products discovered within this study (DP3 to DP12), colour coded according to degradation conditions of their discovery.

Structurally, LIF (Figure 1) is an aromatic heteropolycyclic compound classified as a phenylalanine derivative with one chiral centre. The *S* isomer is the therapeutically active, while the *R* isomer is considered an impurity and therefore requires specific chiral analytical methods for purity assessment during production [13].

At the time this study started, eight LIF-related products were commercially available as pharmaceutical-grade analytical standards, which included degradation products illustrated in Figure 1. At the time of writing, the number of LIF-related products has increased to 36 [14], reflecting the growing scientific and regulatory interest that this drug has inspired.

A literature search (SciFinder, 5 August 2025) identified 287 references related to LIF, of which 2 describe the development of analytical methods for quality control [13,15]. However, there is a notable lack of publicly available data concerning its degradation. Only one LIF degradation study has been reported [16] and it involved analysis using chromatography and MS in accordance with ICH Q1A(R2) guidelines. The results of that study (by Kumar et al.) indicate that LIF remains stable in aqueous solution and in solid form under photolytic and thermal stress conditions. In contrast, exposure to alkaline and acidic hydrolysis leads to decomposition into the known products DP1 and DP2 (Figure 1). They also report that oxidative stress results in the formation of another degradation product which they named “DP3”, characterized by a double bond at position C2=C3 in the dichlorotetrahydroisoquinoline (for LIF numbering, see Figure 1). The structure proposed as “DP3” was supported by the observed [M+H]^+^ 613.0582 and a key fragment ion at 423.0322, corresponding to the loss of the benzofuran carbonyl moiety. In our study, their “DP3” corresponds to the degradation product we identified as DP7, and we suggest that the structure proposed by Kumar et al. should be revised.

## 2. Materials and Methods

### 2.1. General

All solvents were of *puriss p.a.* quality or were distilled over appropriate drying reagents. All chemical reagents were obtained from commercial suppliers and used without further purification.

LIF (Sample ID: 74762, Material ID: 110000307, Batch No.: 82190138) was provided by Glenmark Pharmaceuticals (Maharashtra, India), and was characterized prior to the study using infrared (IR) spectrometry (Perkin Elmer, Shelton, CT, USA). Quality control tests included water content (Karl Fischer titration), sulphate content (gravimetric analysis), lithium content (atomic absorption spectrometry, AAS), and determination of benzylamine content, total impurities, and HPLC enantiomeric purity to ensure compliance with required specifications.

DP1 was obtained from TLC Pharmaceutical Standards, Newmarket, ON, Canada (4410-072A 12), DP2 from TLC Pharmaceutical Standards, Newmarket, ON, Canada (4487-011A1), and DP7 from Ultron Research, Hong Kong, China (ULAC2416).

### 2.2. Degradations

In total, four rounds of forced degradations were performed, starting with a broad set of degradation conditions (initial and extended degradation) and converging into oxidative degradation conditions (extreme and targeted degradation). Detailed experimental conditions can be found in corresponding sections of the Supplementary Materials.

### 2.3. Chromatography and Mass Spectrometry

Chromatography and MS analyses were performed on a Waters UPLC-PDA (Waters Corporation, Milford, CT, USA), Agilent 1260 Infinity II SFC/UHPLC Hybrid System, preparative Agilent 1260 Infinity II with UV detector, and Agilent 1290 Infinity II UHPLC coupled with Agilent 6550 iFunnel Q-TOF (Agilent Technologies, Santa Clara, CA, USA). Chromatographic separation for samples after the initial degradation study and extended oxidative degradation, as well as for the degradation monitoring and structure elucidation of degradants after targeted oxidative degradation, was achieved on an Acquity UPLC HSS T3 (2.1 × 100 mm, 1.8 μm) column, with mobile phases consisting of 20 mM ammonium formate and acetonitrile in the ratios of 99:1 (A) and 20:80 (B). The gradient elution programme was set as follows: initial conditions 100 % A; 0–3 min 100% A; 3–15 min 100–50% A; 16 min 50% A; 16–22 min 50–0% A; 22–27 min 0% A; 27–28 min 0–100% A; and 35 min 100% A. Flow rate was set at 0.4 mL/min, and column temperature was set at 40 °C. For LC-UV methods detection wavelength was set at 210 nm, and MS detection was achieved with electrospray ionization in positive and negative mode. For extreme degradation study, Zorbax XDB C18 (4.6 × 150 mm, 3.5 μm) column was used with mobile phases consisting of aqueous mobile phase with 0.1% formic acid (A) and methanol (B). Isolation of degradation products was achieved on an Extend-C18 column (5 μm, 21.2 × 150 mm) with previously described mobile phases consisting of 20 mM ammonium formate and acetonitrile. Detailed experimental conditions for each performed analysis can be found in corresponding sections of the Supplementary Materials.

All UHPLC-HRMS methods were intended for identification and structural characterization of degradation products, so they were not validated. The reproducibility of the method was ensured by analyzing the LIF standard solution during every run.

### 2.4. Nuclear Magnetic Resonance Spectroscopy

All NMR spectra were recorded on a Bruker Avance AV600 spectrometers equipped with a 5 mm broadband RT probe and a z-gradient accessory (Bruker, Billerica, MA, USA). Standard pulse sequences were used for data acquisition on samples dissolved in DMSO-d_6_ containing TMS as an internal standard. All spectra were acquired at 25 °C and processed using TopSpin 2.1 and 4.2 (Bruker, Billerica, MA, USA) and ACD/Labs Spectrus Processor^TM^ 2021.1.1 (ACD/Labs, Toronto, ON, Canada) software packages. Deuterated solvents for NMR spectroscopy were obtained from EurIsotop (Saint Aubin, France). Detailed experimental conditions for each performed NMR experiment can be found in the corresponding sections of the Supplementary Materials.

### 2.5. Molecular Modelling

To account for its conformational flexibility, the initial geometry of LIF was attained through Conformer-Rotamer Ensemble Sampling Tool (CREST) analysis [17]. Several of the most representative structures identified in this way were then reoptimized in Gaussian 16 (rev. C.01) software [18], employing the B3LYP/Def2TZVPP model supplemented with the implicit SMD solvation corresponding to the aqueous solution. The most stable structure was subsequently used in obtaining the thermodynamic and kinetic values discussed throughout the text. The DFT setup was selected following literature recommendations for the considered properties and system types [19], and was additionally evaluated through a series of single-point energy calculations employing different DFT functionals (Tables S12–S14). Each geometry optimization was followed by vibrational frequency analysis, offering thermal corrections so that all presented results correspond to Gibbs free energies at room temperature and normal pressure. All transition state structures were located through the scan procedure which employed both 1D and 2D scans, the latter specifically utilized to exclude the possibility for concerted mechanisms, and then fully optimized as saddle points on the potential energy surface. Apart from the visualization of the obtained negative frequencies, the validity of all transition states was confirmed through intrinsic reaction coordinate (IRC) calculations in both directions. For the bond dissociation energy calculations, all radicals were treated with the unrestricted UB3LYP formalism.

### 2.6. In Silico Safety Evaluation

The public domain search showed no toxicological data on DP5 and DP7, degradation products which were chosen due to their simultaneous structural diversity from and similarity to LIF. In such cases alternative approaches are applied, including the European Medicines Agency (EMA)-recommended [20] non-animal in silico method Quantitative Structure-Activity Relationships (QSAR) and read-across analysis using analogues, with a preference for data from the active pharmaceutical ingredient (API). Therefore, Derek Nexus v6.2.1 [21], Nexus 2.5.2. [22], Leadscope Genetox Statistical Suite v2022.0.0-31 [23], and Vega IRFMN v.1.0.2 [24] software packages were employed to evaluate the safety of DP5 and DP7 in accordance with the guidelines established by ICH M7 [25], Q3C [26], and Q3D [27].

## 3. Results and Discussion

Aiming to fully understand it, LIF degradation was explored through a series of experiments, with conditions ranging from those commonly employed—initial forced degradation that lasted 7 days and extended forced degradation in duration of 30 days—to extreme oxidative scenarios (30% hydrogen peroxide for 3 months) and targeted approaches (3% H_2_O_2_ for 7 days). These conditions were chosen to induce more extensive degradation, intended to increase the likelihood of producing, identifying, isolating, and characterizing unknown species. The discovered structures were then used to elucidate the degradation pathways and mechanisms, as well as to assess the in silico safety of the most structurally intriguing newly identified degradation products.

### 3.1. Initial and Extended Forced Degradation

The initial seven day forced degradation study (Supplementary Materials, Section S1) included the usual set of stress conditions to evaluate the effects of temperature, relative humidity, light irradiation, pH media, oxidative conditions (H_2_O_2_), and presence of radical initiator (AIBN) on LIF stability. Most of the results confirmed the literature data: LIF in its solid form remains stable, while in solution under acidic and basic conditions it degrades into DP1 (Supplementary Materials, Figures S8–S12 (NMR) and Figures S33–S36 (MS)) and DP2 (Figures S13–S17 (NMR)). However, the outcomes of H_2_O_2_ and AIBN experiments revealed multiple previously uncharacterized degradation products. Curious, we extended those experiments by incorporating elevated temperatures and prolonged exposure times (Supplementary Materials, Section S2). After 30 days at 50 °C/75% RH a comprehensive analysis of H_2_O_2_ oxidative mixture was performed using NMR spectroscopy and MS.

### 3.2. Analyzing Oxidative Forced Degradation Mixture

**Figure 2:**
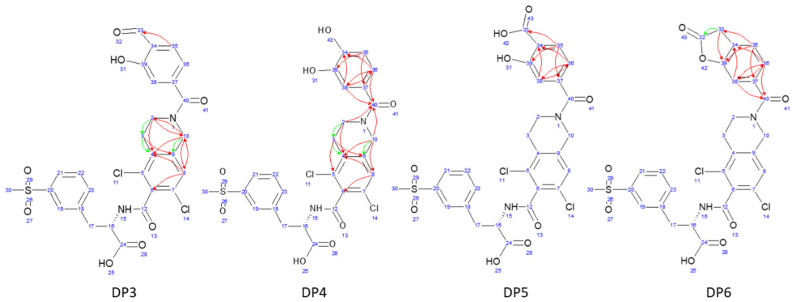
Key ^1^H-^13^C HMBC interactions (green arrows show 3-bond correlations, red arrows show 4-bond correlations) for degradation products discovered in mixture after extended oxidative degradation with 3% H_2_O_2_ after 30 days at 50 °C/75% RH.

The NMR analysis of the oxidative extended degradation mixture (Supplementary Materials, Section S3.1) revealed at least five compounds structurally similar to LIF (Figures S3–S7). Among these, the structures of DP3, DP4, DP5, and DP6 were determined (Figure 2 and Figures S18–S21 (NMR)), while the remaining component(s) could not be identified due to their low molar content and significant spectral overlap.

All elucidated structures share the extensive degradation of the benzofuran ring. DP4 exhibited the characteristic chemical shifts at C34 (147.3 ppm) and C39 (145.1 ppm), consistent with di-hydroxyl substitution. DP5 was characterized by a carboxyl group at 171.5 ppm, correlating with the aromatic doublet CH35 (proton at 7.84 ppm). From this doublet, key ^1^H-^13^C HMBC interactions with C37 (142.1 ppm) and C39 (161.0 ppm) allowed for the identification of the remaining benzene substituents and their positions. During the course of our study, a patent [28] was published describing the preparation of degradation product DP5 from LIF via oxidative degradation, confirming our findings. DP6 showed a ketone group at 173.1 ppm in ^1^H-^13^C HMBC correlation to CH_2_-33 (proton at 3.52 ppm, carbon at 35.6 ppm). Although it was evident from the start that there was one more molecule in the mixture, the structure of DP3 was determined last. A distinctive carbon signal at 193.5 ppm accompanied by a corresponding proton signal at 10.17 ppm gave evidence that DP3 was an aldehyde. This structural assignment was subsequently confirmed through analyses of fragmentation patterns in MS/MS and HRMS.

**Figure 3:**
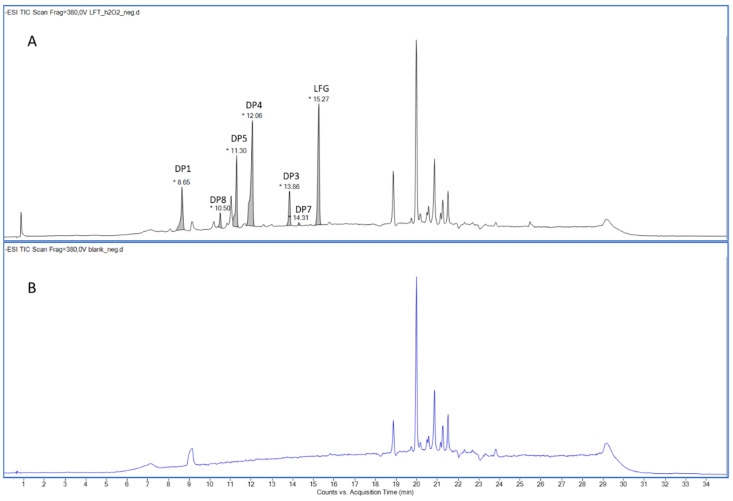
ESI− total ion chromatogram of the LIF (**A**) and blank (**B**) after extended oxidative degradation with 3% H_2_O_2_ after 30 days at 50 °C/75% RH.

Chromatographic analysis (Supplementary Materials, Section S3.2) of the degradation mixture revealed a total of seven peaks (Figure 3 and Figure S30, Table 1) suitable for structural elucidation by MS/MS and HRMS. Two of the detected chromatographic peaks were assigned to the parent compound LIF (Figures S31 and S32) and the known degradation product DP1 (Figures S33–S36) [23]. Peaks at RT 13.86 min, 12.06 min, and 11.30 min were associated with structures proposed during NMR analysis, namely DP3, DP4, and DP5, respectively. All three compounds lacked the characteristic ion at *m*/*z* 145 in their MS/MS fragmentation spectra, suggesting modifications within the benzofuran moiety. For DP3 (Figures S37–S40), fragment ions at *m*/*z* 149 and 376 supported the presence of an aldehyde group; for DP4 (Figures S41–S44), ions at *m*/*z* 137 and *m*/*z* 364 indicated a diol structure; and for DP5 (Figures S45–S48), ions at *m*/*z* 165 and *m*/*z* 392 were consistent with a carboxylic acid derivative.

The remaining chromatographic peaks at RT 10.51 min (DP8) and RT 14.31 min (DP7) corresponded to compounds not visible in the NMR spectra due to their low concentrations. The absence of the characteristic *m*/*z* 145 ion, or any of its analogues, indicates that the benzofuran moiety is absent in DP8 (Figures S53–S56). Its ESI+ *m*/*z* 485.0357 and ESI− *m*/*z* 483.0208 confirm this hypothesis, and suggest there might be a double bond and a hydroxy group attached to the core. Since the ion at *m*/*z* 485 undergoes a neutral loss of 243 Da, resulting in a fragment at *m*/*z* 241 (comparable fragmentation pattern was observed for LIF), it seems that the methylsulfonylbenzene moiety remains unmodified. Therefore, both the double bond and hydroxy group must be in the piperidine ring, although with this fragmentation pattern multiple positions are theoretically possible.

While at this time HRMS and MS/MS analysis provided strong indications of the structure for DP7 (Table 1, Figures S49–S52), two key observations necessitated further investigation. First, the extremely low concentration of this impurity complicated definitive analysis. Second, although the molecular ion [M–H]^−^ at *m*/*z* 629.0589 was detected in ESI− mode, the ESI+ spectrum showed only a prominent peak at *m*/*z* 613.0621, suspiciously similar to the result reported by Kumar et al. [16] for an oxidative degradation product featuring a double bond between C2 and C3 they named “DP3”. Although we found no evidence supporting the presence of that specific compound and believed our observed *m*/*z* 613.0621 in ESI+ to be an in-source fragment of DP7, we opted to investigate this further before drawing final conclusions. Therefore, the structure of DP7 is discussed in detail later in the text.

### 3.3. Extreme Oxidative Degradation

**Figure 4:**
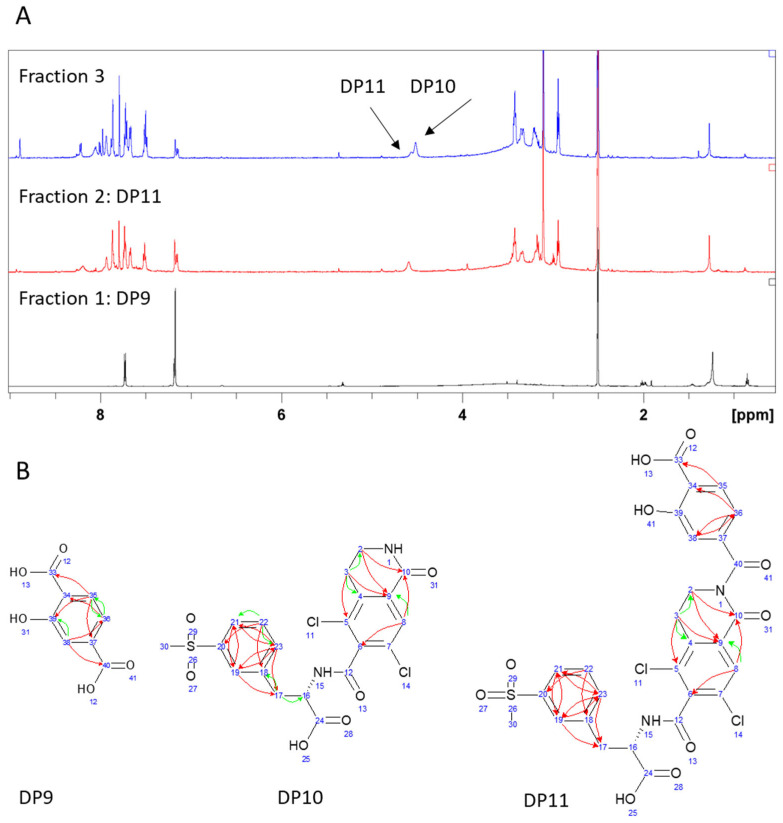
(**A**) ^1^H NMR spectra of three fractions resulting from extreme degradation (30% H_2_O_2_ for 90 days); and (**B**) key ^1^H-^13^C HMBC interactions (green arrows show 3-bond correlations, red arrows show 4-bond correlations) of identified degradation products.

The main idea behind extreme oxidative degradation (Supplementary Materials, Section S4) and using 30% H_2_O_2_ at 50 °C and 75% RH for a period of three months was to produce enough degradation material to enable isolation and individual characterization of DP7. From the resulting degradation mixture, three fractions (Figure 4A) were separated using TLC and column chromatography. LC-UV analysis showed that fraction 1 contained DP9 with ESI− *m*/*z* 180.95 at RT 6.7. The primary NMR evidence (Figures S57–S60) for its structure was provided by the presence of two carbonyl groups, assigned to C33 (170.8 ppm) and C40 (168.1 ppm), and their ^1^H-^13^C HMBC correlations with the benzene ring protons (Figure 4B).

Comparison of the DP11 (fraction 2) NMR spectra (Figure 4A and Figures S65–S68) to DP9 and DP5 immediately revealed strong similarities in chemical shifts and correlation patterns, indicating that the benzofuran ring underwent the same chemical transformation. Regarding the remainder of the molecule, the only notable difference in DP11 in comparison to LIF and DP5 was the presence of a ketone group with a chemical shift at 161.3 ppm, suggesting oxidation at position C10.

Fraction 3 (Figures S61–S64) contained two sets of resonance signals: one corresponding to the major component DP10 and the other to the previously identified DP11 (approximately 20%). A clear distinction between the two was observed in the integral values of the hydroxybenzoic proton signals, suggesting that DP10 lacked this functional group. Additional confirmation was provided by the presence of a ^1^H-^1^H COSY correlation between NH1 and H2 which was visible in DP10, but not in DP11. Finally, DP10 also exhibited a ketone signal at 161.6 ppm, indicating oxidation at position C10.

Although DP7 could not be obtained through such extreme degradation, it was interesting to explore the extent of oxidative transformations under such harsh conditions, especially in the view of understanding the degradation pathways.

### 3.4. Targeted Oxidative Degradation

**Figure 5:**
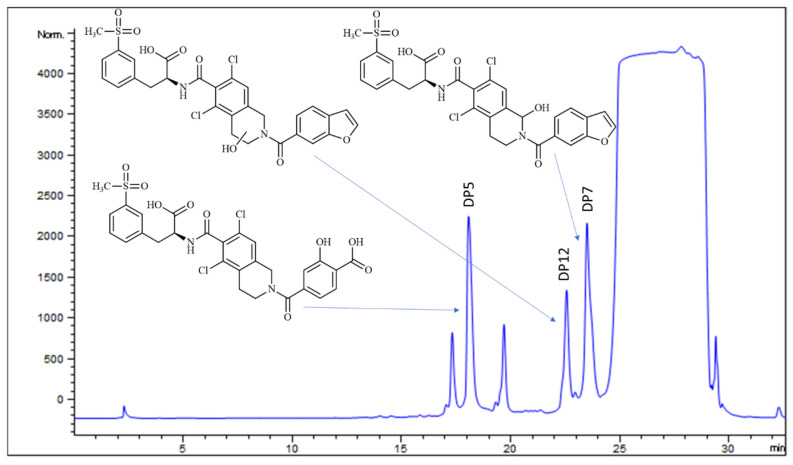
HPLC-UV chromatogram of LIF oxidative degradation mixture before separation with structures of all three degradation products.

Reluctant to abandon our efforts to fully characterize DP7, we experimented with various degradation conditions and durations, finally converging onto targeted efficiency rather than brute force: 50 mg/ml LIF in aqueous solution with 3% H_2_O_2_ for 7 days resulted in a mixture from which three degradation products were isolated by preparative HPLC (Figure 5). To facilitate the comparison with previous MS/MS and HRMS analyses, the three individual compounds were analyzed using exactly the same conditions (Supplementary Materials, Section S5). The isolated amounts were not sufficient for NMR analysis.

Isolated DP5 exhibited matching retention times with previous MS analyses, specifically ESI+ *m*/*z* 635.0691 and ESI− *m*/*z* 633.0525, confirming its structure (Figures S72–S75).

DP12 (Figures S76–S81) was not observed in any of the earlier degradation experiments, suggesting that it may form only under mild oxidative conditions or might be sensitive to further chemical transformations. Its concentration in the degradation mixture was notably low. With a [M+H]^+^ ion at *m*/*z* 631.0711 and [M-H]^−^ ion at *m*/*z* 629.0620, DP12 seems to contain one additional hydroxyl group compared to LIF. The position of this hydroxyl group was inferred from fragmentation *m*/*z* 613.0603 in ESI+, which indicates the loss of a water molecule and formation of a double bond and is feasible only if the hydroxyl group is located in the piperidine ring. Additional fragments at *m*/*z* 451, *m*/*z* 423, and *m*/*z* 370 support this hypothesis, as they correspond to LIF *m*/*z* 453, *m*/*z* 425, and *m*/*z* 372, each reduced by two units, which is also consistent with the formation of a double bond. This narrowed the options for substitution to either C2 or C3, although the exact position could not be determined.

**Figure 6:**
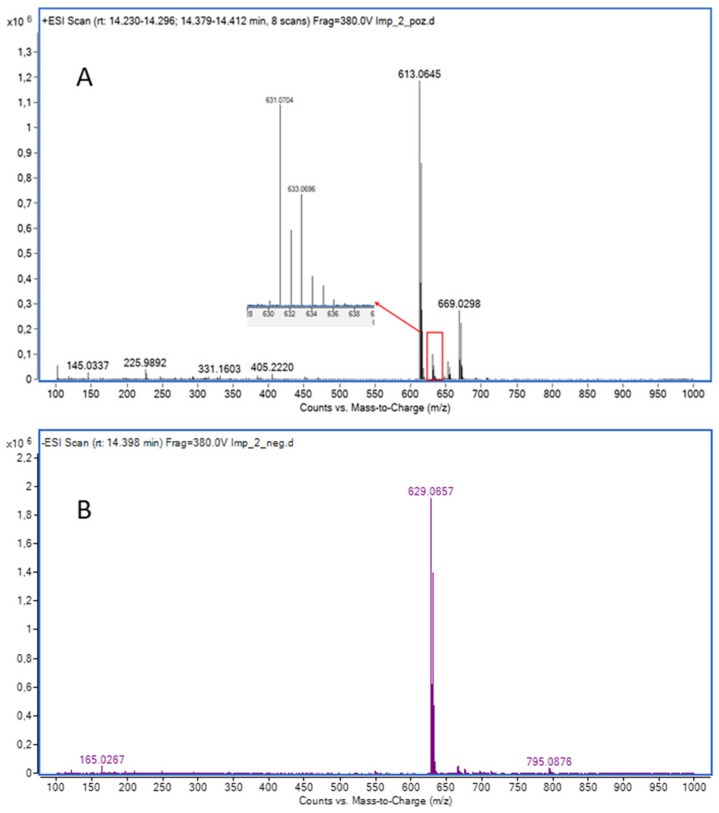
(**A**) ESI+ MS spectrum of isolated DP7; (**B**) ESI− MS spectrum of isolated DP7.

**Figure 7:**
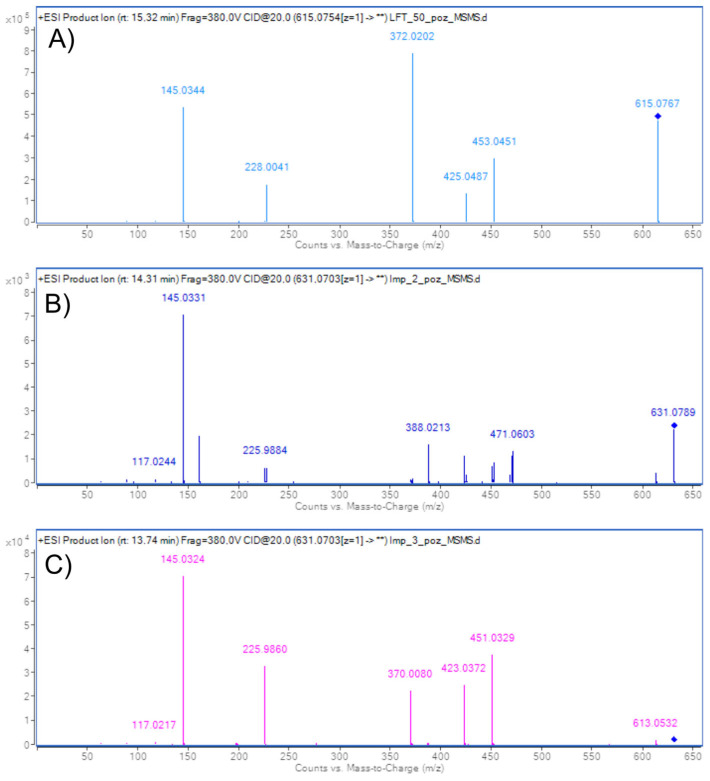
ESI+ MS/MS spectra of LIF (**A**), DP7 (**B**), and DP12 (**C**). Collision energy 20 V.

**Figure 8:**
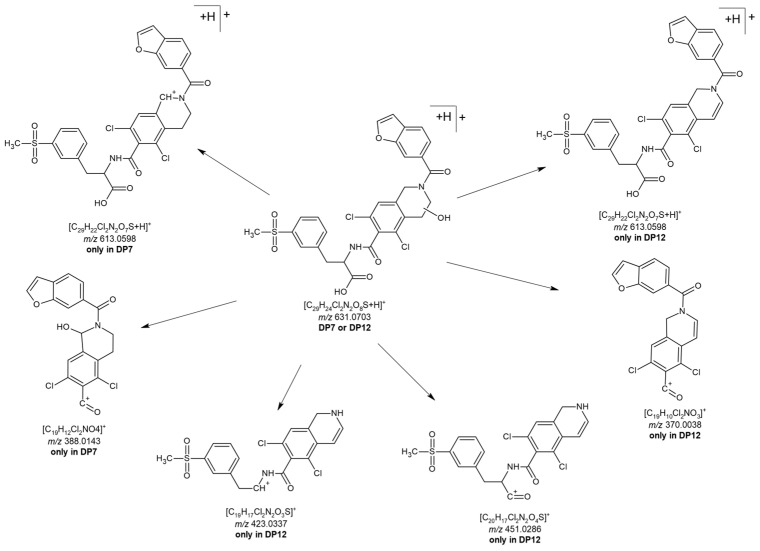
Characteristic fragments of DP7 and DP12 used in elucidation of the structures of degradation products.

Finally, the retention time of the third isolated compound corresponded to DP7, previously determined in the mixture. The molecular ion of isolated DP7, however, was observed in both ESI+ (*m*/*z* 631.0704) and ESI− (*m*/*z* 629.0657), although the signal intensity in ESI+ was very low (Figure 6 and Figures S82–S87). The fragmentation pattern in ESI+ mode for molecular ion *m*/*z* 631 closely resembles that of LIF, with the fragment at *m*/*z* 372 in LIF corresponding to *m*/*z* 388 in DP7, which is consistent with the addition of a hydroxyl group (+16 Da) to the piperidine ring. Based on this, it could be expected that the previously mentioned DP12 would also show the fragment at *m*/*z* 388. Instead, it seems that the loss of water and subsequent formation of a C2=C3 double bond is more favourable in DP12, as indicated by the presence of *m*/*z* 370 (−18 Da). In contrast, the fragmentation pattern of DP7 lacks all three characteristic ions associated with double bond formation, *m*/*z* 451, *m*/*z* 423, and *m*/*z* 370 (found in DP12), which leads to the conclusion that the hydroxyl group in DP7 is positioned where formation of the double bond is not favourable, i.e., at C10 of the piperidine ring (Figure 7 and Figure 8). Fragmentation in the ESI− MS spectrum (Figure 6B and Figures S51 and S52) was less informative, as it predominantly related to fragments of the methylsulfonyl moiety, which is structurally identical across LIF, DP7, and DP12.

Comparing our results to data reported by Kumar et al. [16], the similarities are obvious. They performed MS analysis only in ESI+ mode, yielding *m*/*z* 613.0582 which was attributed to a LIF-like molecule featuring a double bond between positions C2 and C3. Their MS/MS analysis of *m*/*z* 613.0582 revealed a fragmentation pattern consisting of ions at *m*/*z* 423.0322, *m*/*z* 225.9817, and *m*/*z* 145.1280, matching our pattern in ESI+ for *m*/*z* 613 at CE 20 V, which also included *m*/*z* 423.0308, *m*/*z* 225.9811, and *m*/*z* 145.0270. As in their work, when performing analysis in the mixture, we also did not detect the molecular ion in ESI+ mode. However, in both samples we did detect the DP7 molecular ion *m*/*z* 629 in ESI− mode; therefore, we are convinced that the observed *m*/*z* 613.0621 in ESI+ is an in-source fragment of DP7.

### 3.5. Comparison of Isolated DP7 to Purchased Analytical Standard of the Same Structure

During the course of this investigation, a compound with an identical structure to our proposed DP7 became commercially available. To eliminate any remaining uncertainty, we purchased the compound, confirmed its structure using NMR spectroscopy, and compared its MS data to isolated DP7 (Supplementary Materials, Section S6).

Key NMR evidence for the purchased DP7 structure was a C10 carbon chemical shift of 75.2 ppm (revealed by ^1^H-^13^C HMBC interaction of C10 with H8 singlet at 7.34 ppm), which is characteristic of carbons with a hydroxyl group attached to them. UPLC-HRMS analysis revealed a slight difference in retention times between the purchased DP7 and the previously analyzed isolated DP7. To resolve this discrepancy, LIF, the isolated DP7, and the purchased DP7 were re-analyzed at the same time and under identical chromatographic conditions (Figure S91). The peak corresponding to LIF was observed at 15.19 min, while the chromatographic peaks for both the isolated and purchased DP7 shared the same retention time of 14.23 min, both having RRT 0.94. The molecular ion for the purchased DP7 was detected in the ESI− MS spectrum at *m*/*z* 629.0564, and the ESI+ spectrum showed the characteristic *in-source* fragment at *m*/*z* 613, consistent with previous analyses (Figures S88–S90). In short, the purchased DP7 was conclusively confirmed to be identical to the isolated compound DP7.

### 3.6. Computational DFT Analysis of Degradation Mechanisms

Computational DFT analysis was employed to elucidate the mechanistic details of LIF degradation pathways and support the interpretation of NMR and MS data. Initially, we focused on the predominant degradation process under normal condition, which is hydrolytic cleavage of the N1–C40 amide bond, seeking to explain its significantly higher susceptibility to degradation compared to the analogous N15–C12. Additionally, after conducting extensive forced degradation studies via radical initiation of LIF under oxidative conditions, we evaluated the propensity of all relevant chemical bonds, particularly those at heteroatom–H sites, to undergo homolytic cleavage.

Amide bond hydrolysis is a fundamental chemical process with broad implications in organic chemistry, biochemistry, and industrial applications. Yet, this linkage is among the most stable functional groups in organic chemistry due to resonance interaction between involved carbonyl carbon and nitrogen in the C(=O)–NR group. This stability results in slow hydrolysis under neutral conditions, requiring catalysts, high temperatures, or enzymes to proceed at practical rates. Consequently, our analysis focused on acidic, basic, and neutral hydrolysis pathways, employing H_3_O^+^, OH^−^, and H_2_O as hydrolytic agents, and assessed their kinetic and thermodynamic properties.

Given its established importance, the stability of the amide bond has been the subject of extensive research. Since the seminal work by Schowen, Jayaraman, and Kershner [29], a number of studies have identified three possible mechanistic scenarios. The most common mechanism during hydrolysis is the stepwise mechanism [30], especially for the acid- and base-catalyzed processes, which involves forming a tetrahedral intermediate following nucleophilic attack on the amide carbonyl carbon. Additionally, there is evidence for concerted [31] and dissociative mechanisms [32], though these depend on specific substrate structures and reaction conditions.

**Figure 9:**
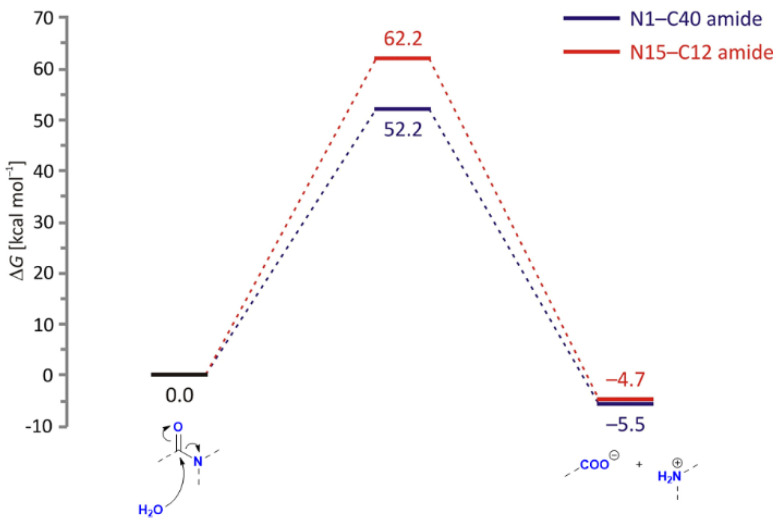
Free energy profiles for the neutral hydrolysis of N1–C40 (blue) and N15–C12 (red) amide bonds in LIF, obtained by the (SMD)/B3LYP/Def2TZVPP model in water.

Our results for the neutral hydrolysis under relevant physiological conditions confirm the high amide stability and align with the concerted mechanisms, where the nucleophilic attack by water and the cleavage of the C–N bond occur simultaneously, without forming a stable tetrahedral intermediate (Figure 9). The concerted nature of neutral hydrolysis ties with similar reports [33,34], while the reaction proceeds with different kinetic requirements among amides. Namely, the reaction is initiated by a water-to-carbonyl approach concerted with a water-to-nitrogen proton transfer. This implies that the transition state effectively features an N-protonated amide, with N1–H and N15–H distances of 1.04 and 1.05 Å, and a hydroxyl nucleophile with C40–O^−^ and C12–O^−^ distances of 2.22 and 2.25 Å, respectively. In line with these differences, the N1–C40 amide between the piperidine and benzofuran units appears much more prone to hydrolysis, with the activation free energy of Δ*G*^‡^ = 52.2 kcal/mol (235*i* cm^−1^) being 10.0 kcal/mol lower than that for the other N15–C12 amide (277*i* cm^−1^). Although both activation parameters indicate extremely slow processes, their trend confirms hydrolytic degradation products observed only for the N1–C40 amide. It turns out that the crucial structural difference contributing to different kinetic parameters is the presence of carboxylate in the vicinity of the more stable N15–C12 amide, which appears to hinder its hydrolysis. Specifically, through charge repulsions with the incoming nucleophile, formally hydroxide, and hydrogen bonding with the protonated amide, this group increases both of the mentioned C–O and N–H distances and renders N15–C12 hydrolysis more demanding. In other words, anionic C24-carboxylate prevents a more efficient N15–C12 hydrolysis, a trend persistent in all other analyzed processes (see later) and strongly in line with experimental observations. Interestingly, although the difference in N(amide)–H bonds of 0.01 Å is only marginal, it results in a significantly larger atomic charge on N15 (−0.63 |e|) compared to N1 (−0.44 |e|), thus also reflecting its lower tendency for the hydrolytic cleavage. The described concerted pathway avoids the high-energy intermediate, and the formed transition state structure immediately collapses into the final products: cleaved amide with carboxylate and protonated amine on either end. The overall thermodynamic profile also promotes the N1–C40 hydrolysis, as its reaction Gibbs free energy (Δ*G*_R_ = −5.5 kcal/mol) is −0.8 kcal/mol more exergonic.

In addition, we were able to locate a stepwise pathway for the N15–C12 hydrolysis, with the intermediate afforded by the C24 carboxylate striping the added N15 proton and featuring the formed C12–O(H) bond at 1.47 Å. However, the transition state for its degradation into final products is even higher at Δ*G*^‡^ = 63.9 kcal/mol (239*i* cm^−1^), and it was not considered further. Still, we mention that these results agree with the work of Boyd and co-workers [34], who also studied both possibilities and demonstrated the prevalence of the concerted over stepwise mechanism in various *N*-(*o*-carboxybenzoyl)-*L*-amino acids.

**Figure 10:**
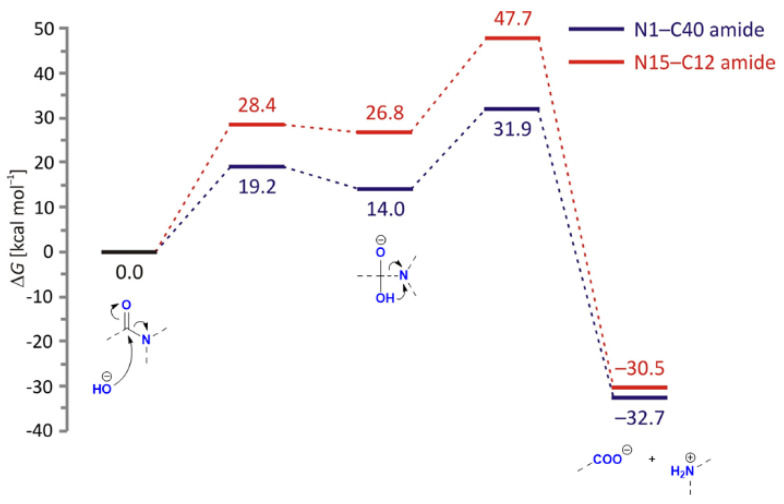
Free energy profiles for the base-catalyzed hydrolysis of N1–C40 (blue) and N15–C12 (red) amide bonds in LIF, obtained using the (SMD)/B3LYP/Def2TZVPP model in water.

Base-catalyzed hydrolysis features the approaching hydroxyl group, which will, expectedly, facilitate amide hydrolysis due to its higher nucleophilicity over neutral water (Figure 10). Unlike the previous case, here the reaction proceeds in a stepwise fashion, with the first transition state featuring a partly formed C(amide)–O(hydroxyl) bond (1.95 Å for N1–C40, and 1.84 Å for N15–C12). This process is again kinetically more favourable for N1–C40 amide, and in both cases offers the high-energy tetrahedral intermediate, allowing only a few kcals/mol stabilization relative to the preceding transition state. The intermediate breakdown turns rate-limiting, with the overall Δ*G*^‡^ = 31.9 kcal/mol (272*i* cm^−1^) for N1–C40 largely outperforming Δ*G*^‡^ = 47.7 kcal/mol (391*i* cm^−1^) for N15–C12 amide. Such a large difference in kinetic requirements eliminates the possibility for the latter process and agrees with the dominance of DP1 and DP2 degradation products, which is further supported by its −2.2 kcal/mol-higher reaction exergonicity.

Interestingly, we also observed the higher feasibility of the C6–C12 cleavage once the tetrahedral intermediate is formed on the N15–C12 amide. In that case, the intermediate breaks through a lower barrier at Δ*G*^‡^ = 40.5 kcal/mol (195*i* cm^−1^) that liberates the fragment containing the benzene unit. Although this process is kinetically still highly unfeasible, a moderate yield of the final product is afforded through the pronounced exergonicity of this process (Δ*G*_R_ = −38.4 kcal/mol), which rationalizes the signal at *m*/*z* 200.0034 from DP1 following the initial hydrolysis of the N1–C40 amide.

Lastly, the efficiency of the acid-catalyzed process relies on the ability of the H_3_O^+^ cation to protonate the substrate, which improves its electrophilicity for the subsequent approach of neutral H_2_O. The amide moiety contains two potential protonation sites: the carbonyl oxygen and the nitrogen atom. Determining the preferred site has been a subject of extensive research due to its implications for chemical reactivity and molecular interactions [35]. Numerous studies support the preference for oxygen protonation. For instance, infrared (IR) spectroscopy of protonated amides shows characteristic shifts in the carbonyl stretching frequency, consistent with oxygen protonation [36]. Additionally, gas-phase proton affinity measurements indicate that the proton affinity of the oxygen site is higher than that of nitrogen [35]. Oxygen protonation results in the formation of an iminium-like cation, stabilized by resonance. The protonated oxygen increases the positive charge on the carbonyl carbon, enhancing the electrophilicity of the amide; however, all of our attempts to locate a feasible process that is initiated in this way failed, only resulting in kinetically demanding pathways. For example, the formation of a tetrahedral intermediate with protonated oxygen on the N1–C40 amide proceeds through the transition state at Δ*G*^‡^ = 50.2 kcal/mol and involves one negative vibration mode (1524*i* cm^−1^) corresponding to the proton transfer from the carbon-attached water to amide nitrogen. This offers a tetrahedral intermediate (Δ*G*_R_ = 19.5 kcal/mol) featuring a newly formed C–OH bond, with both amide oxygen and nitrogen being protonated. The latter requires only 3.8 kcal/mol to reach the next transition state to cleave the amide N–C bond and afford the final products in the exergonic fashion, Δ*G*_R_ = −24.2 kcal/mol. Yet, the high kinetic barrier of the first step of this stepwise process renders this pathway as highly unlikely.

**Figure 11:**
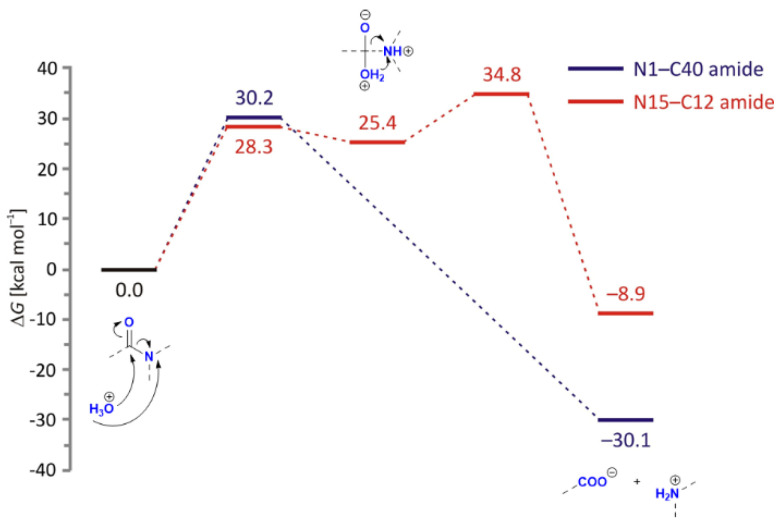
Free energy profiles for the acid-catalyzed hydrolysis of N1–C40 (in blue) and N15–C12 (in red) amide bonds in LIF, obtained using the (SMD)/B3LYP/Def2TZVPP model in water.

Instead, our DFT calculations show that all processes that are started by the excess proton transfer from H_3_O^+^ onto the amide nitrogen are much more feasible (Figure 11). This notion has already been recognized in the literature, and the fact that amide nitrogen protonation is a critical step in both acid-catalyzed [37] and enzymatic amide hydrolysis [38] was reported. The latter is based on the fact that nitrogen protonation disrupts the resonance stabilization of the amide bond and improves the leaving group ability of the amine. The mentioned enzymatic study is particularly notable for its focus on tertiary amides, such as N1–C40 here. Therefore, it is not surprising that our analysis identified a concerted mechanism of its hydrolytic cleavage, with a single transition state structure (154*i* cm^−1^) describing C–OH_2_ bond formation with a simultaneous amide breaking involving N-protonated amide. The computed barrier of Δ*G*^‡^ = 30.2 kcal/mol indicates a feasible process, while excessive exergonicity of Δ*G*_R_ = −30.1 kcal/mol further supports its viability and confirms the predominance of DP1 and DP2 in the MS spectra.

Alternatively, an analogous acid-catalyzed process investigated on a secondary N15–C12 amide turned out to be a stepwise process. It features a barrier of 28.3 kcal/mol to reach the high-energy tetrahedral intermediate that proceeds to the final products through a rate-limiting step, having Δ*G*^‡^ = 34.8 kcal/mol (129*i* cm^−1^) for a moderate reaction exergonicity of Δ*G*_R_ = −8.9 kcal/mol. These results again convincingly confirm the much higher susceptibility of a tertiary N1–C40 amide towards hydrolytic cleavage from both kinetic and thermodynamic aspects in all three cases, neutral, and acid- and base-catalyzed processes, which strongly aligns with the demonstrated prevalence of the DP1 and DP2 degradation products.

Lastly, we attempted to provide some insight into degradation mechanisms under oxidative conditions by computing the homolytic bond dissociation energies (BDEs) of all relevant bonds in LIF (Table 2). This is justified through the fact that oxidative degradations with strong oxidants, such as H_2_O_2_, involve the breakdown of organic compounds through reactions driven by the reactive oxygen species (ROS) generated from H_2_O_2_ [39]. Hydrogen peroxide is a relatively stable oxidant but can decompose to produce highly reactive species, for instance hydroxyl radicals (OH^•^), which are key to oxidative degradation. Data in Table 2 confirm the higher degradation instability of N1–C40 amide over N15–C12 as its BDE is 13.2 kcal/mol lower, which indicates an easier cleavage. The latter predominantly occurs due to higher stability of the tertiary amine radical (R_1_R_2_N1^•^) compared to the secondary analogue (HR_1_N15^•^), afforded through the increased alkyl electron donation. Since the BDE value for the C12–C6 bond, neighbouring to the latter amide, is 3.5 kcal/mol higher, it justifies only a very marginal population of degradation products with the cleaved C12–C6 bond.

Aromatic C–H cleavages are significantly more demanding than aliphatic ones. The former typically exceed 100 kcal/mol, with some differences among particular sites. The least favourable aromatic C–H bond scissions are on the furan ring, with C32–H and C33–H BDEs exceeding 112 kcal/mol. Yet, since the furan ring is frequently damaged in the identified products, our results suggest that its degradation mechanism likely involves a direct OH^•^ attachment onto the C32=C33 double bond followed by the oxidation of the formed products. The same holds for all C–H bonds within the more stable aromatic phenyl ring of either the benzofuran moiety or the one bearing the –SO_2_Me group, where high BDE values around 104 kcal/mol justify the perseverance of these structural units in most degradation products.

In contrast, BDE values for three aliphatic C–H bonds within the piperidine moiety are much lower and span a range of 11 kcal/mol, with the value for C10–H being the lowest at 70.4 kcal/mol. These results better align with the distribution of the degradation products since the processes at the saturated carbons are initiated by the C–H homolytic cleavage. In other words, the obtained trend strongly confirms the presence of the C10–OH group in DP7 and its further oxidation into a ketone in DP10 and DP11. Since the BDE for C3–H is only 6 kcal/mol higher, yet still as low as 76.4 kcal/mol, this notion could explain an analogous OH^•^ attachment to that site which is later followed by the dehydration and the C2=C3 double bond formation, as in DP8. This process is likely thermodynamically facilitated by a favourable water elimination, especially since BDE values for aliphatic C16–H and C17–H are also found at 76–78 kcal/mol; however, degradation processes are yet to be identified at those sites. The is probably due to the increased steric demands to form the potential C16=C17 double bond, and is only marginally observed as a higher degradation process in, for example, DP7 or DP5. Similarly, although several carbon–carbon bond cleavages are linked with relatively small BDE values, such as C16–C17 (37.4 kcal/mol) and C16–C24 (48.3 kcal/mol), these processes are kinetically hindered and the matching degradation products are only observed upon higher collision energies. The same holds for relatively low BDE = 78.2 kcal/mol for C17–C18 bond and, especially, BDE = 57.2 kcal/mol for C20–S cleavage that requires increased collision energies to occur. Lastly, we notice that C–Cl BDEs around 78 kcal/mol appear too high to eliminate chlorine from the aromatic unit.

### 3.7. Proposed Mechanistic Pathways of Oxidative Degradation

**Scheme 1:**
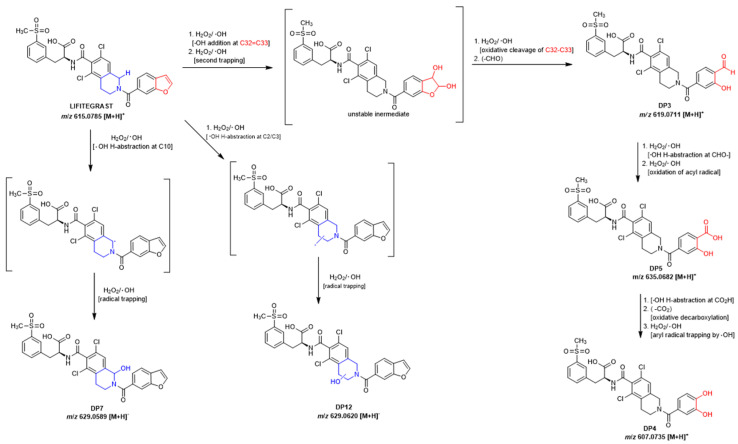
Proposed oxidative degradation pathways of LIF under H_2_O_2_ stress conditions, highlighting benzofuran-ring cleavage and piperidine-ring hydroxylation.

The oxidative degradation of LIF under hydrogen peroxide stress conditions is best explained by hydroxyl radical attack at the most labile positions of both the piperidine and benzofuran moieties (Scheme 1).

At the piperidine ring, homolytic C–H cleavage is initiated at C10–H, which exhibits the lowest calculated BDE (70.4 kcal/mol), leading to hydroxylated DP7. In parallel, ·OH abstraction may also occur at C2–H or C3–H, where the BDEs remain comparably low (C3–H: 76.4 kcal/mol; C2–H: 81.7 kcal/mol). While the lower BDE suggests a higher likelihood of radical initiation at C3–H, abstraction at C2–H cannot be excluded. Both scenarios converge to the experimentally detected hydroxylated product DP12 through radical trapping, consistent with literature data showing that hydroxyl radicals initiate piperidine oxidation via C–H abstraction on α-carbons adjacent to nitrogen [40]. DP12 was observed only in low abundance, consistent with its absence in earlier degradation studies and its susceptibility to further oxidation. Due to the insufficient yield, NMR characterization could not be performed. MS/MS fragmentation, however, confirmed hydroxylation on the piperidine ring via diagnostic H_2_O loss and double bond formation, though the exact position (C2–H vs. C3–H) could not be unambiguously assigned.

The benzofuran moiety undergoes attack at its most electrophilic site, the C32=C33 double bond. Direct ·OH addition generates an unstable 1,2-diol intermediate, which readily undergoes oxidative cleavage with elimination of a formaldehyde unit to yield aldehyde DP3. Further oxidation of DP3 gives the corresponding carboxylic acid DP5, in agreement with literature reports describing benzofuran oxidations with H_2_O_2_ in the presence of Mn(III)-porphyrins, often via epoxide intermediates ultimately affording aldehydes and carboxylic acids [41]. Formation of the dihydroxylated derivative DP4 can be rationalized by ·OH-mediated oxidative decarboxylation of DP5, generating an aryl radical that undergoes ·OH trapping on the aromatic ring. While an alternative route via a transient diol intermediate followed by the elimination of two formaldehyde units cannot be entirely excluded, such a process would require two consecutive C–C scissions and thus appears less plausible under the applied non-catalytic H_2_O_2_ stress conditions. Our mechanistic proposal therefore prioritizes the DP5→decarboxylation pathway as the dominant route to DP4.

To the best of our knowledge, mechanistic descriptions of LIF degradation are absent from the literature. Previous studies have mainly focused on formulation stability and analytical identification of degradation products [13,15,16], while patents emphasized antioxidant stabilization without mechanistic insight.

### 3.8. In Silico Toxicology Evaluation of DP5 and DP7

Since DP5 and DP7 were observed in initial, extended, and targeted oxidative forced degradation studies, they were chosen for in silico toxicity evaluation. They are both structurally similar to LIF, with DP5 having an identical core but hydroxybenzoic functionality instead of benzofuran and DP7 having benzofuran functionality but a slightly modified core (hydroxyl group at position C10). Since no experimental data were available for them in the public domain, toxicity was assessed in silico based on their structural similarity to the LIF. Relevant data on LIF has been extracted from available documents produced by the EMA [42] and FDA [43].

In silico predictions indicated no potential for bacterial mutagenicity or chromosomal damage for either degradation product. DP5 was predicted to have potential nephrotoxicity due to the salicylic acid moiety, though this was deemed negligible given the low dose and expected minimal uptake, which is consistent with bioavailability data for LIF. The presence of a phenethylbenzamide substructure in both molecules indicated potential agonistic activity at the glucocorticoid receptor, as seen in LIF. This was predicted by Derek Nexus [22] software, which also predicted a comparable potential for teratogenicity, with a lowest observed adverse effect level (LOAEL) of 3 mg/kg bw/day. For other toxicity endpoints, both degradation products are expected to exhibit toxicity levels comparable to or lower than those of LIF.

Other data on LIF [42,43] provided further insights. In dogs and rabbits exposed to LIF via topical ocular administration, only mild ocular irritation, such as squinting and blinking, was observed. A no-observed-adverse-effect level (NOAEL) of 5.25 mg/eye/day or 10.5 mg/day (based on a 35 μL drop volume) was established. In human studies, ocular adverse events were primarily limited to instillation site irritation and reaction following exposure to 10 mg LIF/day. Eye irritation and pain were reported with incidences of 16% and 15%, respectively, while dysgeusia was the most common non-ocular adverse event. None of these effects were clinically significant, and no systemic effects were observed in animals or humans.

The NOAEL of 10 mg/day, supported by similar effects across species and the relevance of human data, is proposed for systemic toxicity assessment and permitted daily exposure (PDE) derivation for both degradation products.

## 4. Conclusions

In this study, we investigated the degradation behaviour of LIF under a variety of conditions ranging from standard stress scenarios to more extreme environments. Three consistent degradation sites of LIF were identified: the amide bond linking the piperidine and benzofuran rings is prone to hydrolysis, while both of these rings are sensitive to oxidation. Computational DFT analysis provided critical mechanistic insights into these pathways. The inter-ring amide bond’s higher susceptibility to hydrolysis compared to the other amide was attributed to the stabilizing effect of a nearby carboxylate group on the latter, which increases the kinetic barrier through charge repulsions and hydrogen bonding under both neutral and base-catalyzed scenarios. For oxidative degradation, bond dissociation energy calculations revealed that the C10–H bond in the piperidine ring is the most vulnerable to radical-mediated cleavage, supporting the formation of DP7, DP10, and DP11, while the benzofuran ring’s degradation proceeds via hydroxyl radical addition to its double bond, consistent with products such as DP3, DP4, and DP5.

In total, twelve degradation products were identified, of which three were previously known and nine were newly discovered, and all were characterized using MS and/or NMR spectroscopy. The structure elucidation of DP7 posed significant challenges due to its low abundance and discrepancies with earlier reports that conflicted with our interpretation of the experimental data. Based on the spectroscopic evidence, we confirmed that DP7 possesses a hydroxyl group at position C10, indicating that the compound previously reported as DP3 in reference [16] is structurally identical and should be revised accordingly.

Additionally, DP5 and DP7, impurities observed in initial, extended, and targeted oxidative forced degradation, were selected for in silico toxicity assessment. This indicated that both are expected to have toxicity profiles comparable to or lower than LFG.

These findings contribute to understanding the greater picture of the compound’s stability and will help in the development of safer drug formulations for medicinal use.

## Figures and Tables

**Table 1:** LIF degradation products discovered by LC-HRMS after extended initial oxidative degradation with 3% H_2_O_2_ for 30 days.

	RT/min	Ion	Obs. *m*/*z*	Calc. *m*/*z*	Error/ppm	Suggested Structure
LIF	15.27	[M-H]^−^	613.0597	613.0609	−1.96	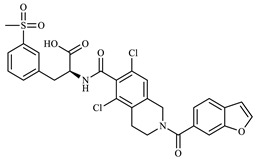
		[M+H]^+^	615.0785	615.0754	5.04	
DP7	14.31	[M-H]^−^	629.0589	629.0558	4.93	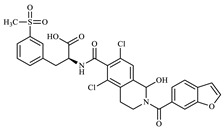
DP3	13.86	[M-H]^−^	617.0581	617.0558	3.73	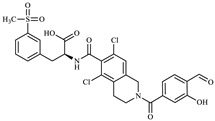
		[M+H]^+^	619.0711	619.0703	1.29	
DP4	12.06	[M-H]^−^	605.0543	605.0558	−2.48	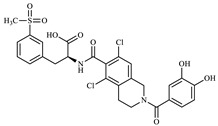
		[M+H]^+^	607.0735	607.0703	5.27	
DP5	11.30	[M-H]^−^	633.0549	633.0507	6.63	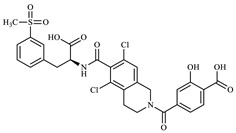
		[M+H]^+^	635.0682	635.0652	4.72	
DP8	10.51	[M-H]^−^	483.0208	483.0190	3.73	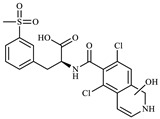
		[M+H]^+^	485.0357	485.0335	4.54	
DP1	8.66	[M-H]^−^	469.0383	469.0397	−2.98	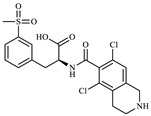
		[M+H]^+^	471.0561	471.0543	3.82	

**Table 2:** Computed homolytic bond dissociation energies (BDEs) at the UB3LYP/Def2TZVPP level of theory (all values in kcal/mol, atom numbering consistent with Figure 1).

Structure	Bond	BDE	Bond	BDE	Bond	BDE
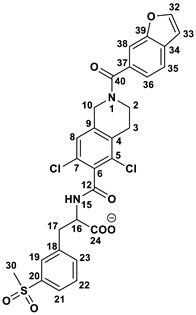	N1–C40	60.1	N15–C16	66.6	C23–H	103.1
	C2–H	81.7	C16–H	75.6	C30–H	95.1
	C3–H	76.4	C16–C24	48.3	C32–H	112.6
	C5–Cl	77.1	C16–C17	37.4	C33–H	112.1
	C6–C12	76.8	C17–H	77.9	C35–H	104.8
	C7–Cl	78.7	C17–C18	78.2	C36–H	103.5
	C8–H	105.8	C19–H	103.7	C37–C40	76.6
	C10–H	70.4	C20–S	57.2	C38–H	106.3
	C12–N15	73.3	C21–H	104.6		
	N15–H	98.5	C22–H	103.6		

## Data Availability

All data is available through Supplementary Material.

## Supplementary Materials

The following supporting information can be downloaded at: https://www.mdpi.com/article/10.3390/pharmaceutics17101299/s1. Supplementary Materials contains experimental conditions for degradation, full analytical characterisation of each identified compound, procedure for isolation of degradation products, MS spectra and fully assigned NMR spectra with extracted proton and carbon chemical shifts.

## Abbreviations

The following abbreviations are used in this manuscript:

API	Active pharmaceutical ingredient
BDE	Bond dissociation energies
CREST	Conformer-rotamer ensemble sampling tool
DFT	Density functional theory
DP	Degradation product
EMA	European Medicines Agency
ESI	Electrospray ionization
FDA	U.S. Food and Drug Administration
HMBC	Heteronuclear multiple bond correlation
HRMS	High-resolution mass spectrometry
ICAM-1	Intercellular adhesion molecule
ICH	The International Council for Harmonisation of Technical Requirements for Pharmaceuticals for Human Use
IR	Infrared spectroscopy
IRC	Intrinsic reaction coordinate
LC	Liquid chromatography
LFA-1	Lymphocyte function-associated antigen 1
LIF	Lifitegrast
LOAEL	Lowest observed adverse effect level
NMR	Nuclear magnetic resonance
NOAEL	No-observed-adverse-effect level
PDE	Permitted daily exposure
QSAR	Quantitative structure–activity relationship
RH	Relative humidity
RT	Room temperature
