# Supplementary material for: Lifitegrast Degradation: Products and Pathways
Source: Pharmaceutics. 2025 Oct 4;17(10):1299. doi: 10.3390/pharmaceutics17101299 (PMC12567358; doi:10.3390/pharmaceutics17101299)
Supplement: Supplementary file 1 [file pharmaceutics-17-01299-s001.zip › pharmaceutics-3879422-supplementary.pdf]

Supplementary data

Lifitegrast degradation: products and pathways

Leo Štefan[a], Ivan Sušanj[a], Jadranka Buljević[a], Marin Roje[b], Mladenka Jurin[b], Anđela Buljan[b], Tamara Rinkovec[c], Robert Vianello[c], Marijana Pocrnić[d], Nives Galić[d] and Ana Čikoš[e]*

[a]*JGL d.d. Jadran Galenski Laboratorij, 51000 Rijeka, Croatia*

[b]*Laboratory for Chiral Technologies, Division of Organic Chemistry and Biochemistry, Ruđer Bošković Institute, 10000 Zagreb, Croatia*

[c]*Laboratory for the Computational Design and Synthesis of Functional Materials, Division of Organic Chemistry and Biochemistry, Ruđer Bošković Institute, 10000 Zagreb, Croatia*

[d]*Department of Chemistry, Faculty of Science, University of Zagreb, 10000 Zagreb, Croatia*

[e]*NMR Centre, Ruđer Bošković Institute, 10000 Zagreb, Croatia*

Corresponding author: ana.cikos@irb.hr

Table of Contents

Lifitegrast degradation: products and pathways *1*

S1. *Initial forced degradation* *4*

S2. *Extended forced degradation* *6*

S3. *Analysing oxidative forced degradation mixture (3% H_2O_2, 50 °C/75% RH, 30 days)* *8*

S3.1. **NMR analysis** **8**

S3.1.1. Lifitegrast 8

S3.1.2. DP1 13

S3.1.3. DP2 18

S3.1.4. DP3, DP4, DP5 and DP6 in the oxidative degradation mixture 23

S3.2. **MS analysis** **27**

S3.2.1. Lifitegrast 28

S3.2.2. MS chromatograms of oxidative degradation mixture 33

S3.2.3. Lifitegrast 33

S3.2.4. DP1 34

S3.2.5. DP3 37

S3.2.6. DP4 39

S3.2.7. DP5 41

S3.2.8. DP7 43

S3.2.9. DP8 45

S4. *Extreme oxidative degradation* *47*

S4.1. **NMR analysis** **48**

S4.1.1. DP9 (fraction 1) 48

S4.1.2. DP10 (fraction 3) 52

S4.1.3. DP11 (fraction 2) 56

S4.2. **LC-UV/MS analysis** **60**

S4.2.1. Fraction 1 (DP9) 61

S4.2.2. Fraction 2 (DP11) 62

S4.2.3. Fraction 3 (DP10 and DP11) 62

S5. *Targeted oxidative degradation* *63*

S5.1. **MS analysis** **63**

S5.1.1. DP5 66

S5.1.2. DP12 69

S5.1.3. DP7 73

S6. *Comparison of isolated DP7 to purchased analytical standard of the same structure* *76*

S6.1. **HRMS and MS/MS analysis** **76**

S6.2. **NMR analysis** **80**

S7. *Quantitative context for degradation kinetics of DP5 and DP7* *85*

S8. *Single-point energy calculations employing different DFT functionals* *86*

S9. *In silico toxicity scope* *89*

S10. *List of Tables and Figures* *90*

S1. Initial forced degradation

Lifitegrast API samples were tested in both solid and dissolved forms. For solid-state testing, Lifitegrast (0.1 g) was weighed into three separate stability test containers. For solution-based stability testing, seven samples (0.05 g each) were weighed into amber-colored vials. These samples were subjected to various stress conditions to evaluate the effects of temperature, relative humidity, and light irradiation, as well as stability in aqueous media, (neutral, acidic and basic), oxidative conditions using hydrogen peroxide (H_2O_2) and the radical initiator azobisisobutyronitrile (AIBN). The pH of the aqueous solutions was adjusted using NaOH or HCl, and dissolution of Lifitegrast was aided by sonication in an ultrasonic bath. Samples were collected after seven days, and concentrations were adjusted to 5% for analysis.

Table S1. Sample preparation for the initial forced degradation study

Sample Label	Condition	Weight	Volume (Solvent)
1	50 °C	0.1 g	/
2	50 °C/75% RH	0.1 g	/
3	25 °C/45% RH (photo)	0.1 g	/
4	Aqueous solution pH=7; photo	0.05 g	17 ml (9 ml aqueous solution + 8 ml ACN)
5	0.1 N HCl; 50 °C/75% RH	0.05 g	17 ml (9 ml HCl + 8 ml ACN)
6	Aqueous solution; 50 °C/75% RH	0.05 g	17 ml (9 ml aqueous solution + 8 ml ACN)
7	0.1 N NaOH; 50 °C/75% RH	0.05 g	17 ml (9 ml NaOH + 8 ml ACN)
8	3% H_2O_2; 50 °C/75% RH	0.05 g	17 ml (9 ml 3% H_2O_2 + 8 ml ACN)
9	Aqueous solution pH=7 with AIBN; 50 °C/75% RH	0.05 g	17 ml (9 ml aqueous solution + 8 ml ACN)
10	50 °C/75% RH (control)	0.05 g	17 ml (ACN)

Table S2. Experimental UPLC-UV parameters for initial forced degradation study

Instrument	Waters
Flow rate	0.4 mL/min
Detector	Waters PDA (Resolution: 4.8 nm), λ = 210 nm
Injection volume	3 µL
Autosampler temperature	20 °C
Column	ACQUITY UPLC HSS T3 (2.1 × 100 mm, 1.8 µm)
Column specifications	Code: RA-KT3-HSS-98(4); Part No.: 186003539; Serial No.: 02783220915115; Lot No.: 0278322091
Manufacturer	Waters
Column temperature	40 °C

Mobile phase	Phase A: 20 mM ammonium formate: acetonitrile (99:1 v/v)
	Phase B: 20 mM ammonium formate: acetonitrile (20:80 v/v)
Chromatography time	30 min

Table S3. Results of initial forced degradation study

ID	Conditions	Individual impurities	Total impurities
1	50 °C	DP2 (0.09%)	0.4%
2	50 °C/75% RH	DP1 (0.1%), DP2 (0.1%)	0.2%
3	25 °C/45% RH, photo	DP2 (0.1%)	2.2%
4	Water, pH=7; photo	DP1 (0.1%), DP2 (0.1%)	0.6%
5	0.1 N HCl; 50 °C/75% RH	DP1 (2.6%), DP2 (0.8%)	3.5%
6	Water; 50 °C/75% RH	DP1 (0.1%), DP2 (0.1%)	0.2%
7	0.1 N NaOH; 50°C/75% RH	DP1 (22.5%), DP2 (6.8%)	29.7%
8	3% H_2O_2; 50°C/75% RH	DP1 (0.7%), DP2 (2.1%) Unknown degradants	13.8%
9	Water, pH=7, AIBN, 50 °C/75% RH	DP1 (0.4%), DP2 (2.3%) Unknown degradants	78.5%
10	acetonitrile, 50 °C/75% RH	DP2 (0.1%)	0.1%

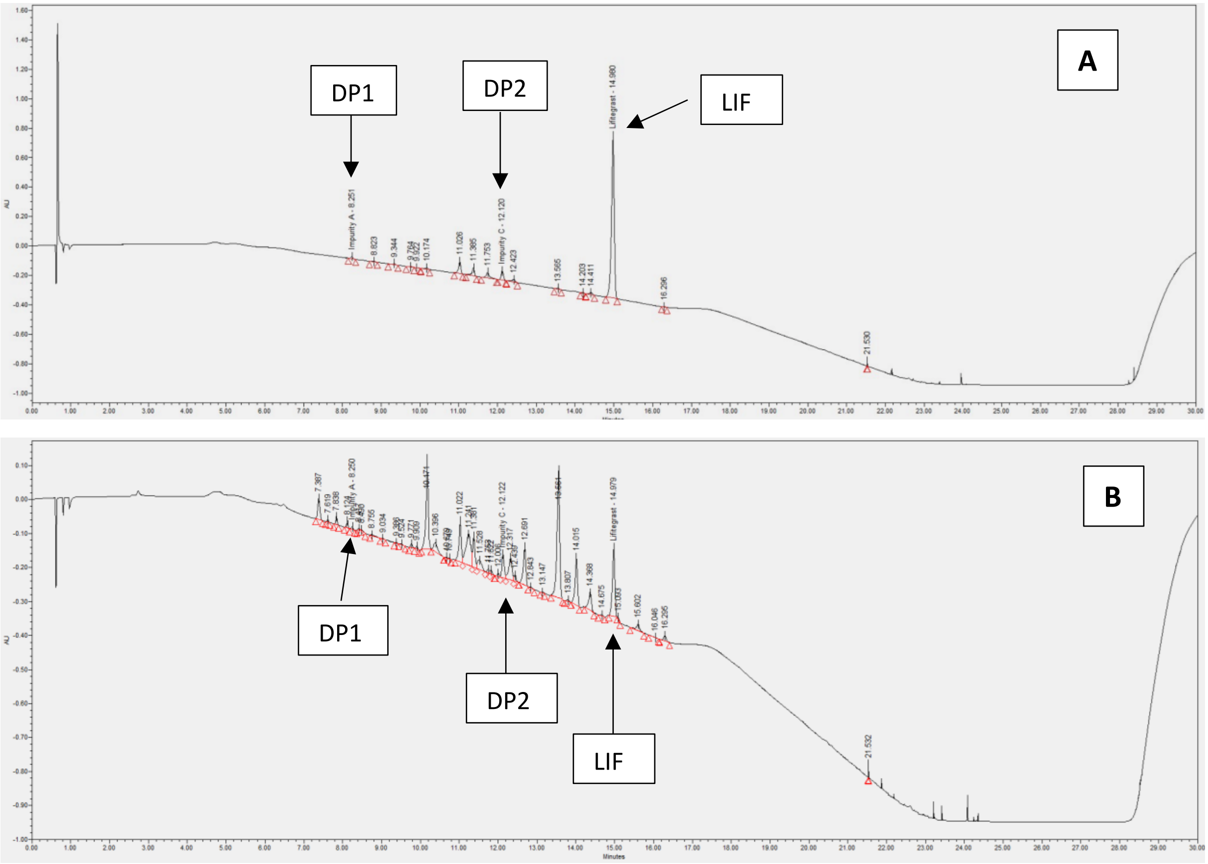

Fig. S1. UPLC-UV chromatograms after initial degradations of Lifitegrast in solution: (A) 3% H_2O_2; 50 °C/75% RH and (B) Water, pH=7, AIBN, 50 °C/75% RH after 7 days

S2. Extended forced degradation

Extended forced degradation was conducted to determine the timeframe required to enhance the degradation of Lifitegrast. These studies were carried out under varying temperatures and relative humidity conditions (25 °C/60% RH, 40 °C/75% RH, and 50 °C/75% RH). Samples were evaluated at predefined time points of 0, 15, and 30 days.

Table S4. Sample preparation for extended forced degradation

Condition (25 °C/60% RH, 40 °C/75% RH, 50 °C/75% RH; T0, T15, T30)	Weight (Lifitegrast)	Volume (Solvent)
**3% H_2O_2**	0.05 g	17 ml (9 mL H_2O_2 + 8 ml ACN)
**Aqueous solution pH=7 with AIBN**	0.05 g	17 ml (9 ml pH=7 solution + 8 ml ACN)

Table S5. Results of extended forced degradation (H_2O_2 and AIBN)

Conditions	T15	T30
3% H_2O_2, 25 °C/60% RH	DP1 (0.3%), DP2 (1.4%), Total: 3.1%	DP1 (0.2%), DP2 (0.3%), Total: 8.7%
3% H_2O_2, 40 °C/75% RH	DP1 (1.0%), DP2 (1.1%), Total: 9.6%	DP1 (1.0%), DP2 (0.4%), Total: 34.9%
3% H_2O_2, 50 °C/75% RH	DP1 (0.2%), DP2 (1.1%), Total: 20.9%	DP1 (0.1%), DP2 (0.8%), Total: 30.1%
AIBN, 25 °C/60% RH	DP2 (<1%), Total: 7.5%	DP1 (0.1%), DP2 (0.5%), Total: 11.1%
AIBN, 40 °C/75% RH	DP1 (0.1%), DP2 (1.1%), Total: 28.5%	DP1 (0.2%), DP2 (1.4%), Total: 36.1%
AIBN, 50 °C/75% RH	DP1 (0.2%), DP2 (1.6%), Total: 47.8%	DP1 (0.3%), DP2 (1.7%), Total: 48.9%

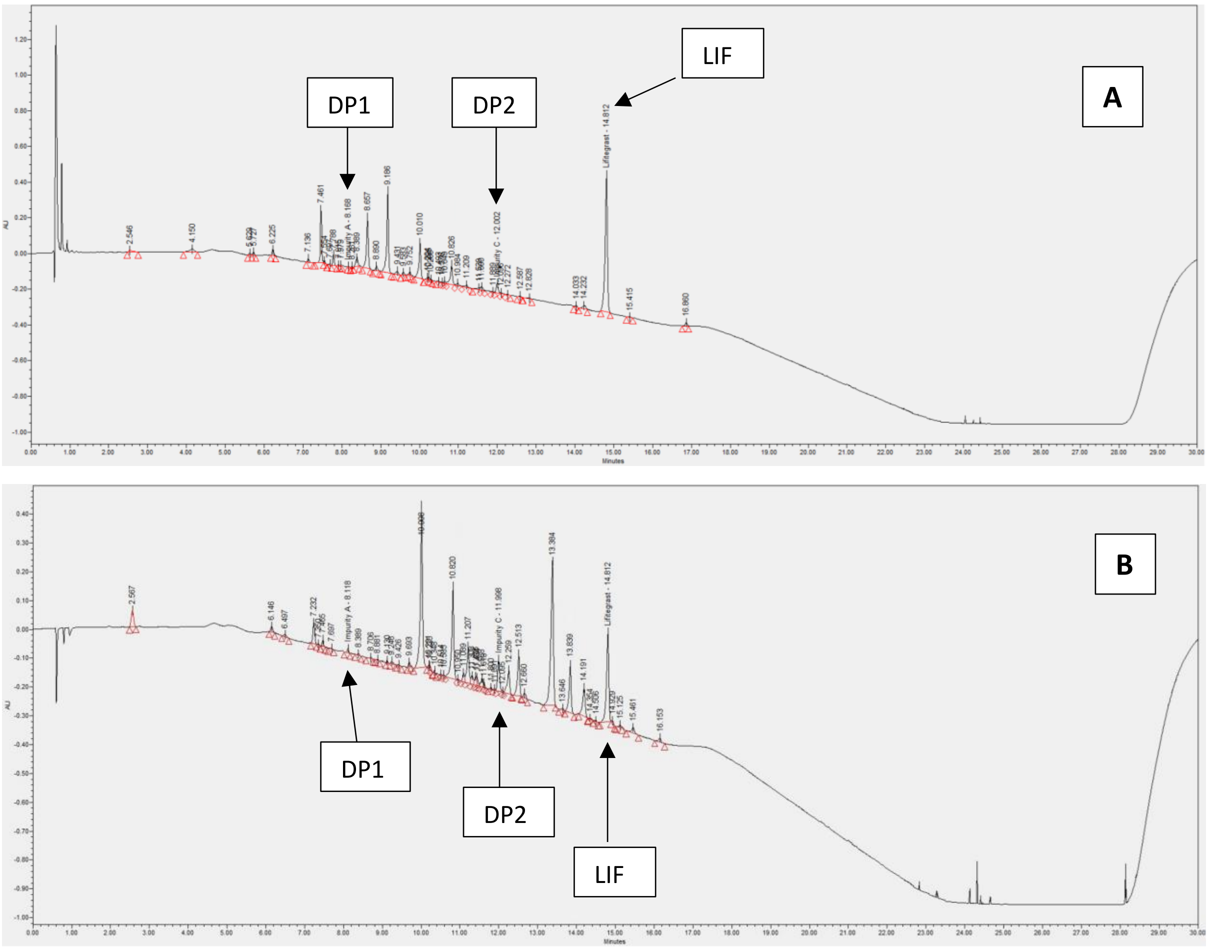

Fig. S2. UPLC-UV chromatograms after extended degradations of Lifitegrast in solution: (A) 3% H_2O_2; 50 °C/75% RH and (B) Water, pH=7, AIBN, 50 °C/75% RH after 30 days

S3. Analysing oxidative forced degradation mixture (3% H_2O_2, 50 °C/75% RH, 30 days)

S3.1. NMR analysis

S3.1.1. Lifitegrast

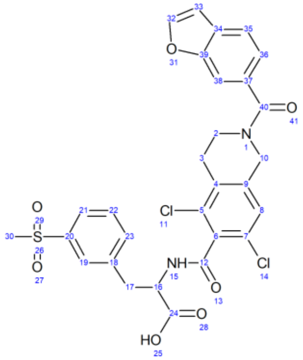

Shift1 (ppm)	H's	Type	J (Hz)	Atom1	Multiplet1
2.81	2.000	t	6.1	3	M01
3.11	1.000	dd	14.3, 10.3	17<">	M02
3.12	3.000	s	-	30	M05
3.32	2.000	dd	14.3, 5.1	17<'>	M03
3.76	2.000	br t	5.7	2	M04
4.73	2.000	s	-	10	M06
4.83	1.000	ddd	9.5, 8.4, 5.1	16	M07
7.01	1.000	dd	2.2, 1.1	33	M08
7.31	1.000	br s	-	8	M09
7.32	1.000	dd	8.1, 1.5	36	M18
7.56	1.000	t	8.4	22	M10
7.66	1.000	s	-	38	M17
7.67	2.000	dt	6.4, 1.5	23	M11
7.74	1.000	d	8.1	35	M12
7.77	1.000	dt	7.7, 1.5	21	M13
7.88	1.000	t	1.7	19	M14
8.05	1.000	d	2.2	32	M15
8.73	1.000	d	8.1	15	M16

^{1}H NMR (600 MHz, DMSO-d_6): δ = 2.81 (t, *J* = 6.1 Hz, 2 H, 3), 3.11 (dd, *J* = 14.3, 10.3 Hz, 1 H, 17<">), 3.12 (s, 3 H, 30), 3.32 (dd, *J* = 14.3, 5.1 Hz, 2 H, 17<'>), 3.76 (br t, *J* = 5.7 Hz, 2 H, 2), 4.73 (s, 2 H, 10), 4.83 (ddd, *J* = 9.5, 8.4, 5.1 Hz, 1 H, 16), 7.01 (dd, *J* = 2.2, 1.1 Hz, 1 H, 33), 7.31 (br s, 1 H, 8), 7.32 (dd, *J* = 8.1, 1.5 Hz, 1 H, 36), 7.56 (t, *J* = 8.4 Hz, 1 H, 22), 7.67 (dt, *J* = 6.4, 1.5 Hz, 2 H, 23), 7.66 (s, 1 H, 38), 7.74 (d, *J* = 8.1 Hz, 1 H, 35), 7.77 (dt, *J* = 7.7, 1.5 Hz, 1 H, 21), 7.88 (t, *J* = 1.7 Hz, 1 H, 19), 8.05 (d, *J* = 2.2 Hz, 1 H, 32), 8.73 (d, *J* = 8.1 Hz, 1 H, 15) ppm

Solvent	**DMSO-d6**
Pulse Sequence	**zg30**
Temperature (degree C)	**80.500**
Acquisition Time (sec)	**1.3631**
Number of Transients	**64**
Points Count	**32768**
Sweep Width (Hz)	**12018.86**
Spectrum Offset (Hz)	**5401.1729**
Original Points Count	**16384**
Nucleus	**1H**
Frequency (MHz)	**600.1300**

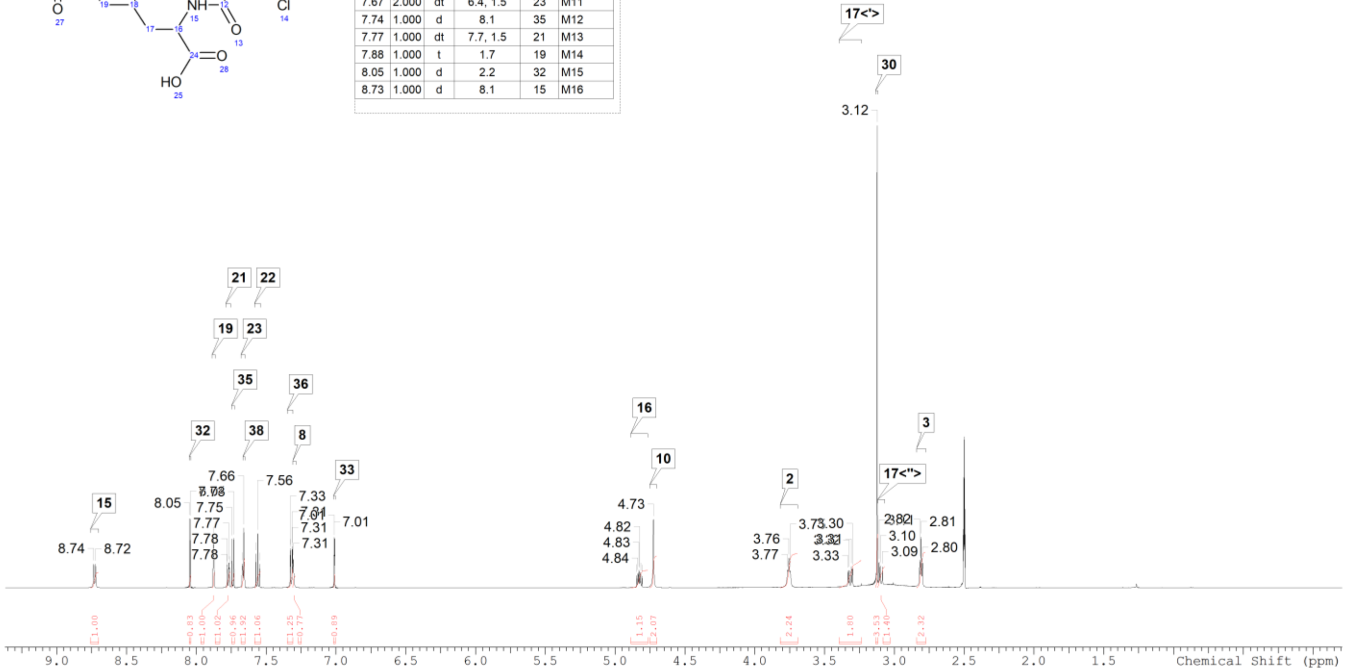

Fig. S3. Fully assigned ^{1}H spectrum of Lifitegrast in DMSO-d_6 at 80 °C

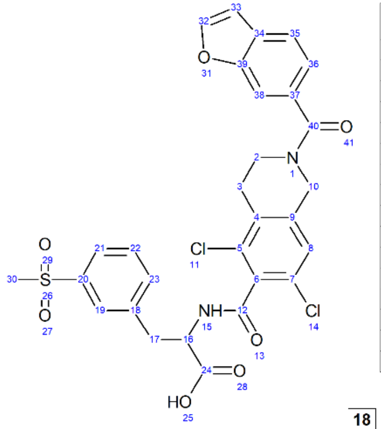

Shift1 (ppm)	C's	Type	Atom1	Multiplet1	Shift1 (ppm)	C's	Type	Atom1	Multiplet1
26.43	1.000	s	3	M01	131.28	1.000	s	5	M15
36.63	1.000	s	17	M02	131.76	1.000	s	4	M16
42.02	1.000	s	2	M28	131.84	1.000	s	37	M17
43.76	1.000	s	30	M03	134.29	1.000	s	23	M18
47.27	1.000	s	10	M29	134.72	1.000	s	6	M19
53.14	1.000	s	16	M04	137.05	1.000	s	9	M20
106.71	1.000	s	33	M05	139.16	1.000	s	18	M21
110.15	1.000	s	38	M06	141.01	1.000	s	20	M22
121.32	1.000	s	35	M07	147.46	1.000	s	32	M23
121.87	1.000	s	36	M08	153.79	1.000	s	39	M24
124.87	1.000	s	21	M09	163.47	1.000	s	12	M25
125.62	1.000	s	8	M10	169.49	1.000	s	40	M26
127.53	1.000	s	19	M11	171.65	1.000	s	24	M27
128.59	1.000	s	34	M12					
128.65	1.000	s	7	M13					
129.12	1.000	s	22	M14					

^{13}C NMR (151 MHz, DMSO-d_6): δ = 26.4 (3), 36.6 (17), 42.0 (2), 43.8 (30), 47.3 (10), 53.1 (16), 106.7 (33), 110.2 (38), 121.3 (35), 121.9 (36), 124.9 (21), 125.6 (8), 127.5 (19), 128.6 (34), 128.6 (7), 129.1 (22), 131.3 (5), 131.8 (4), 131.8 (37), 134.3 (23), 134.7 (6), 137.0 (9), 139.2 (18), 141.0 (20), 147.5 (32), 153.8 (39), 163.5 (12), 169.5 (40), 171.7 (24) ppm

Solvent	DMSO-d6
Pulse Sequence	deptqgpsp .2
Temperature (degree C)	80.200
Acquisition Time (sec)	0.5999
Number of Transients	2048
Points Count	32768
Sweep Width (Hz)	39368.88
Spectrum Offset (Hz)	15014.3535
Original Points Count	23619
Nucleus	13C
Frequency (MHz)	150.9029

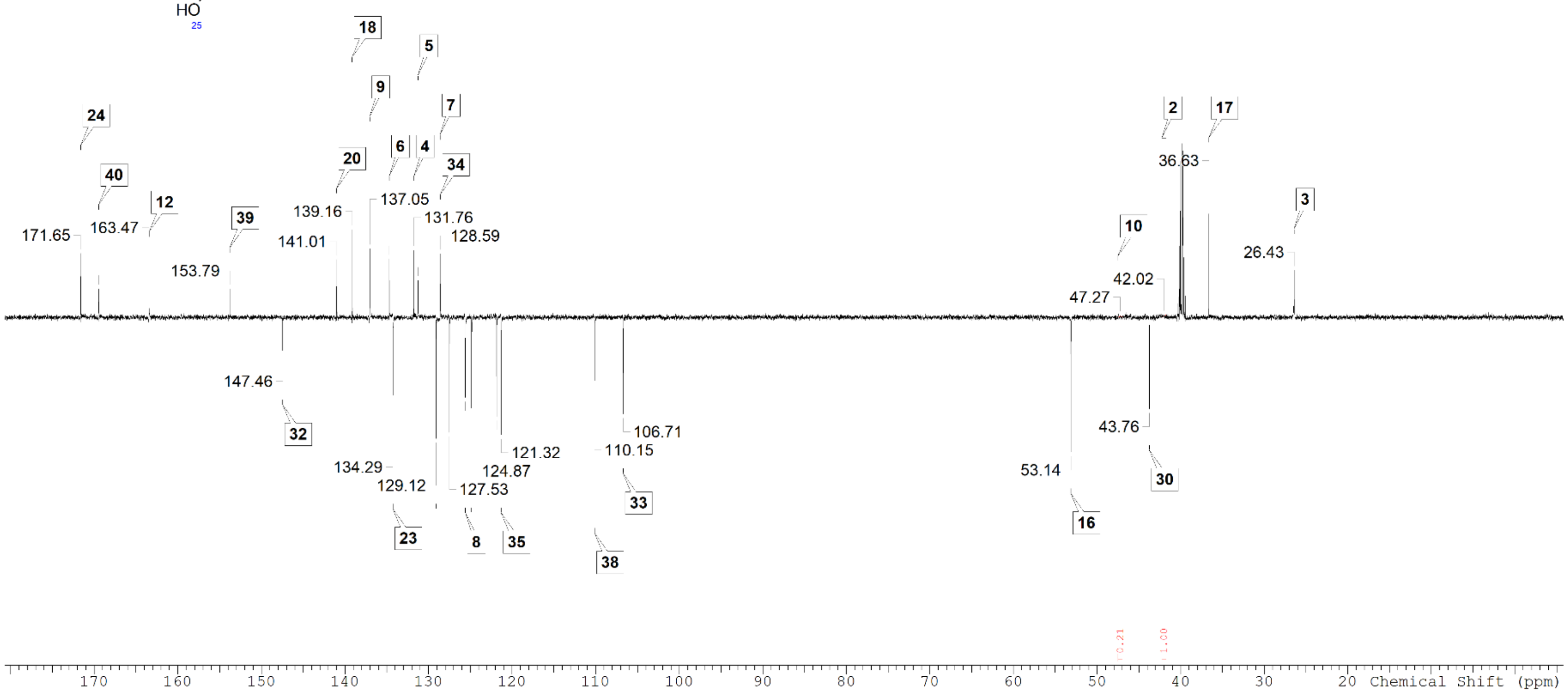

Fig. S4. Fully assigned ^{13}C spectrum of Lifitegrast in DMSO-d_6 at 80 °C

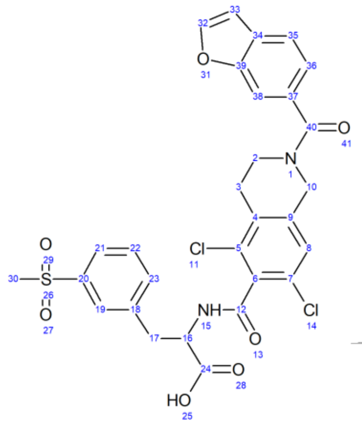

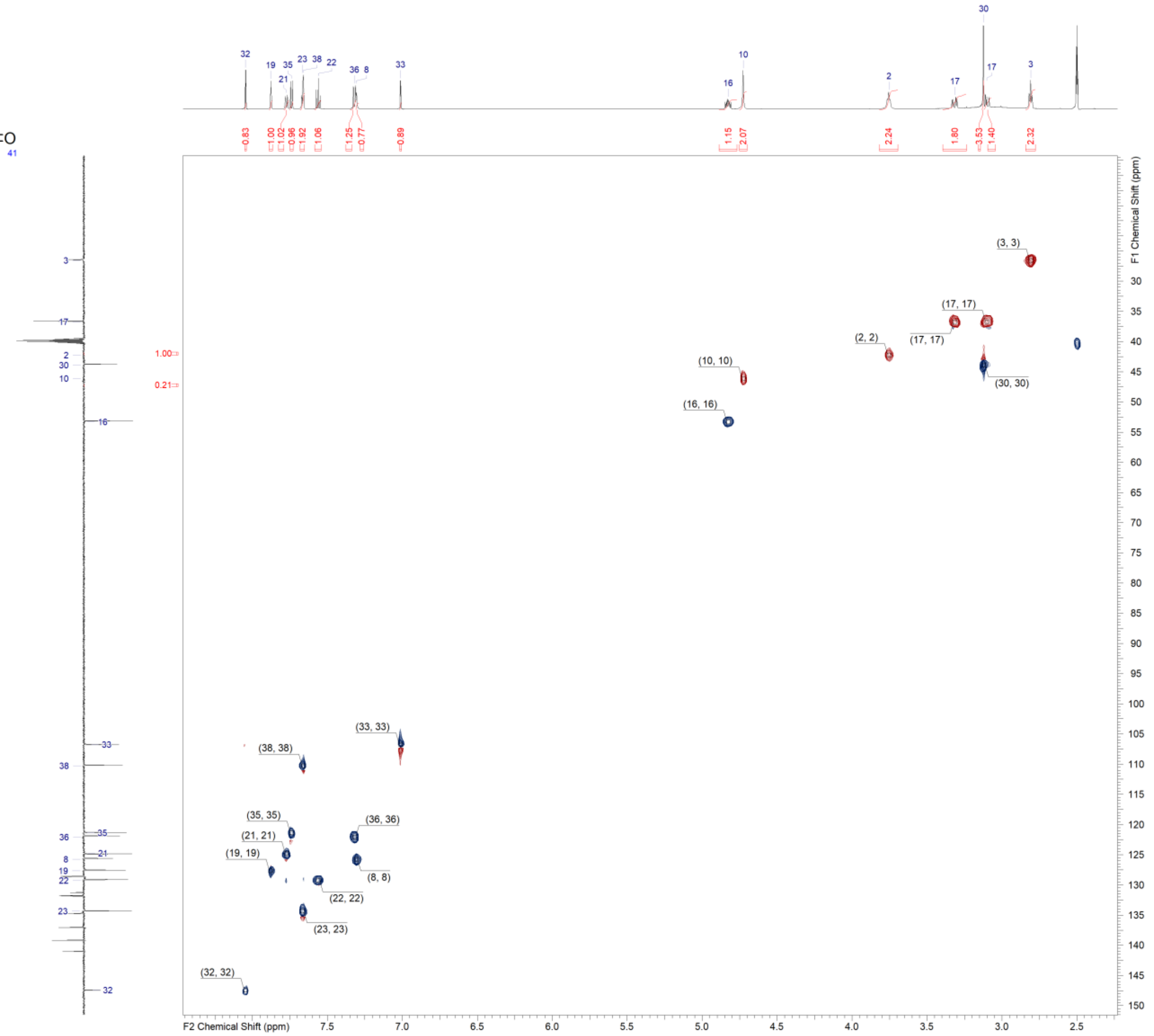

Acquisition Time (sec)	(0.1139, 0.0094)
Date	03 Apr 2023 18:45:44
Frequency (MHz)	(600.1300, 150.9029)
Nucleus	(1H, 13C)
Original Points Count	(1024, 256)
Points Count	(1024, 1024)
Pulse Sequence	hsqcedetgp
Solvent	DMSO-d6
Sweep Width (Hz)	(8984.02, 27138.29)
Number of Transients	24
Spectrum Type	HSQC-DEPT
Temperature (degree C)	80.200

Fig. S5. Fully assigned ^{1}H-^{1}H COSY spectrum of Lifitegrast in DMSO-d_6 at 80 °C

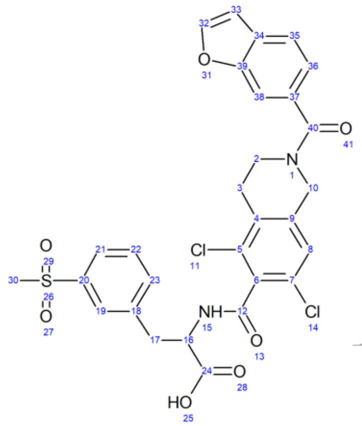

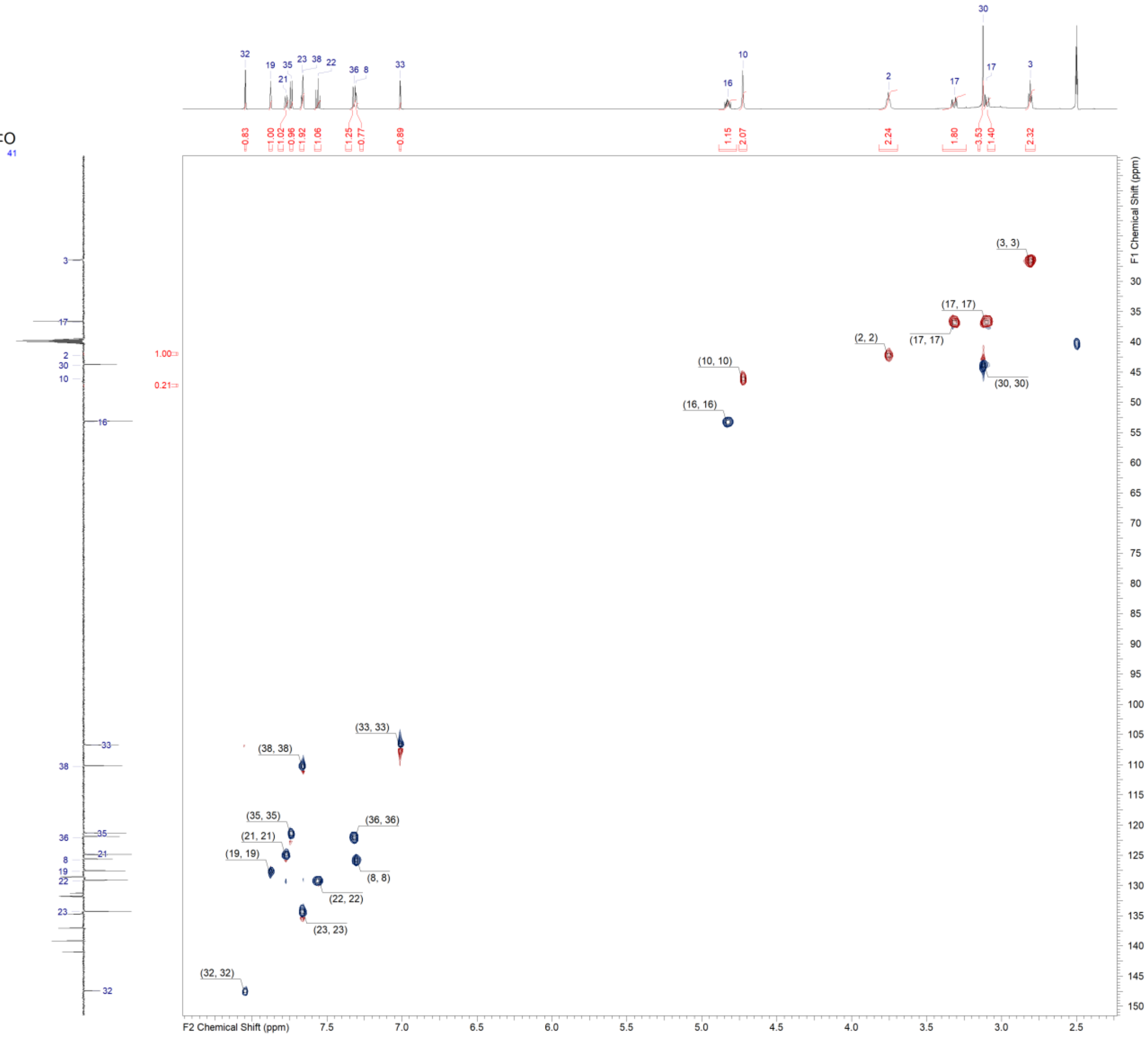

Acquisition Time (sec)	(0.1139, 0.0094)
Date	03 Apr 2023 18:45:44
Frequency (MHz)	(600.1300, 150.9029)
Nucleus	(1H, 13C)
Original Points Count	(1024, 256)
Points Count	(1024, 1024)
Pulse Sequence	hsqcedetgp
Solvent	DMSO-d6
Sweep Width (Hz)	(8984.02, 27138.29)
Number of Transients	24
Spectrum Type	HSQC-DEPT
Temperature (degree C)	80.200

Fig. S6. Fully assigned ^{1}H-^{13}C HSQCe spectrum of Lifitegrast in DMSO-d_6 at 80 °C

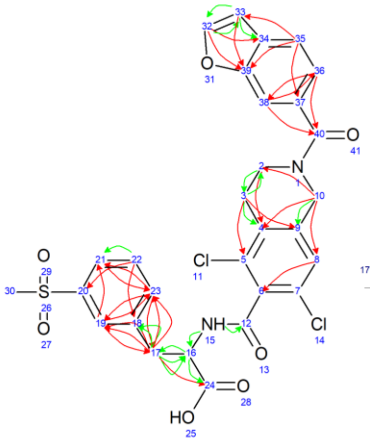

Acquisition Time (sec)	(0.1139, 0.0071)
Date	03 Apr 2023 19:49:56
Frequency (MHz)	(600.1300, 150.9029)
Nucleus	(1H, 13C)
Original Points Count	(1024, 256)
Points Count	(2048, 1024)
Pulse Sequence	hmbcgplpndqf
Solvent	DMSO-d6
Sweep Width (Hz)	(8988.42, 36185.27)
Constant (Hz)	10.0
Number of Transients	32
Spectrum Type	HMBC
Temperature (degree C)	80.000

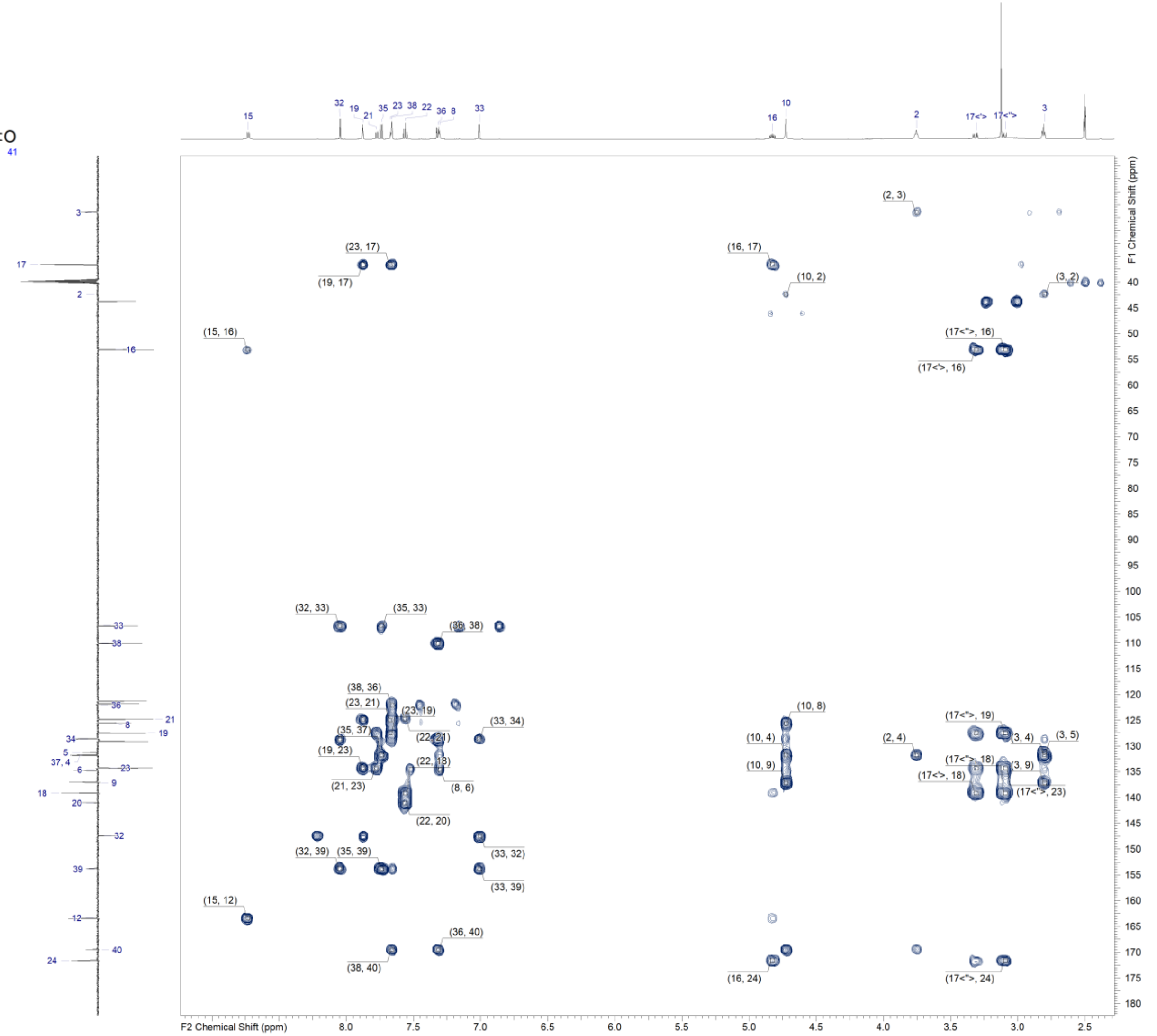

Fig. S7. Fully assigned ^{1}H-^{13}C HMBC spectrum of Lifitegrast in DMSO-d_6 at 80 °C

S3.1.2. DP1

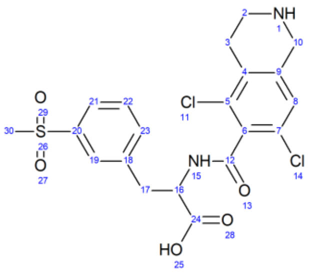

Shift1 (ppm)	H's	Type	J (Hz)	Atom1	Multiplet1
2.57	2.000	t	6.6	3	M02
3.00	2.000	t	6.1	2	M03
3.04	1.000	dd	13.9, 9.5	17<">	M14
3.14	3.000	s	-	30	M04
3.28	1.000	dd	15.0, 4.4	17<'>	M05
3.87	2.000	s	-	10	M06
4.66	1.000	td	8.5, 5.0	16	M07
7.18	1.000	s	-	8	M08
7.55	1.000	t	7.7	22	M09
7.66	1.000	dt	7.7, 1.0	23	M10
7.75	1.000	dt	7.7, 1.0	21	M11
7.85	1.000	t	1.6	19	M12
8.73	1.000	br s	-	15	M13

^{1}H NMR (600 MHz, DMSO-d_6): δ = 2.57 (t, *J* = 6.6 Hz, 2 H, 3), 3.00 (t, *J* = 6.1 Hz, 2 H, 2), 3.04 (dd, *J* = 13.9, 9.5 Hz, 1 H, 17<">), 3.14 (s, 3 H, 30), 3.28 (dd, *J* = 15.0, 4.4 Hz, 1 H, 17<'>), 3.87 (s, 2 H, 10), 4.66 (td, *J* = 8.5, 5.0 Hz, 1 H, 16), 7.18 (s, 1 H, 8), 7.55 (t, *J* = 7.7 Hz, 1 H, 22), 7.66 (dt, *J* = 7.7, 1.0 Hz, 1 H, 23), 7.75 (dt, *J* = 7.7, 1.0 Hz, 1 H, 21), 7.85 (t, *J* = 1.6 Hz, 1 H, 19), 8.73 (br s, 1 H, 15) ppm

Solvent	**DMSO-d6**
Pulse Sequence	**zg30**
Temperature (degree C)	**24.700**
Acquisition Time (sec)	**1.3631**
Number of Transients	**64**
Points Count	**32768**
Sweep Width (Hz)	**12018.86**
Spectrum Offset (Hz)	**5401.1729**
Original Points Count	**16384**
Nucleus	**1H**
Frequency (MHz)	**600.1300**

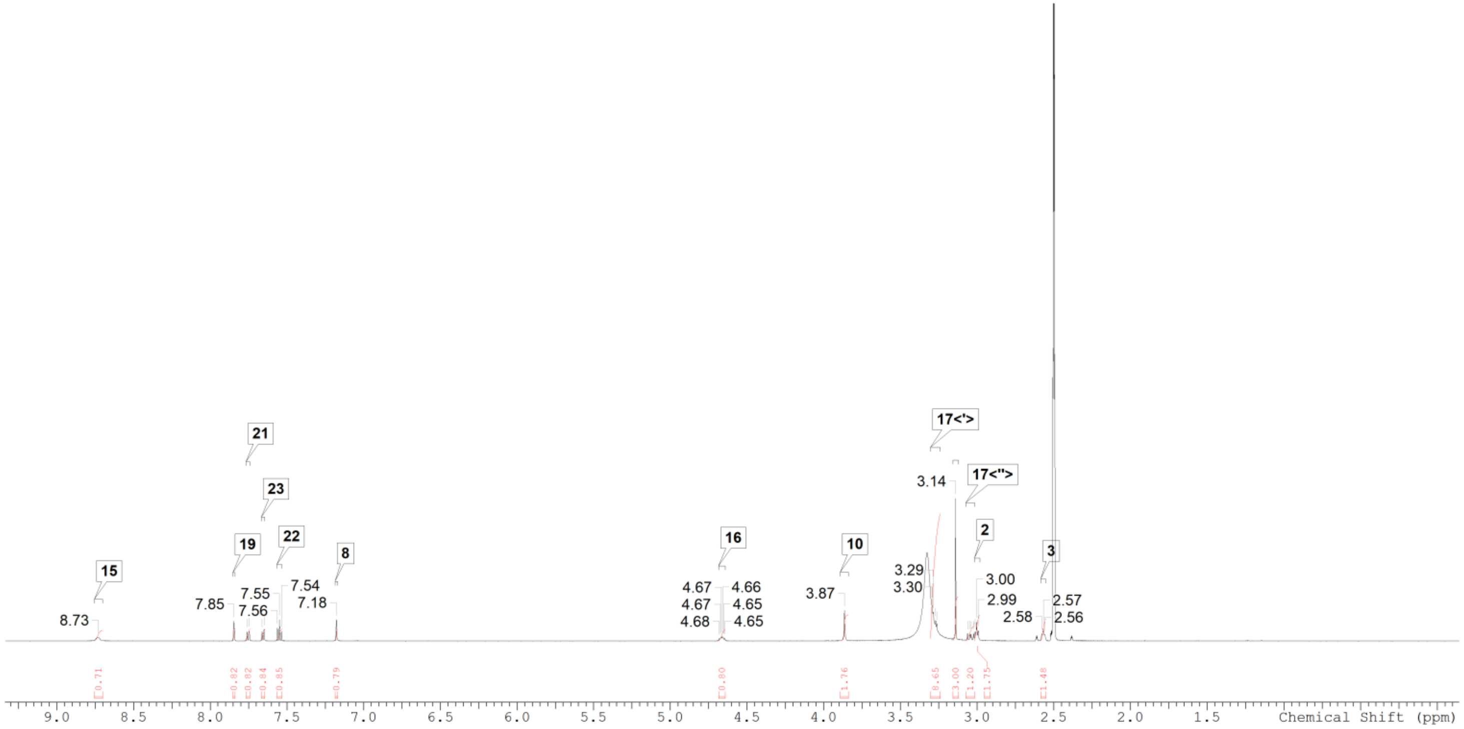

Fig. S8. Fully assigned ^{1}H spectrum of DP1 in DMSO-d_6 at 25 °C

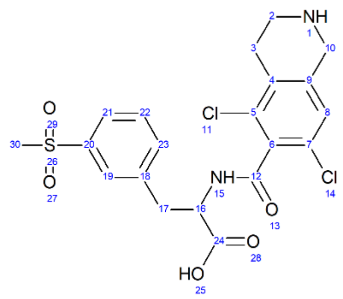

Shift1 (ppm)	C's	Type	Atom1	Multiplet1
26.04	1.000	s	3	M02
36.49	1.000	s	17	M03
42.25	1.000	s	2	M06
43.59	1.000	s	30	M07
46.74	1.000	s	10	M08
53.51	1.000	s	16	M09
124.88	1.000	s	21	M13
125.31	1.000	s	8	M14
127.63	1.000	s	7	M15
127.77	1.000	s	19	M16
129.09	1.000	s	22	M17
131.20	1.000	s	5	M18
131.87	1.000	s	4	M19
134.14	1.000	s	6	M20
134.49	1.000	s	23	M21
139.00	1.000	s	9	M24
139.37	1.000	s	18	M22
140.48	1.000	s	20	M23
163.49	1.000	s	12	M26
171.88	1.000	s	24	M27

^{13}C NMR (151 MHz, DMSO-d_6): δ = 26.0 (3), 36.5 (17), 42.2 (2), 43.6 (30), 46.7 (10), 53.5 (16), 124.9 (21), 125.3 (8), 127.6 (7), 127.8 (19), 129.1 (22), 131.2 (5), 131.9 (4), 134.1 (6), 134.5 (23), 139.0 (9), 139.4 (18), 140.5 (20), 163.5 (12), 171.9 (24) ppm

Solvent	**DMSO-d6**
Pulse Sequence	**deptqgpsp.2**
Temperature (degree C)	**25.100**
Acquisition Time (sec)	**0.5999**
Number of Transients	**8192**
Points Count	**32768**
Sweep Width (Hz)	**39368.88**
Spectrum Offset (Hz)	**15014.3535**
Original Points Count	**23619**
Nucleus	**13C**
Frequency (MHz)	**150.9029**

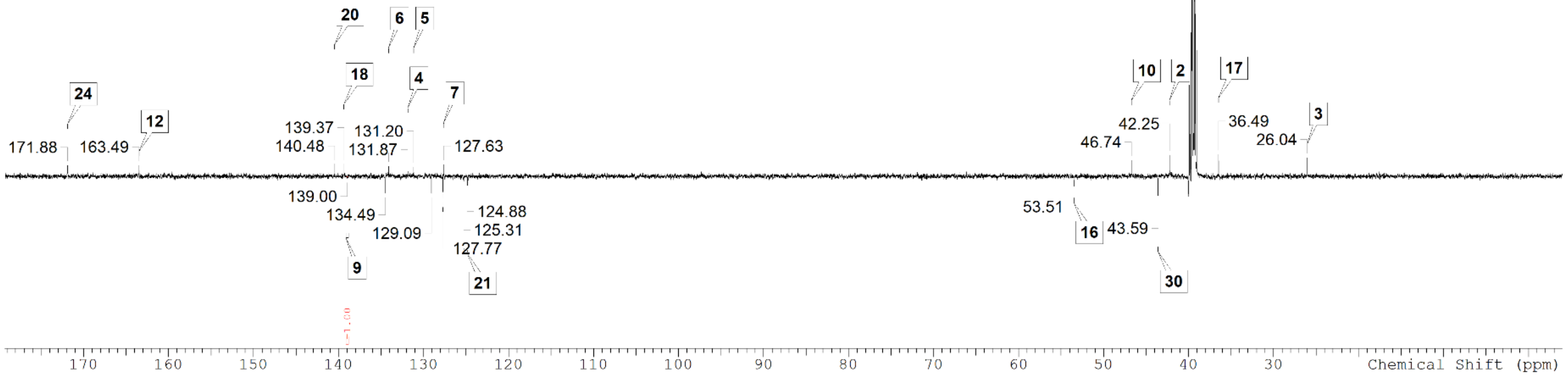

Fig. S9. Fully assigned ^{13}C spectrum of DP1 in DMSO-d_6 at 25 °C

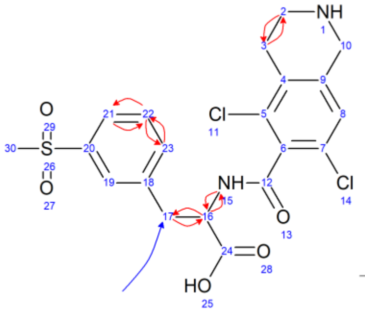

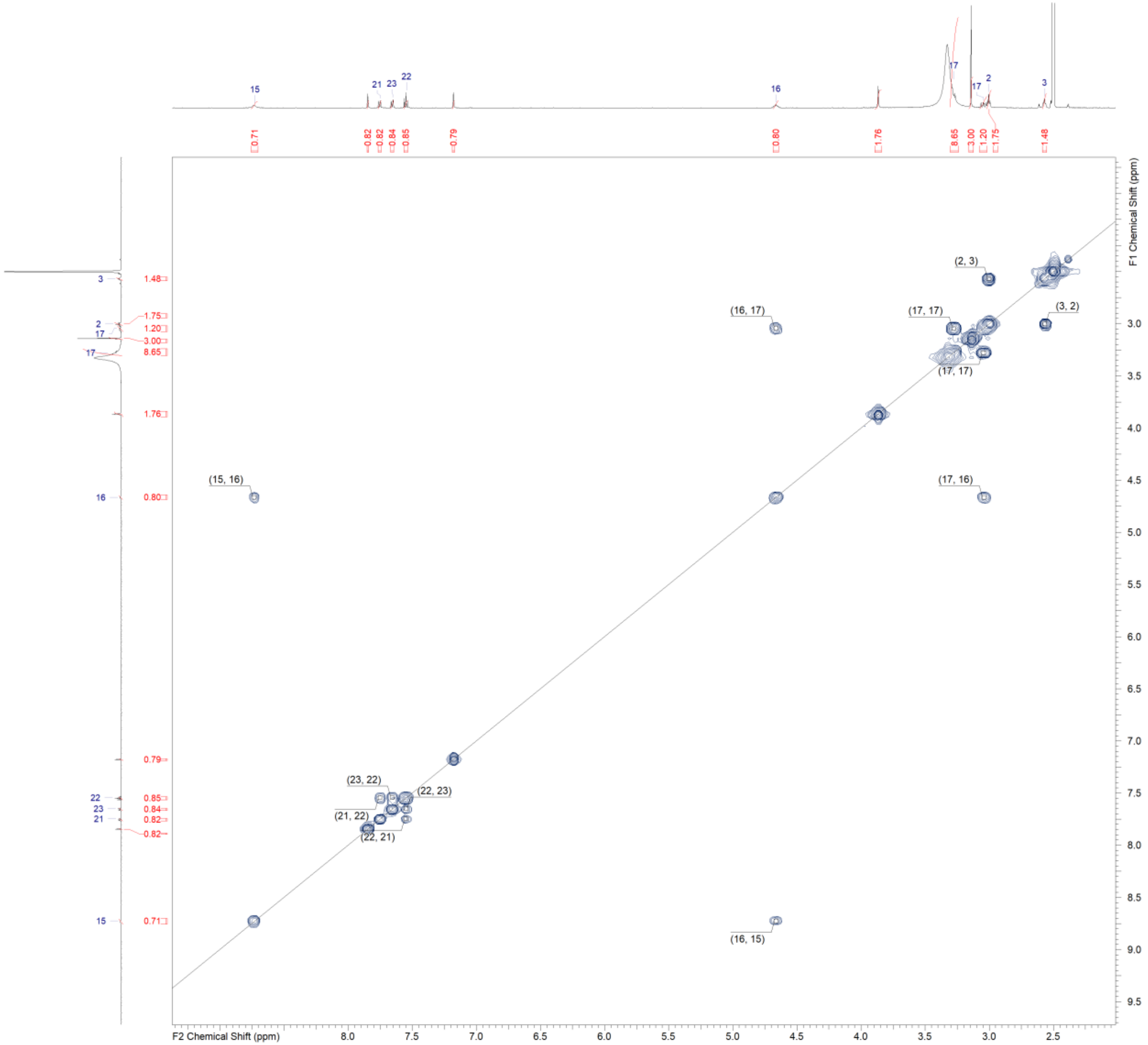

Acquisition Time (sec)	(0.1065, 0.0267)
Date	25 Oct 2022 14:07:14
Frequency (MHz)	(600.1300, 600.1300)
Nucleus	(1H, 1H)
Original Points Count	(1024, 256)
Points Count	(2048, 2048)
Pulse Sequence	cosygpqf
Solvent	DMSO-d6
Sweep Width (Hz)	(9610.69, 9597.45)
Number of Transients	4
Spectrum Type	COSY
Temperature (degree C)	25.000

Fig. S10. Fully assigned ^{1}H-^{1}H COSY spectrum of DP1 in DMSO-d_6 at 25 °C

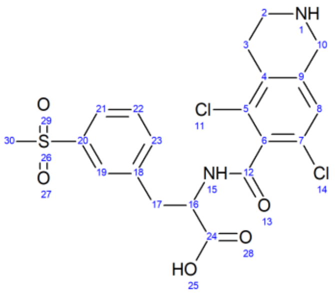

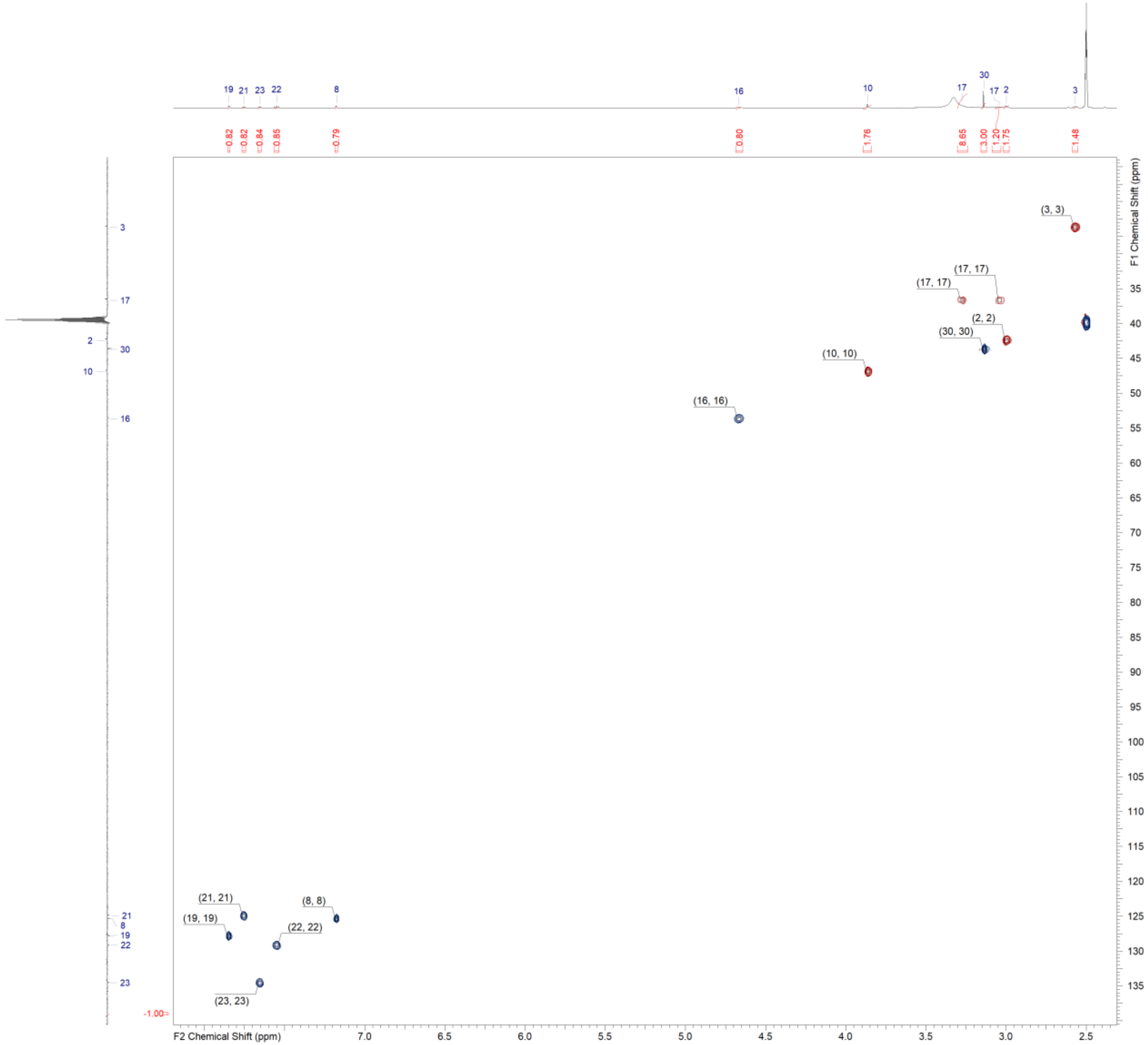

Acquisition Time (sec)	(0.1139, 0.0094)
Date	25 Oct 2022 14:07:16
Frequency (MHz)	(600.1300, 150.9029)
Nucleus	(1H, 13C)
Original Points Count	(1024, 256)
Points Count	(1024, 1024)
Pulse Sequence	hsqcedetgpsisp.2
Solvent	DMSO-d6
Sweep Width (Hz)	(8984.02, 27138.29)
Number of Transients	64
Spectrum Type	HSQC-DEPT
Temperature (degree C)	25.100

Fig. S11. Fully assigned ^{1}H-^{13}C HSQCe spectrum of DP1 in DMSO-d_6 at 25 °C

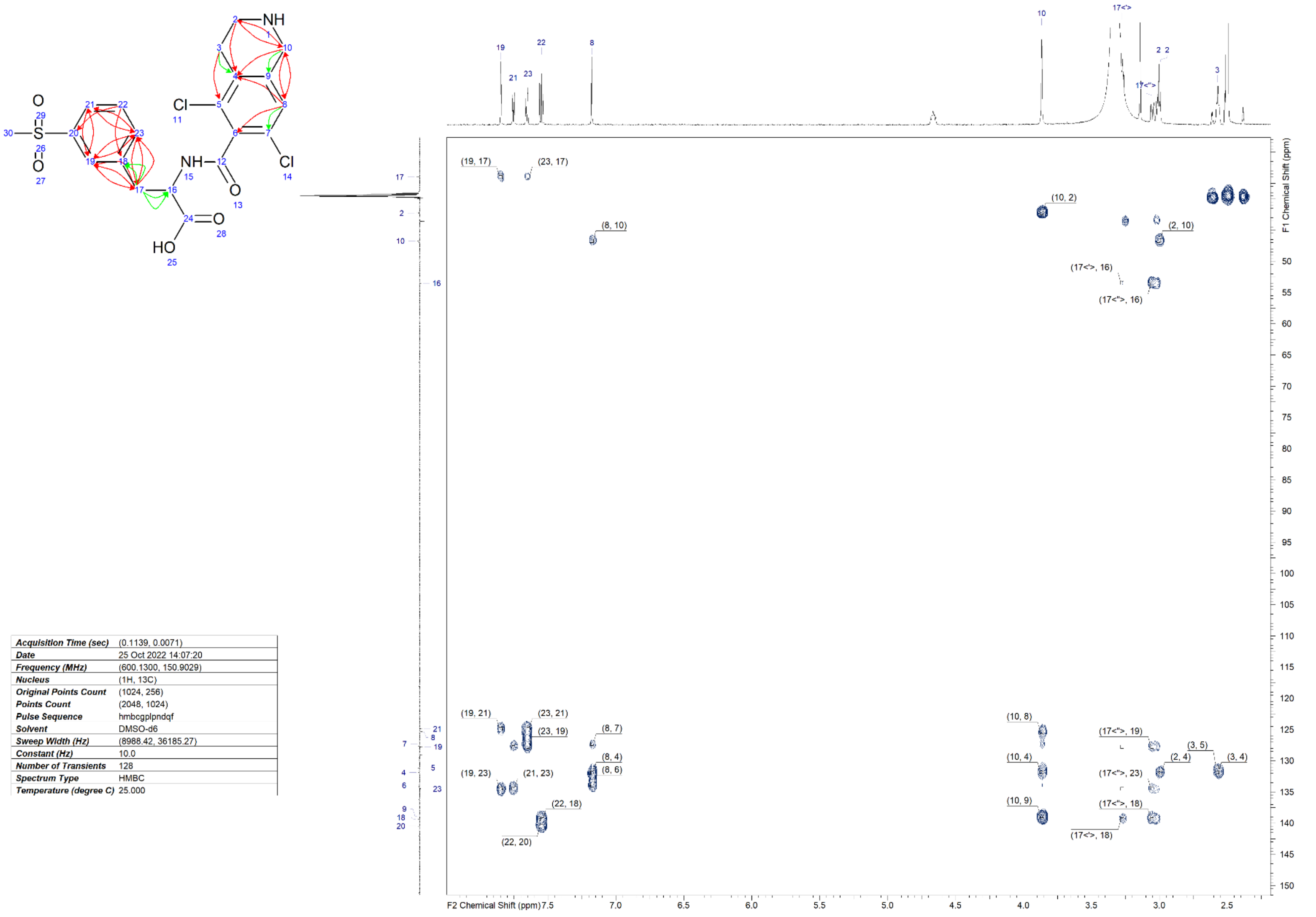

Acquisition Time (sec)	(0.1139, 0.0071)
Date	25 Oct 2022 14:07:20
Frequency (MHz)	(600.1300, 150.9029)
Nucleus	(1H, 13C)
Original Points Count	(1024, 256)
Points Count	(2048, 1024)
Pulse Sequence	hmbcgplpndqf
Solvent	DMSO-d6
Sweep Width (Hz)	(8988.42, 36185.27)
Constant (Hz)	10.0
Number of Transients	128
Spectrum Type	HMBC
Temperature (degree C)	25.000

Fig. S12. Fully assigned ^{1}H-^{13}C HMBC spectrum of DP1 in DMSO-d_6 at 25 °C

S3.1.3. DP2

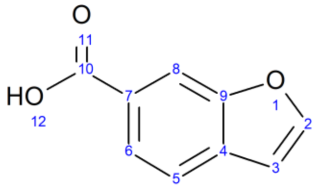

Shift1 (ppm)	H's	Type	J (Hz)	Atom1	Multiplet1
7.07	1.000	dd	2.2, 1.1	3	M01
7.76	1.000	d	8.1	5	M02
7.87	1.000	dd	8.1, 1.5	6	M03
8.12	1.000	s	-	8	M05
8.19	1.000	d	2.2	2	M04
12.96	1.000	br s	-	12	M06

^{1}H NMR (600 MHz, DMSO-d_6): δ = 7.07 (dd, J = 2.2, 1.1 Hz, 1 H, 3), 7.76 (d, J = 8.1 Hz, 1 H, 5), 7.87 (dd, J = 8.1, 1.5 Hz, 1 H, 6), 8.12 (s, 1 H, 8), 8.19 (d, J = 2.2 Hz, 1 H, 2), 12.96 (br s, 1 H, 12) ppm

Solvent	**DMSO-d 6**
Pulse Sequence	**zg30**
Temperature (degree C)	**25.000**
Acquisition Time (sec)	**1.3631**
Number of Transients	**16**
Points Count	**32768**
Sweep Width (Hz)	**12018.86**
Spectrum Offset (Hz)	**5401.172 9**
Original Points Count	**16384**
Nucleus	**1H**
Frequency (MHz)	**600.1300**

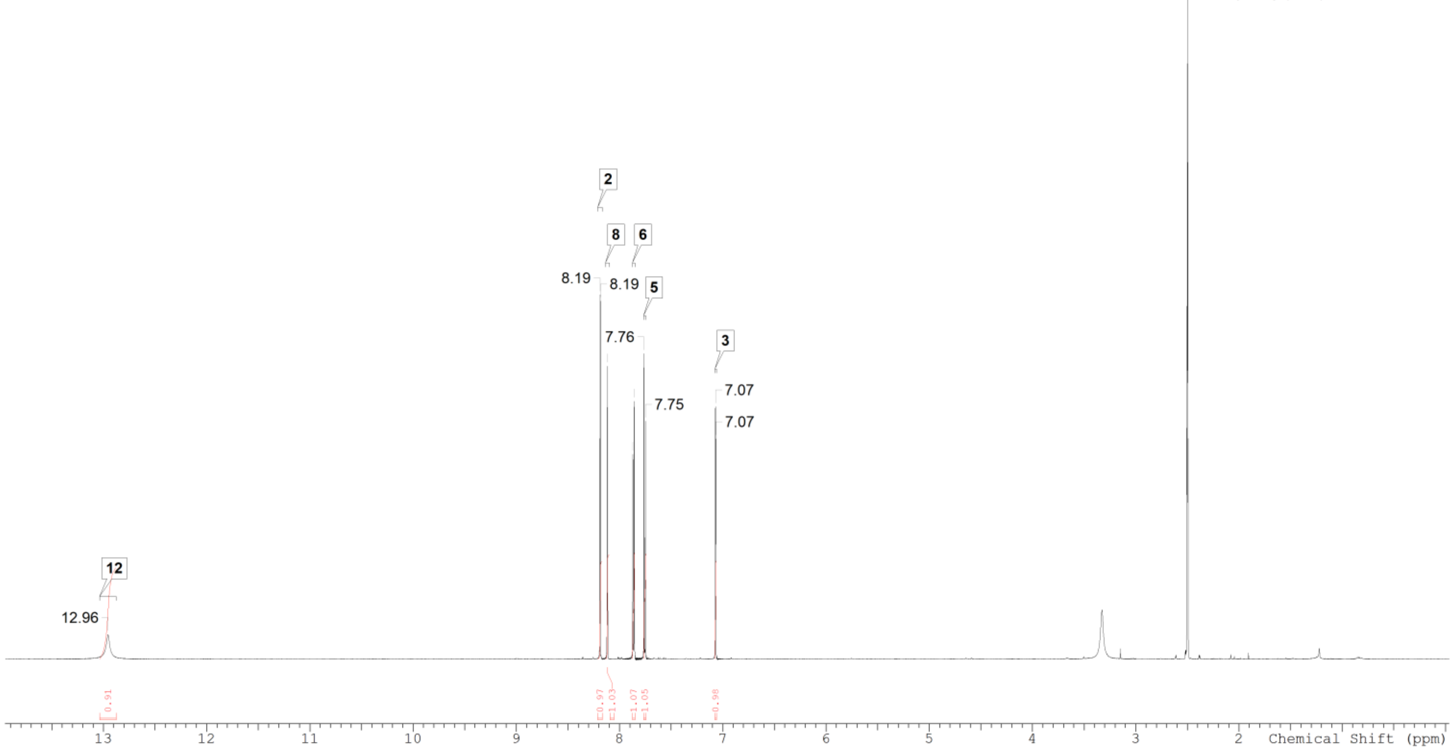

Fig. S13.. Fully assigned ^{1}H spectrum of DP2 in DMSO-d_6 at 25 °C

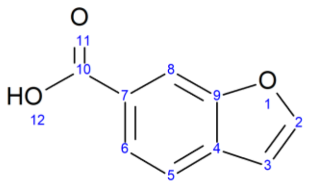

Shift1 (ppm)	C's	Type	Atom1	Multiplet1
106.99	1.000	s	3	M01
112.32	1.000	s	8	M02
121.13	1.000	s	5	M03
123.93	1.000	s	6	M04
126.95	1.000	s	7	M05
131.37	1.000	s	4	M06
149.01	1.000	s	2	M07
153.77	1.000	s	9	M08
167.34	1.000	s	10	M09

^{13}C NMR (151 MHz, DMSO-d_6): δ = 107.0 (3), 112.3 (8), 121.1 (5), 123.9 (6), 126.9 (7), 131.4 (4), 149.0 (2), 153.8 (9), 167.3 (10) ppm

Solvent	**DMSO-d6**
Pulse Sequence	**deptqgpsp.2**
Temperature (degree C)	**25.200**
Acquisition Time (sec)	**0.5999**
Number of Transients	**519**
Points Count	**32768**
Sweep Width (Hz)	**39368.88**
Spectrum Offset (Hz)	**15014.3535**
Original Points Count	**23619**
Nucleus	**13C**
Frequency (MHz)	**150.9029**

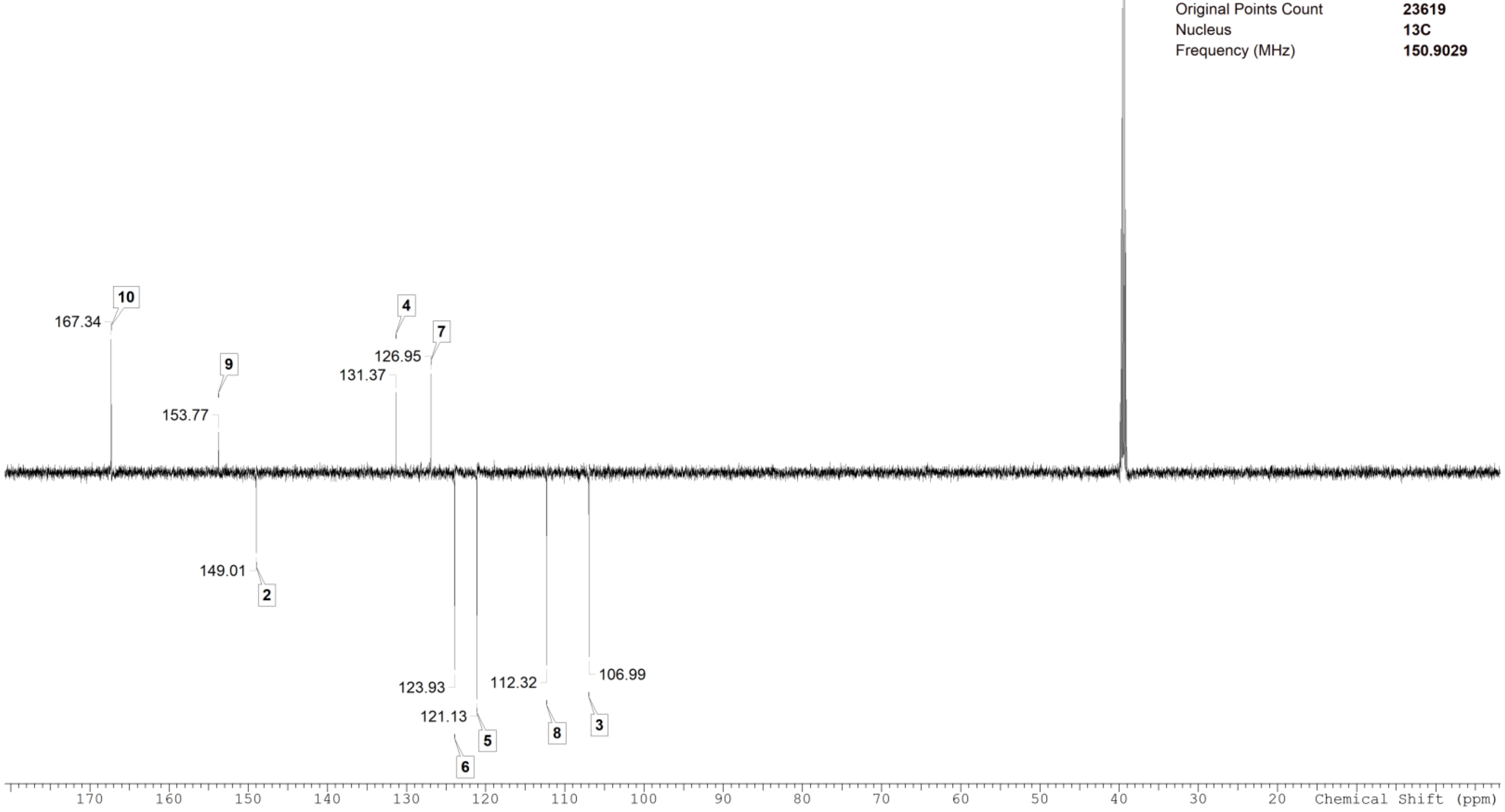

Fig. S14. Fully assigned ^{13}C spectrum of DP2 in DMSO-d_6 at 25 °C

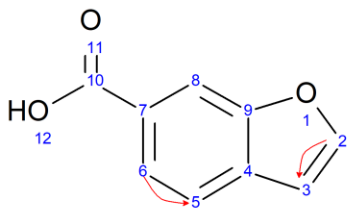

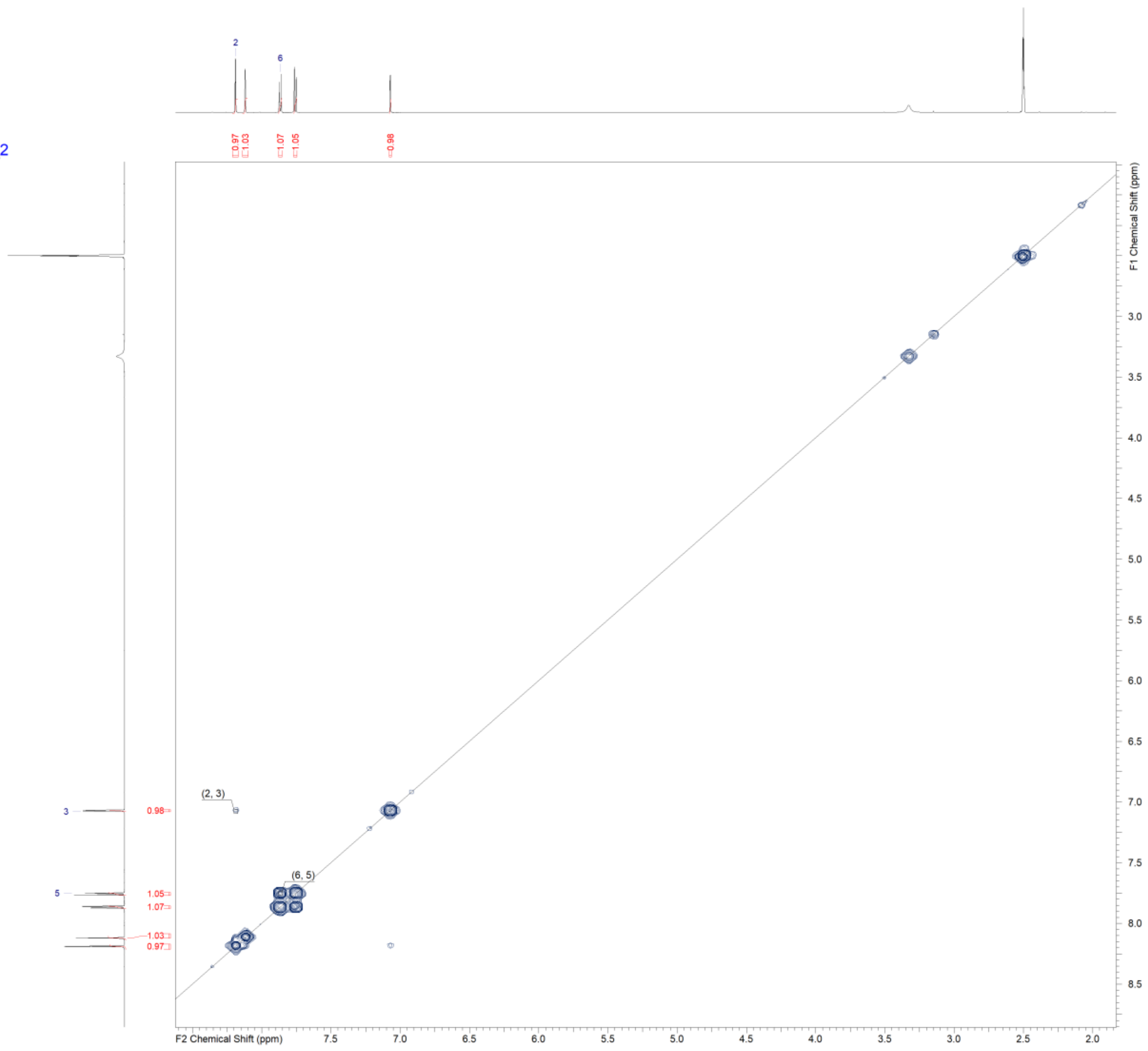

Acquisition Time (sec)	(0.1065, 0.0267)
Date	09 Sep 2022 18:50:46
Frequency (MHz)	(600.1300, 600.1300)
Nucleus	(1H, 1H)
Original Points Count	(1024, 256)
Points Count	(2048, 2048)
Pulse Sequence	cosygpqf
Solvent	DMSO-d6
Sweep Width (Hz)	(9610.69, 9597.45)
Number of Transients	4
Spectrum Type	COSY
Temperature (degree C)	25.000

Fig. S15. Fully assigned ^{1}H-^{1}H COSY spectrum of DP2 in DMSO-d$_6$ at 25 °C

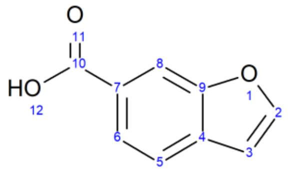

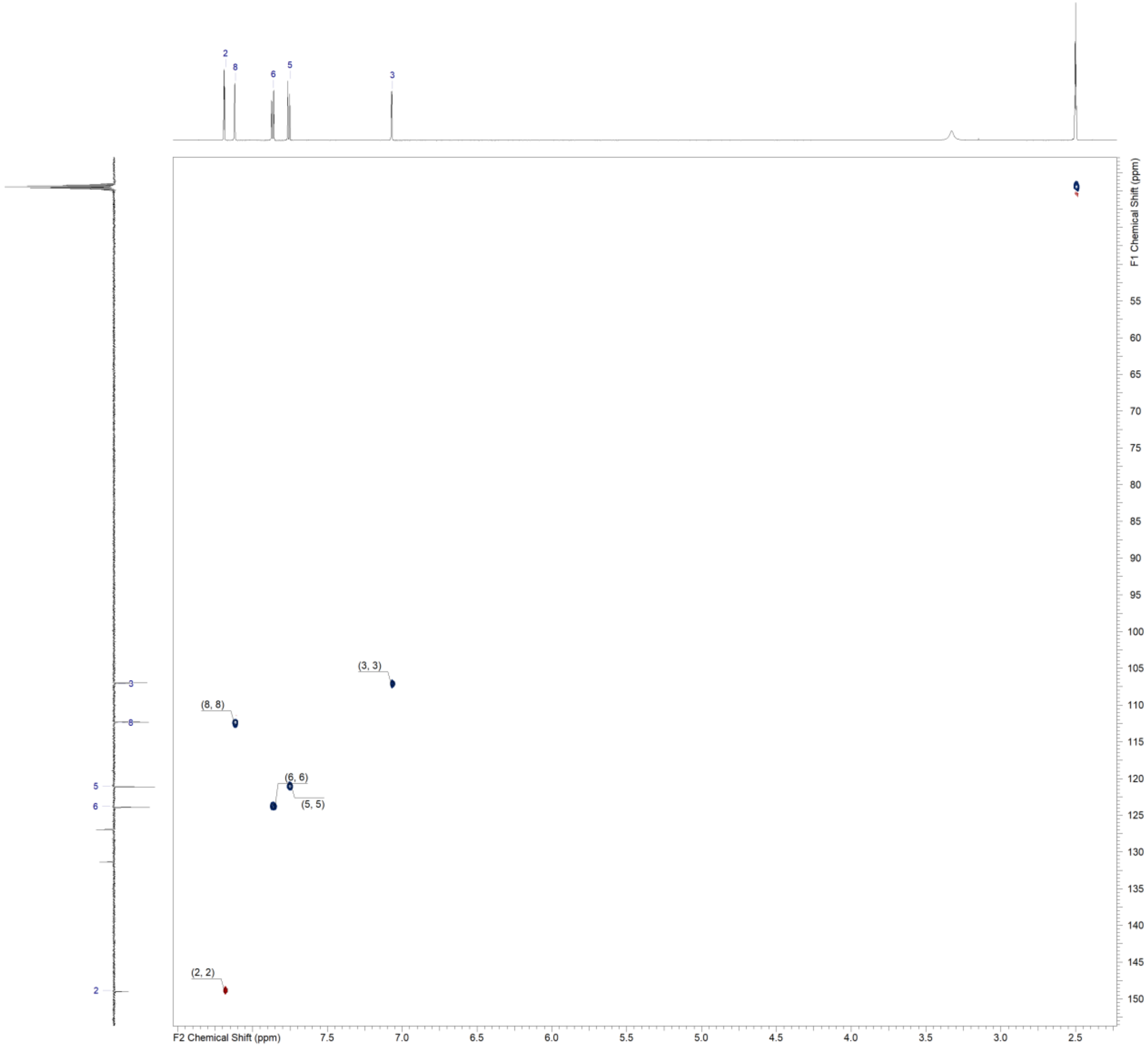

Acquisition Time (sec)	(0.1139, 0.0094)
Date	15 Sep 2022 10:56:36
Frequency (MHz)	(600.1300, 150.9029)
Nucleus	(1H, 13C)
Original Points Count	(1024, 256)
Points Count	(1024, 1024)
Pulse Sequence	hsqcedetgpsisp.2
Solvent	DMSO-d6
Sweep Width (Hz)	(8984.02, 27138.29)
Number of Transients	16
Spectrum Type	HSQC-DEPT
Temperature (degree C)	25.100

Fig. S16. Fully assigned ^{1}H-^{13}C HSQCe spectrum of DP2 in DMSO-d_6 at 25 °C

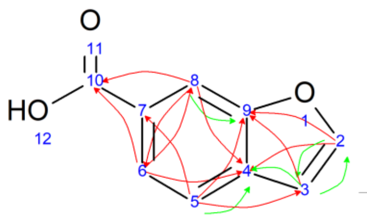

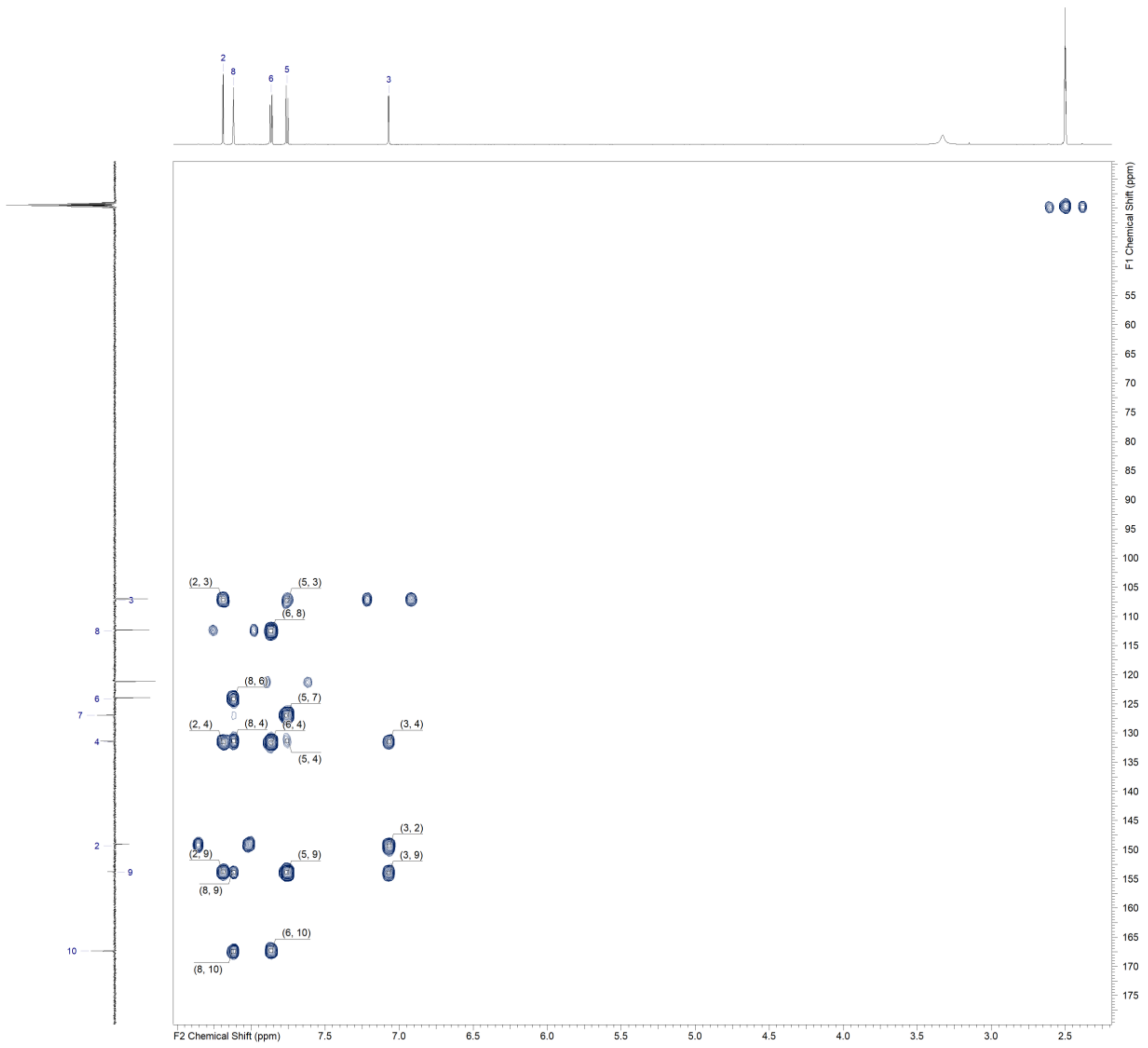

Acquisition Time (sec)	(0.1139, 0.0049)
Date	09 Sep 2022 19:50:52
Frequency (MHz)	(600.1300, 150.9029)
Nucleus	(1H, 13C)
Original Points Count	(1024, 178)
Points Count	(2048, 1024)
Pulse Sequence	hmbcgplpndqf
Solvent	DMSO-d6
Sweep Width (Hz)	(8988.42, 36185.27)
Constant (Hz)	10.0
Number of Transients	32
Spectrum Type	HMBC
Temperature (degree C)	24.900

Fig. S17. Fully assigned ^{1}H-^{13}C HMBC spectrum of DP2 in DMSO-d_6 at 25 °C

S3.1.4. DP3, DP4, DP5 and DP6 in the oxidative degradation mixture

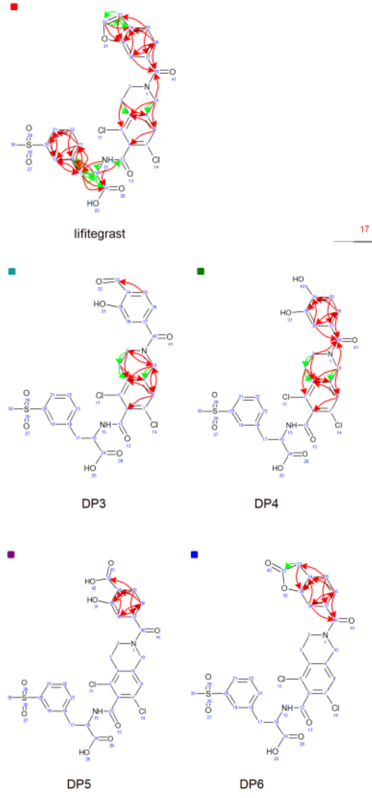

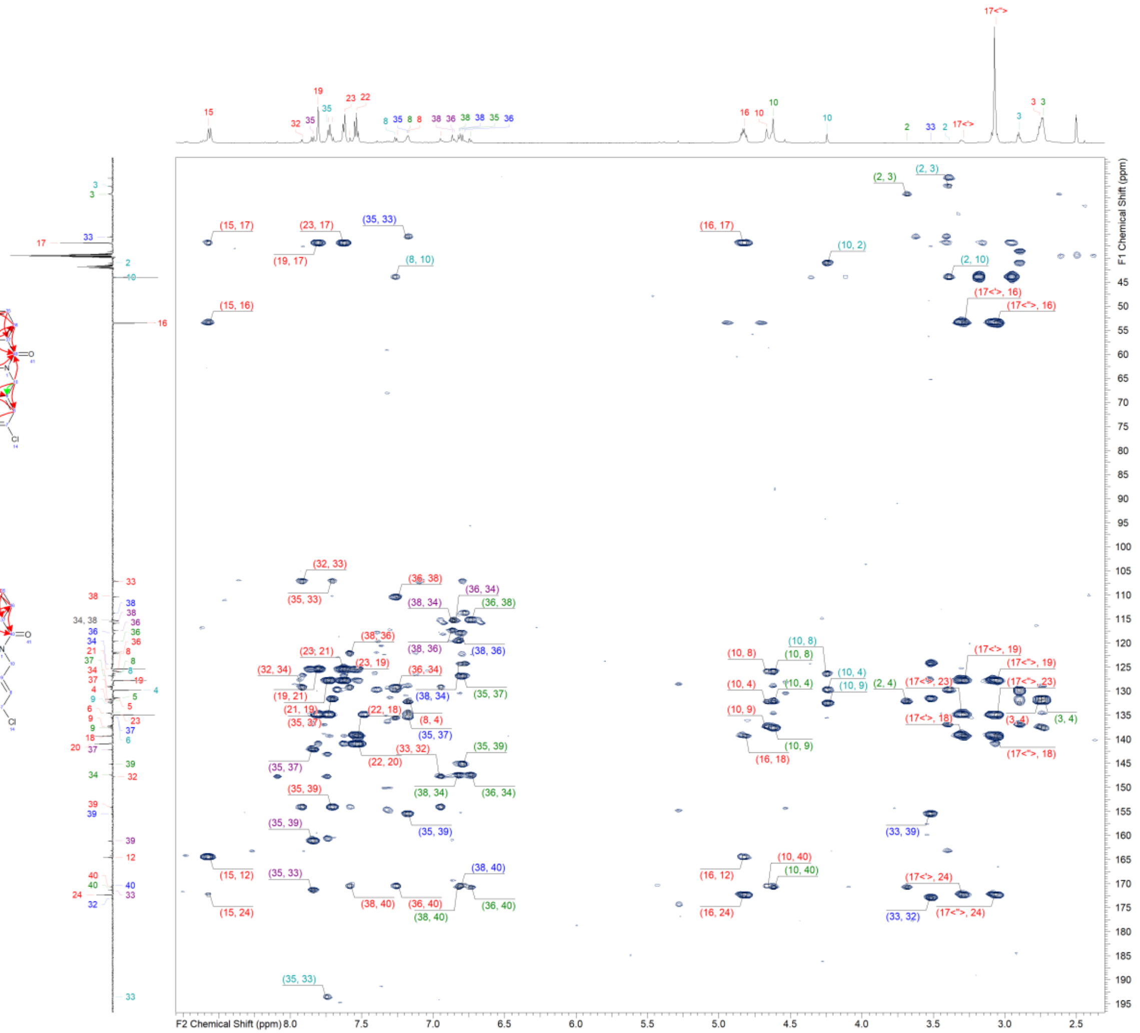

Acquisition Time (sec)	(0.1139, 0.0283)
Date	05 Dec 2022 11:42:44
Frequency (MHz)	(600.1300, 150.9028)
Nucleus	(1H, 13C)
Original Points Count	(1024, 1024)
Points Count	(2048, 1024)
Pulse Sequence	hmbcgplpndqf
Solvent	DMSO-d6
Sweep Width (Hz)	(8988.42, 36185.27)
Constant (Hz)	10.0
Number of Transients	108
Spectrum Type	HMBC
Temperature (degree C)	80.000

Fig. S18. Full ^{1}H-^{13}C HMBC spectrum of the oxidative degradation mixture in DMSO-d_6 at 80 °C

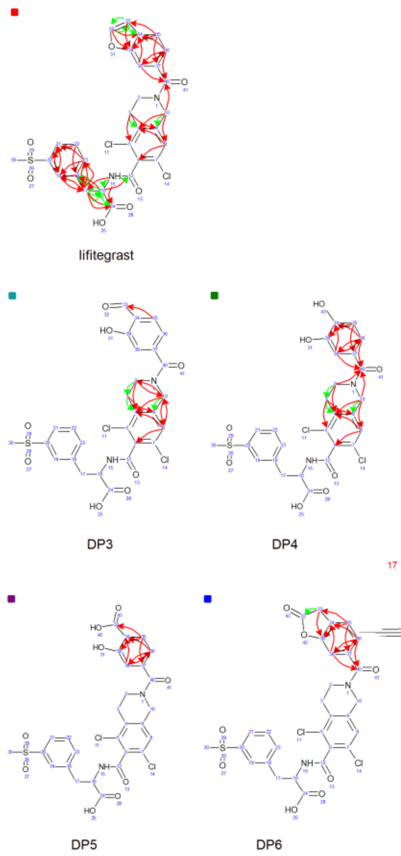

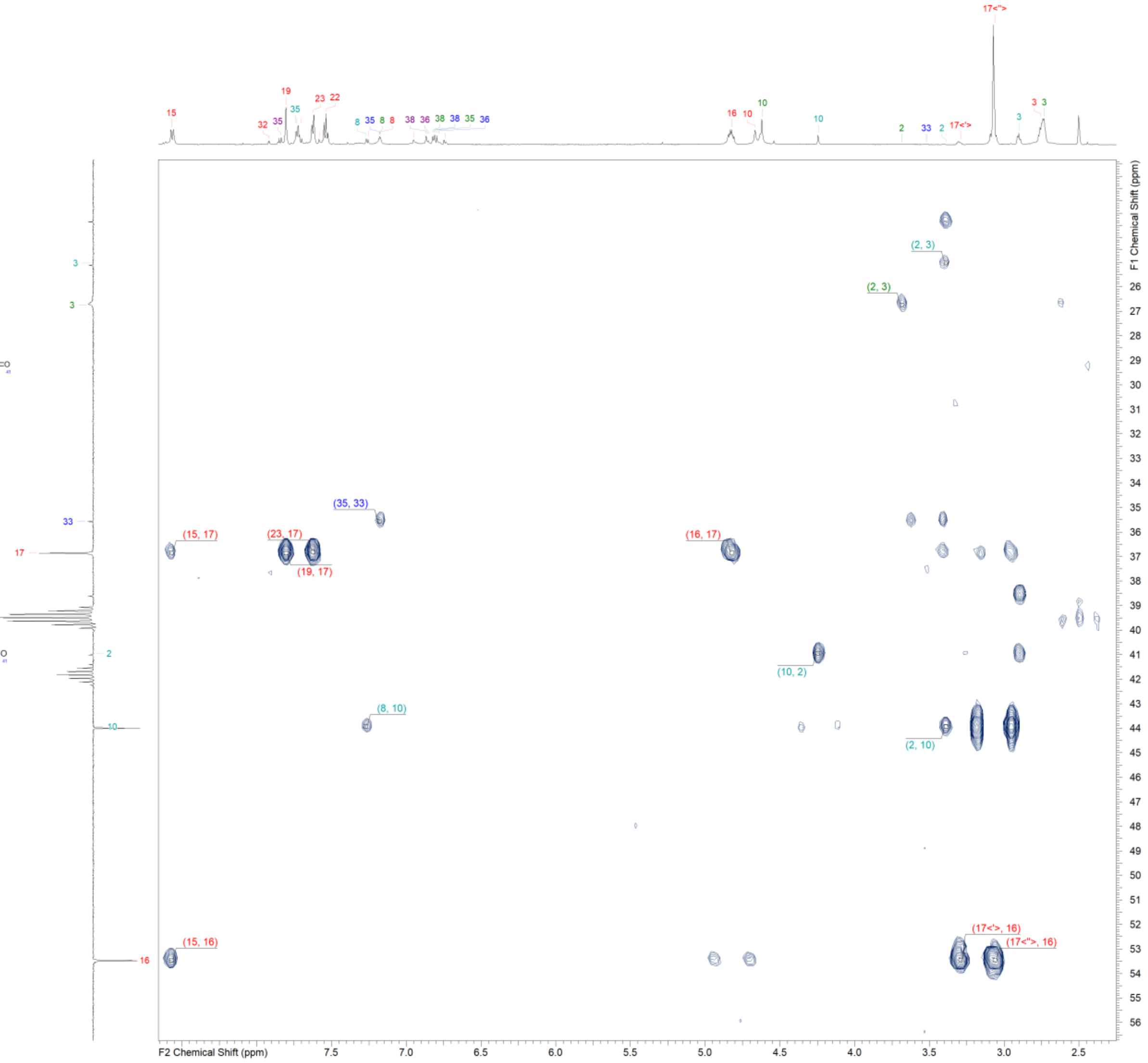

Acquisition Time (sec)	(0.1139, 0.0283)
Date	05 Dec 2022 11:42:44
Frequency (MHz)	(600.1300, 150.9028)
Nucleus	(1H, 13C)
Original Points Count	(1024, 1024)
Points Count	(2048, 1024)
Pulse Sequence	hmbcgplpndqf
Solvent	DMSO-d6
Sweep Width (Hz)	(8988.42, 36185.27)
Constant (Hz)	10.0
Number of Transients	108
Spectrum Type	HMBC
Temperature (degree C)	80.000

Fig. S19. Aliphatic region of ^{1}H-^{13}C HMBC spectrum of the oxidative degradation mixture in DMSO-d_6 at 80 °C

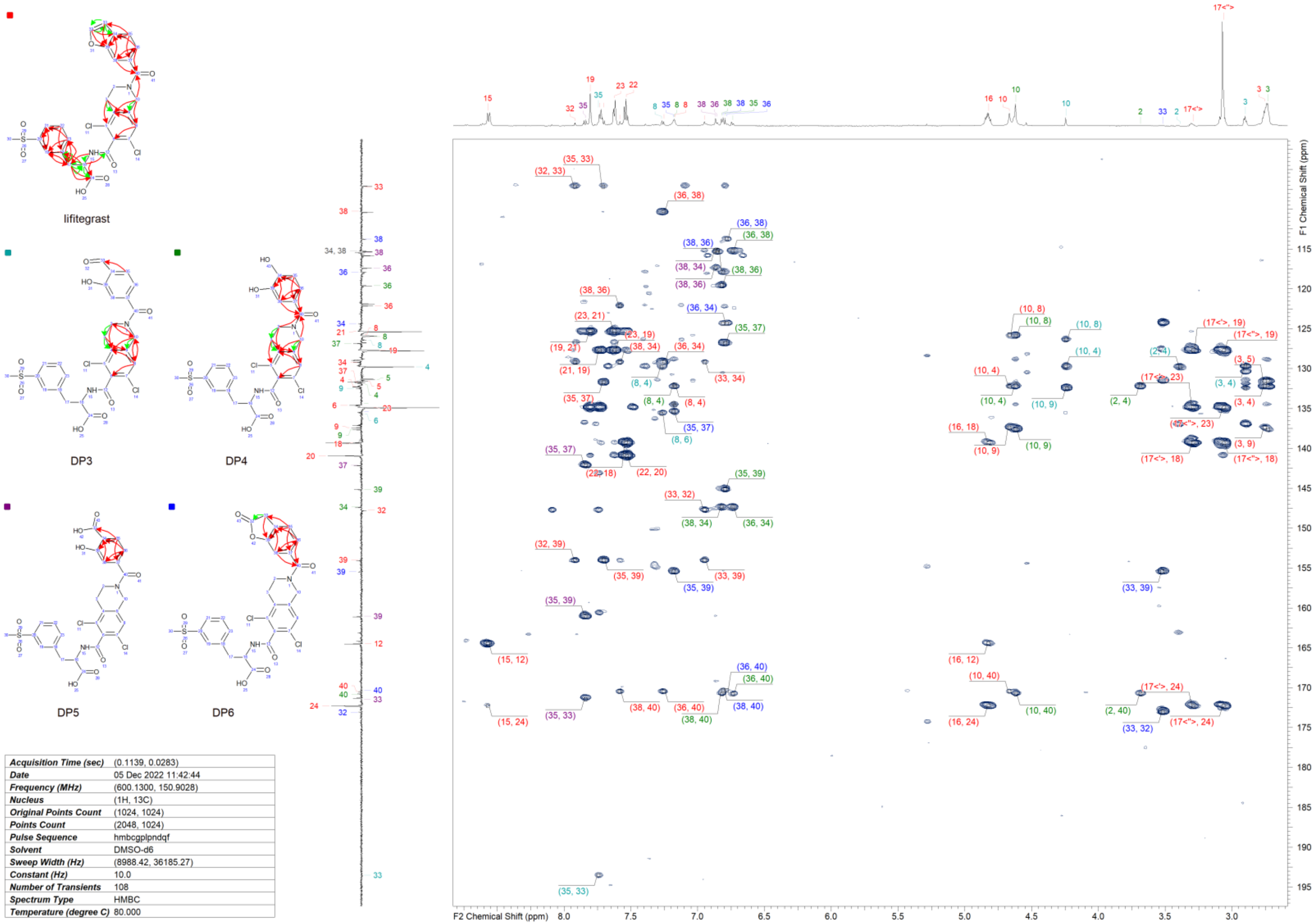

Acquisition Time (sec)	(0.1139, 0.0283)
Date	05 Dec 2022 11:42:44
Frequency (MHz)	(600.1300, 150.9028)
Nucleus	(1H, 13C)
Original Points Count	(1024, 1024)
Points Count	(2048, 1024)
Pulse Sequence	hmbcgplpndqf
Solvent	DMSO-d6
Sweep Width (Hz)	(8988.42, 36185.27)
Constant (Hz)	10.0
Number of Transients	108
Spectrum Type	HMBC
Temperature (degree C)	80.000

Fig. S20. Aromatic region of ^{1}H-^{13}C HMBC spectrum of the oxidative degradation mixture in DMSO-d_6 at 80 °C

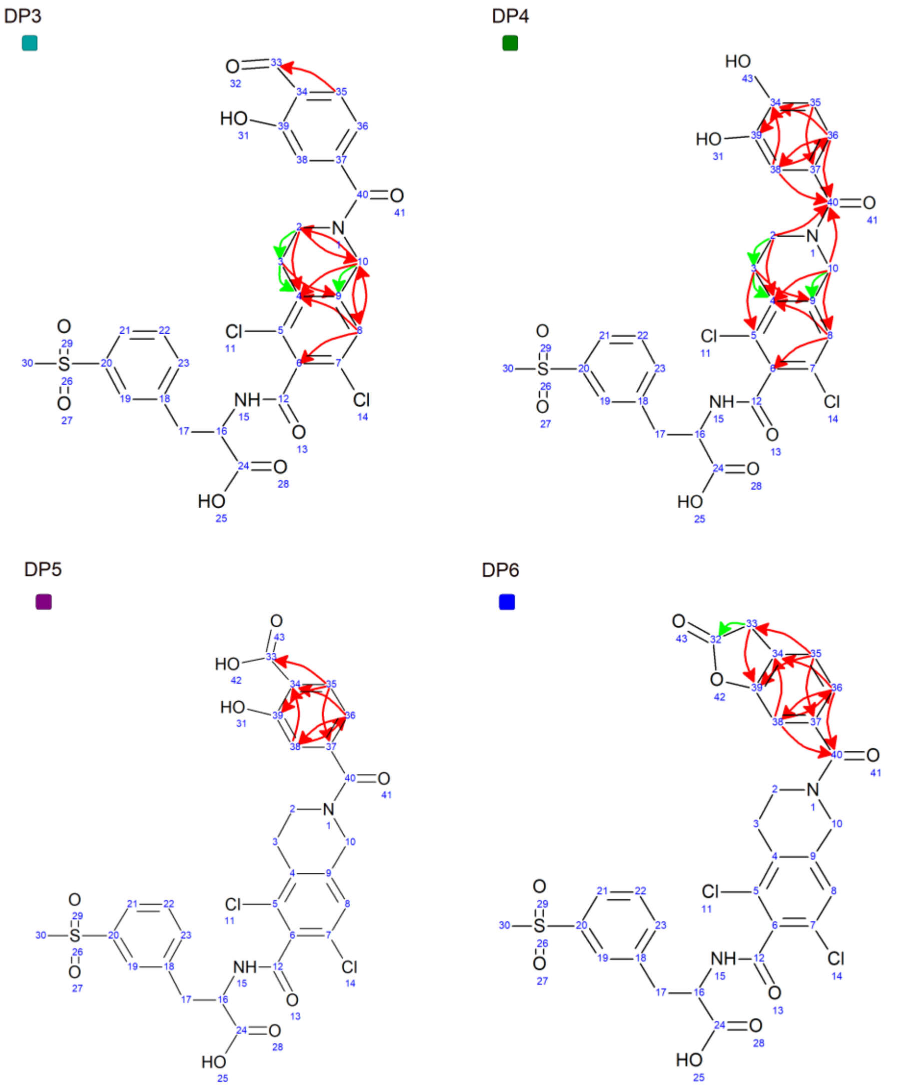

F2 Atom	F1 Atom	F2 (ppm)	F1 (ppm)	Component
10	2	4.25	40.9	DP3
2	3	3.39	25	DP3
2	4	3.39	129.8	DP3
3	4	2.9	129.8	DP3
8	4	7.27	129.8	DP3
10	4	4.25	129.8	DP3
8	6	7.27	135.7	DP3
10	8	4.24	126.4	DP3
3	9	2.9	132.5	DP3
10	9	4.24	132.5	DP3
2	10	3.39	43.9	DP3
8	10	7.27	43.9	DP3
35	33	7.74	193.5	DP3
2	3	3.68	26.7	DP4
2	4	3.68	132	DP4
3	4	2.73	132	DP4
8	4	7.18	132	DP4
10	4	4.62	132	DP4
3	5	2.73	131.4	DP4
8	6	7.18	134.3	DP4
10	8	4.62	126	DP4
3	9	2.73	137.6	DP4
10	9	4.62	137.6	DP4
36	34	6.74	147.3	DP4
38	34	6.82	147.3	DP4
38	36	6.82	119.6	DP4
35	37	6.8	126.8	DP4
36	38	6.74	115.2	DP4
35	39	6.8	145.1	DP4
2	40	3.68	170.9	DP4
10	40	4.62	170.9	DP4
36	40	6.74	170.9	DP4
38	40	6.82	170.9	DP4
35	33	7.84	171.5	DP5
36	34	6.85	115.2	DP5
38	34	6.87	115.2	DP5
38	36	6.87	117.4	DP5
35	37	7.84	142.1	DP5
36	38	6.86	115.4	DP5
35	39	7.84	161	DP5
33	32	3.52	173.1	DP6
35	33	7.18	35.6	DP6
36	34	6.78	124.3	DP6
38	34	6.81	124.3	DP6
38	36	6.81	117.9	DP6
35	37	7.18	135.4	DP6
36	38	6.78	113.7	DP6
33	39	3.52	155.5	DP6
35	39	7.18	155.5	DP6
36	40	6.78	170.3	DP6
38	40	6.81	170.3	DP6

Fig. S21. List and depiction of all ^1H-^{13}C HMBC interactions found in oxidative degradation mixture in DMSO-d_6 at 80 °C

S3.2. MS analysis

Table S6. Experimental parameters for UHPLC-HRMS method used for analysis of degradation products mixture after extended oxidative degradation with 3% H_2O_2 after 30 days at 50 °C/75% RH

**LC parameters**			
UHPLC system	Agilent 1290 Infinity II UHPLC		
Column	Acquity HSS T3 (1.8 μm, 2.1×100 mm)		
Mobile phase	Mobile phase A: 20 mM ammonium formate (pH 4.0) /ACN (99:1)<br>Mobile phase B: 20 mM ammonium formate (pH 4.0) /ACN (20:80)		
Flow rate	0.4 ml/min		
Gradient program	Time (min)	%A	%B
	0.0	100	0
	3.0	100	0
	15.0	50	50
	16.0	50	50
	22.0	0	100
	27.0	0	100
	28.0	100	0
	35.0	100	0
Analysis time	35 min		
Injection volume	3 μl		
Column temperature	40 °C		
**MS parameters**			
MS system	Agilent 6550 Series Accurate-Mass-Quadrupole Time-of-flight		
Drying gas temp.	200 °C		
Drying gas flow	14 l/ min		
Nebulizer	35 psi		
Sheath gas temp.	200 °C		
Sheath gas flow	11 l/min		
Capillary voltage	3000 V		
Nozzle voltage	1000 V		
Fragmentor	380 V		
MS range	*m/z* 100–1000		
MS/MS range	*m/z* 50–700		
Collision energies	10 V, 20 V, 30 V		

S3.2.1. Lifitegrast

The electrospray ionization was performed in positive (ESI+) and negative (ESI–) mode. First the standard solution of Lifitegrast (γ(LIF) = 50 µg/ml) was analyzed.

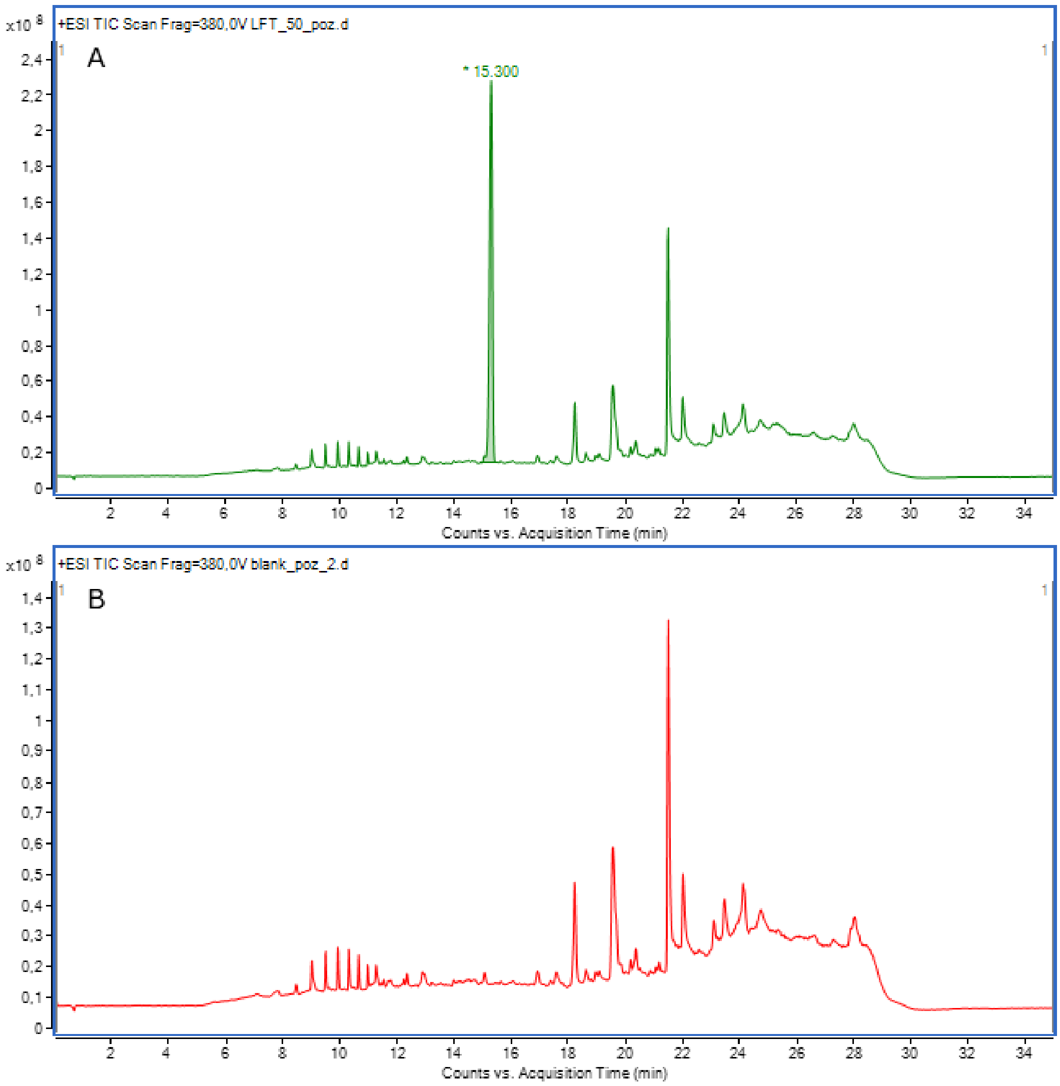

Fig. S22. ESI+ total ion chromatogram of standard solution of Lifitegrast, γ = 50 µg/ml (A) and blank (B)

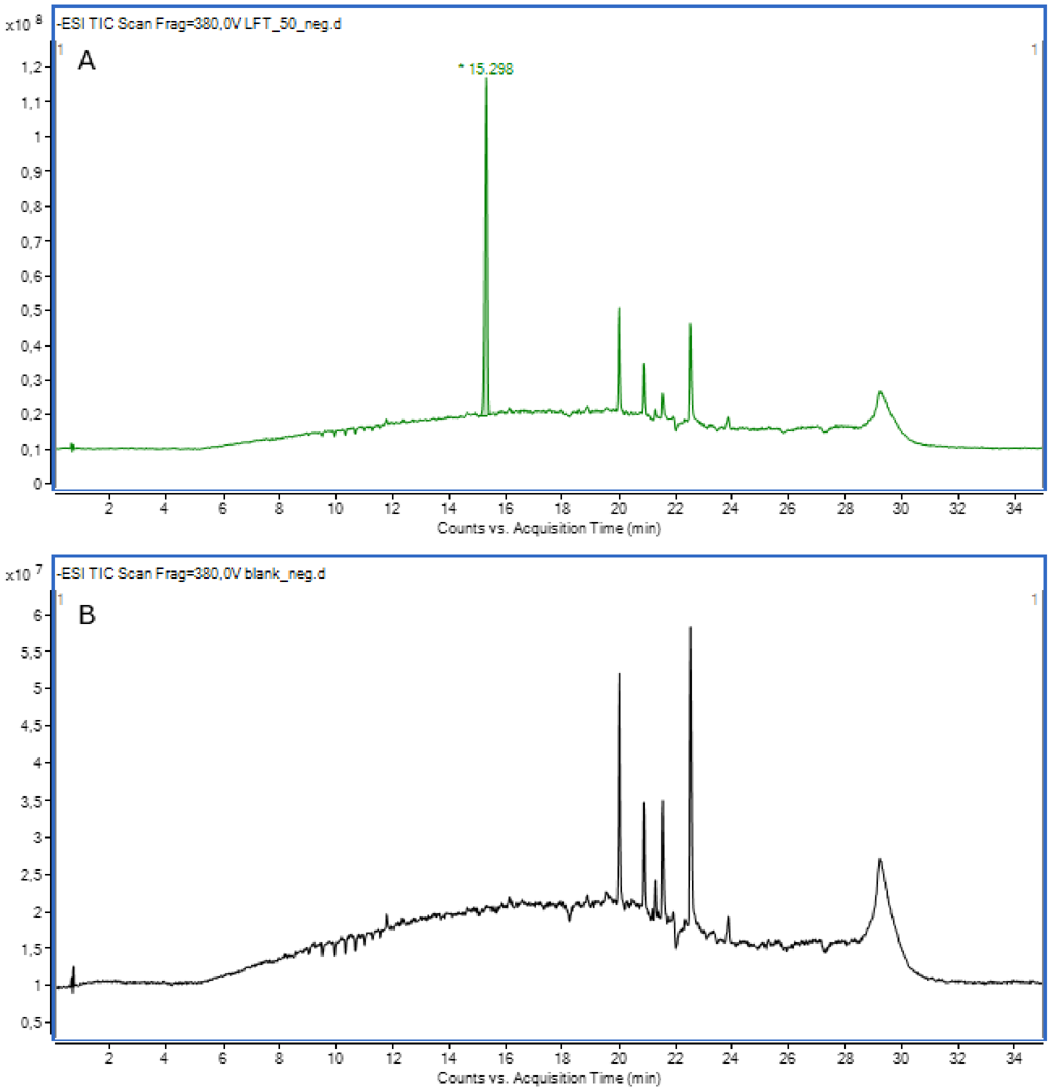

Fig. S23. ESI- total ion chromatogram of standard solution of Lifitegrast, γ = 50 μg/ml (A) and blank (B)

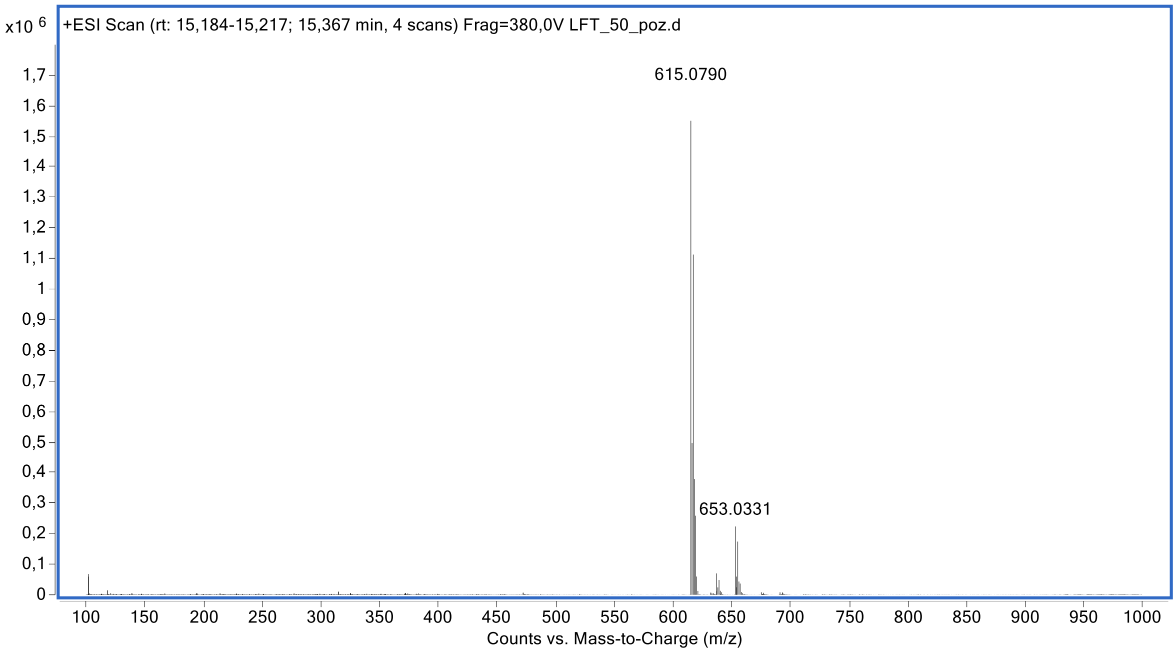

Fig. S24. ESI+ MS spectrum of standard solution of Lifitegrast, γ = 50 μg/ml

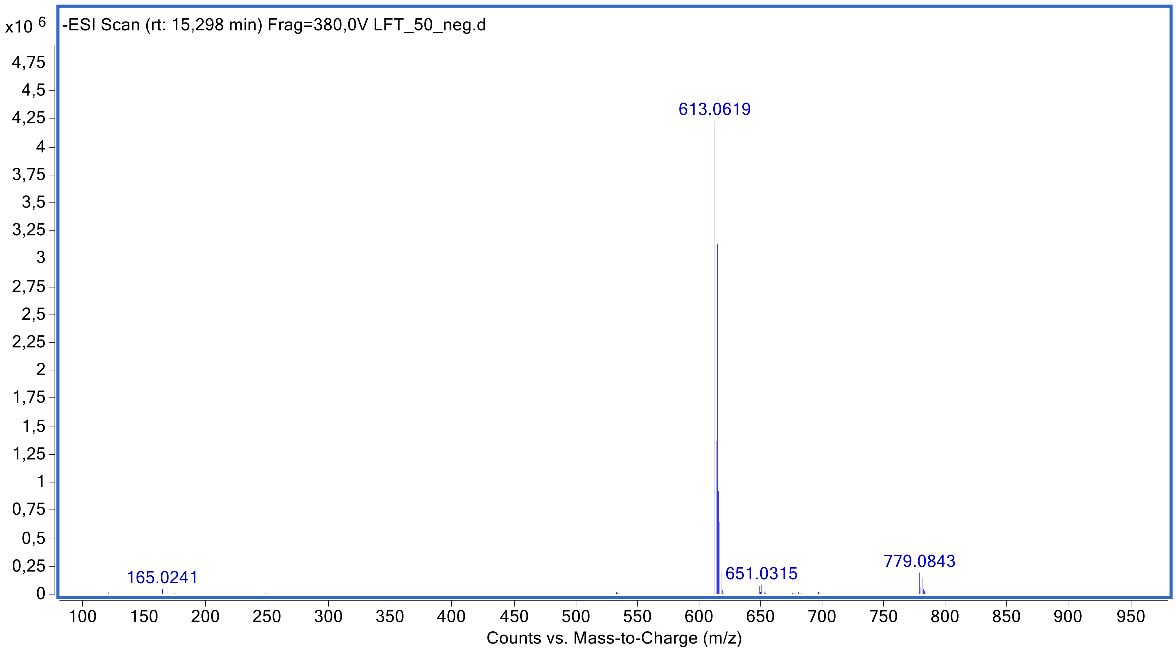

Fig. S25. ESI– MS spectrum of standard solution of Lifitegrast, γ = 50 μg/ml

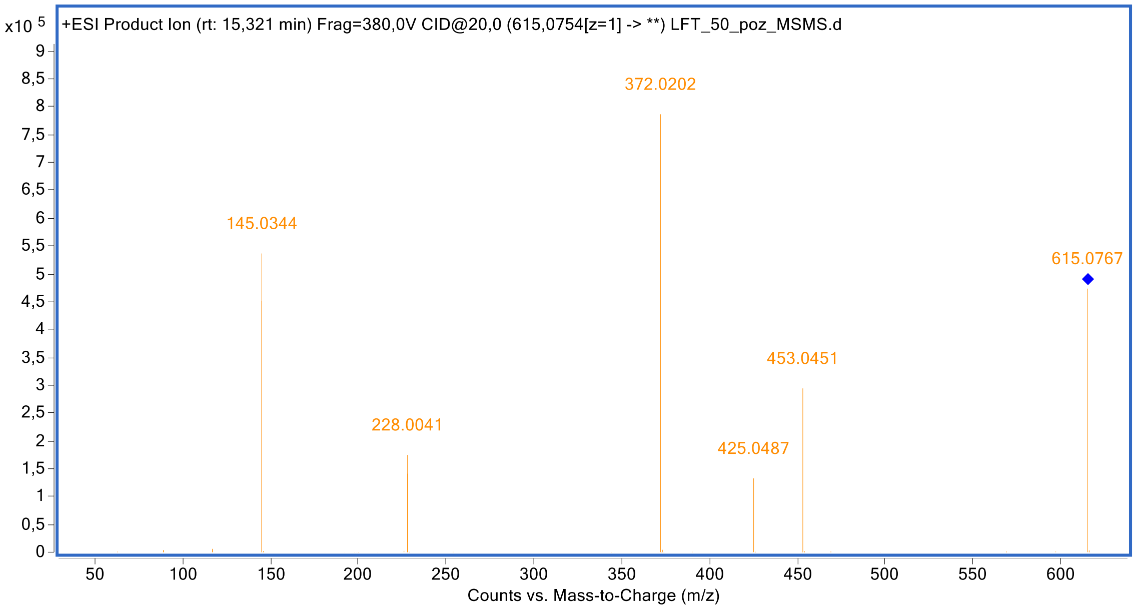

Fig. S26. ESI+ MSMS spectrum of ion at m/z 615.0754, CE 20 V

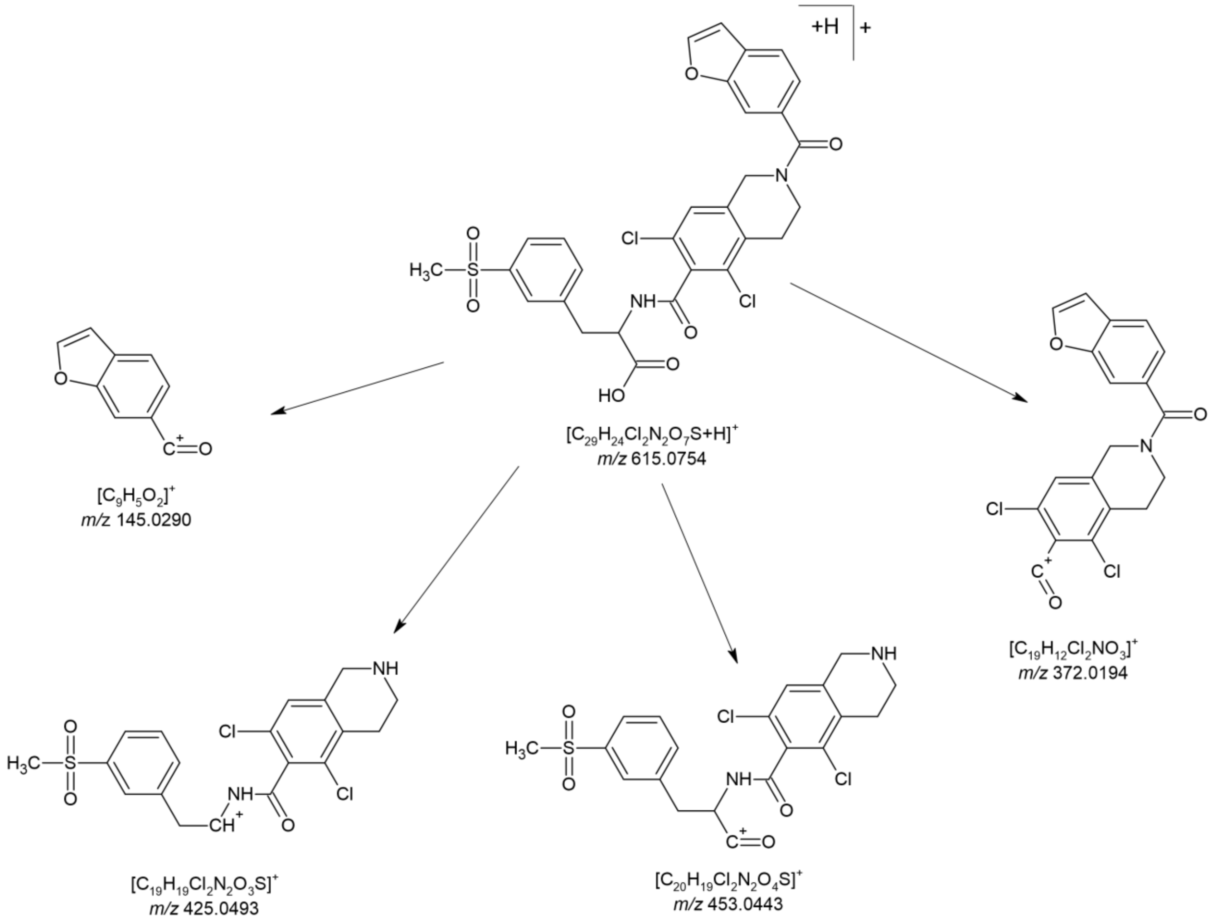

Fig. S27. Possible fragmentation pathway of lifitegrast, ESI+

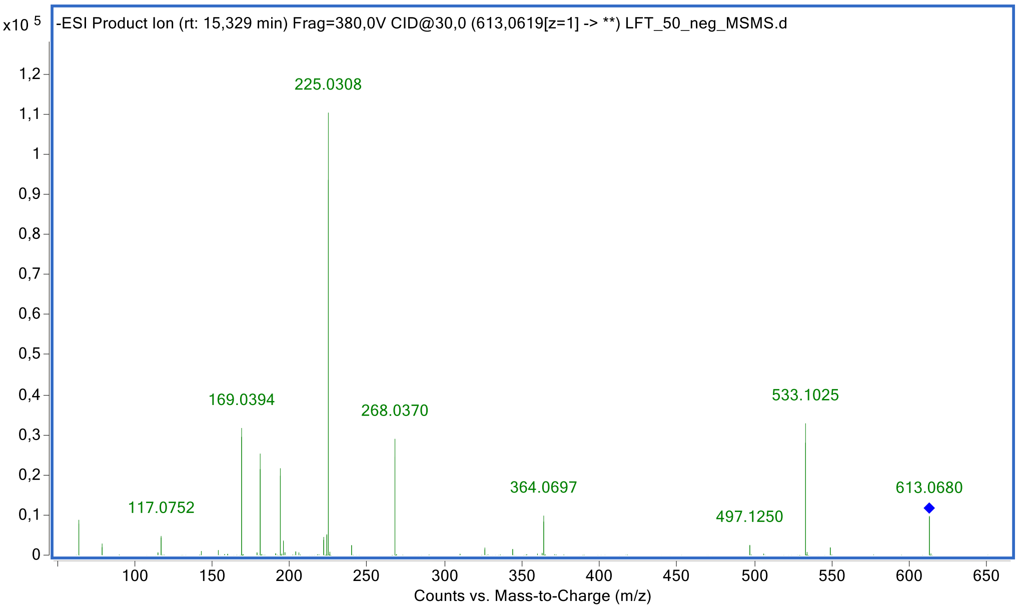

Fig. S28. ESI- MSMS spectrum of ion at m/z 613.0619, CE 30 V

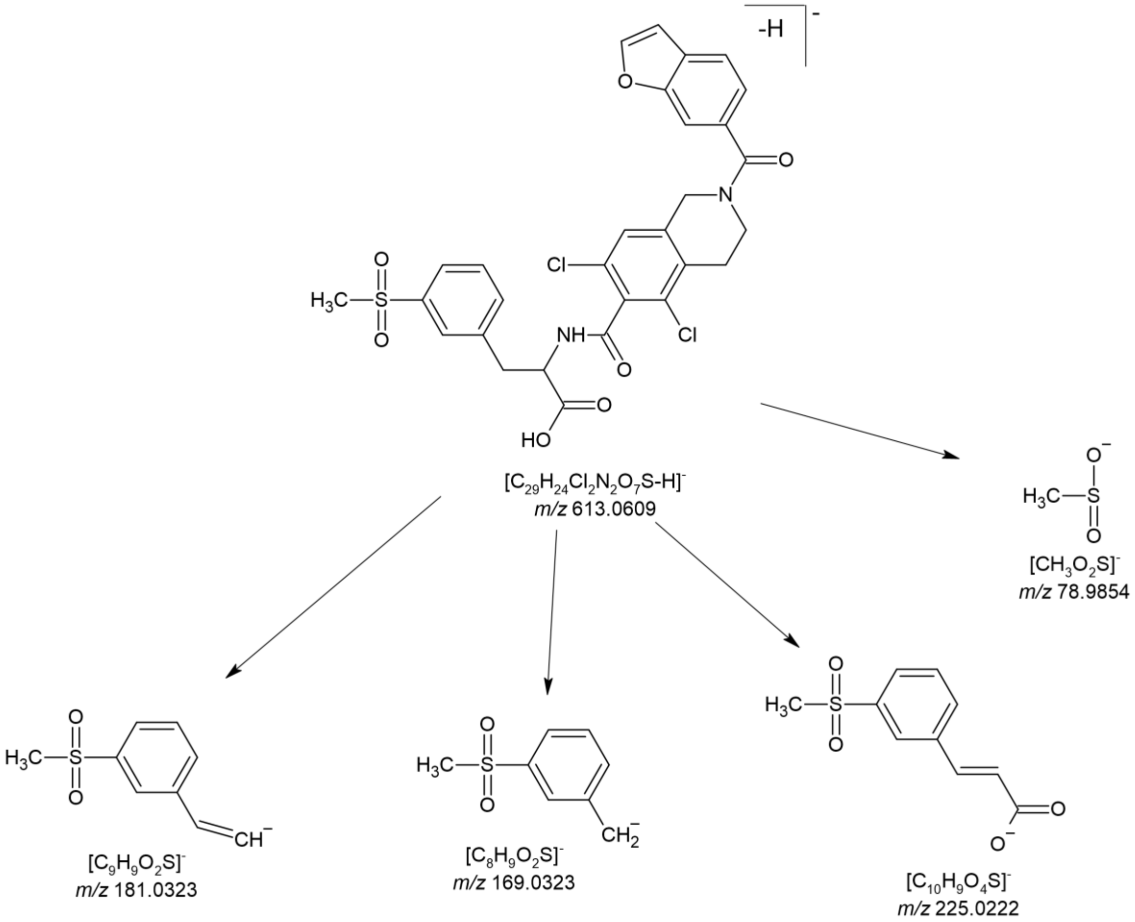

Fig. S29. Possible fragmentation pathway of lifitegrast, ESI-

S3.2.2. MS chromatograms of oxidative degradation mixture

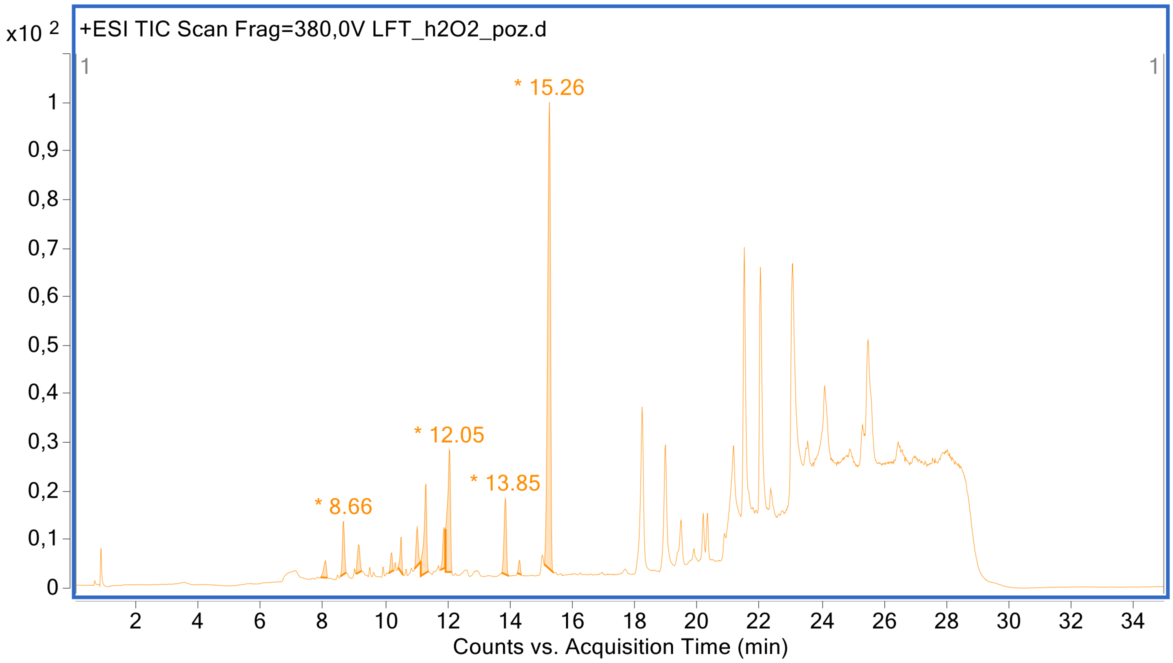

Fig. S30. ESI+ total ion chromatogram of the Lifitegrast degradation products mixture after extended oxidative degradation with 3% H_2O_2 after 30 days at 50 °C/75% RH

S3.2.3. Lifitegrast

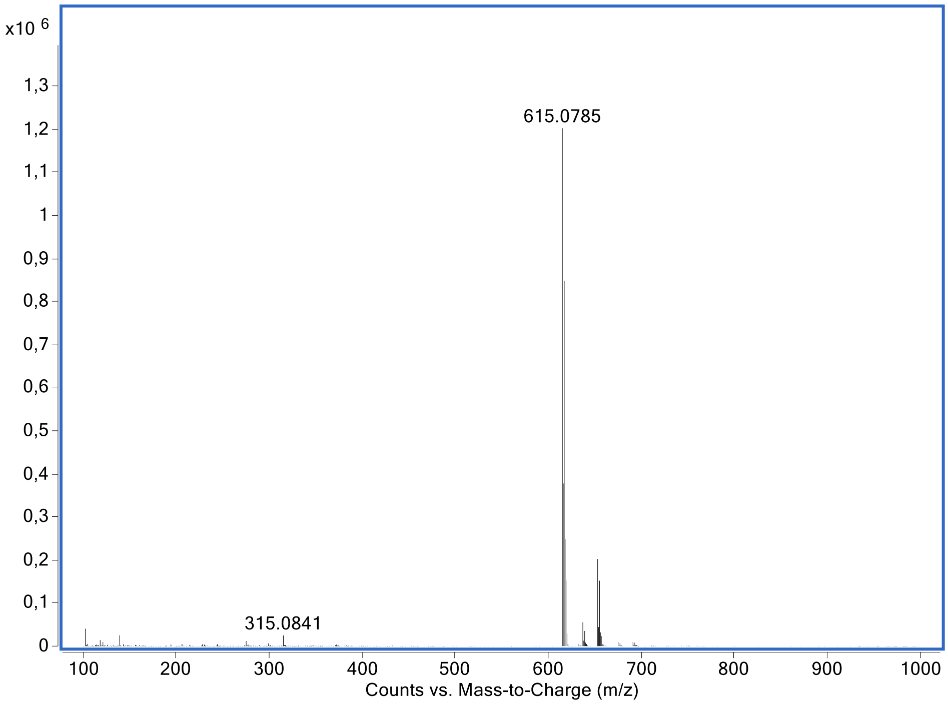

Fig. S31. ESI+ MS spectrum of Lifitegrast

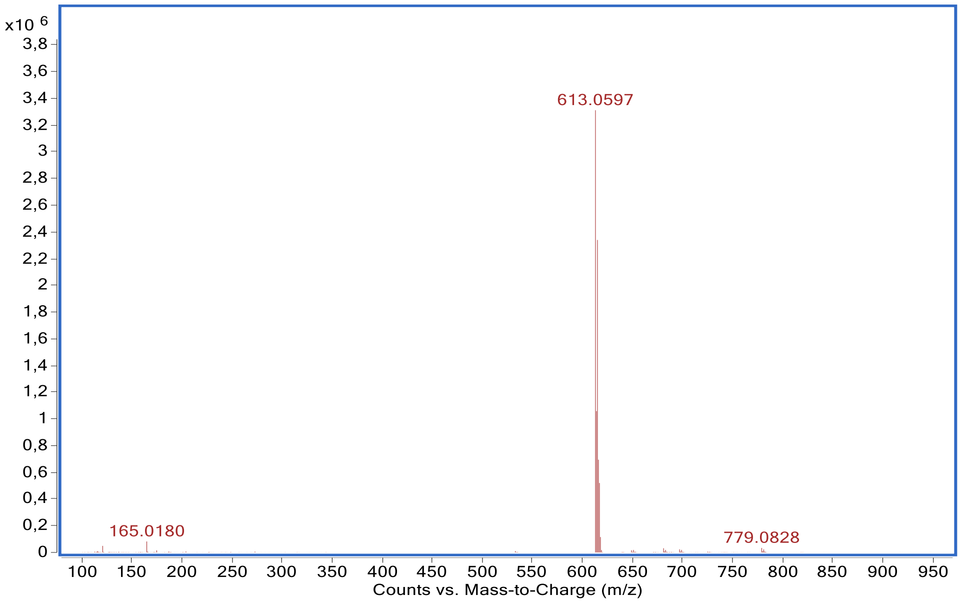

Fig. S32. ESI- MS spectrum of Lifitegrast

S3.2.4. DP1

Chromatographic peak of DP1 (ESI+ m/z 471.0561; ESI– m/z 469.0383) was observed at retention times of 8.66 minutes.

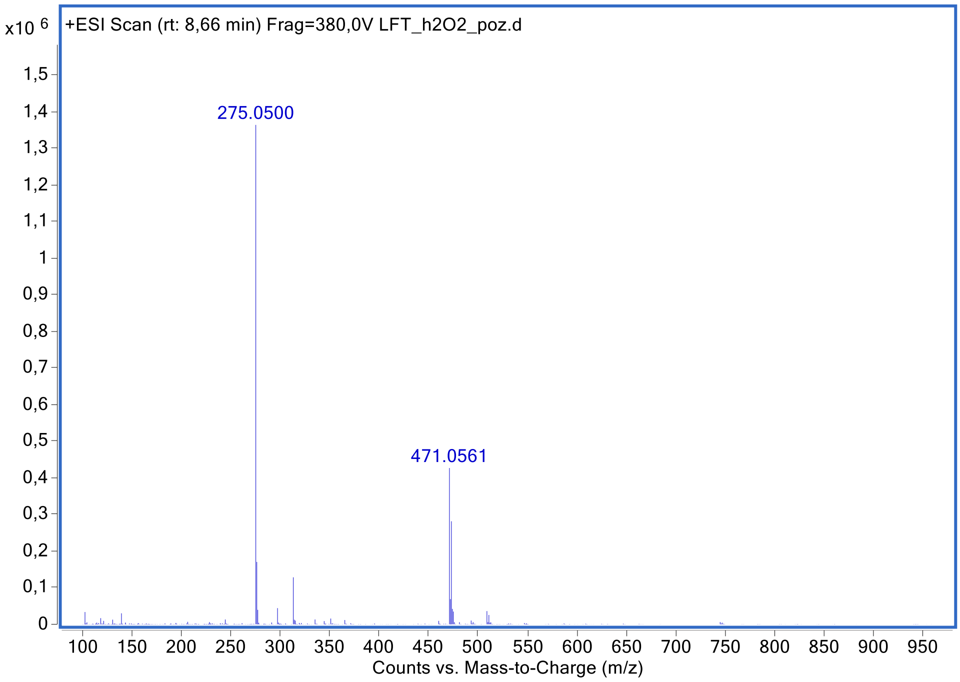

Fig. S33. ESI+ MS spectrum of DP1

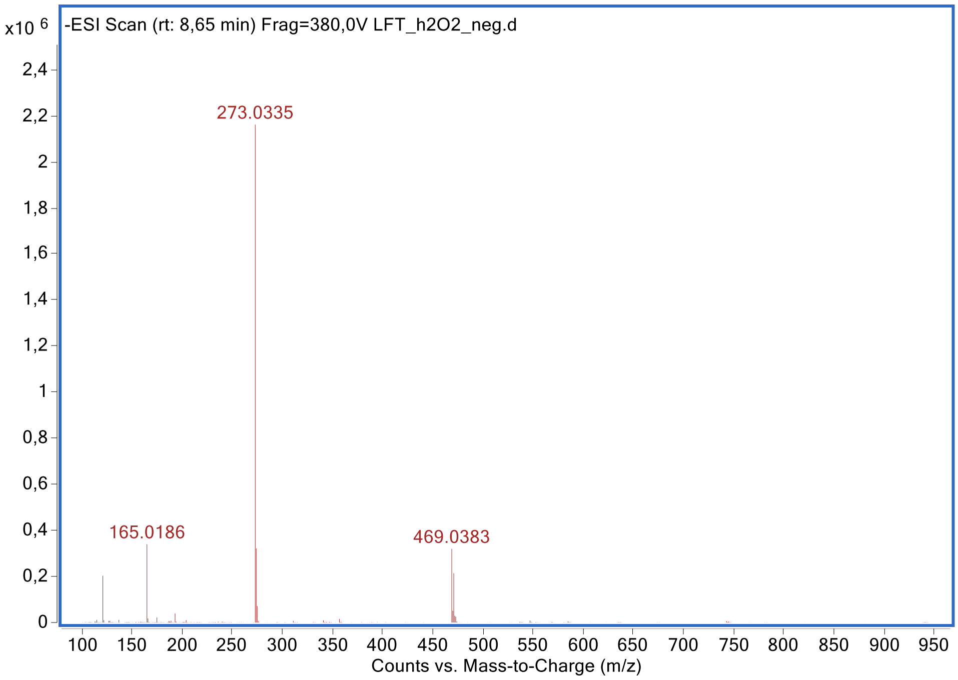

Fig. S34. ESI- MS spectrum of DP1

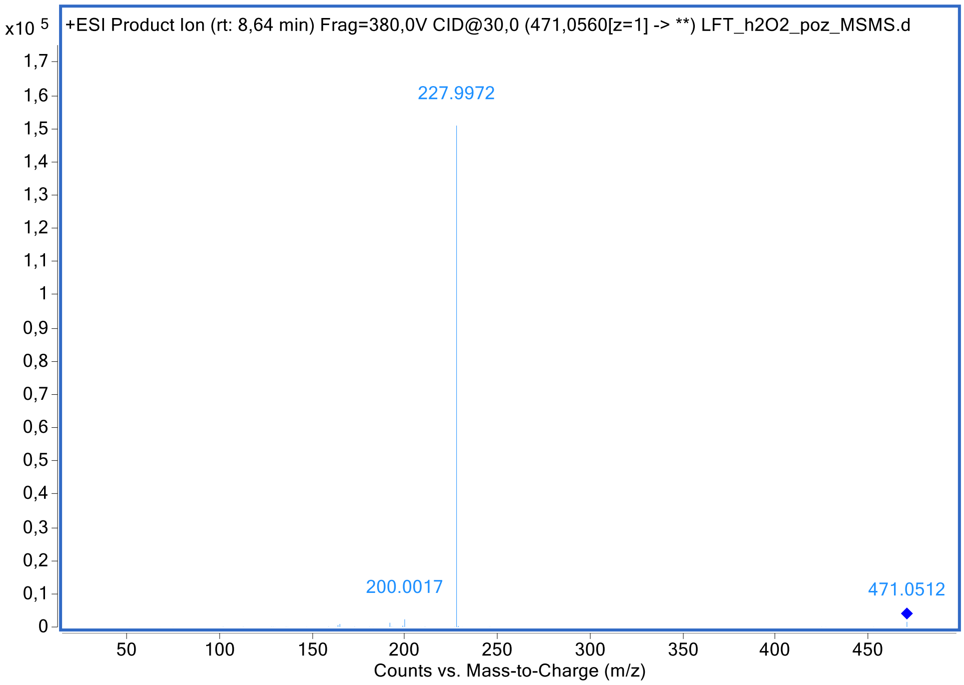

Fig. S35. ESI+ MS/MS spectrum of DP1, collision energy 30 V

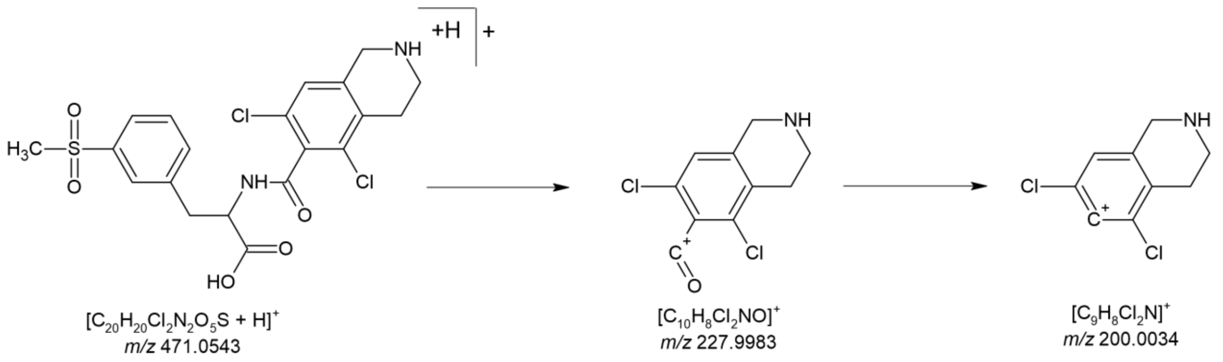

Fig. S36. Possible fragmentation pathway of DP1

S3.2.5. DP3

A degradation product, designated as DP3, was detected at a retention time of 13.86 minutes (ESI+ m/z 619.0711; ESI– m/z 617.0581).

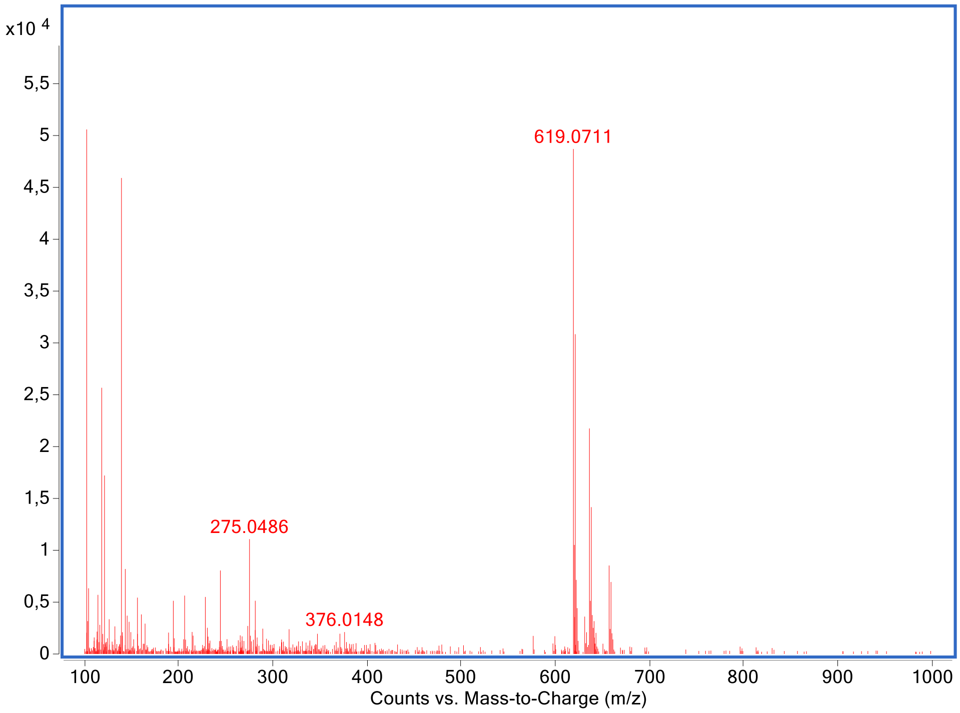

Fig. S37. ESI+ MS spectrum of DP3

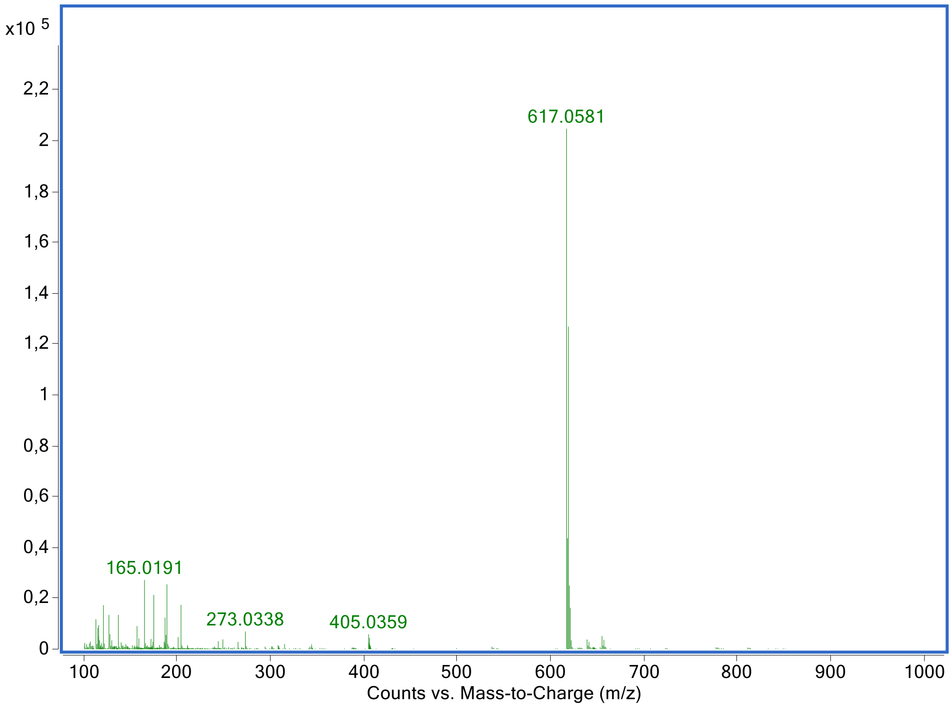

Fig. S38. ESI- MS spectrum of DP3

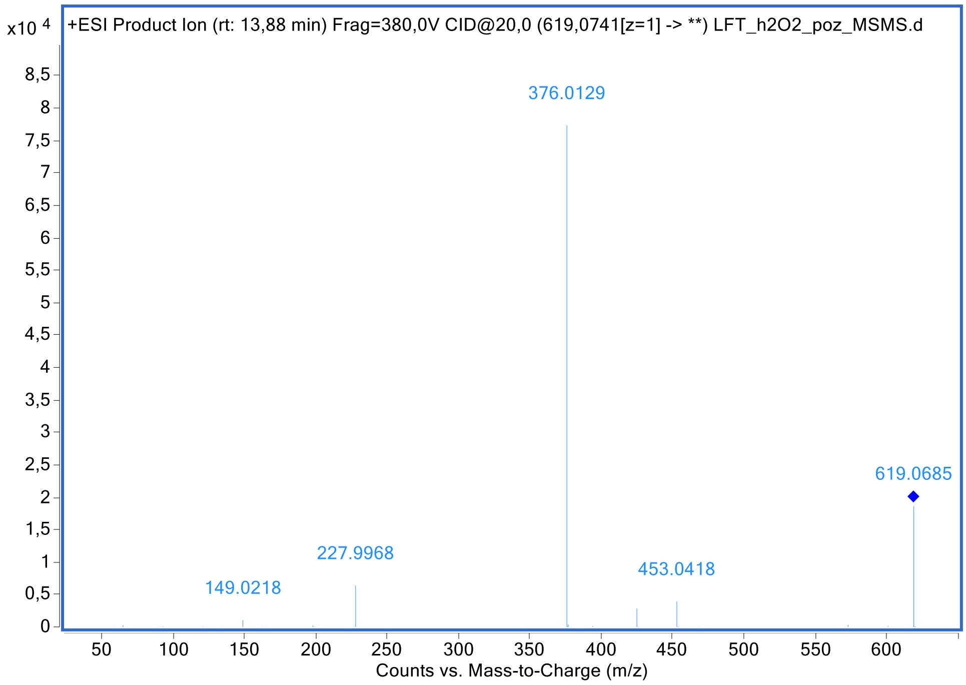

Fig. S39. ESI+ MS/MS spectrum of DP3, collision energy 20 V

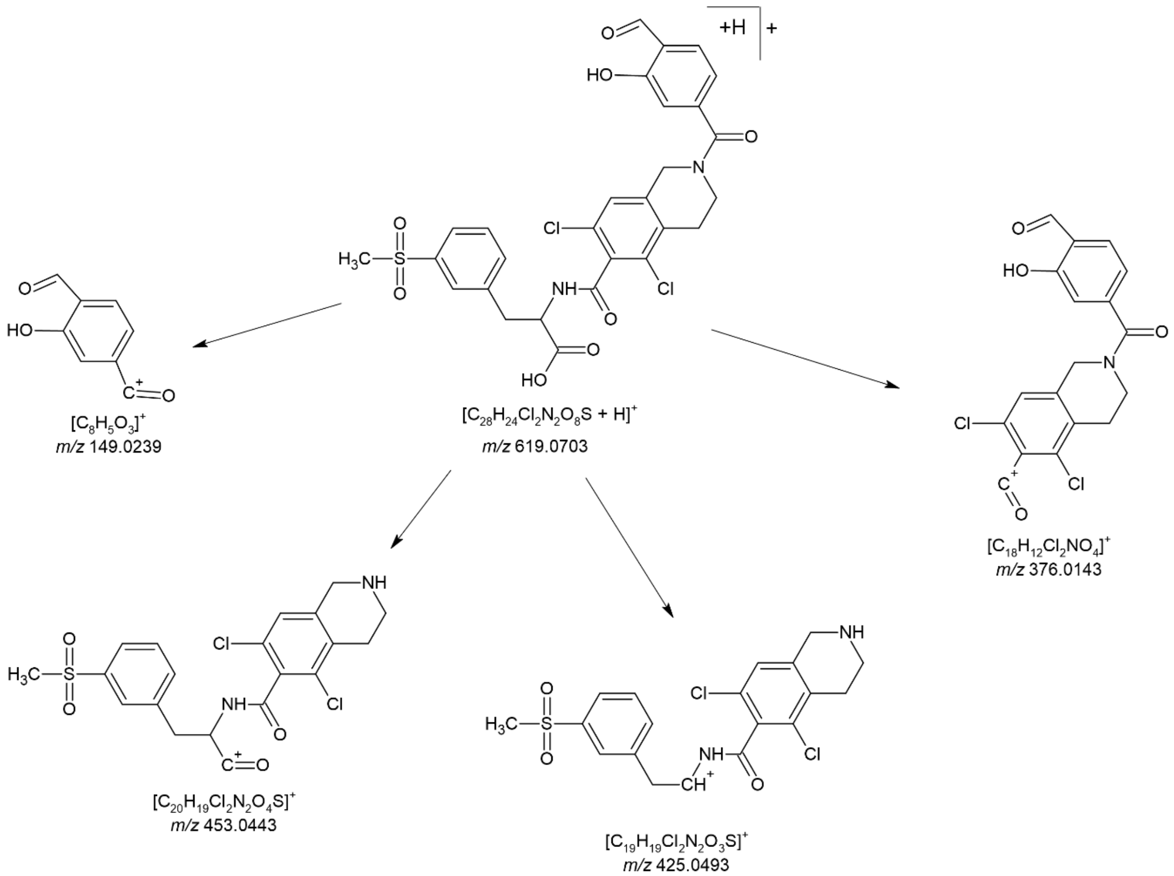

Fig. S40. Possible fragmentation pathway of DP3

S3.2.6. DP4

A degradation product, designated as DP4, was detected at a retention time of 12.06 minutes (ESI+ m/z 607.0735; ESI– m/z 605.0543).

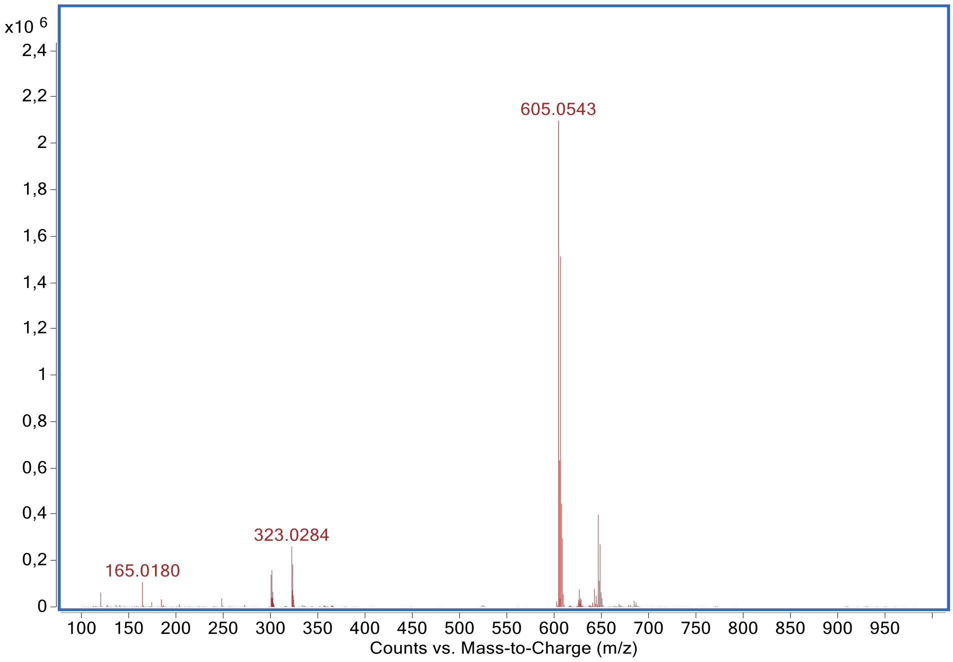

Fig. S41. ESI+ MS spectrum of DP4

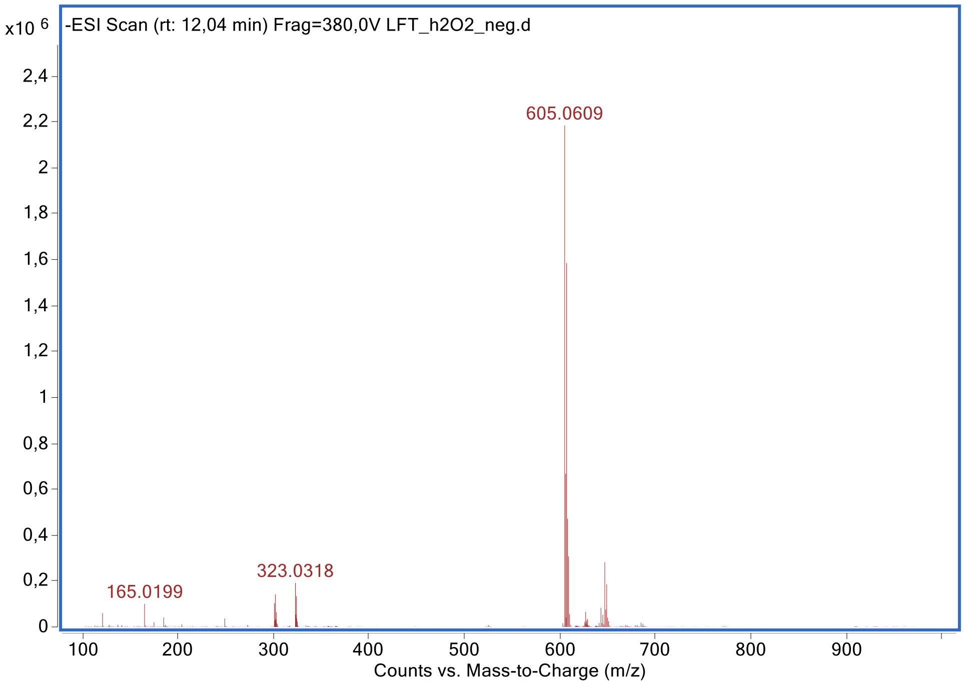

Fig. S42. ESI- MS spectrum of DP4

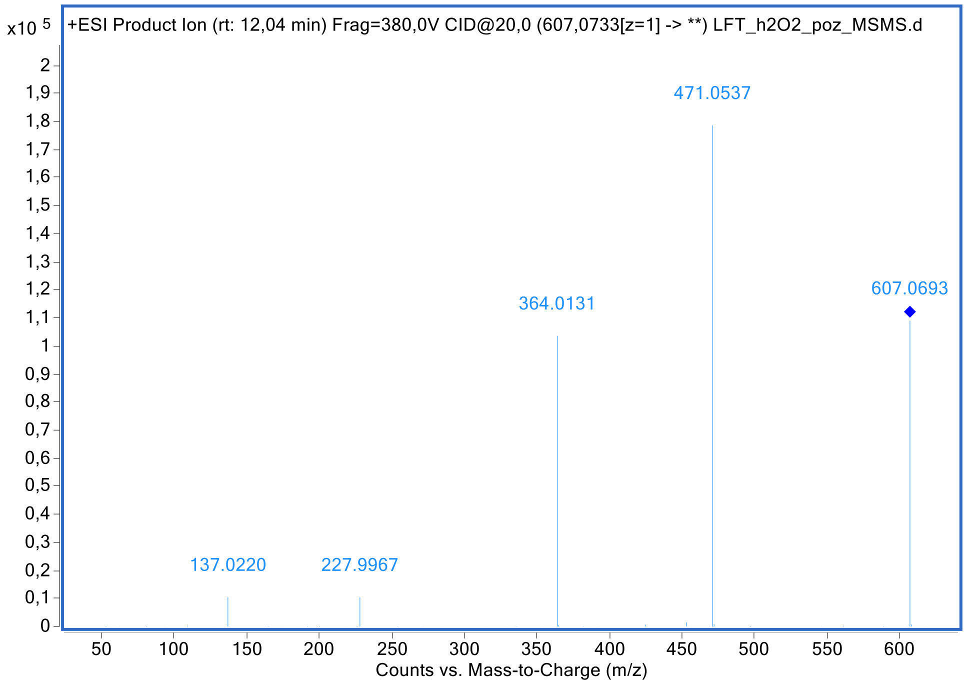

Fig. S43. ESI+ MS/MS spectrum of DP4, collision energy 20 V

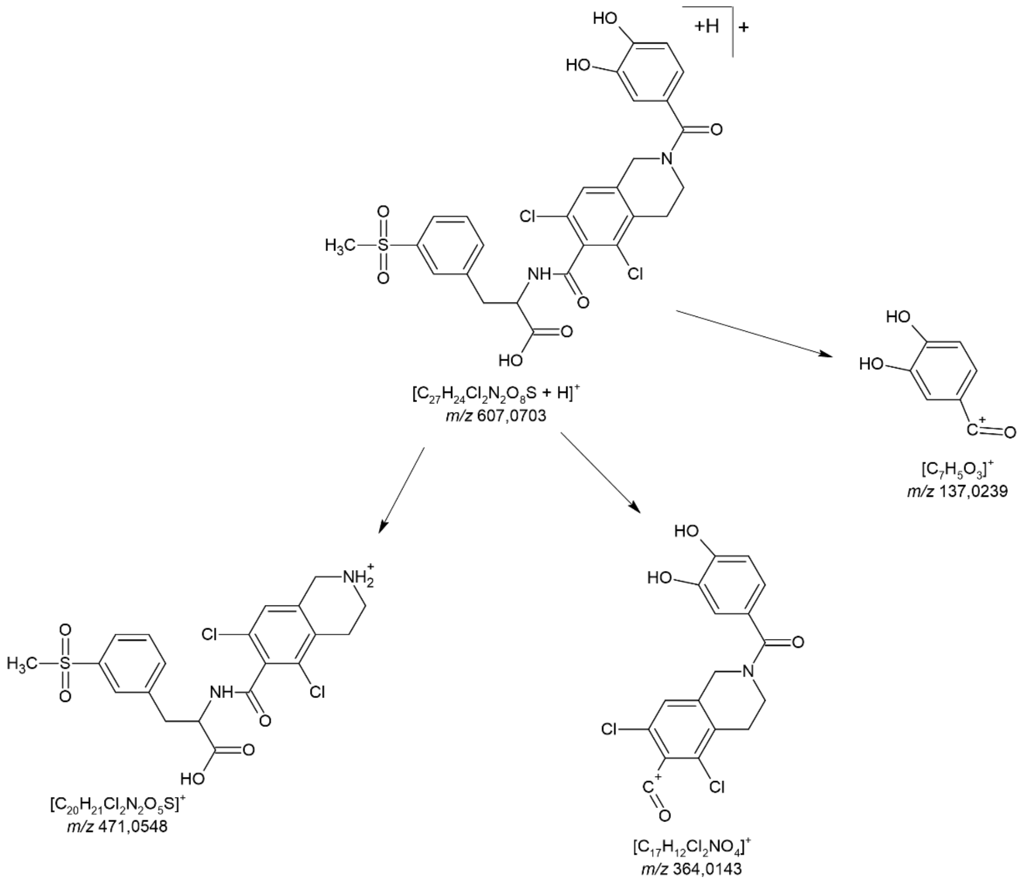

Fig. S44. Possible fragmentation pathway of DP4

S3.2.7. DP5

A degradation product, designated as DP5 was observed at a retention time of 11.30 minutes (ESI+ m/z 635.0682; ESI– m/z 633.0549).

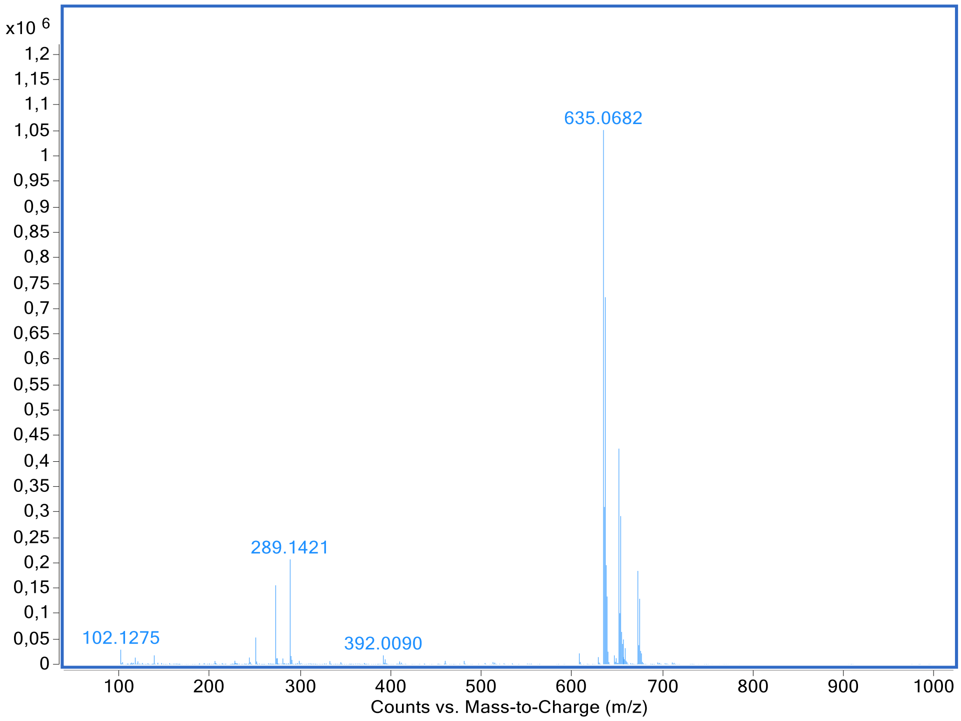

Fig. S45. ESI+ MS spectrum of DP5

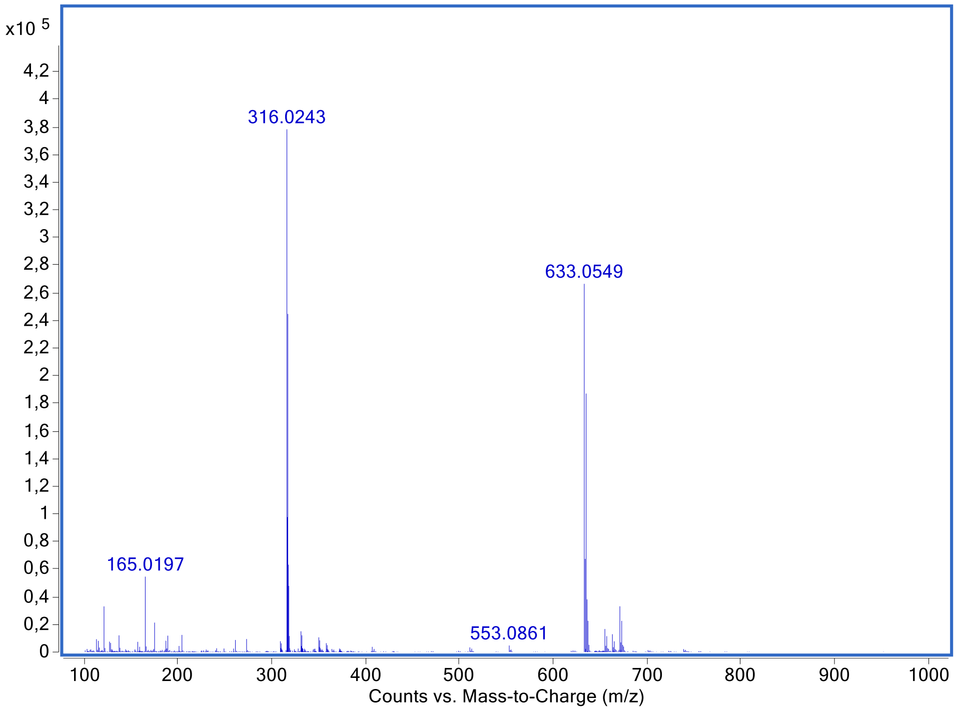

Fig. S46. ESI- MS spectrum of DP5

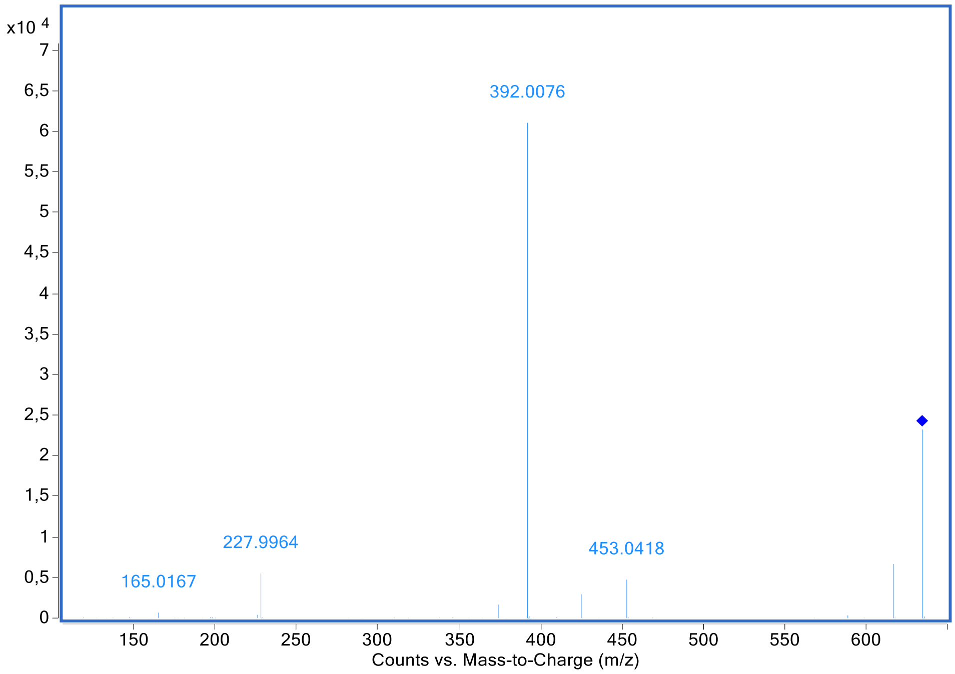

Fig. S47. ESI+ MS/MS spectrum of DP5, collision energy 20 V

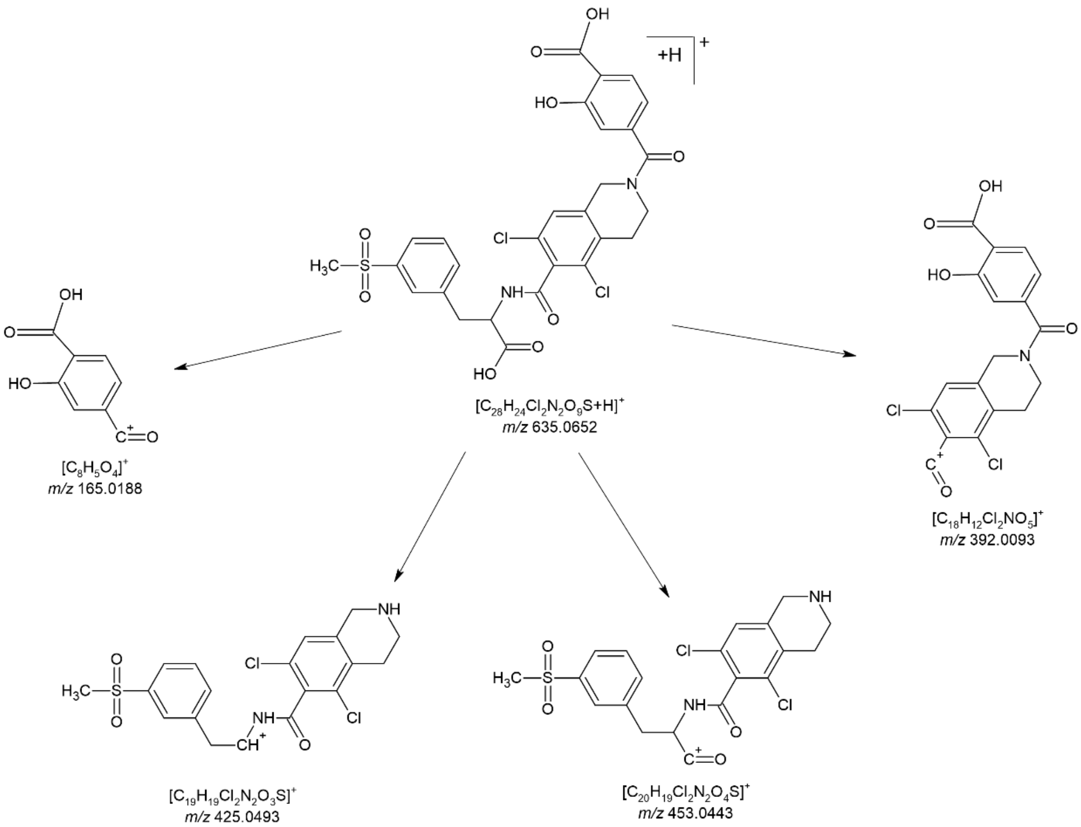

Fig. S48. Possible fragmentation pathway of DP5

S3.2.8. DP7

A degradation product, designated as DP7 was detected at a retention time of 14.31 minutes (ESI+ m/z not observed; ESI- m/z 629.0589).

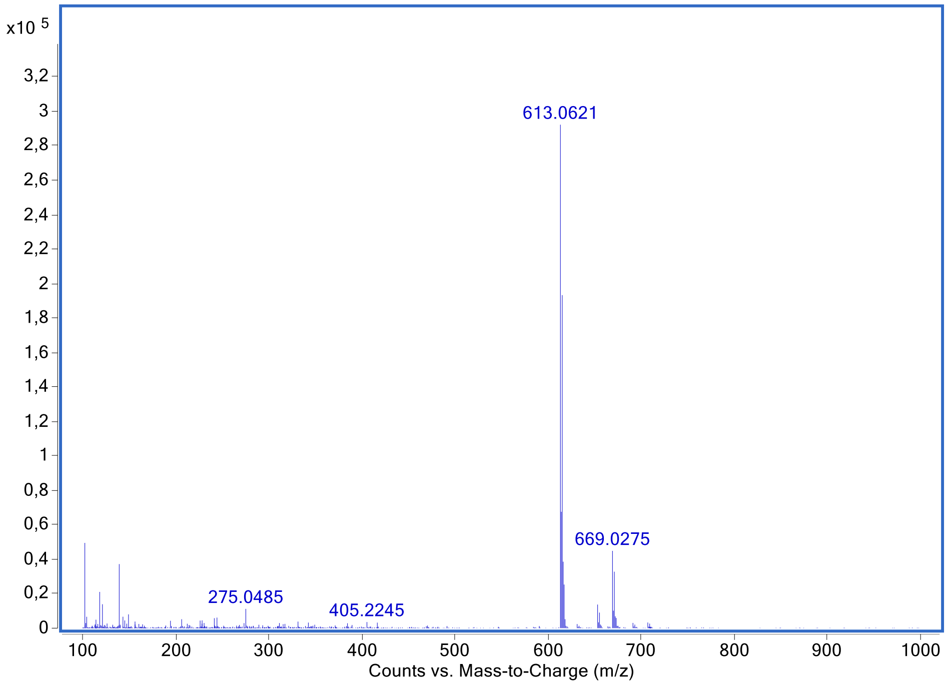

Fig. S49. ESI+ MS spectrum of DP7

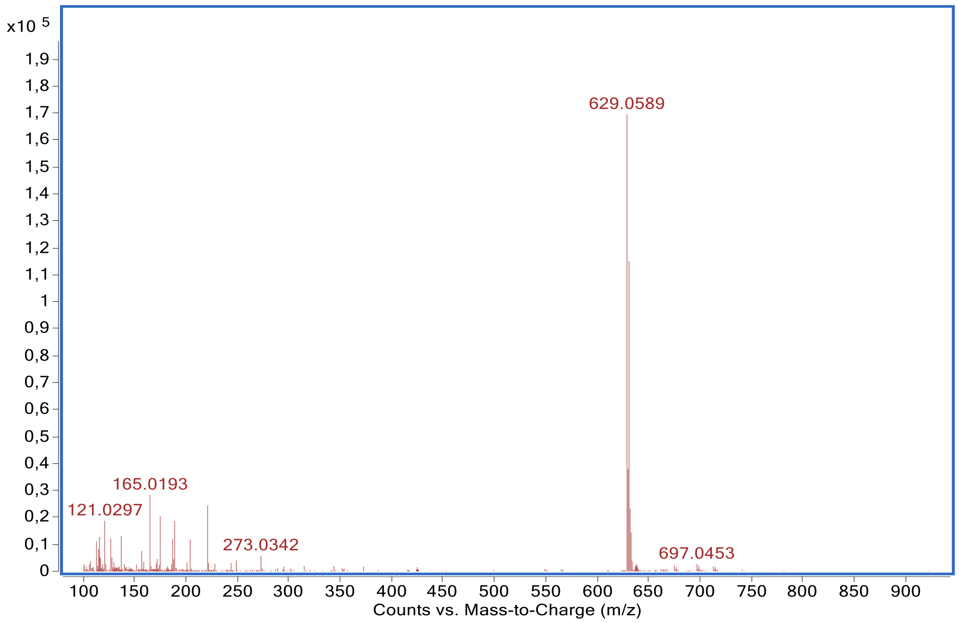

Fig. S50. ESI- MS spectrum of DP7

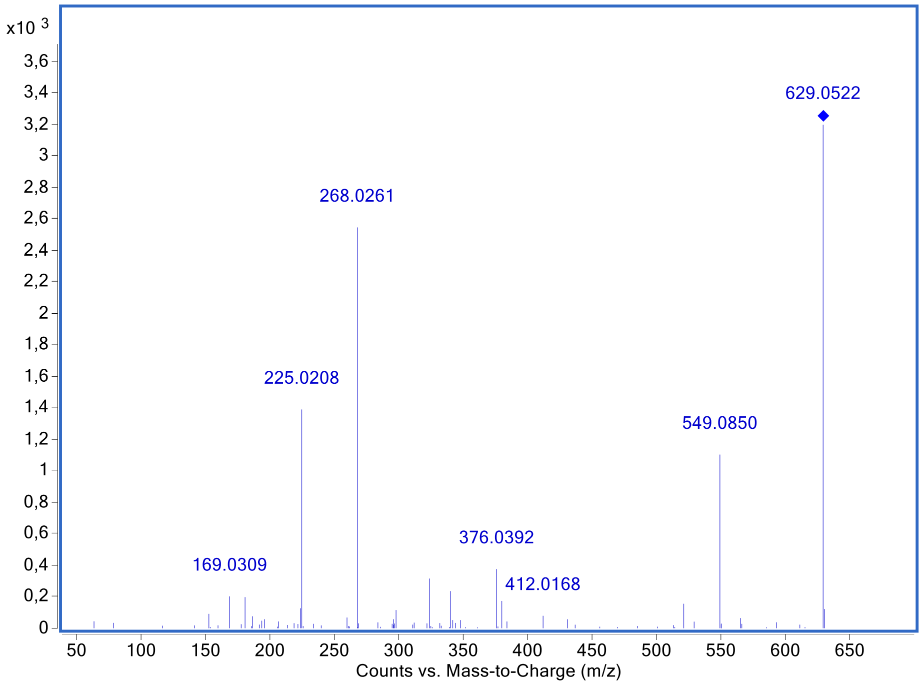

Fig. S51. ESI- MS/MS spectrum of DP7, collision energy 20 V

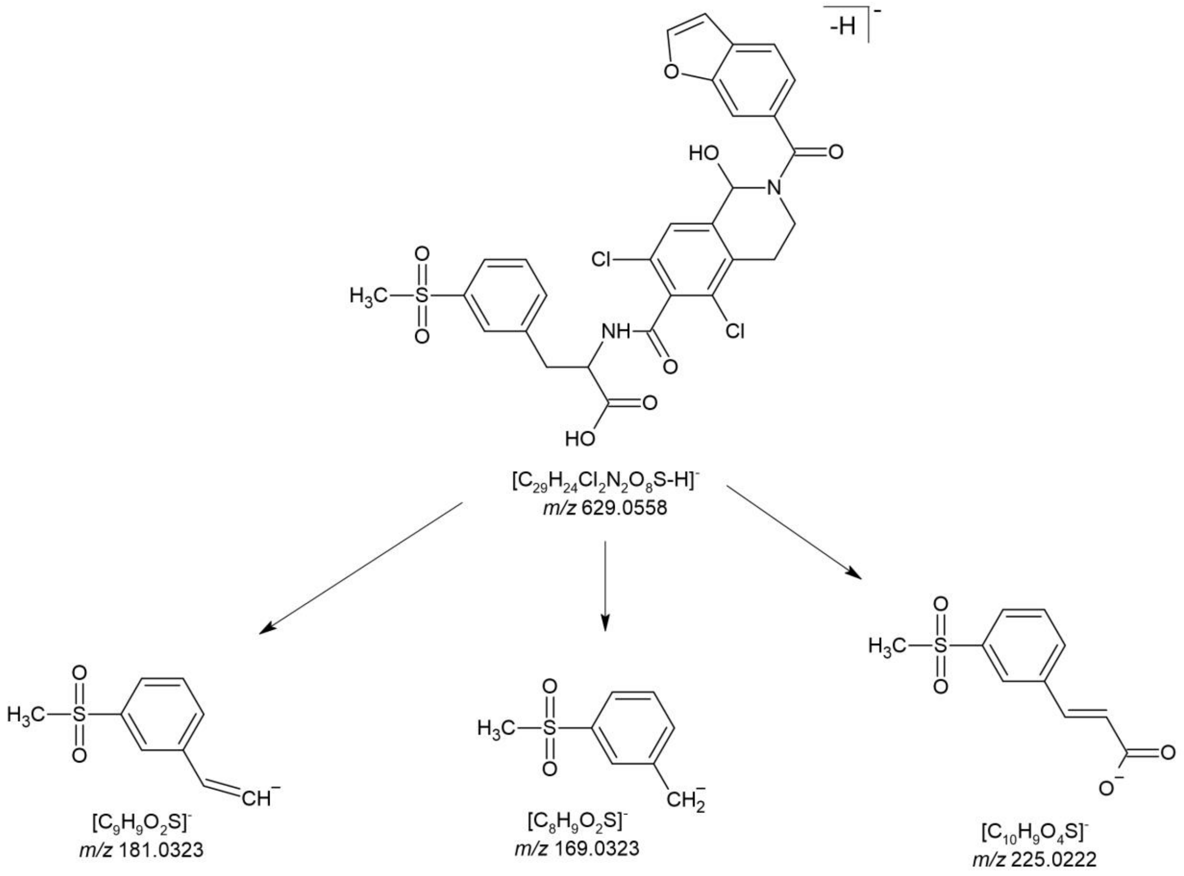

Fig. S52. Possible fragmentation pathway of DP7

S3.2.9. DP8

The chromatographic peak at a retention time of 10.51 minutes corresponds to the degradation product designated as DP8 (ESI+ m/z 485.0357; ESI– m/z 483.0208).

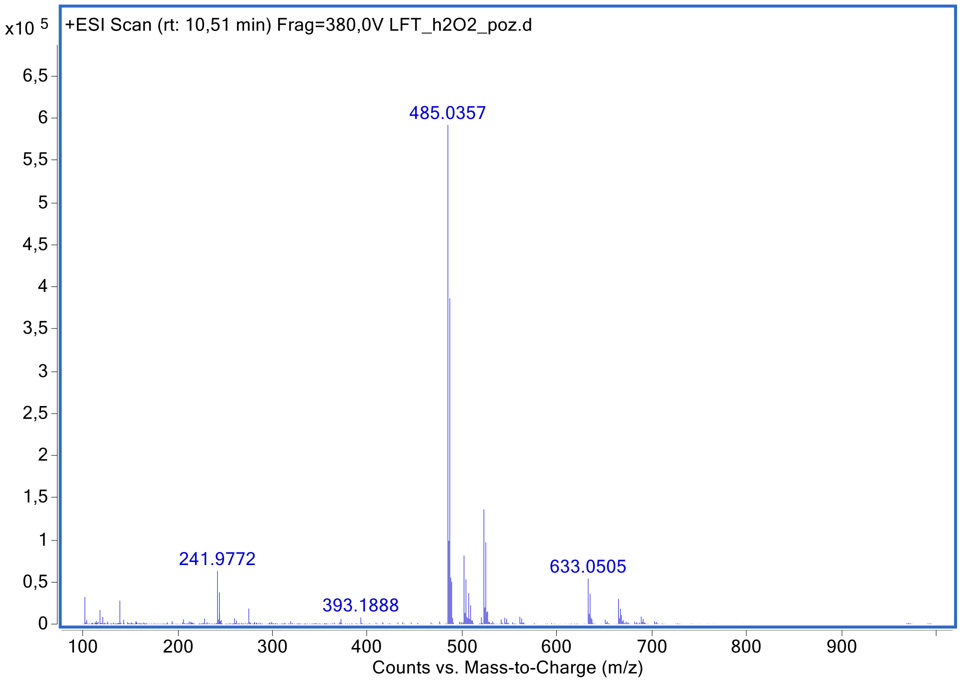

Fig. S53. ESI+ MS spectrum of DP8

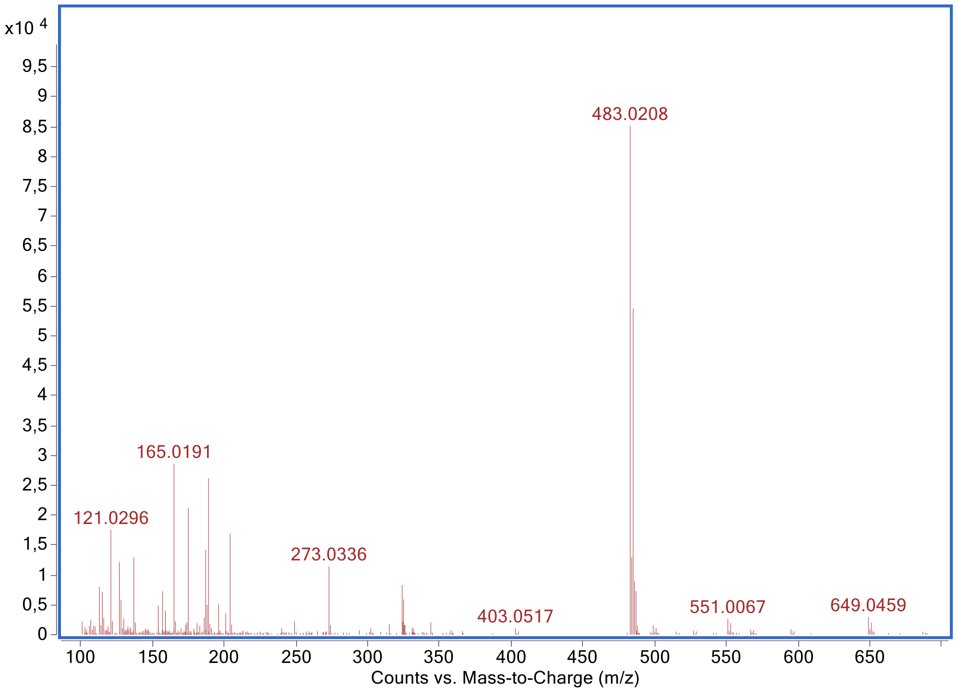

Fig. S54. ESI- MS spectrum of DP8

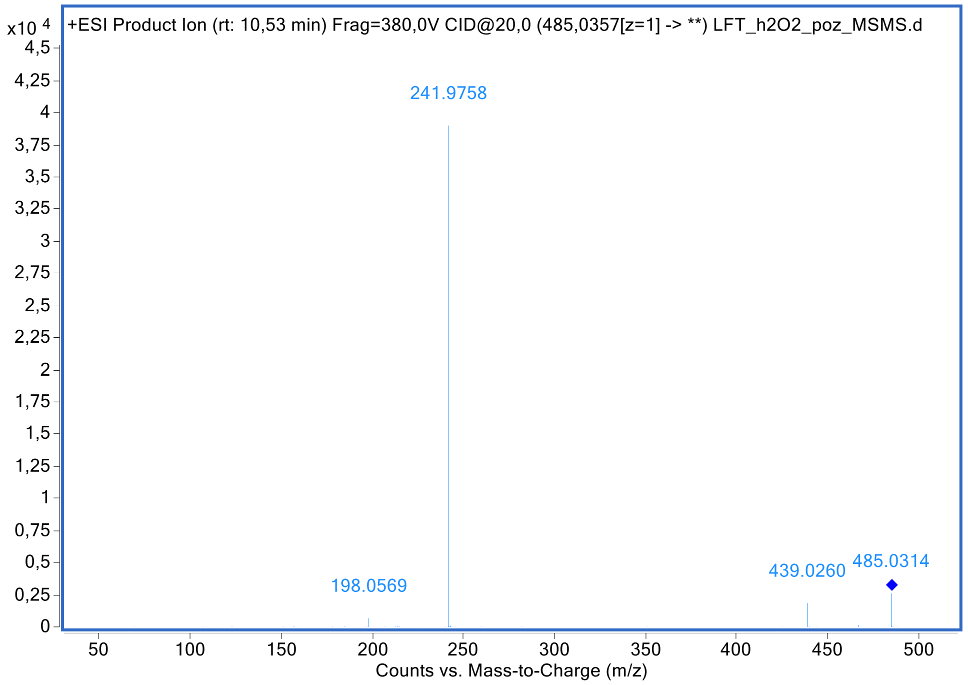

Fig. S55. ESI+ MS/MS spectrum of DP8, collision energy 20 V

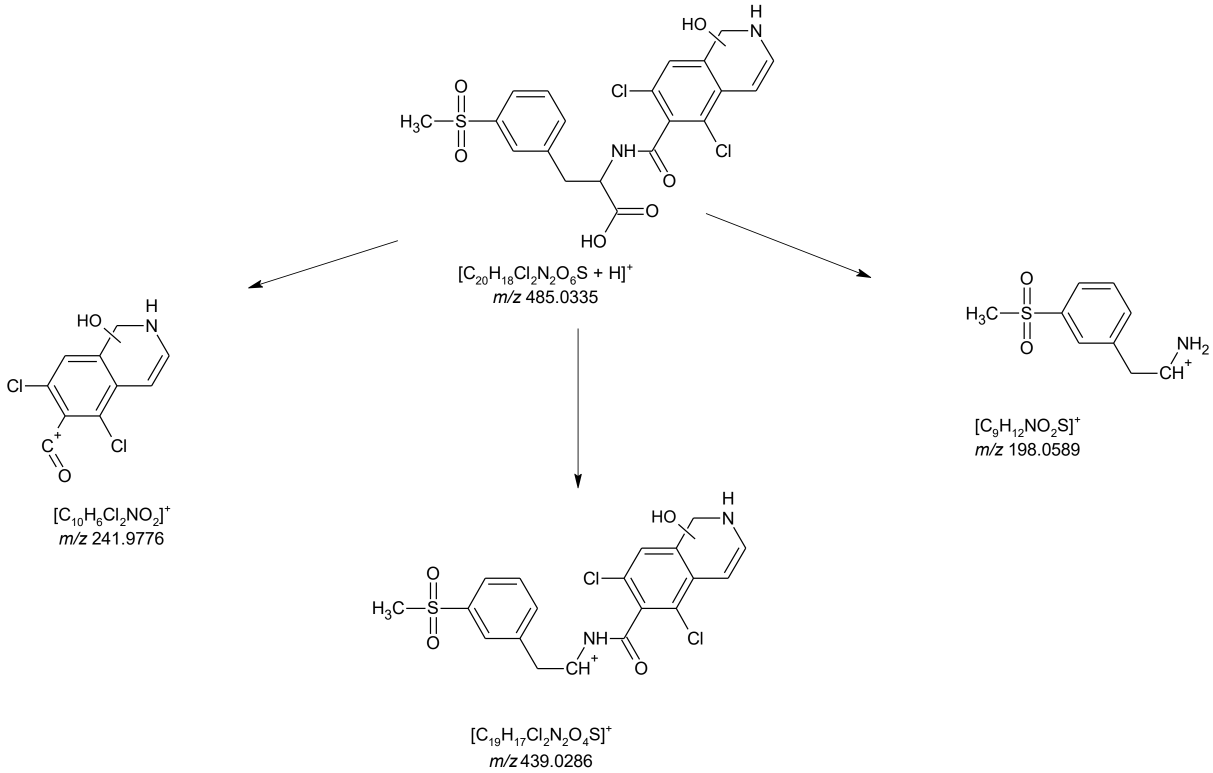

Fig. S56. Possible fragmentation pathway of DP8

S4. Extreme oxidative degradation

Extreme degradation was carried out under oxidative conditions over a period of three months. A solution of Lifitegrast in hydrogen peroxide (H_2O_2) was prepared at a 1:6 ratio, by dissolving 1 g of Lifitegrast in 18 ml of 30% H_2O_2. The solution was stored in a stability chamber under controlled conditions (50 °C/75% RH). Three fractions containing degradation products were separated using thin-layer chromatography (TLC) and column chromatography. Silica gel Kieselgel 60 F_{254} (Merck KGaA, Darmstadt, Germany) was employed as the stationary phase, and a mixture of ethyl acetate and ethanol (EtOAc:EtOH, 4:6) was used as the mobile phase for both chromatographic techniques. Visualization of the separated spots was performed using a UV lamp at 254 nm or iodine (I_2) vapor.

S4.1. NMR analysis

S4.1.1. DP9 (fraction 1)

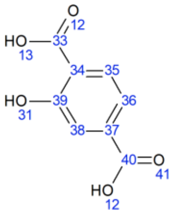

Shift1 (ppm)	H's	Type	J (Hz)	Atom1	Multiplet1
7.171	1.000	s	-	38	M05
7.178	1.000	dd	8.0, 1.0	36	M03
7.727	1.000	d	8.1	35	M04

Atom	Exp. Shift (ppm)
36	116.72
38	116.82
34	124.55
35	129.75
37	134.18
39	162.44
40	168.13
33	170.82

^{1}H NMR (600 MHz, DMSO-d_6): δ = 7.17 (s, 1 H, 38), 7.18 (dd, J = 8.0, 1.0 Hz, 1 H, 36), 7.73 (d, J = 8.1 Hz, 1 H, 35) ppm

^{13}C NMR (151 MHz, DMSO-d_6): δ = 116.7 (36), 116.8 (38), 124.5 (34), 129.7 (35), 134.2 (37), 162.4 (39), 168.1 (40), 170.8 (33) ppm

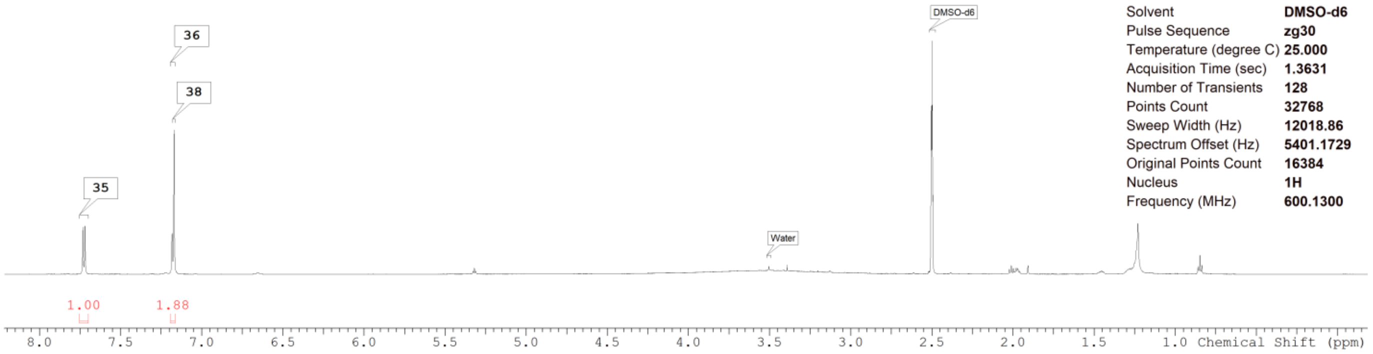

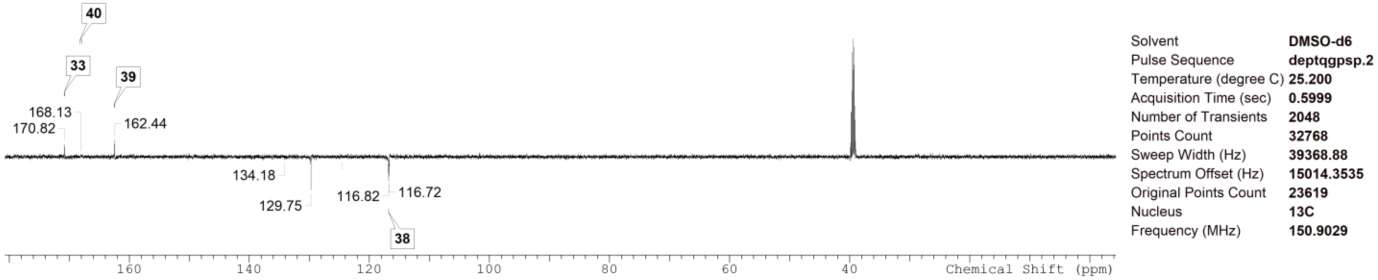

Fig. S57. Fully assigned ^{1}H and ^{13}C spectra of DP9 in DMSO-d_6 at 25 °C

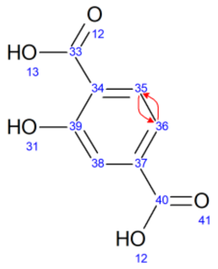

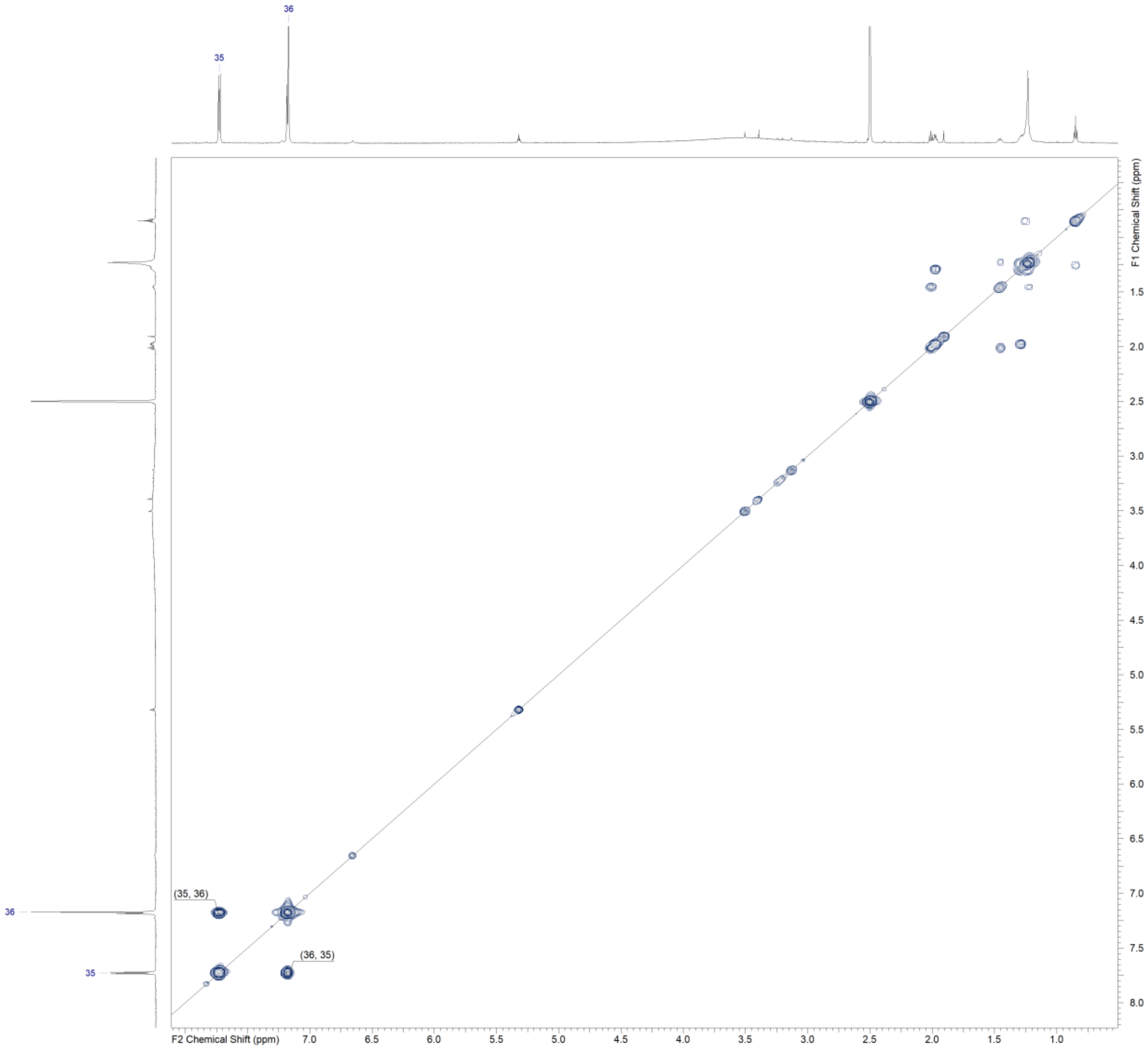

Acquisition Time (sec)	(0.1065, 0.0267)
Date	30 Jul 2023 08:56:10
Frequency (MHz)	(600.1300, 600.1300)
Nucleus	(1H, 1H)
Points Count	(2048, 2048)
Pulse Sequence	cosygpqf
Solvent	DMSO-d6
Sweep Width (Hz)	(9610.69, 9597.45)
Number of Transients	4
Spectrum Type	COSY
Temperature (degree C)	25.000

Fig. S58. Fully assigned ^{1}H-^{1}H COSY spectrum of DP9 in DMSO-d_6 at 25 °C

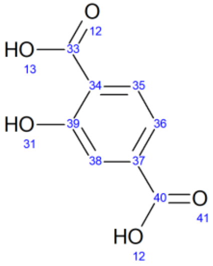

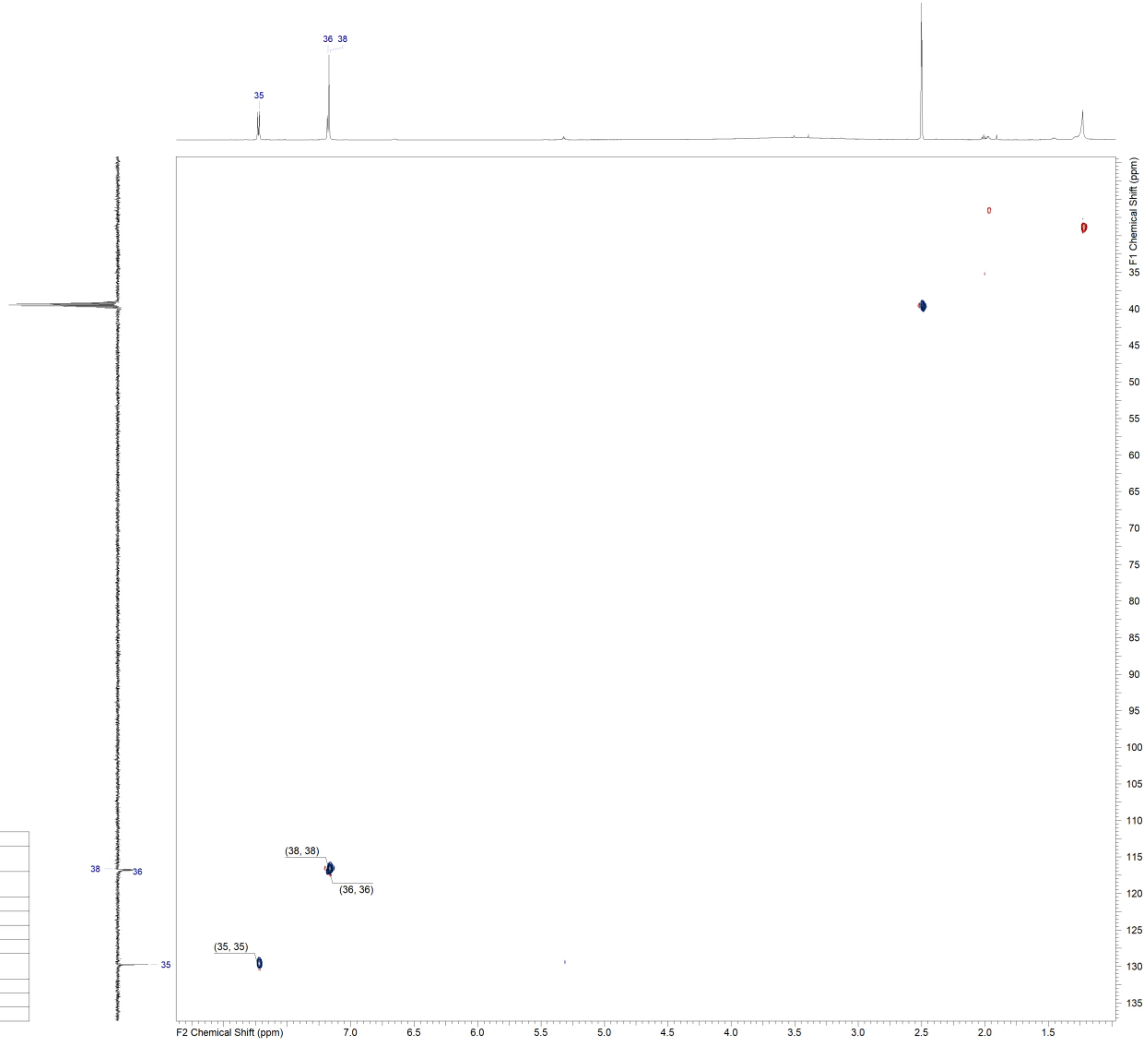

Acquisition Time (sec)	(0.1139, 0.0094)
Date	30 Jul 2023 09:37:10
Frequency (MHz)	(600.1300, 150.9029)
Nucleus	(1H, 13C)
Points Count	(1024, 1024)
Pulse Sequence	hsqcedetgp
Solvent	DMSO-d6
Sweep Width (Hz)	(8984.02, 27138.29)
Number of Transients	8
Spectrum Type	HSQC-DEPT
Temperature (degree C)	25.100

Fig. S59. Fully assigned ^{1}H-^{13}C HSQCe spectrum of DP9 in DMSO-d_6 at 25 °C

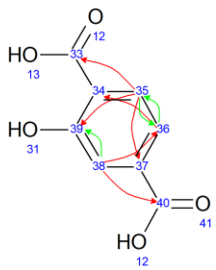

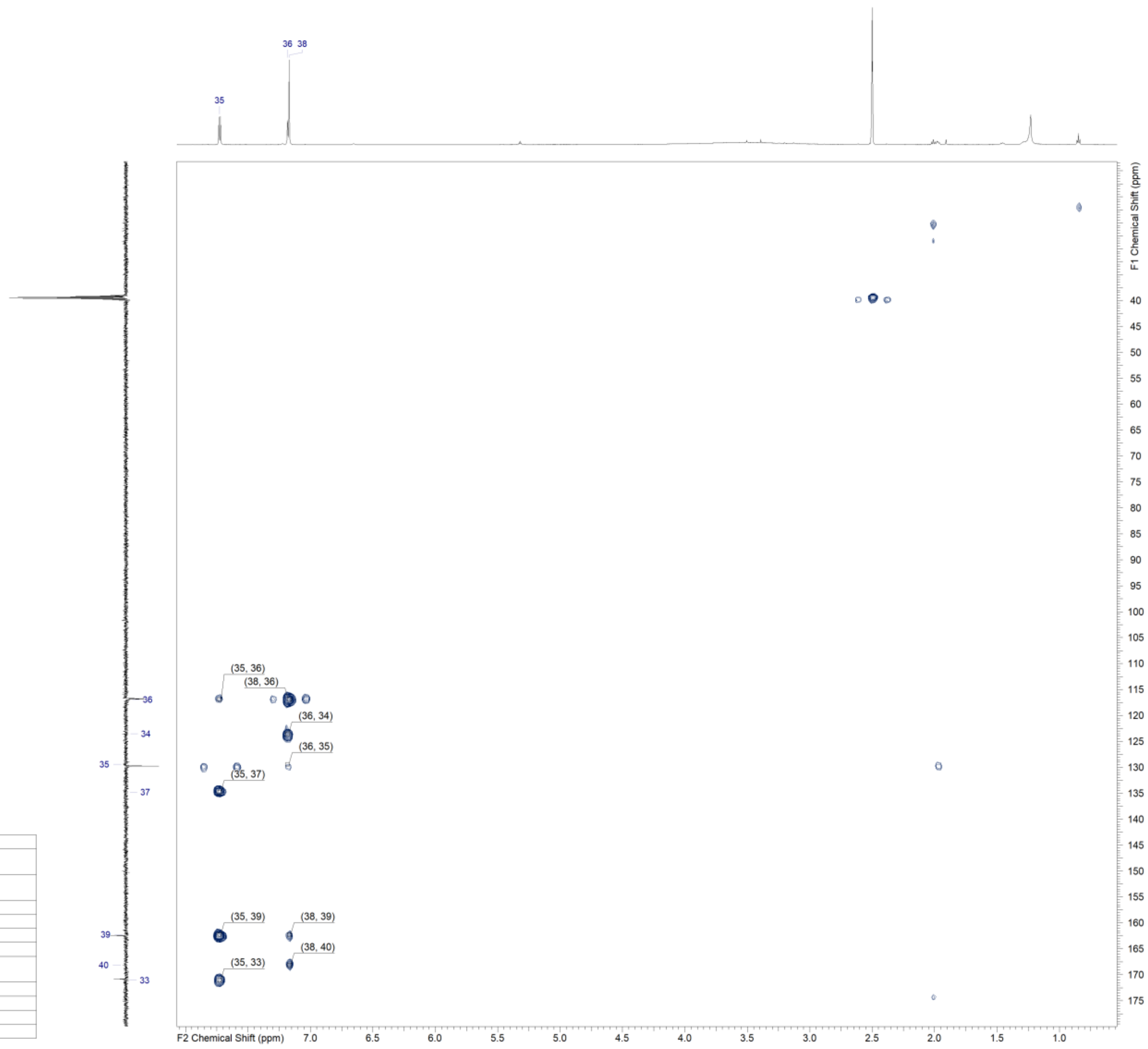

Acquisition Time (sec)	(0.1139, 0.0071)
Date	30 Jul 2023 10:59:36
Frequency (MHz)	(600.1300, 150.9029)
Nucleus	(1H, 13C)
Points Count	(2048, 1024)
Pulse Sequence	hmbcgplpndqf
Solvent	DMSO-d6
Sweep Width (Hz)	(8988.42, 36185.27)
Constant (Hz)	10.0
Number of Transients	16
Spectrum Type	HMBC
Temperature (degree C)	24.900

Fig. S60. Fully assigned ^{1}H-^{13}C HMBC spectrum of DP9 in DMSO-d_6 at 25 °C

S4.1.2. DP10 (fraction 3)

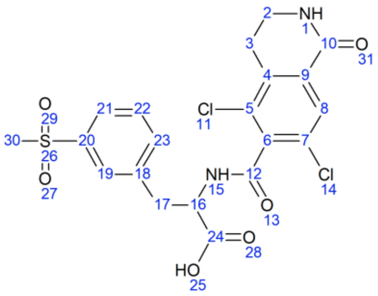

Shift1 (ppm)	H's	Type	J (Hz)	Atom1	Multiplet1
2.939	2.000	br t	6.6	3	M02
3.102	3.000	m	-	30	M03
3.194	1.000	m	-	17<">	M04
3.336	1.000	br dd	13.8, 3.9	17<'>	M05
3.420	2.000	td	6.6, 2.6	2	M06
4.514	1.000	br s	-	16	M14
7.498	1.000	t	7.4	22	M08
7.669	1.000	br d	7.7	23	M09
7.720	1.000	t	7.7	21	M10
7.791	1.000	s	-	8	M11
7.863	1.000	s	-	19	M12
7.932	1.000	br s	-	1	M13
8.050	1.000	br s	-	15	M07

Atom	Exp. Shift (ppm)
24	-
7	-
12	-
3	24.63
17	37.13
2	37.83
30	43.41
16	54.51
21	123.92
8	125.83
19	127.38
22	128.26
4	128.72
5	129.59
23	134.24
9	136.00
6	140.02
18	140.27
20	140.27
10	161.60
Atom	Exp. Shift (ppm)

^{1}H NMR (600 MHz, DMSO-d_6): δ = 2.94 (br t, J = 6.6 Hz, 2 H, 3), 3.07 - 3.13 (m, 3 H, 30), 3.14 - 3.25 (m, 1 H, 17<">), 3.34 (br dd, J = 13.8, 3.9 Hz, 1 H, 17<'>), 3.42 (td, J = 6.6, 2.6 Hz, 2 H, 2), 4.51 (br s, 1 H, 16), 7.50 (t, J = 7.4 Hz, 1 H, 22), 7.67 (br d, J = 7.7 Hz, 1 H, 23), 7.72 (t, J = 7.7 Hz, 1 H, 21), 7.79 (s, 1 H, 8), 7.86 (s, 1 H, 19), 7.93 (br s, 1 H, 1), 8.05 (br s, 1 H, 15) ppm

^{13}C NMR (151 MHz, DMSO-d_6): δ = 24.6 (3), 37.1 (17), 37.8 (2), 43.4 (30), 54.5 (16), 123.9 (21), 125.8 (8), 127.4 (19), 128.3 (22), 128.7 (4), 129.6 (5), 134.2 (23), 136.0 (9), 140.0 (6), 140.3 (20, 18), 161.6 (10) ppm

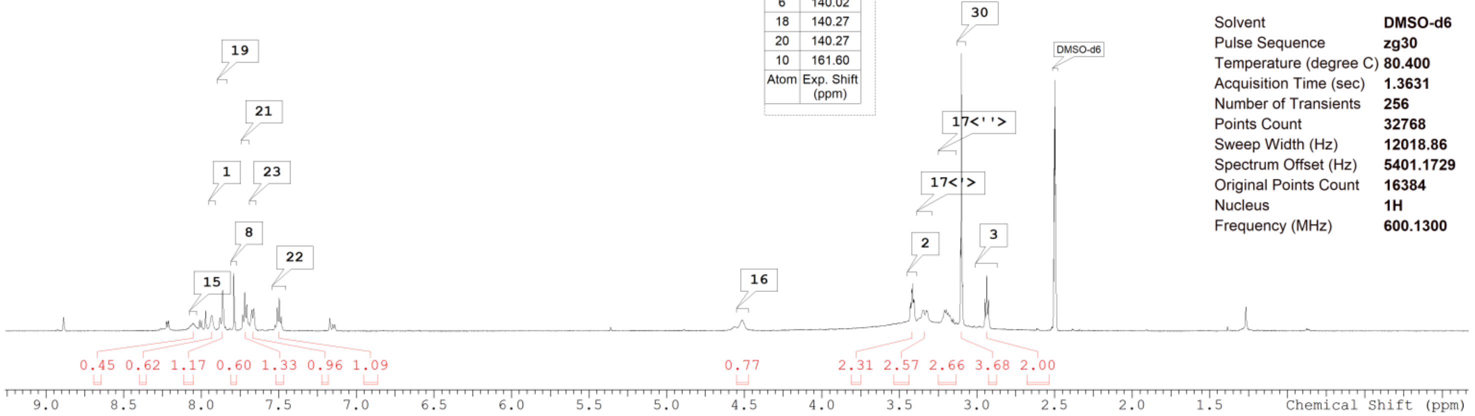

Solvent	**DMSO-d6**
Pulse Sequence	**zg30**
Temperature (degree C)	**80.400**
Acquisition Time (sec)	**1.3631**
Number of Transients	**256**
Points Count	**32768**
Sweep Width (Hz)	**12018.86**
Spectrum Offset (Hz)	**5401.1729**
Original Points Count	**16384**
Nucleus	**1H**
Frequency (MHz)	**600.1300**

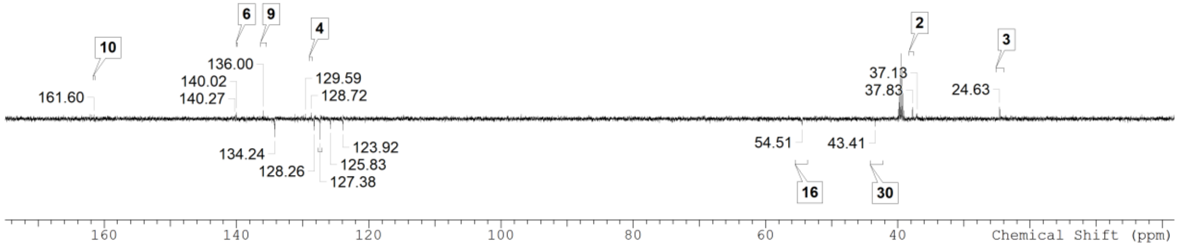

Solvent	**DMSO-d6**
Pulse Sequence	**deptqgpsp.2**
Temperature (degree C)	**80.200**
Acquisition Time (sec)	**0.5999**
Number of Transients	**4096**
Points Count	**32768**
Sweep Width (Hz)	**39368.88**
Spectrum Offset (Hz)	**14951.0342**
Original Points Count	**23619**
Nucleus	**13C**
Frequency (MHz)	**150.9029**

Fig. S61. Fully assigned ^{1}H and ^{13}C spectra, structure, numbering and full assignment of DP10 in DMSO-d_6 at 80 °C

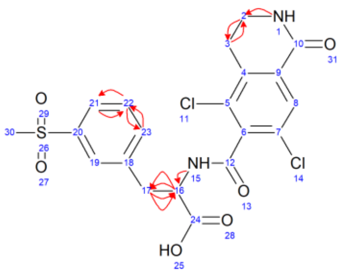

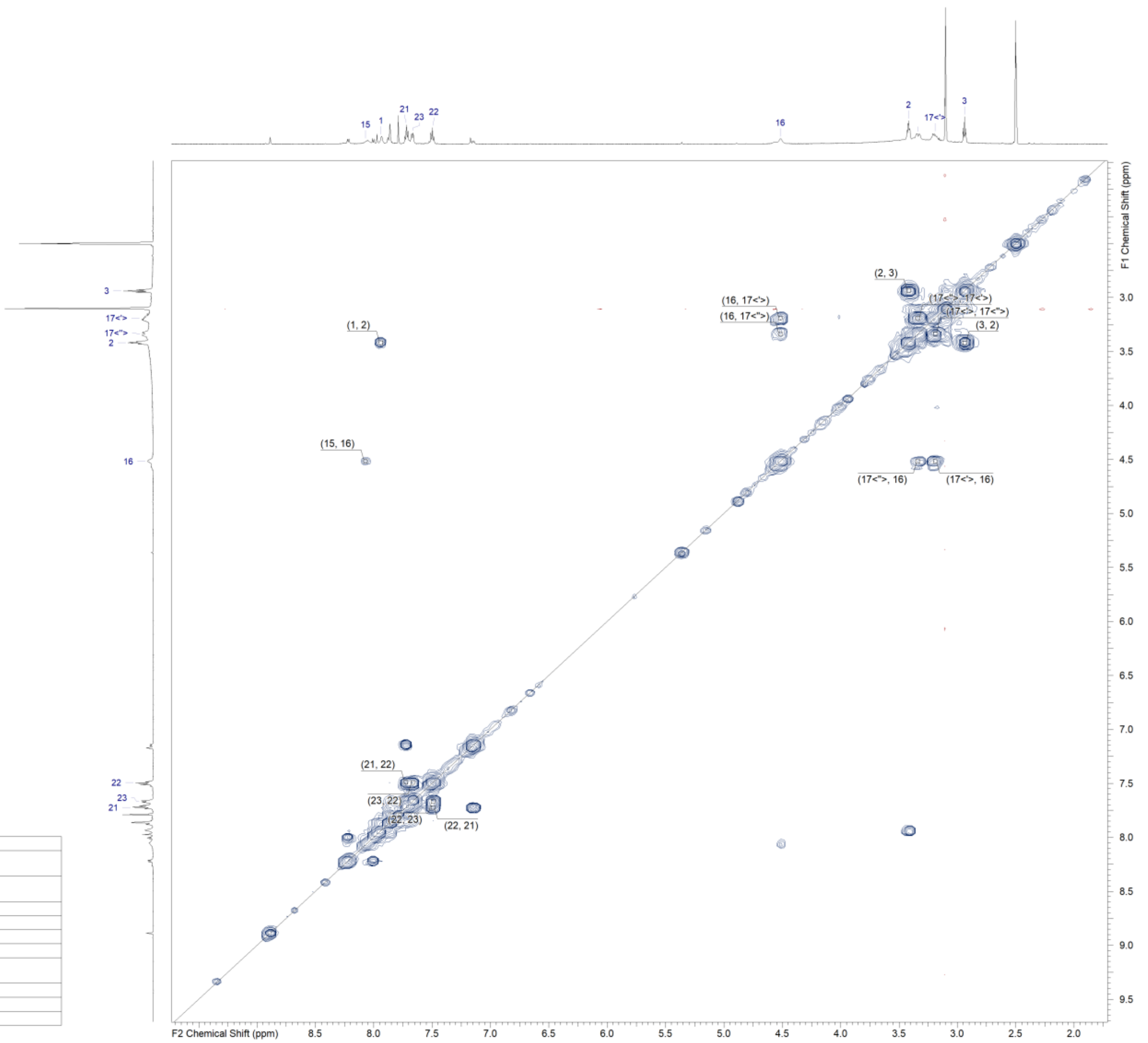

**Acquisition Time (sec)**	(0.1065, 0.0267)
**Date**	02 Aug 2023 13:40:32
**Frequency (MHz)**	(600.1300, 600.1300)
**Nucleus**	(1H, 1H)
**Points Count**	(2048, 2048)
**Pulse Sequence**	cosygpqf
**Solvent**	DMSO-d6
**Sweep Width (Hz)**	(9610.69, 9597.45)
**Number of Transients**	4
**Spectrum Type**	COSY
**Temperature (degree C)**	80.400

Fig. S62. Fully assigned ^{1}H-^{1}H COSY spectrum of DP10 in DMSO-d₆ at 80 °C

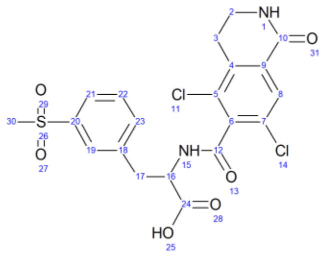

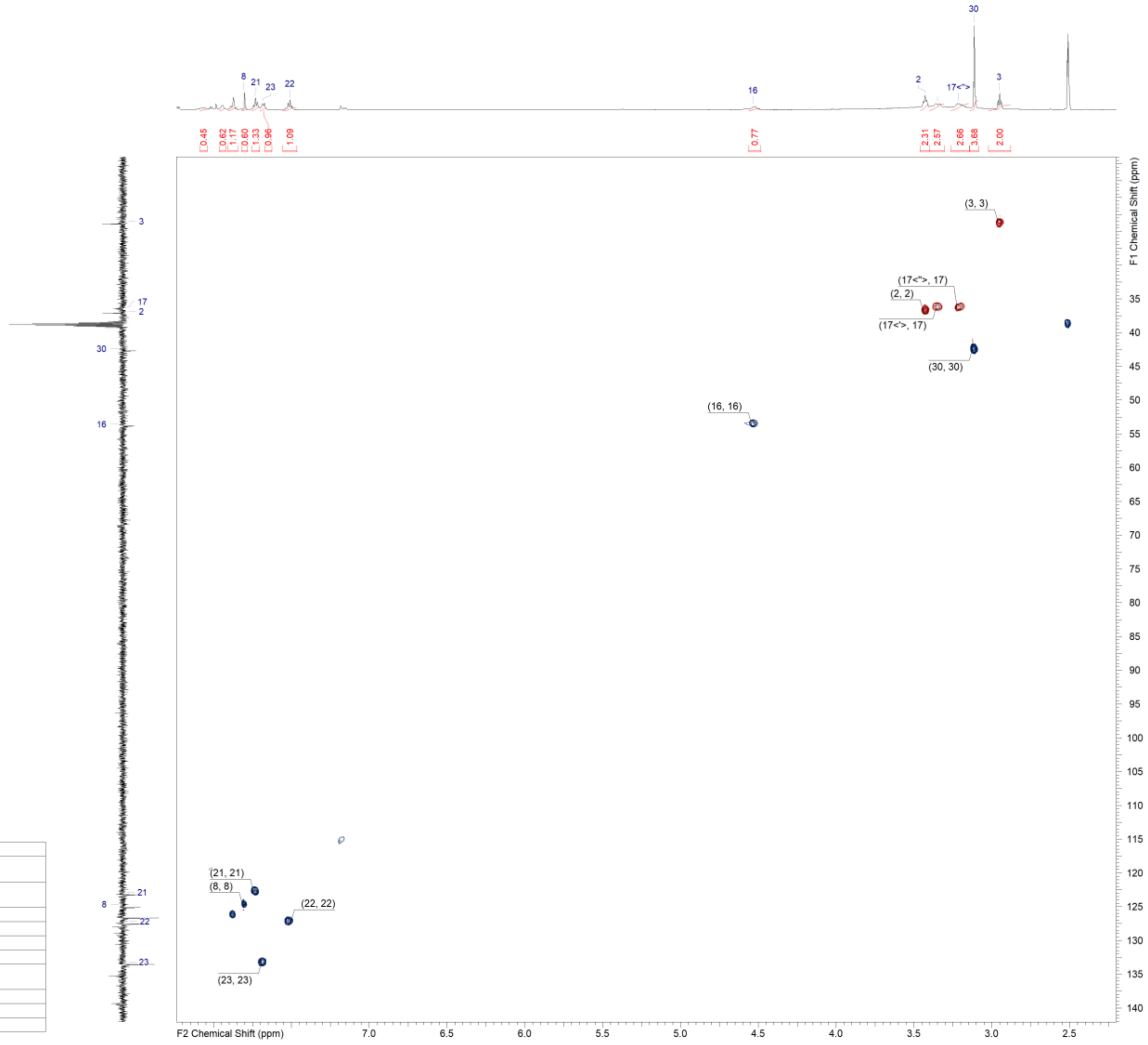

Acquisition Time (sec)	(0.1139, 0.0094)
Date	02 Aug 2023 13:40:36
Frequency (MHz)	(600.1300, 150.9029)
Nucleus	(1H, 13C)
Points Count	(1024, 1024)
Pulse Sequence	hsqcedetgp
Solvent	DMSO-d6
Sweep Width (Hz)	(8984.02, 27138.29)
Number of Transients	64
Spectrum Type	HSQC-DEPT
Temperature (degree C)	80.100

Fig. S63. Fully assigned ^{1}H-^{13}C HSQCe spectrum of DP10 in DMSO-d_6 at 80 °C

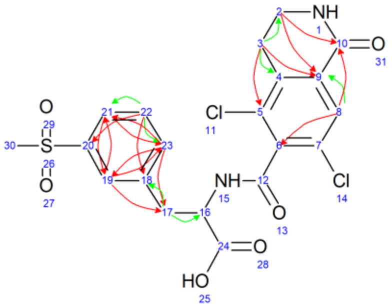

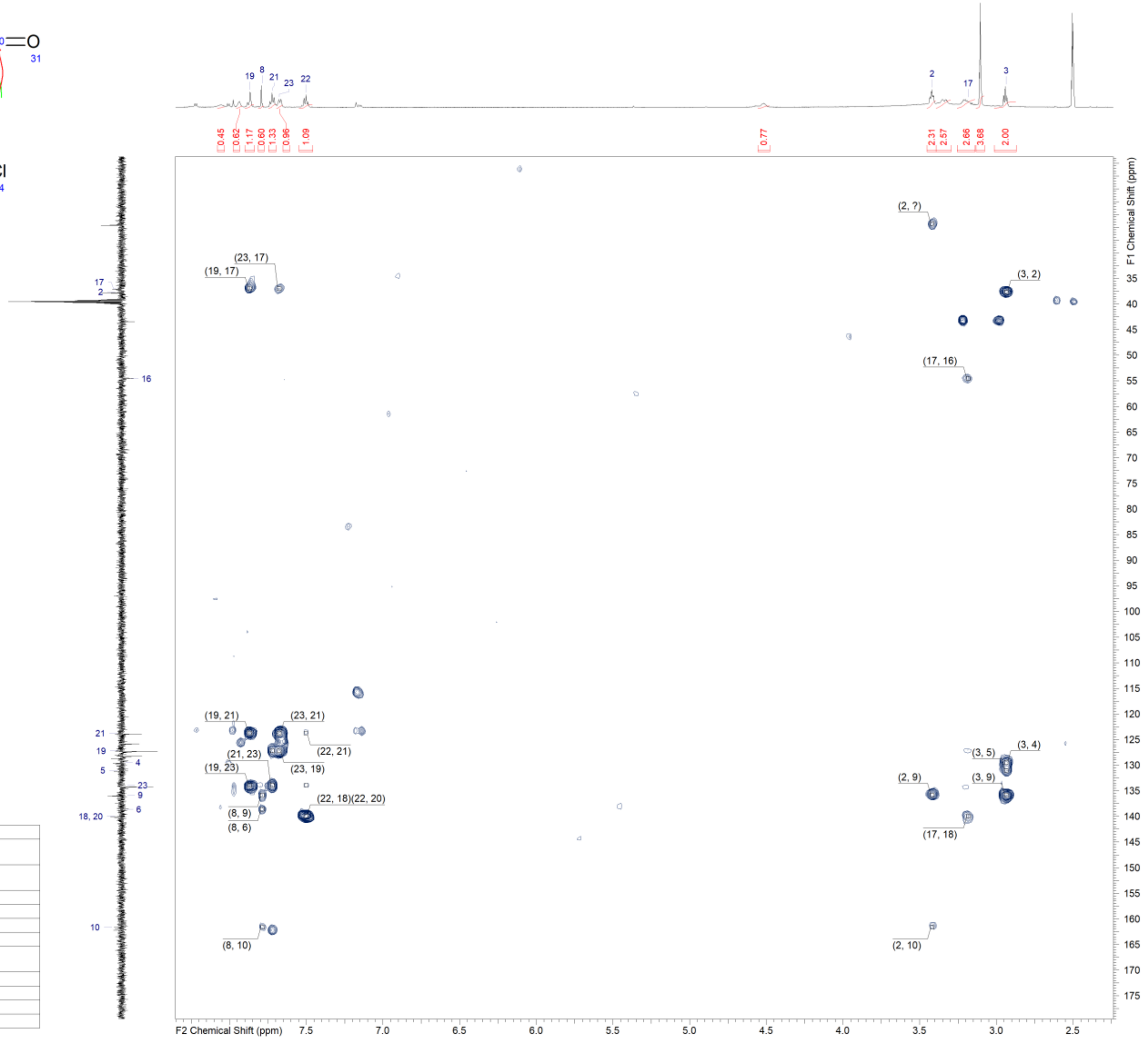

Acquisition Time (sec)	(0.1139, 0.0071)
Date	02 Aug 2023 13:40:38
Frequency (MHz)	(600.1300, 150.9029)
Nucleus	(1H, 13C)
Points Count	(2048, 1024)
Pulse Sequence	hmbcgplpndqf
Solvent	DMSO-d6
Sweep Width (Hz)	(8988.42, 36185.27)
Constant (Hz)	10.0
Number of Transients	96
Spectrum Type	HMBC
Temperature (degree C)	80.000

Fig. S64. Fully assigned ^{1}H-^{13}C HMBC spectrum of DP10 in DMSO-d_6 at 80 °C

S4.1.3. DP11 (fraction 2)

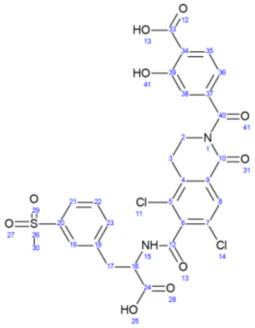

Shift1 (ppm)	H's	Type	J (Hz)	Atom1	Multiplet1
2.939	2.000	br t	6.4	3	M06
3.106	3.000	s	-	30	M01
3.173	2.000	m	-	17<">	M04
3.341	1.000	dd	13.9, 4.0	17<'>	M03
3.420	2.000	t	6.6	2	M02
4.592	1.000	m	-	16	M13
7.154	1.000	br d	7.7	36	M14
7.178	1.000	s	-	38	M15
7.511	1.000	br t	7.5	22	M16
7.670	1.000	br d	8.0	23	M18
7.725	1.000	d	7.5	21	M19
7.738	1.000	d	7.6	35	M22
7.792	1.000	m	-	8	M20
7.863	1.000	m	-	19	M21
8.197	1.000	m	-	15	M23

Shift1 (ppm)	C's	Type	Atom1	Multiplet1
24.632	1.000	s	3	M01
36.843	1.000	s	17	M02
37.838	1.000	s	2	M03
43.451	1.000	s	30	M04
54.009	1.000	s	16	M05
115.825	1.000	s	36	M07
116.550	1.000	s	38	M06
123.954	1.000	s	34	M08
124.058	1.000	s	21	M09
125.833	1.000	s	8	M10
127.322	1.000	s	19	M11
128.079	1.000	s	22	M12
128.381	1.000	s	35	M15
129.615	1.000	s	4	M13
130.929	1.000	br s	5	M14
134.162	1.000	s	23	M16
135.985	1.000	s	9	M19
138.732	1.000	s	6	M18
139.552	1.000	s	18	M20
141.287	1.000	s	20	M17
161.256	1.000	s	10	M22
162.379	1.000	s	33	M21

^{1}H NMR (600 MHz, DMSO-d_6): δ = 2.94 (br t, J = 6.4 Hz, 2 H, 3), 3.11 (s, 3 H, 30), 3.12 - 3.23 (m, 2 H, 17<">), 3.34 (dd, J = 13.9, 4.0 Hz, 1 H, 17<'>), 3.42 (t, J = 6.6 Hz, 2 H, 2), 4.52 - 4.66 (m, 1 H, 16), 7.15 (br d, J = 7.7 Hz, 1 H, 36), 7.18 (s, 1 H, 38), 7.51 (br t, J = 7.5 Hz, 1 H, 22), 7.67 (br d, J = 8.0 Hz, 1 H, 23), 7.72 (d, J = 7.5 Hz, 1 H, 21), 7.74 (d, J = 7.6 Hz, 1 H, 35), 7.77 - 7.80 (m, 1 H, 8), 7.85 - 7.88 (m, 1 H, 19), 8.13 - 8.26 (m, 1 H, 15) ppm

^{13}C NMR (151 MHz, DMSO-d_6): δ = 24.6 (3), 36.8 (17), 37.8 (2), 43.5 (30), 54.0 (16), 115.8 (36), 116.5 (38), 124.0 (34), 124.1 (21), 125.8 (8), 127.3 (19), 128.1 (22), 128.4 (35), 129.6 (4), 130.9 (5), 134.2 (23), 136.0 (9), 138.7 (6), 139.6 (18), 141.3 (20), 161.3 (10), 162.4 (33) ppm

Solvent	**DMSO-d6**
Pulse Sequence	**zg30**
Temperature (degree C)	**80.400**
Acquisition Time (sec)	**1.3631**
Number of Transients	**256**
Points Count	**32768**
Sweep Width (Hz)	**12018.86**
Spectrum Offset (Hz)	**5401.1729**
Original Points Count	**16384**
Nucleus	**1H**
Frequency (MHz)	**600.1300**

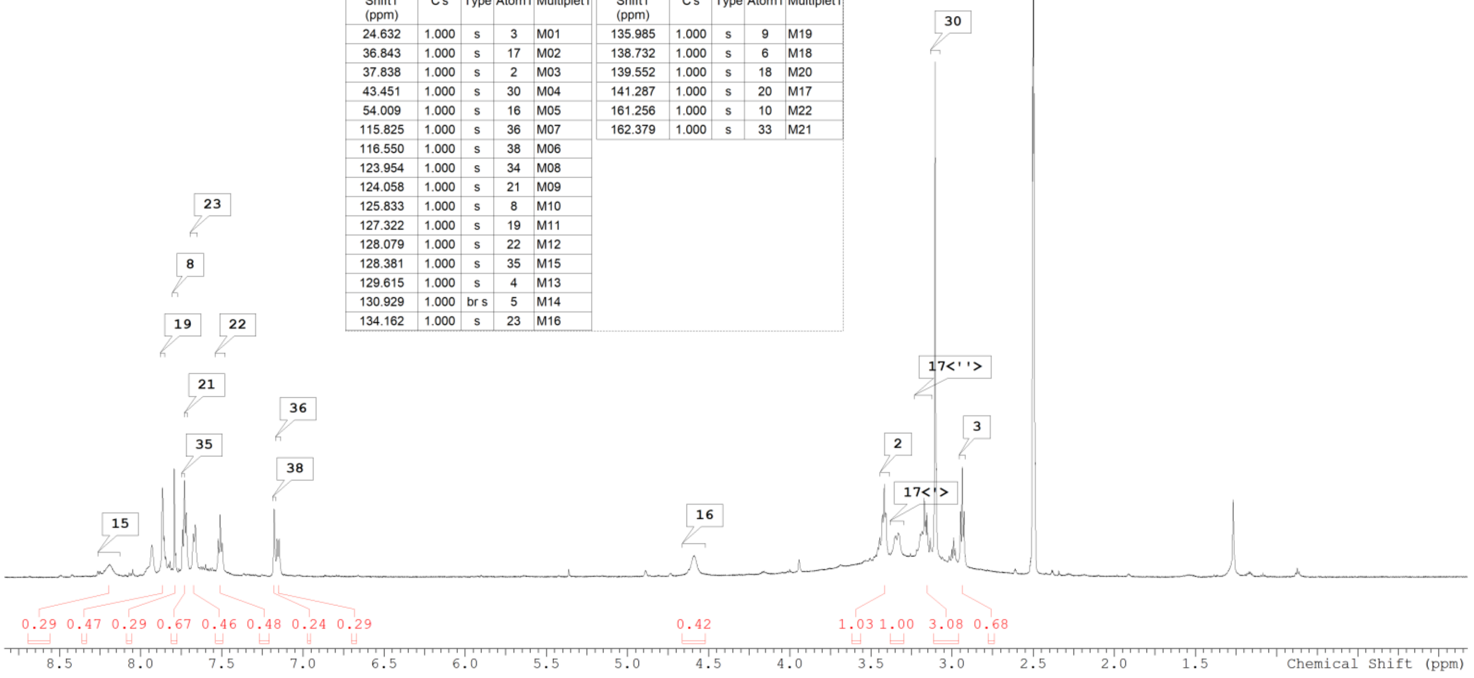

Fig. S65. Fully assigned ^{1}H spectrum and ^{13}C chemical shifts (extracted from ^{1}H-^{13}C HMBC) of DP11 in DMSO-d_6 at 80 °C

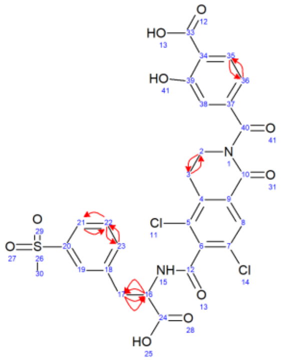

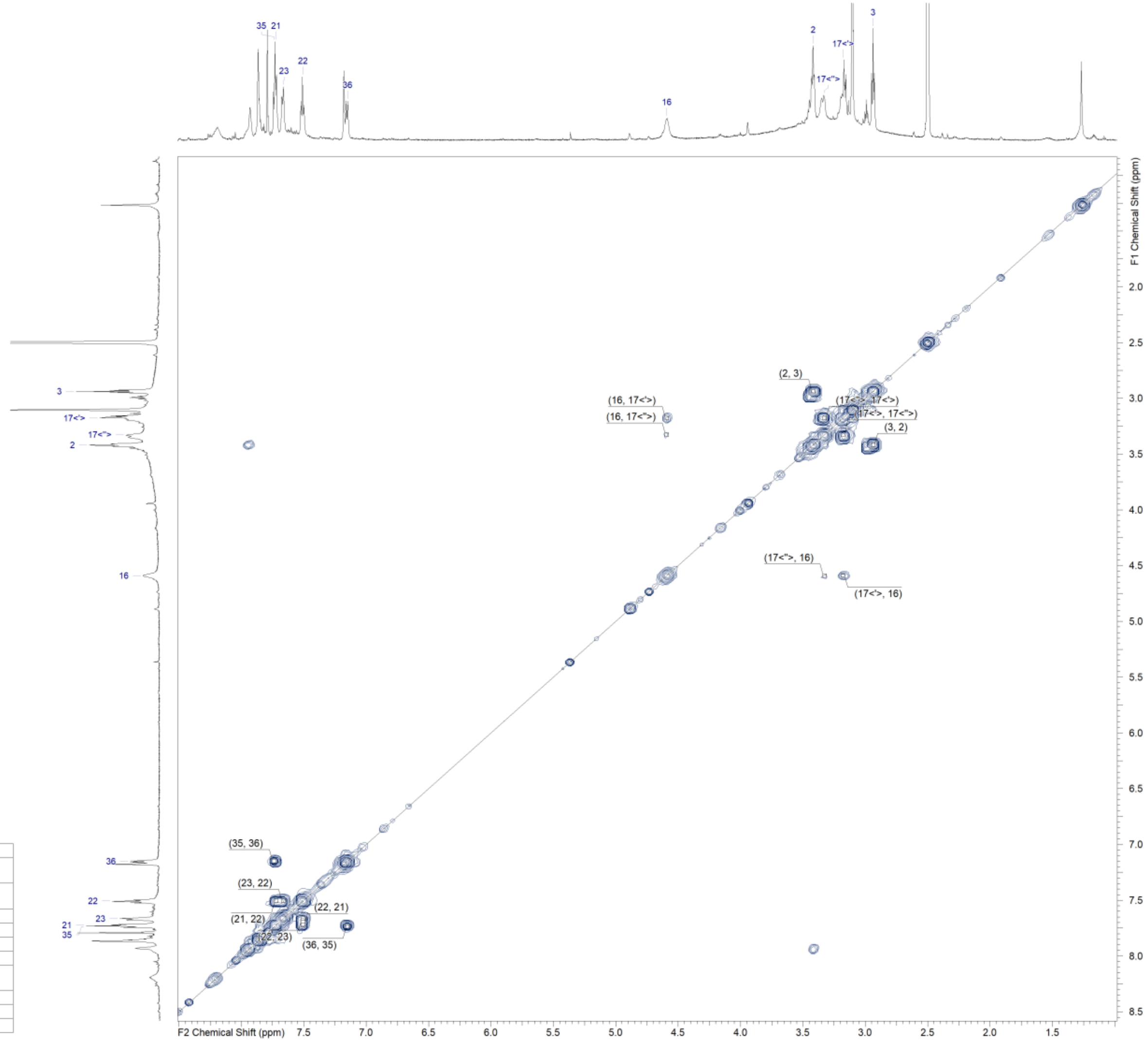

Acquisition Time (sec)	(0.1065, 0.0267)
Date	31 Jul 2023 17:46:30
Frequency (MHz)	(600.1300, 600.1300)
Nucleus	(1H, 1H)
Points Count	(2048, 2048)
Pulse Sequence	cosygpqf
Solvent	DMSO-d6
Sweep Width (Hz)	(9610.69, 9597.45)
Number of Transients	4
Spectrum Type	COSY
Temperature (degree C)	80.300

Fig. S66. Fully assigned ^1H-^1H COSY spectrum of DP11 in DMSO-d_6 at 80 °C

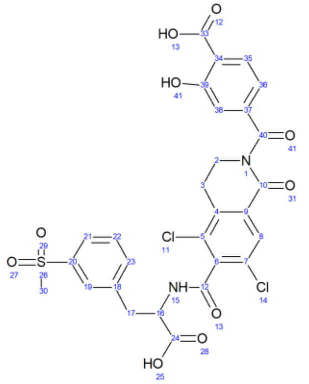

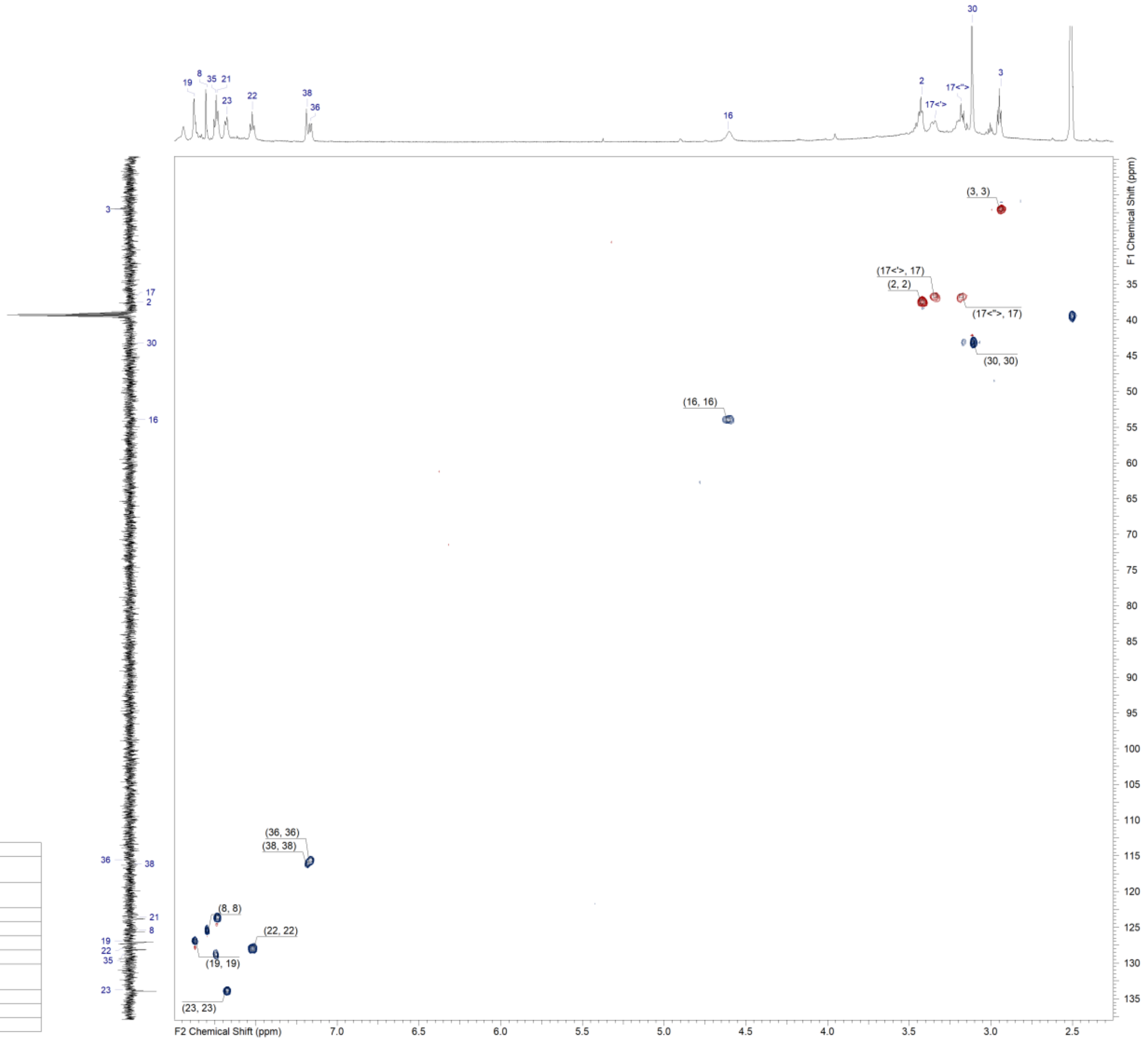

Acquisition Time (sec)	(0.1139, 0.0094)
Date	31 Jul 2023 22:58:44
Frequency (MHz)	(600.1300, 150.9029)
Nucleus	(1H, 13C)
Points Count	(1024, 1024)
Pulse Sequence	hsqcedetgp
Solvent	DMSO-d6
Sweep Width (Hz)	(8984.02, 27138.29)
Number of Transients	64
Spectrum Type	HSQC-DEPT
Temperature (degree C)	80.200

Fig. S67. Fully assigned ^{1}H-^{13}C HSQCe spectrum of DP11 in DMSO-d_6 at 80 °C

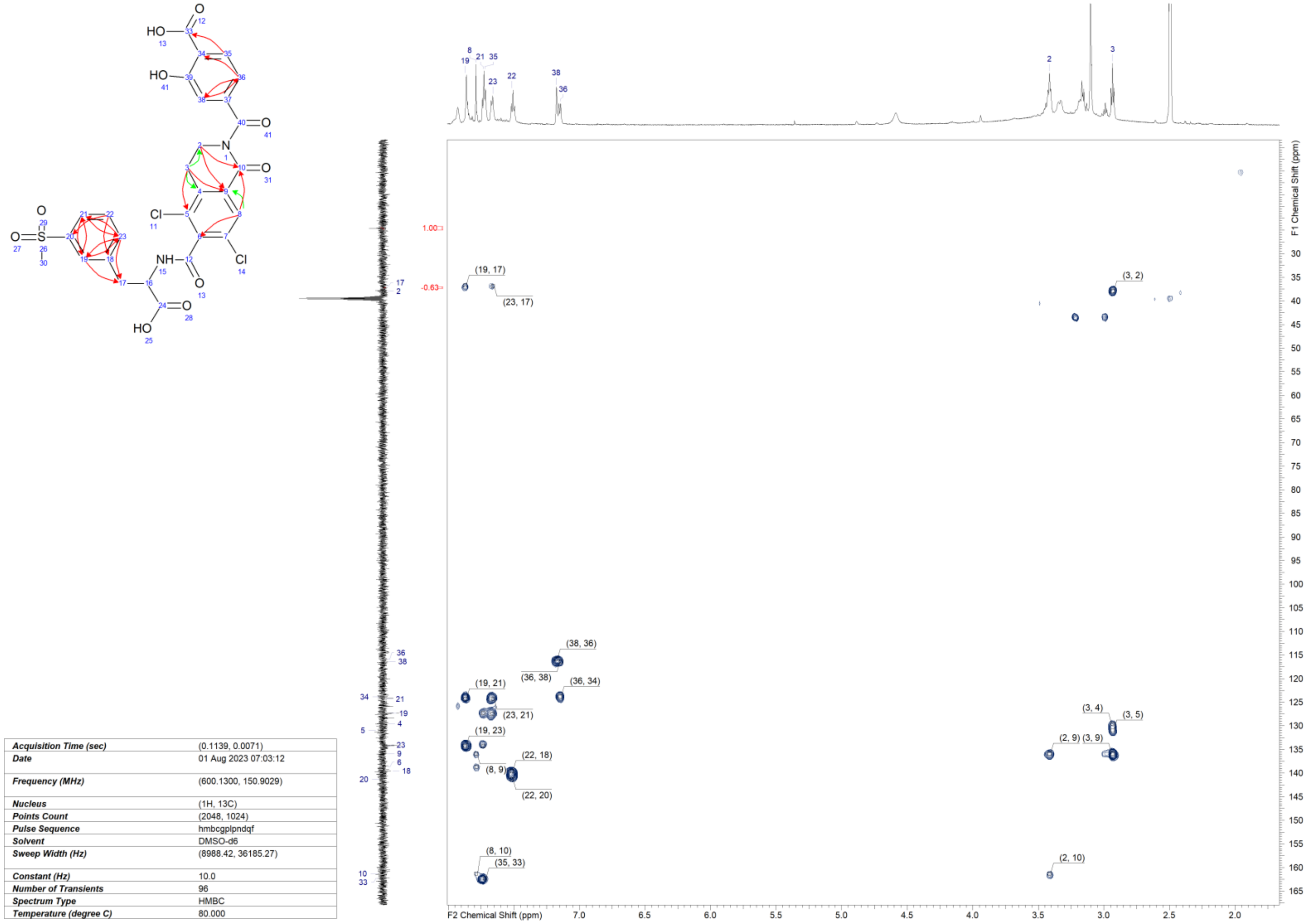

**Acquisition Time (sec)**	(0.1139, 0.0071)
**Date**	01 Aug 2023 07:03:12
**Frequency (MHz)**	(600.1300, 150.9029)
**Nucleus**	(1H, 13C)
**Points Count**	(2048, 1024)
**Pulse Sequence**	hmbcgplpndqf
**Solvent**	DMSO-d6
**Sweep Width (Hz)**	(8988.42, 36185.27)
**Constant (Hz)**	10.0
**Number of Transients**	96
**Spectrum Type**	HMBC
**Temperature (degree C)**	80.000

Fig. S68. Fully assigned ^{1}H-^{13}C HMBC spectrum of DP11 in DMSO-d_6 at 80 °C

S4.2. LC-UV/MS analysis

Table S7. Experimental parameters for HPLC-MS method for extreme degradation study

<table>
<tr><td colspan="4">LC parameters</td></tr>
<tr><td>HPLC system</td><td colspan="3">Shimadzu LC-MS 2020</td></tr>
<tr><td>Column</td><td colspan="3">Zorbax XDB C18 column (4.6 × 500 mm, 3.5 µm, Agilent Technologies, USA)</td></tr>
<tr><td>Mobile phase</td><td colspan="3">Mobile phase A: H20 + 0.1% FA
Mobile phase B: MeOH</td></tr>
<tr><td>Flow rate</td><td colspan="3">0.5 ml/min</td></tr>
<tr><td rowspan="5">Gradient program</td><td>Time (min)</td><td>%A</td><td>%B</td></tr>
<tr><td>0.0</td><td>90</td><td>10</td></tr>
<tr><td>12.0</td><td>10</td><td>90</td></tr>
<tr><td>17.0</td><td>10</td><td>90</td></tr>
<tr><td>20.0</td><td>90</td><td>10</td></tr>
<tr><td>Analysis time</td><td colspan="3">20 min</td></tr>
<tr><td>Injection volume</td><td colspan="3">10 µl</td></tr>
<tr><td>Column temperature</td><td colspan="3">25 °C</td></tr>
<tr><td colspan="4">MS parameters</td></tr>
<tr><td>MS system</td><td colspan="3">LC-MS 2020 SQ</td></tr>
<tr><td>Heatblock</td><td colspan="3">200 °C</td></tr>
<tr><td>Drying gas flow</td><td colspan="3">10 l/ min</td></tr>
<tr><td>Nebulizing gas flow</td><td colspan="3">1.5 l/ min</td></tr>
<tr><td>DL temperature</td><td colspan="3">250 °C</td></tr>
<tr><td>Interface</td><td colspan="3">350 °C</td></tr>
<tr><td>Interface voltage</td><td colspan="3">4500 V</td></tr>
<tr><td>MS range</td><td colspan="3">m/z 100–1000</td></tr>
</table>

S4.2.1. Fraction 1 (DP9)

(A)

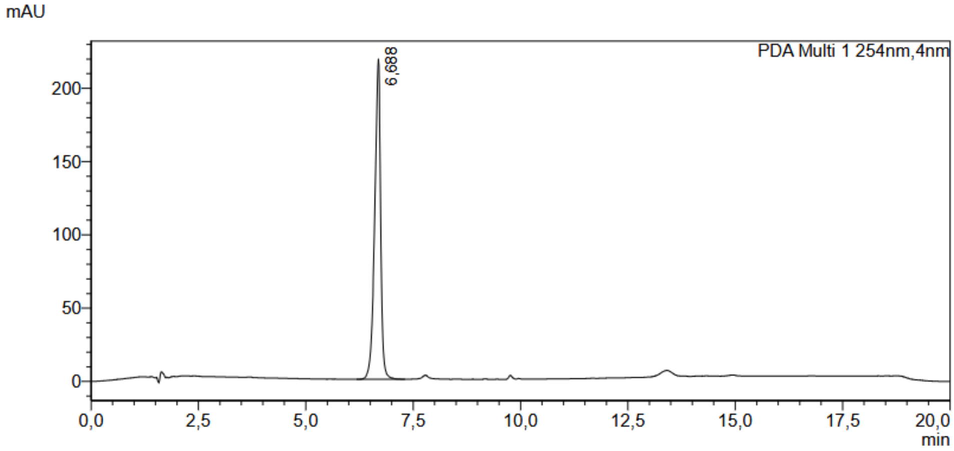

Peak Table

PDA Ch1 254nm

Peak#	Ret. Time	Area	Height	Conc.	Unit	Mark	Name
1	6,688	2112459	218354	100,000			
Total		2112459	218354				

(B)

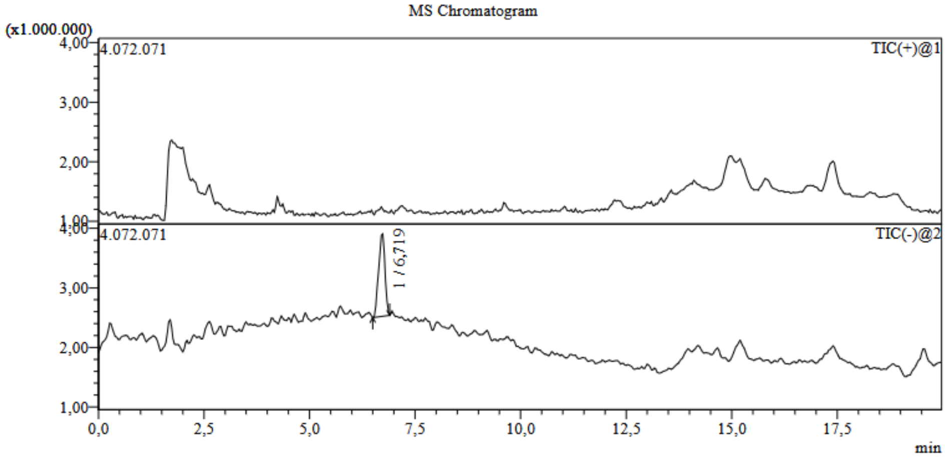

MASS Peak Table TIC

Peak#	Ret. Time	m/z	Area	Mark	Compound Name	A/H	Event#
1	6,719	TIC	14546544			10,463	1-2
Total			14546544				

(C)

MS Spectrum

Line#:1 R.Time:----(Scan#:----)
MassPeaks:4
Spectrum Mode:Averaged 6,682-6,749(402-406) Base Peak:181(939471)
BG Mode:Calc Segment 1 - Event 2

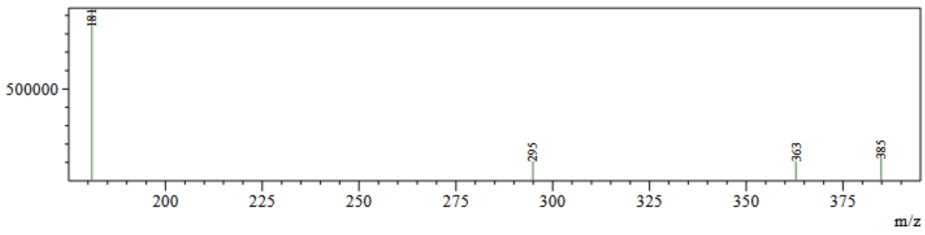

Fig. S69. (A) LC-UV, (B) LC-MS chromatograms and (C) MS spectrum of fraction 1

S4.2.2. Fraction 2 (DP11)

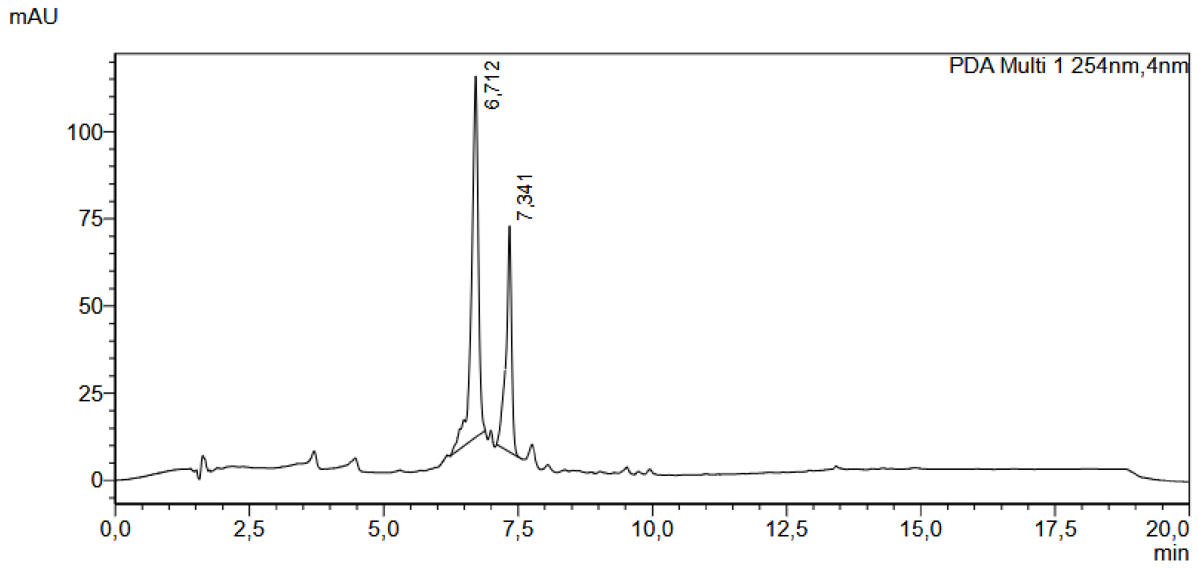

Peak Table

PDA Ch1 254nm

Peak#	Ret. Time	Area	Height	Conc.	Unit	Mark	Name
1	6,712	908775	103503	66,313		M	
2	7,341	461666	64808	33,687		M	
Total		1370441	168311				

Fig. S70. LC-UV chromatograms of fraction 2

S4.2.3. Fraction 3 (DP10 and DP11)

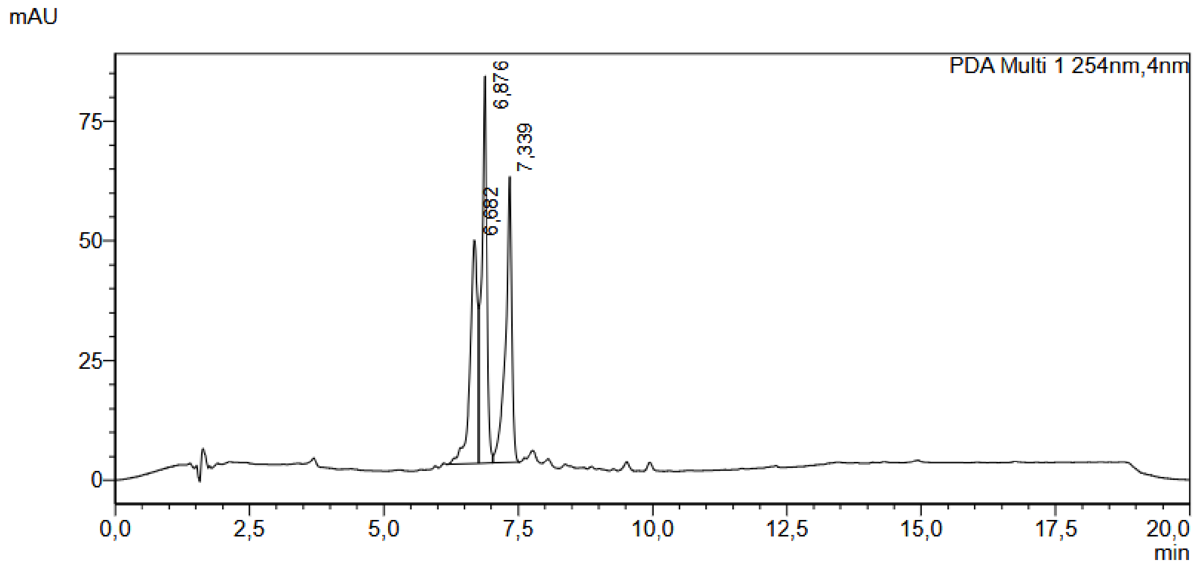

Peak Table

PDA Ch1 254nm

Peak#	Ret. Time	Area	Height	Conc.	Unit	Mark	Name
1	6,682	464337	46567	30,956		M	
2	6,876	555777	80836	37,052		V M	
3	7,339	479891	59712	31,993		V M	
Total		1500006	187114				

Fig. S71. LC-UV chromatograms of fraction 3

S5. Targeted oxidative degradation

S5.1. MS analysis

Targeted oxidative degradation of 50 mg/ml Lifitegrast in aqueous solution was conducted with 3% H_2O_2 in duration of 7 days. The structures of three isolated degradation products were determined using UHPLC-HRMS. Detailed experimental parameters for isolation (analytical and preparative HPLC), as well as MS/MS and HRMS methods for structure elucidation are provided below.

Table S8. Experimental parameters for degradation monitoring - analytical HPLC method

Instrument	1260 Infinity II SFC/UHPLC Hybrid System (Agilent Technologies)		
Column	ACQUITY UPLC HSS T3, 1,8 µm, 150 mm x 4,6 mm i.d.		
Mobile phase A	20 mM ammonium formate/acetonitrile = 99/1 (v/v%)		
Mobile phase B	20 mM ammonium formate/acetonitrile = 20/80 (v/v%)		
Gradient program	time/min	% MF A	% MF B
	0	100	0
	3	100	0
	15	50	50
	16	50	50
	22	0	100
	27	0	100
	28	100	0
	35	100	0
Run time	32 min		
Flow rate	0.4 ml/min		
Detector	UV at 210 nm (Bandwith = 4), DAD 190 – 490 nm		
Column temperature	40 ˚C		
Injection volume	3 µl		
Diluent	H_2O:acetonitrile = 99:1		

Table S9. Experimental parameters for degradation products isolation - analytical HPLC method

Instrument	1260 Infinity II SFC/UHPLC Hybrid System (Agilent Technologies)
Column	Zorbax Extend-C18, Agilent Technologies, 5 µm, 150 mm x 4,6 mm i.d.
Mobile phase A	20 mM ammonium formate/acetonitrile = 99/1 (v/v%)
Mobile phase B	20 mM ammonium formate/acetonitrile = 20/80 (v/v%)

Gradient program	time/min	% MF A	% MF B
	0	100	0
	4	100	0
	23	50	50
	25	50	50
	27	0	100
	30	0	100
	30.01	100	0
	32	100	0
Run time	32 min		
Flow rate	1.0 ml/min		
Detector	UV at 210 nm (Bandwith = 4), DAD 190 – 490 nm		
Column temperature	40 ˚C		
Injection volume	10 µl		
Diluent	H_2O:acetonitrile = 99:1		

50 mg/ml Lifitegrast solution was diluted with a water/acetonitrile mixture (99:1, v/v; 2 ml) and filtered through a 0.45 µm RC filter. The solution was purified using preparative HPLC on an Extend-C18 column (Agilent Technologies, 5 µm, 150 mm × 21.2 mm i.d.).

Table S10. Experimental parameters for degradation products isolation - preparative HPLC method

Instrument	Agilent 1260 Infinity II with UV detector		
Column	Extend-C18, Agilent Technologies, 5 µm, 150 mm x 21.2 mm i.d.		
Mobile phase A	20 mM ammonium formate/acetonitrile = 99/1 (v/v%)		
Mobile phase B	20 mM ammonium formate/acetonitrile = 20/80 (v/v%)		
Gradient program	time/min	% MF A	% MF B
	0	100	0
	4.06	100	0
	23.45	50	50
	25.47	50	50
	27.53	0	100
	30.59	0	100
	30.60	100	0
	32.62	100	0
Run time	32.62 min		
Flow rate	12.50 ml/min		
Detector	UV at 210 nm		
Column temperature	RT		
Injection volume	500 µl		
Diluent	H_2O:acetonitrile = 99:1		

Table S11. Experimental parameters for UHPLC-HRMS method used for structure elucidation of degradation products

**LC parameters**	
UHPLC system	Agilent 1290 Infinity II UHPLC
Column	Acquity HSS T3 (1.8 µm, 2.1×100 mm)
Mobile phase	Mobile phase A: 20 mM ammonium formate (pH 4.0) /ACN (99:1)<br>Mobile phase B: 20 mM ammonium formate (pH 4.0) /ACN (20:80)
Flow rate	0.4 ml/min
Gradient program	see below
Analysis time	35 min
Injection volume	3 µl
Column temperature	40 °C
**MS parameters**	
MS system	Agilent 6550 Series Accurate-Mass-Quadrupole Time-of-flight
Drying gas temp.	200 °C
Drying gas flow	14 l/ min
Nebulizer	35 psi
Sheath gas temp.	200 °C
Sheath gas flow	11 l/min
Capillary voltage	3000 V
Nozzle voltage	1000 V
Fragmentor	380 V
MS range	*m/z* 100–1000
MS/MS range	*m/z* 50–700
Collision energies	10 V, 20 V, 30 V

Gradient program:

Time (min)	%A	%B
0.0	100	0
3.0	100	0
15.0	50	50
16.0	50	50
22.0	0	100
27.0	0	100
28.0	100	0
35.0	100	0

S5.1.1. DP5

The chromatographic peak observed at retention time 11.338 min corresponds to DP5 (ESI+ *m/z* 635.0691, ESI– *m/z* 633.0525). Ions at *m/z* 392 and *m/z* 165 support the presence of a hydroxybenzoic functional group. Ions at *m/z* 453 and *m/z* 425 in ESI+ (along with ions at *m/z* 225, *m/z* 169, and *m/z* 181 in ESI-) were also observed in the MS/MS spectra of Lifitegrast, indicating that both compounds share the same molecular core.

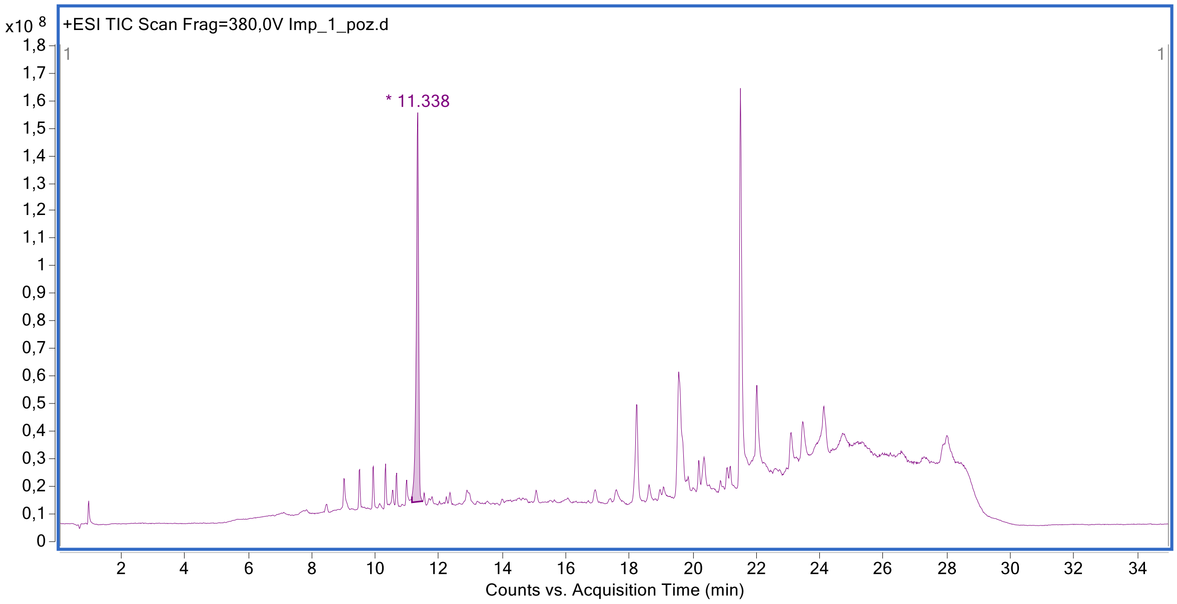

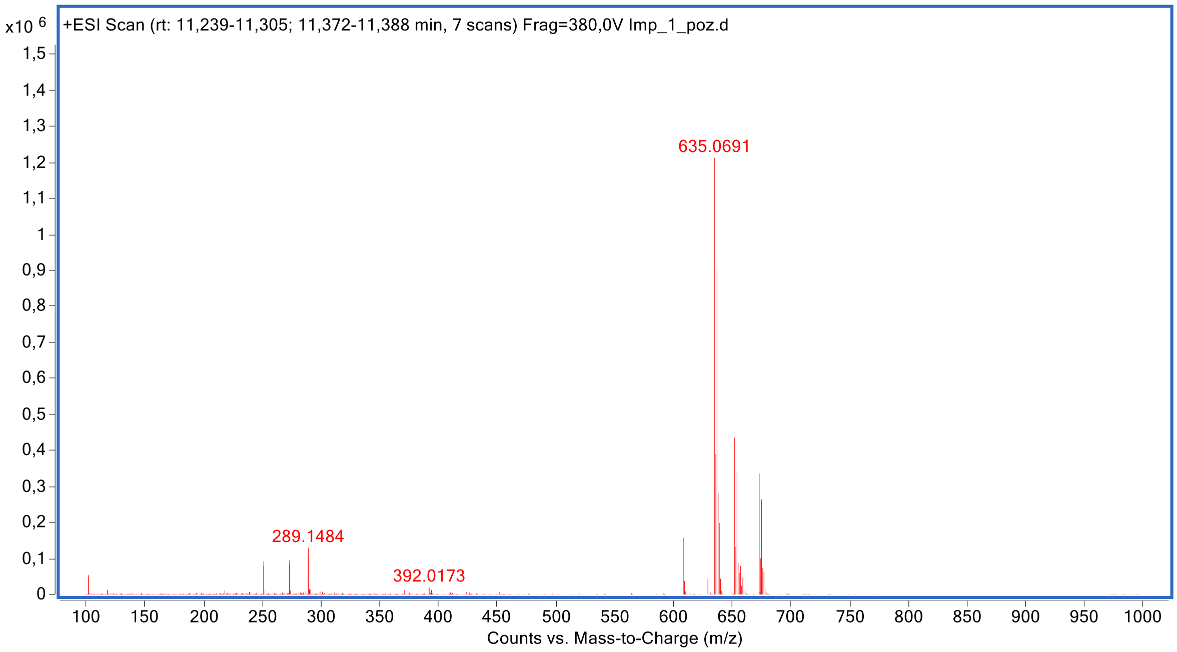

Fig. S72. ESI+ total ion chromatogram of DP5 and MS spectrum of chromatographic peak at retention time 11.338 min

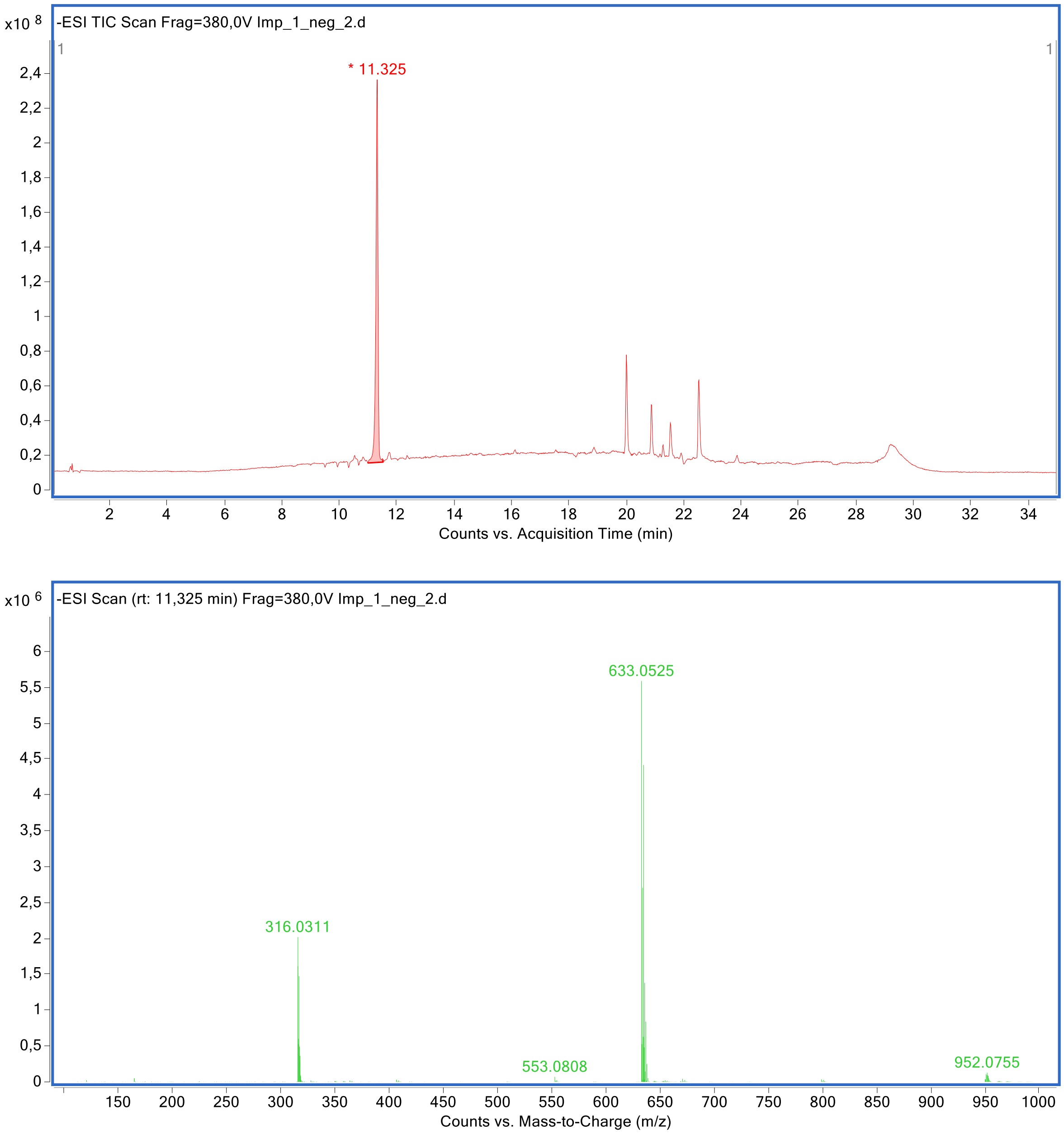

Fig. S73. ESI– total ion chromatogram of DP5 and MS spectrum of chromatographic peak at retention time 11.325 min

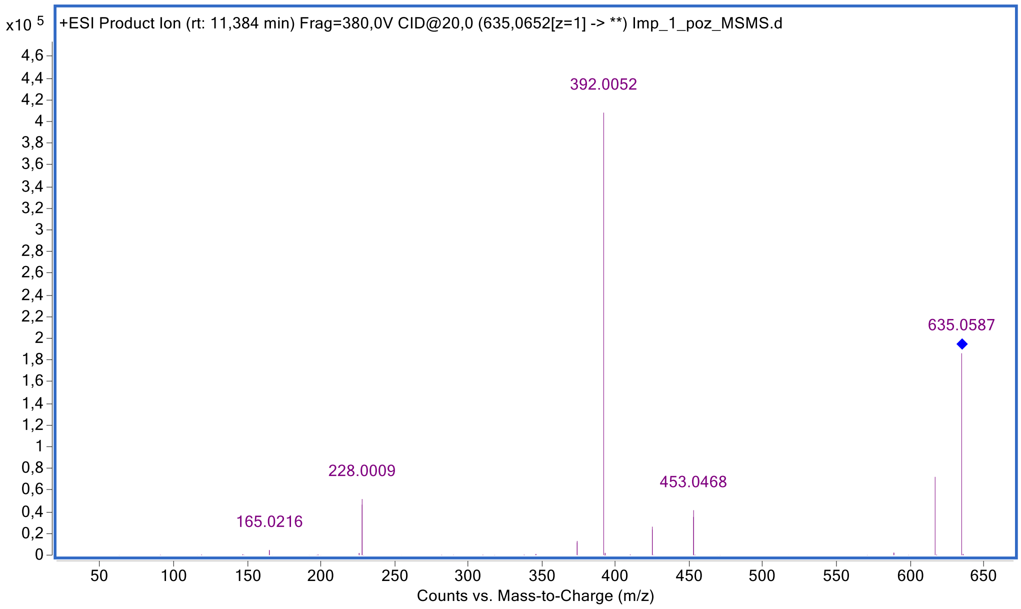

Fig. S74. ESI+ MS/MS spectrum of ion at m/z 635.0652, CE 20 V

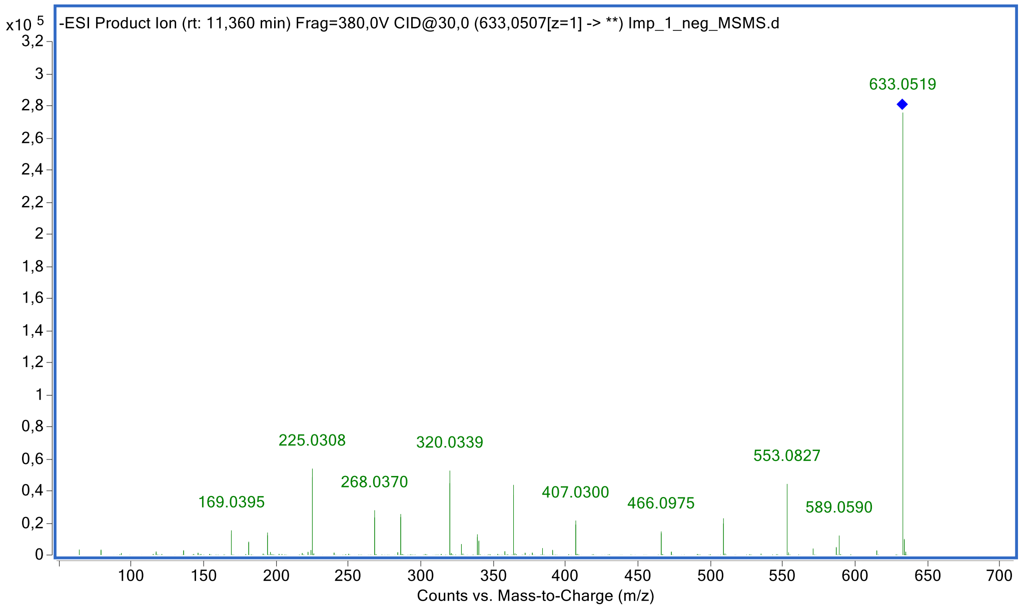

Fig. S75. ESI– MS/MS spectrum of ion at m/z 633.0507, CE 30 V

S5.1.2. DP12

The chromatographic peak observed at retention time 13.739 min corresponds to the DP12 (ESI+ *m/z* 631.0711, ESI– *m/z* 629.0620).

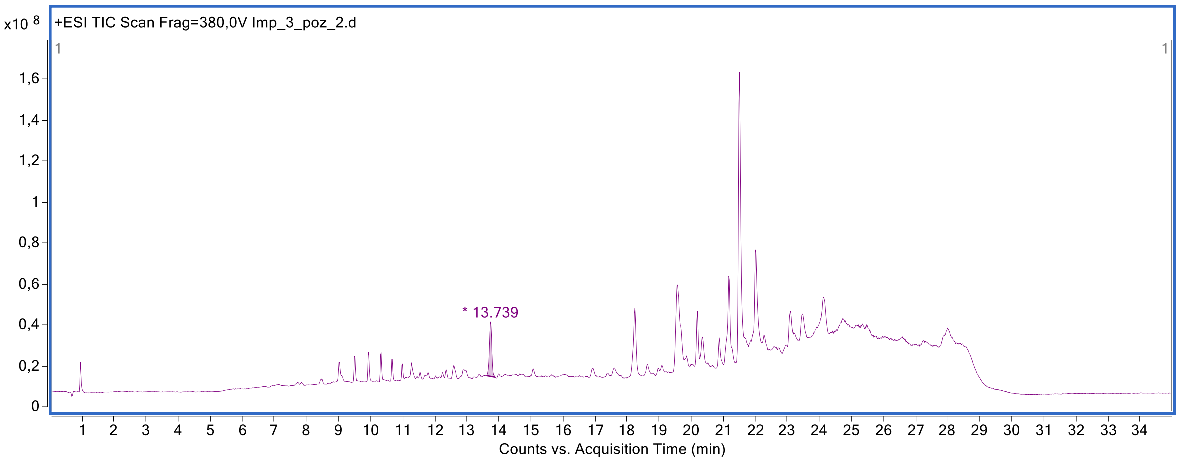

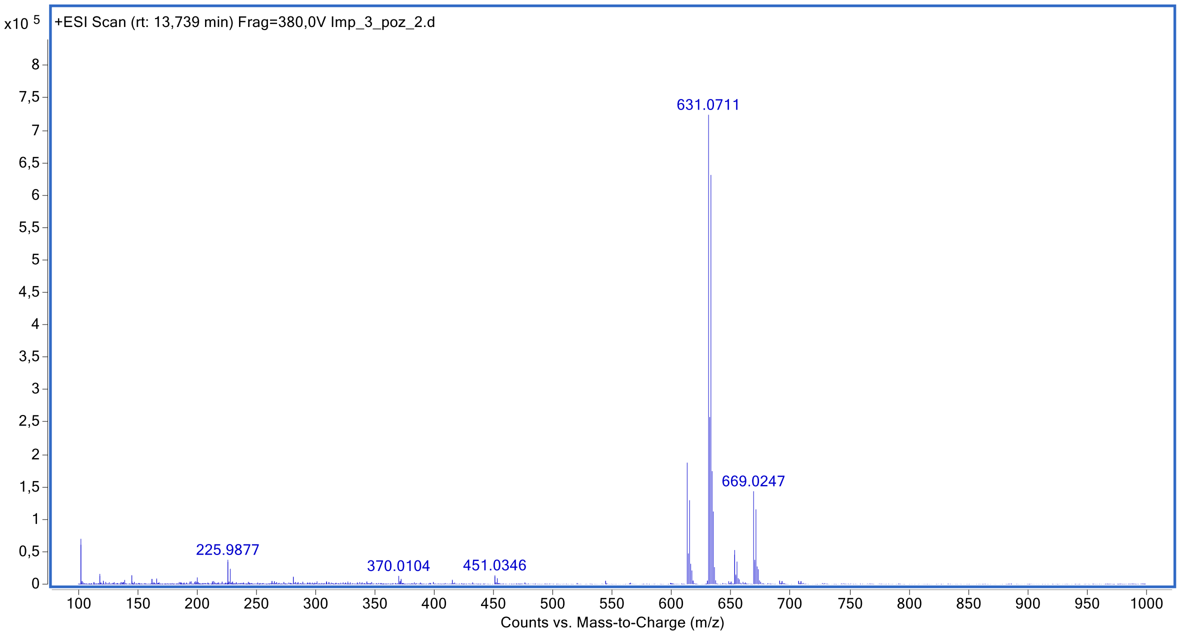

Fig. S76. ESI+ total ion chromatogram of DP12 and MS spectrum of chromatographic peak at retention time 13.739 min

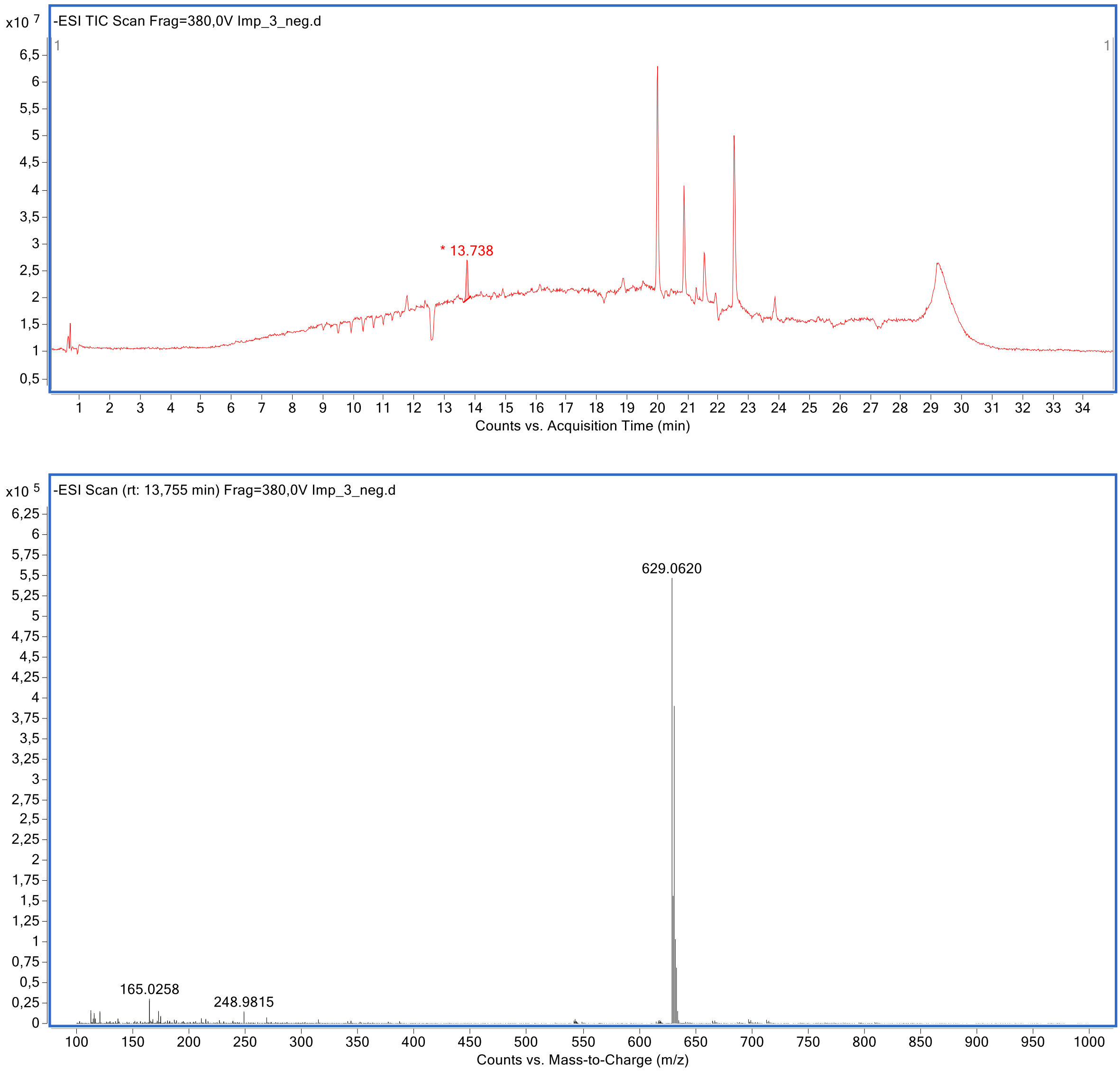

Fig. S77. ESI– total ion chromatogram of DP12 and MS spectrum of chromatographic peak at retention time 13.738 min

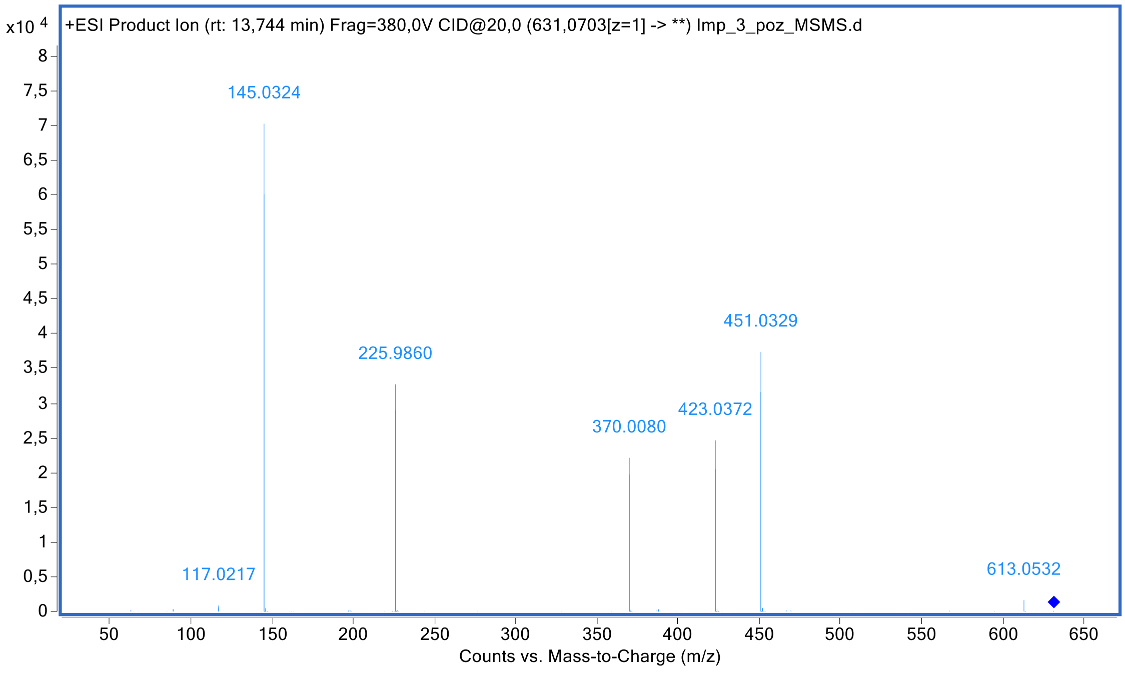

Fig. S78. ESI+ MS/MS spectrum of DP12, CE 20 V

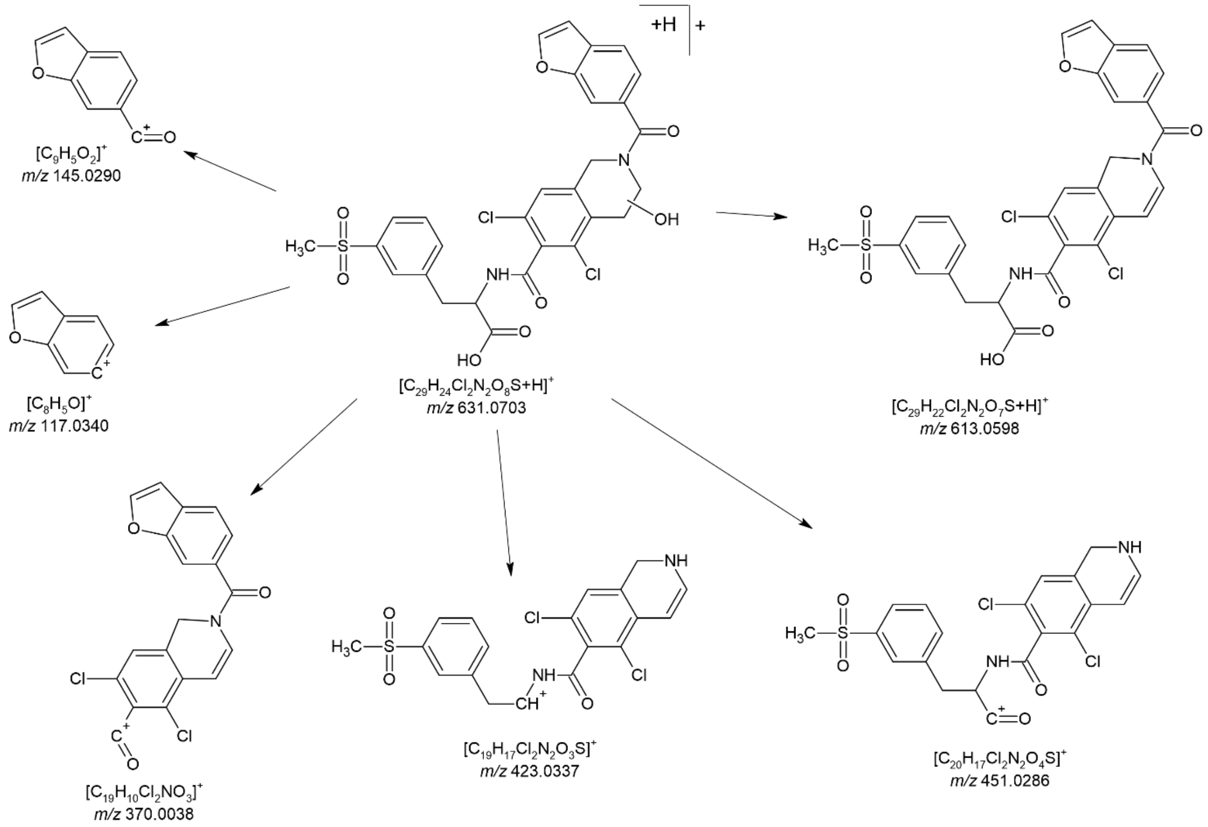

Fig. S79. Possible fragmentation pathway of DP12, ESI+

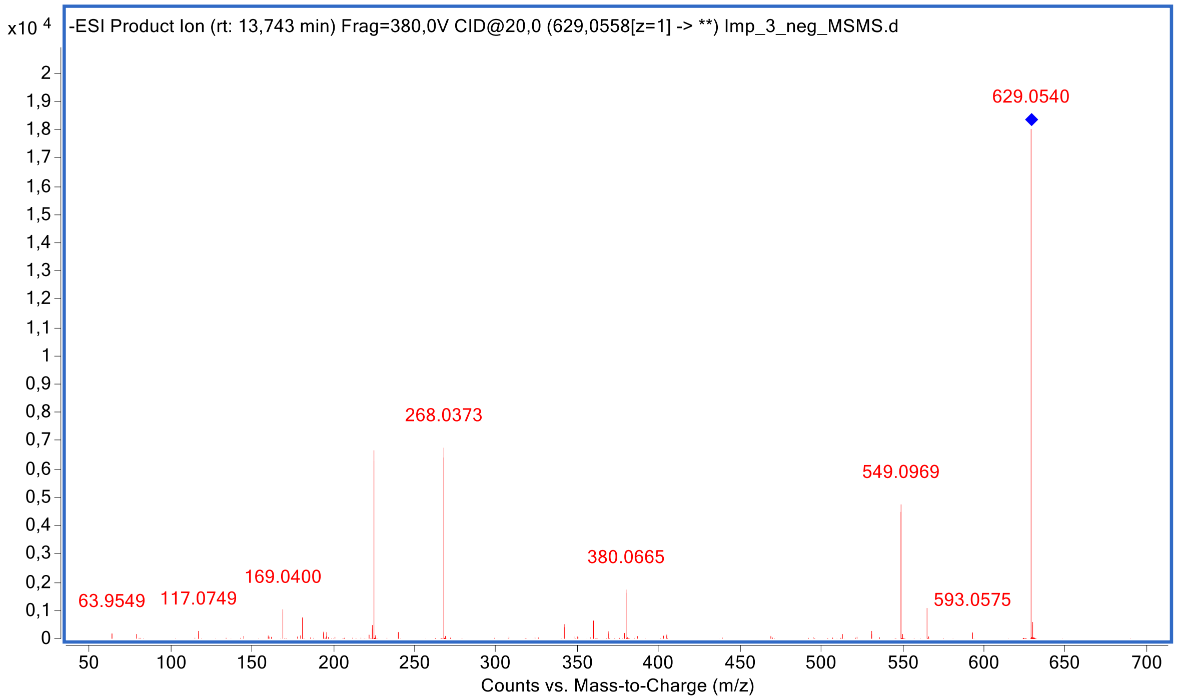

Fig. S80. ESI– MS/MS spectrum of DP12, CE 20 V

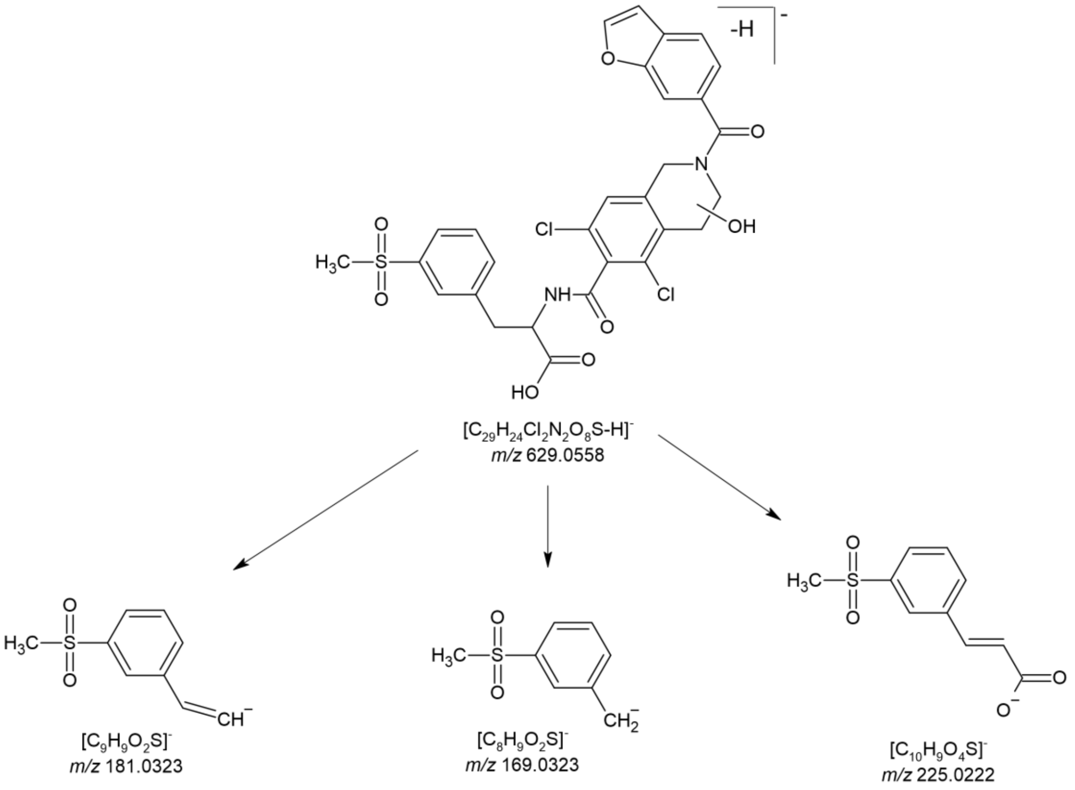

Fig. S81. Possible fragmentation pathway of DP12, ESI-

S5.1.3. DP7

The chromatographic peak observed at retention time 14.346 min corresponds to DP7 (ESI+ *m/z* 631.0704, ESI– *m/z* 629.0657)

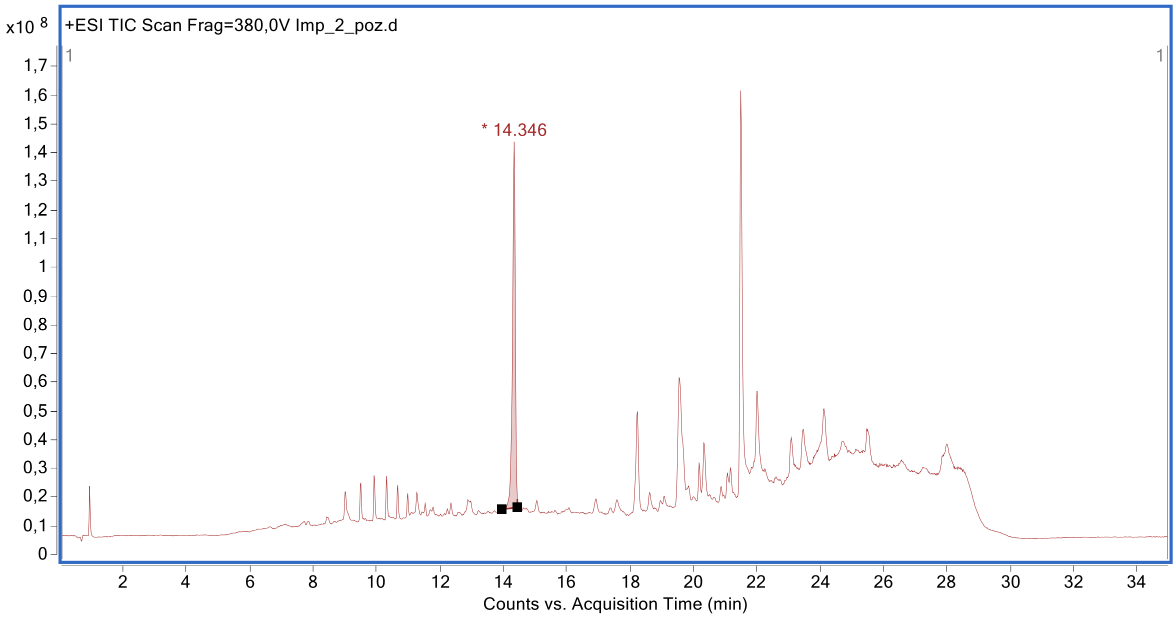

Fig. S82. ESI+ total ion chromatogram of DP7

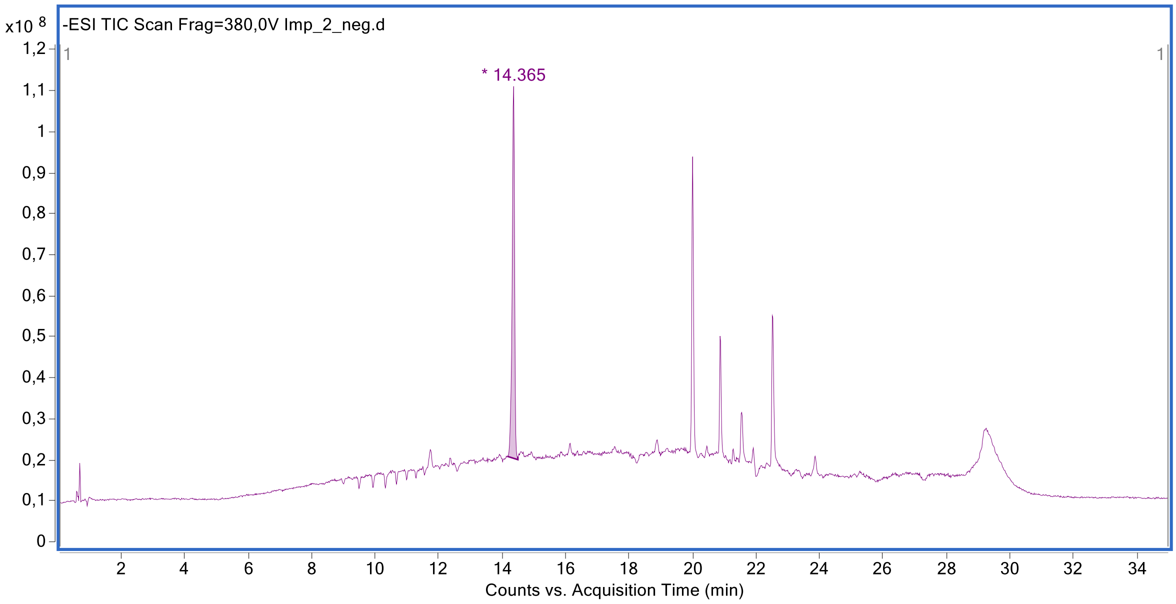

Fig. S83. ESI– total ion chromatogram of DP7 and MS spectrum of chromatographic peak at retention time 14.365 min

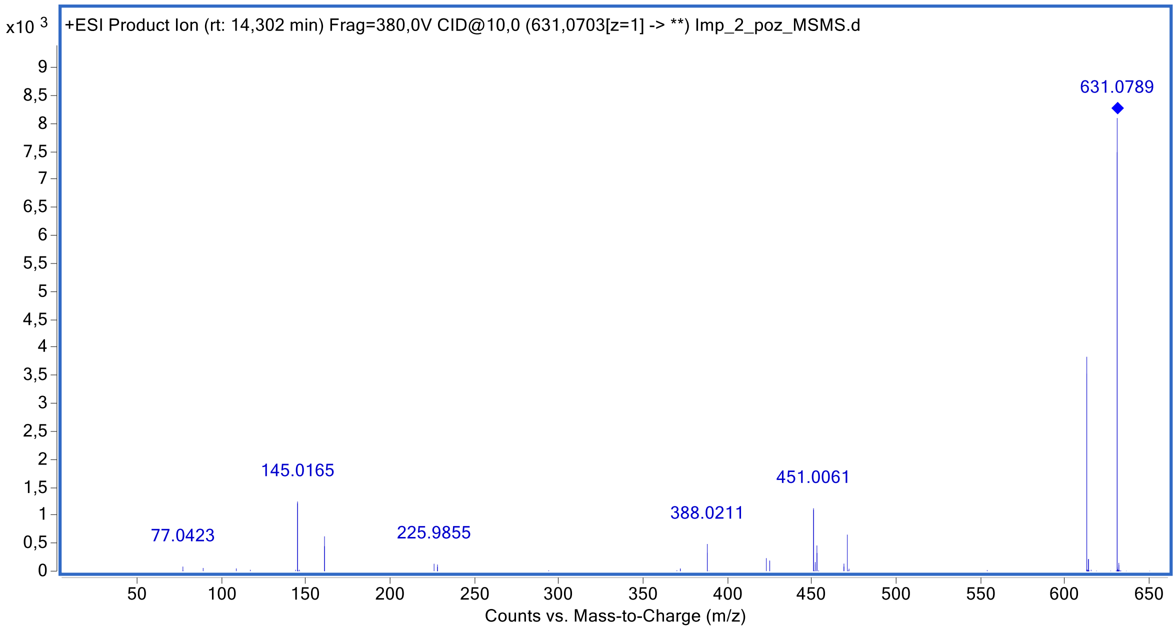

Fig. S84. ESI+ MS/MS spectrum of ion at m/z 631.0703, CE 10 V

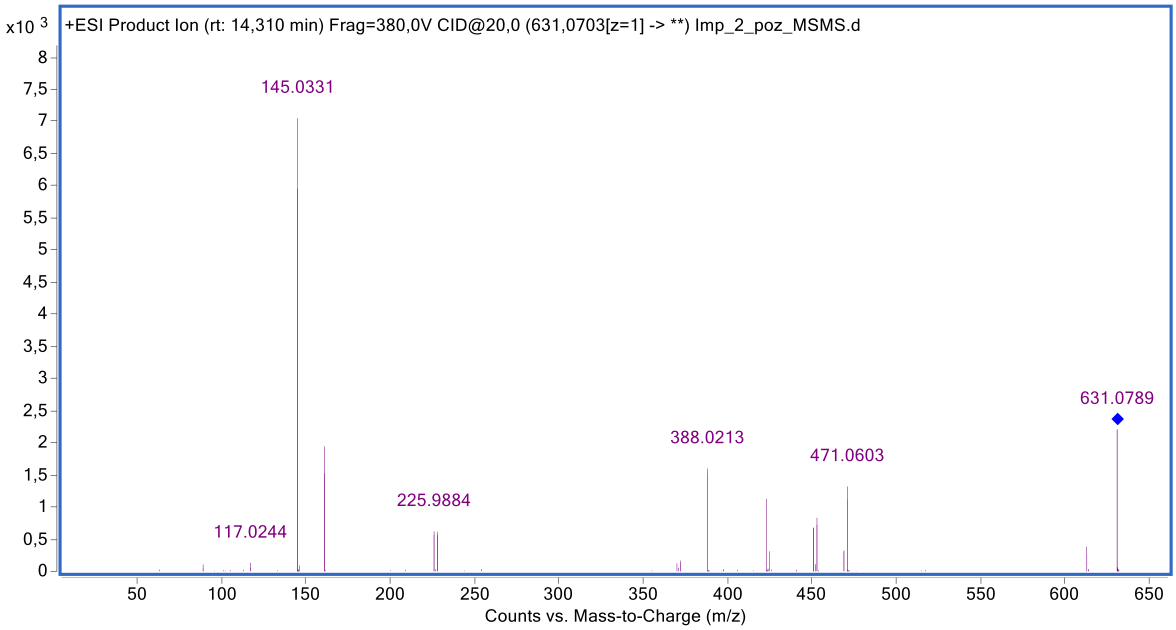

Fig. S85. ESI+ MS/MS spectrum of ion at m/z 631.0703, CE 20 V

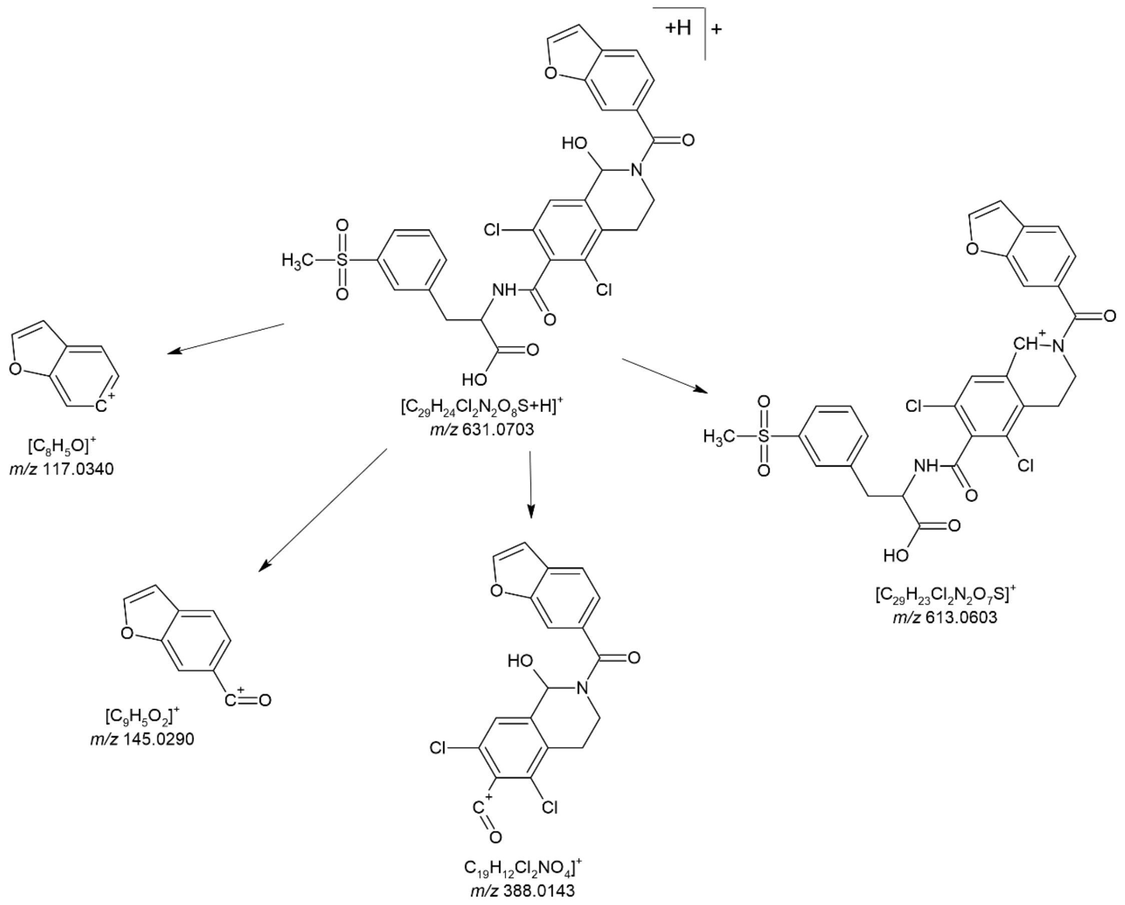

Fig. S86. Possible fragmentation pathway of DP7

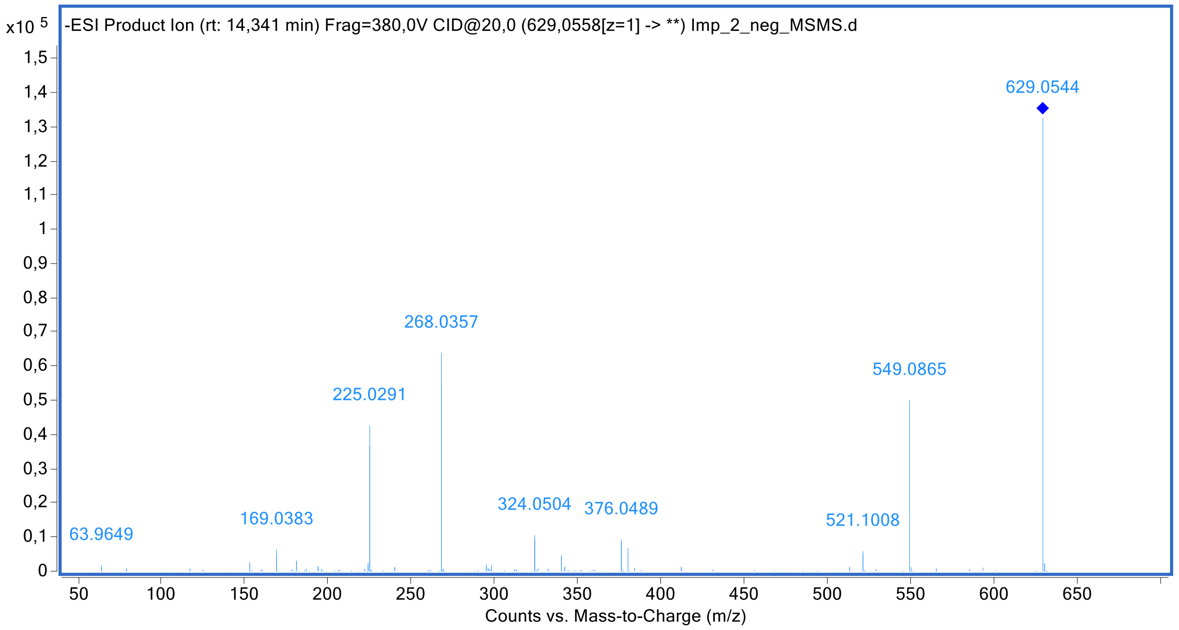

Fig. S87. ESI– MS/MS spectrum of ion at m/z 629.0558, CE 20 V

S6. Comparison of isolated DP7 to purchased analytical standard of the same structure

S6.1. HRMS and MS/MS analysis

For unambiguous confirmation of the DP7 the stock solution of purchased DP7 was prepared by weighing 1.11 mg and dissolving it in 1 ml of acetonitrile, giving the final concentration of 1.11 µg/ml. The stock solution was then diluted to achieve final concentration of 50 µg/ml.

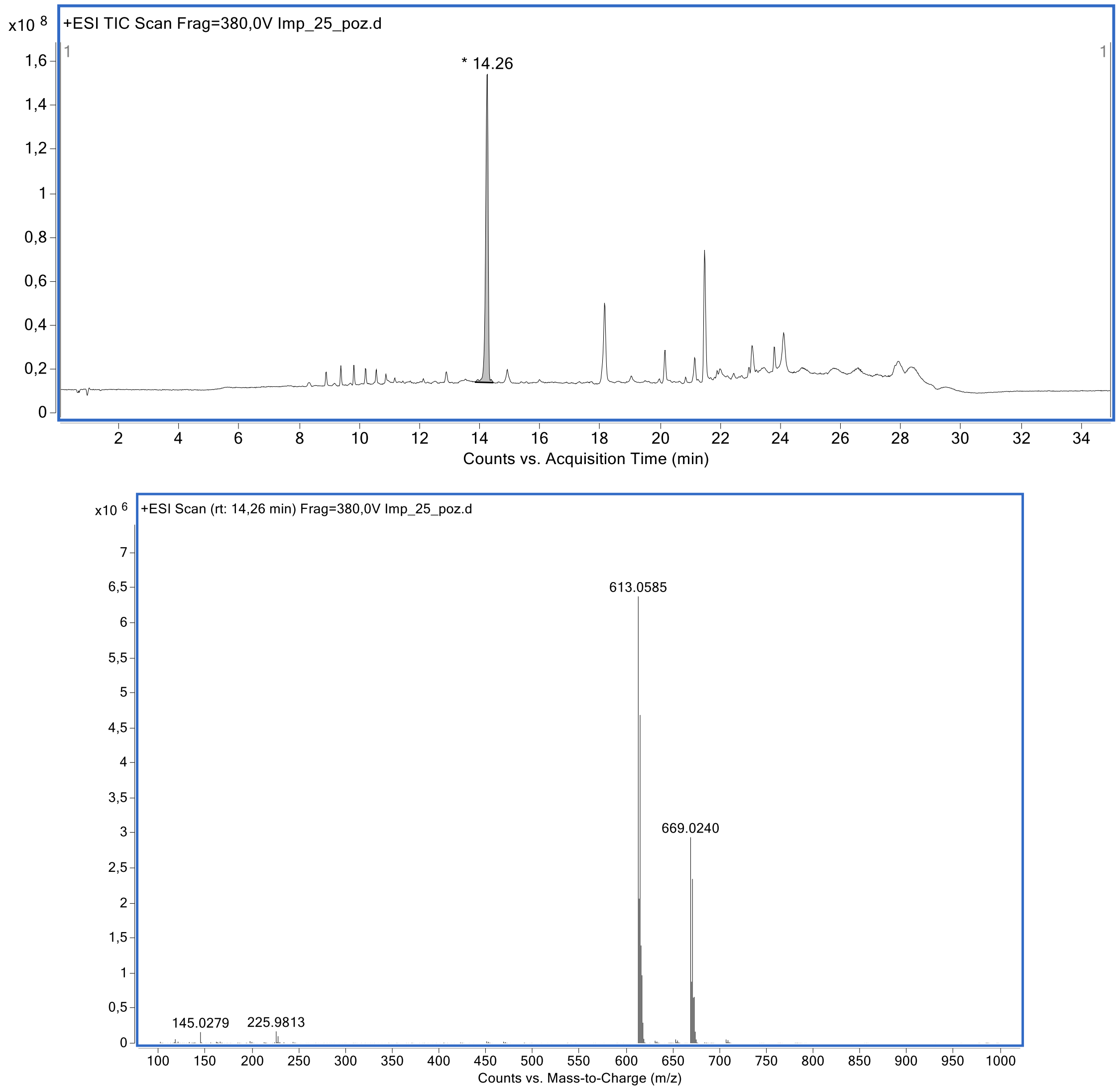

Fig. S88. ESI+ total ion chromatogram of purchased DP7 and MS spectra of chromatographic peak at 14.26 min

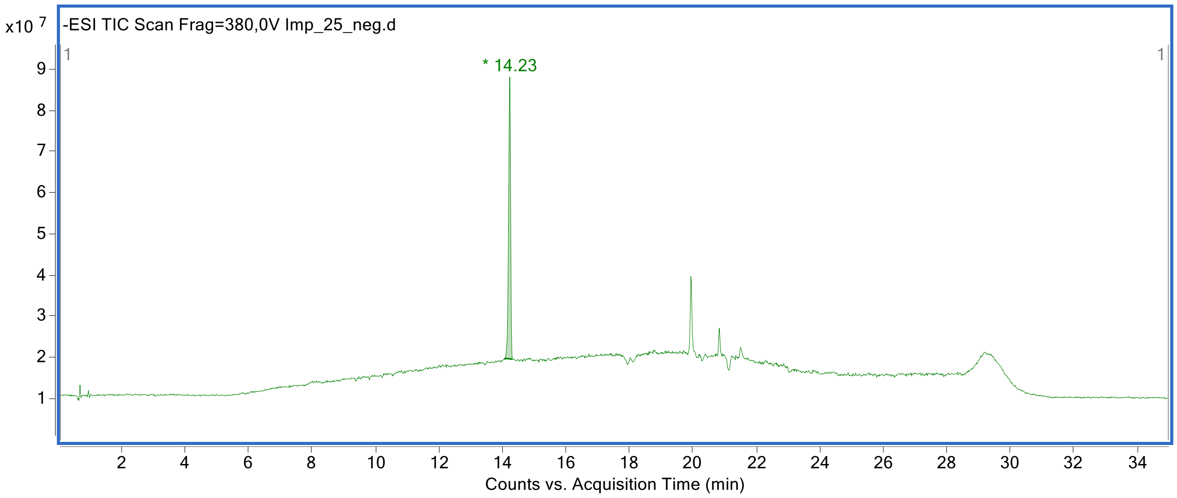

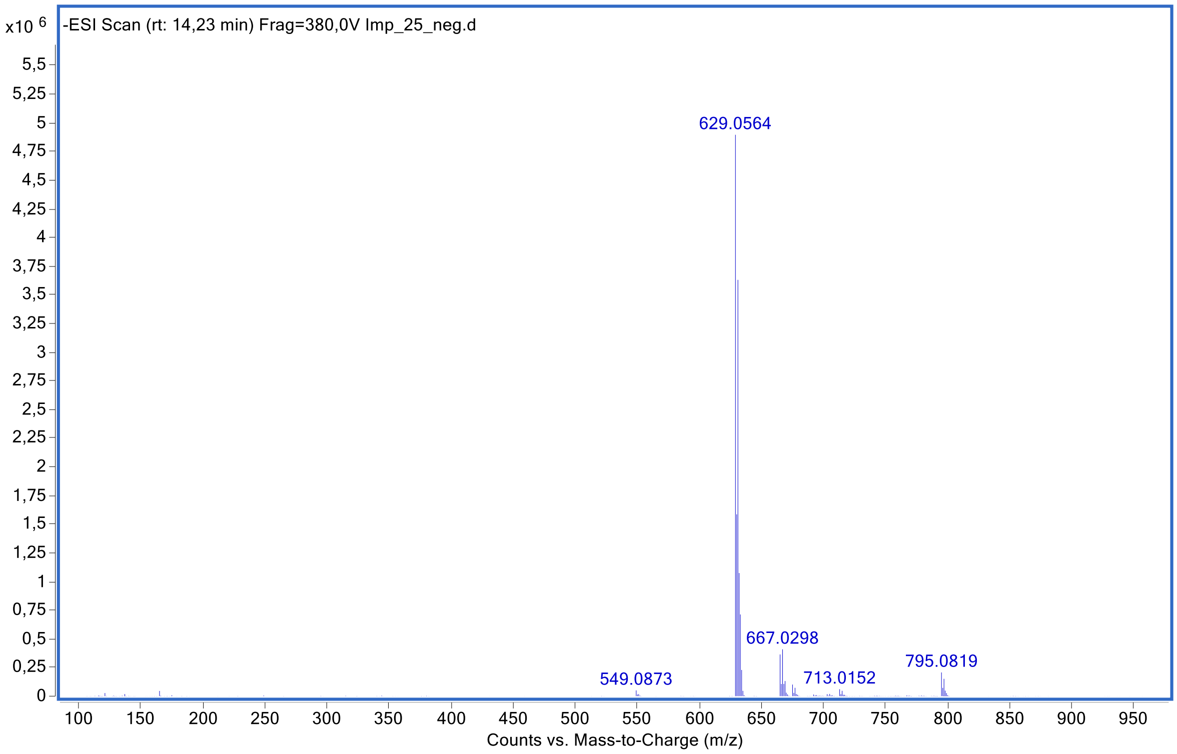

Fig. S89. ESI- total ion chromatogram of purchased DP7 and MS spectra of chromatographic peak at 14.23 min

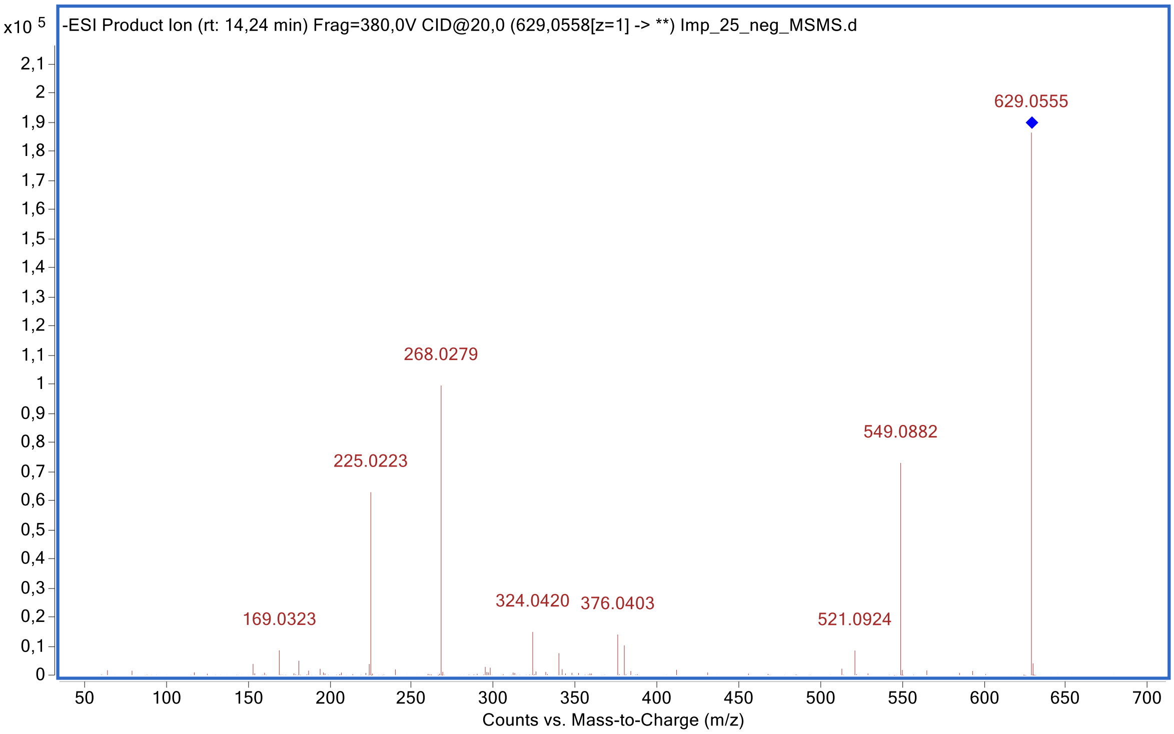

Fig. S90. ESI- MS/MS spectrum of purchased DP7, CE 20 V

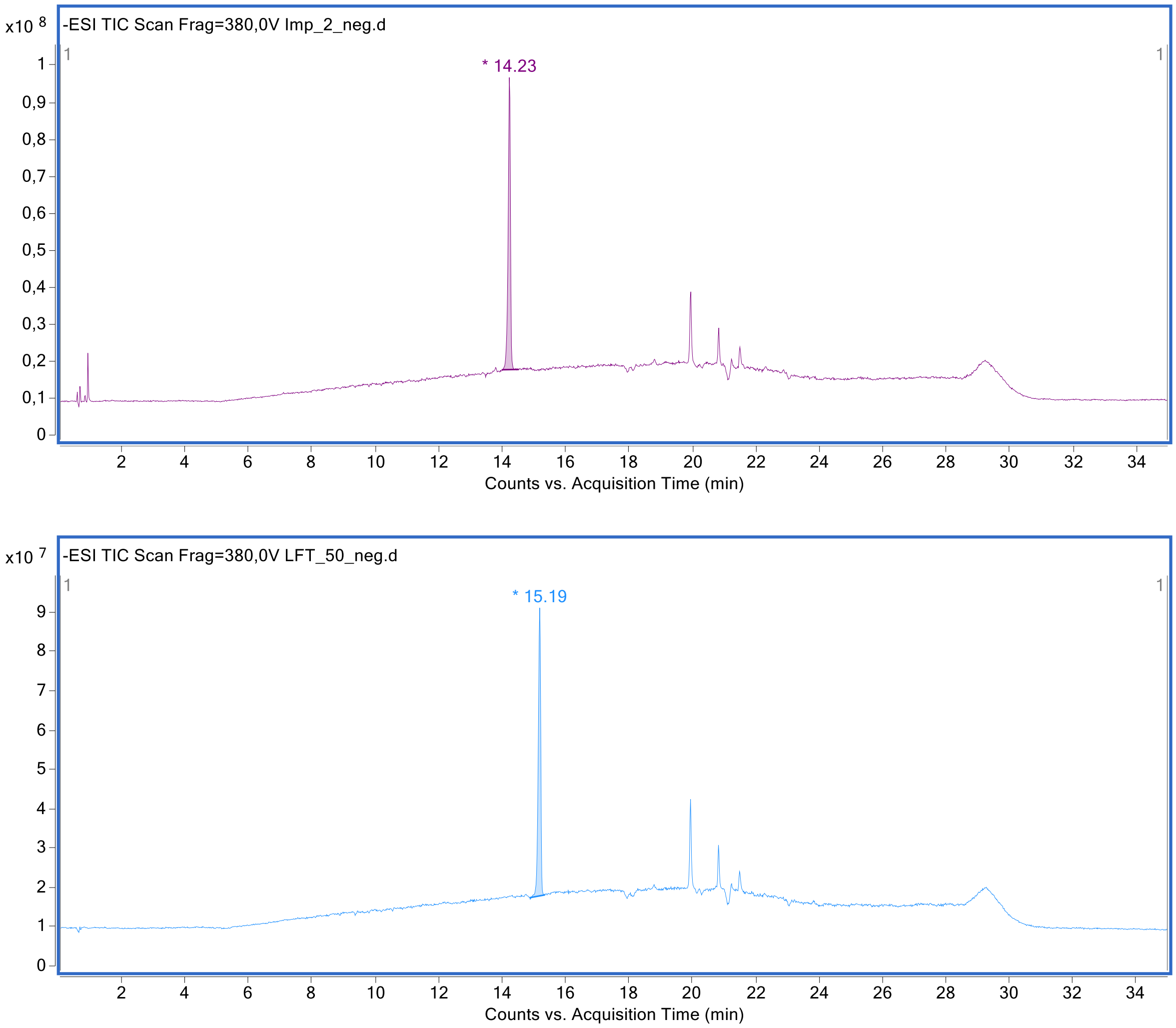

Fig. S91. ESI- total ion chromatograms of the sample of isolated DP7 (upper) and standard solution of Lifitegrast (lower).

S6.2. NMR analysis

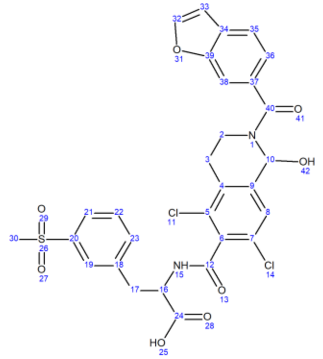

Shift1 (ppm)	H's	Type	J (Hz)	Atom1	Multiplet1
2.77	1.000	m	-	3<">	M16
2.87	1.000	dt	17.3, 2.1	3<'>	M14
3.10	3.000	br s	-	30	M17
3.18	1.000	br dd	14.0, 7.5	17<">	M15
3.31	1.000	br dd	14.0, 5.3	17<'>	M13
3.49	1.000	m	-	2<">	M12
4.23	1.000	m	-	2<'>	M11
4.51	1.000	br s	-	16	M10
6.16	1.000	br s	-	10	M09
7.01	1.000	d	1.8	33	M08
7.34	1.000	s	-	8	M07
7.37	1.000	dd	8.1, 1.1	36	M06
7.50	1.000	td	7.7, 1.8	22	M05
7.66	1.000	br d	7.9	23	M04
7.72	3.000	m	-	21, 38, 35	M03
7.86	1.000	s	-	19	M02
8.04	1.000	d	2.2	32	M01

^{1}H NMR (600 MHz, DMSO-d_6): δ = 2.72 - 2.81 (m, 1 H, 3<">), 2.87 (dt, J = 17.3, 2.1 Hz, 1 H, 3<'>), 3.10 (br s, 3 H, 30), 3.18 (br dd, J = 14.0, 7.5 Hz, 1 H, 17<">), 3.31 (br dd, J = 14.0, 5.3 Hz, 1 H, 17<'>), 3.44 - 3.54 (m, 1 H, 2<">), 4.17 - 4.29 (m, 1 H, 2<'>), 4.51 (br s, 1 H, 16), 6.16 (br s, 1 H, 10), 7.01 (d, J = 1.8 Hz, 1 H, 33), 7.34 (s, 1 H, 8), 7.37 (dd, J = 8.1, 1.1 Hz, 1 H, 36), 7.50 (td, J = 7.7, 1.8 Hz, 1 H, 22), 7.66 (br d, J = 7.9 Hz, 1 H, 23), 7.69 - 7.76 (m, 3 H, 21, 38, 35), 7.86 (s, 1 H, 19), 8.04 (d, J = 2.2 Hz, 1 H, 32) ppm

Solvent	DMSO-d6
Pulse Sequence	zg30
Temperature (degree C)	80.600
Acquisition Time (sec)	1.1403
Number of Transients	128
Points Count	32768
Sweep Width (Hz)	14367.38
Spectrum Offset (Hz)	5401.1719
Original Points Count	16384
Nucleus	1H
Frequency (MHz)	600.1300

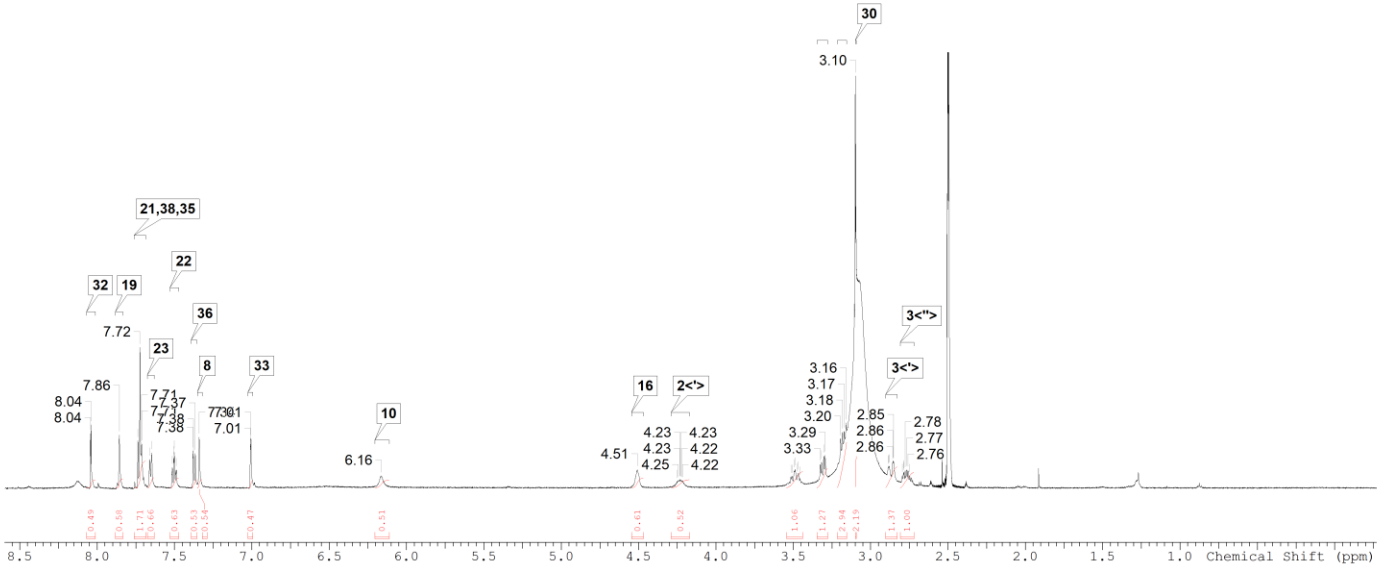

Fig. S92. Fully assigned ^{1}H spectrum of purchased DP7 in DMSO-d_6 at 80 °C

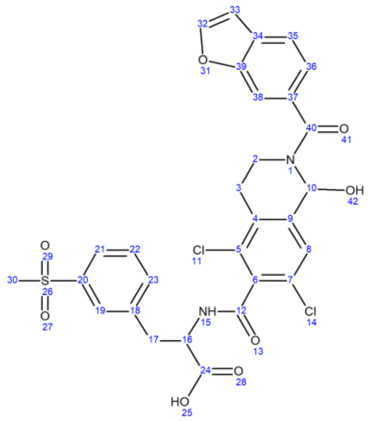

Atom	Exp. Shift (ppm)	Atom	Exp. Shift (ppm)
3	26.2	23	134.6
17	37.2	6	136.2
2	40.4	9	139.3
30	43.8	18	140.2
16	54.5	20	140.7
10	75.0	32	147.5
33	106.7	39	153.7
38	110.3	12	162.9
35	121.2	40	169.7
36	122.1	24	171.1
21	124.4		
8	127.6		
19	127.7		
22	128.5		
7	128.7		
34	128.8		
5	130.9		
4	131.7		
37	132.1		

^{13}C NMR (151 MHz, DMSO-d_6): δ = 26.2 (3), 37.2 (17), 40.4 (2), 43.8 (30), 54.5 (16), 75.0 (10), 106.7 (33), 110.3 (38), 121.2 (35), 122.1 (36), 124.4 (21), 127.6 (8), 127.7 (19), 128.5 (22), 128.7 (7), 128.8 (34), 130.9 (5), 131.7 (4), 132.1 (37), 134.6 (23), 136.2 (6), 139.3 (9), 140.2 (18), 140.7 (20), 147.5 (32), 153.7 (39), 162.9 (12), 169.7 (40), 171.1 (24) ppm

Solvent	DMSO-d6
Pulse Sequence	zgpg30
Temperature (degree C)	80.200
Acquisition Time (sec)	0.9110
Number of Transients	44800
Points Count	32768
Sweep Width (Hz)	35970.13
Spectrum Offset (Hz)	15014.3584
Original Points Count	32768
Nucleus	13C
Frequency (MHz)	150.9029

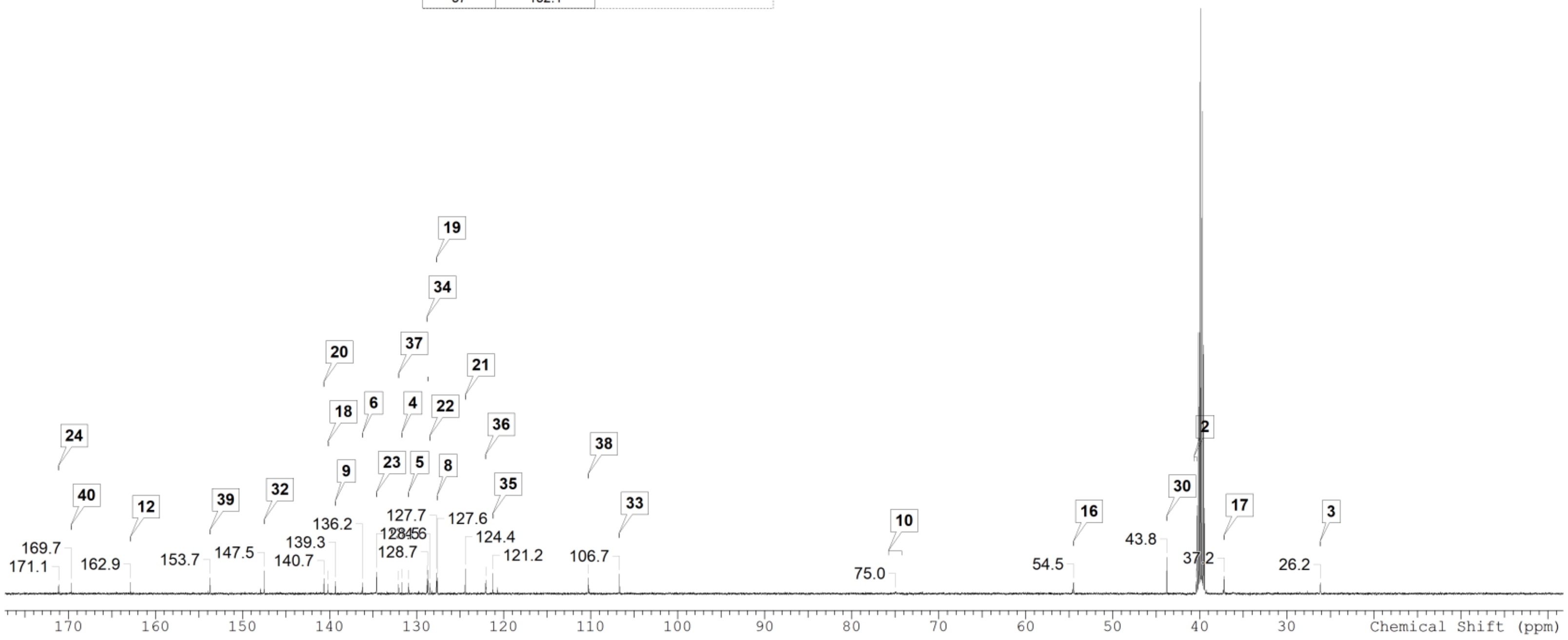

Fig. S93. Fully assigned ^{13}C spectrum of purchased DP7 in DMSO-d_6 at 80 °C

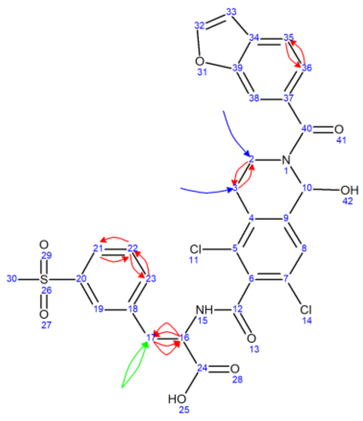

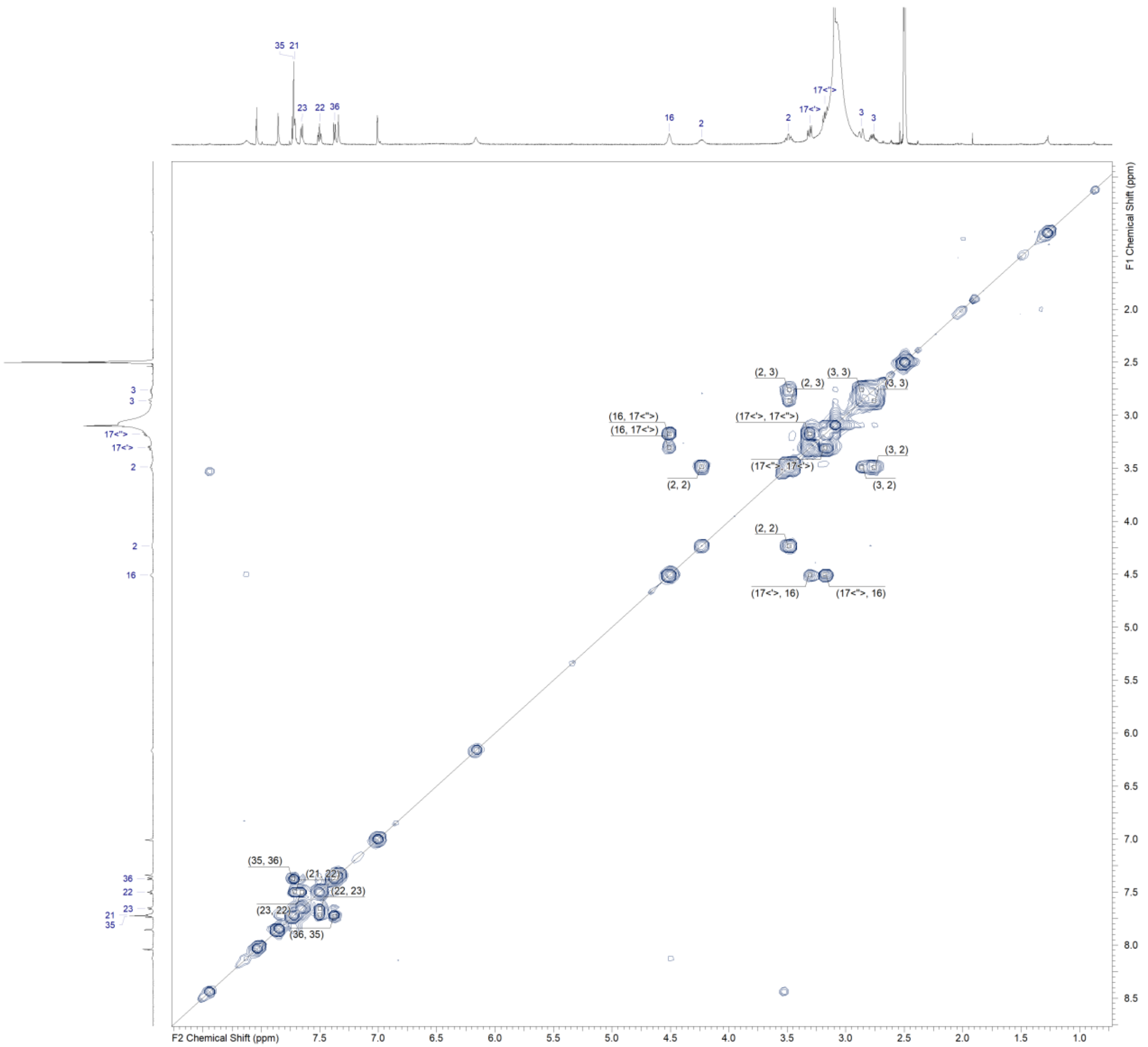

Acquisition Time (sec)	(0.1065, 0.0267)
Date	17 Jun 2024 13:22:58
Frequency (MHz)	(600.1300, 600.1300)
Nucleus	(1H, 1H)
Points Count	(2048, 2048)
Pulse Sequence	cosygpqf
Solvent	DMSO-d6
Sweep Width (Hz)	(9610.69, 9597.45)
Number of Transients	4
Spectrum Type	COSY
Temperature (degree C)	80.600

Fig. S94. Fully assigned ^{1}H-^{1}H COSY spectrum of purchased DP7 in DMSO-d_6 at 80 °C

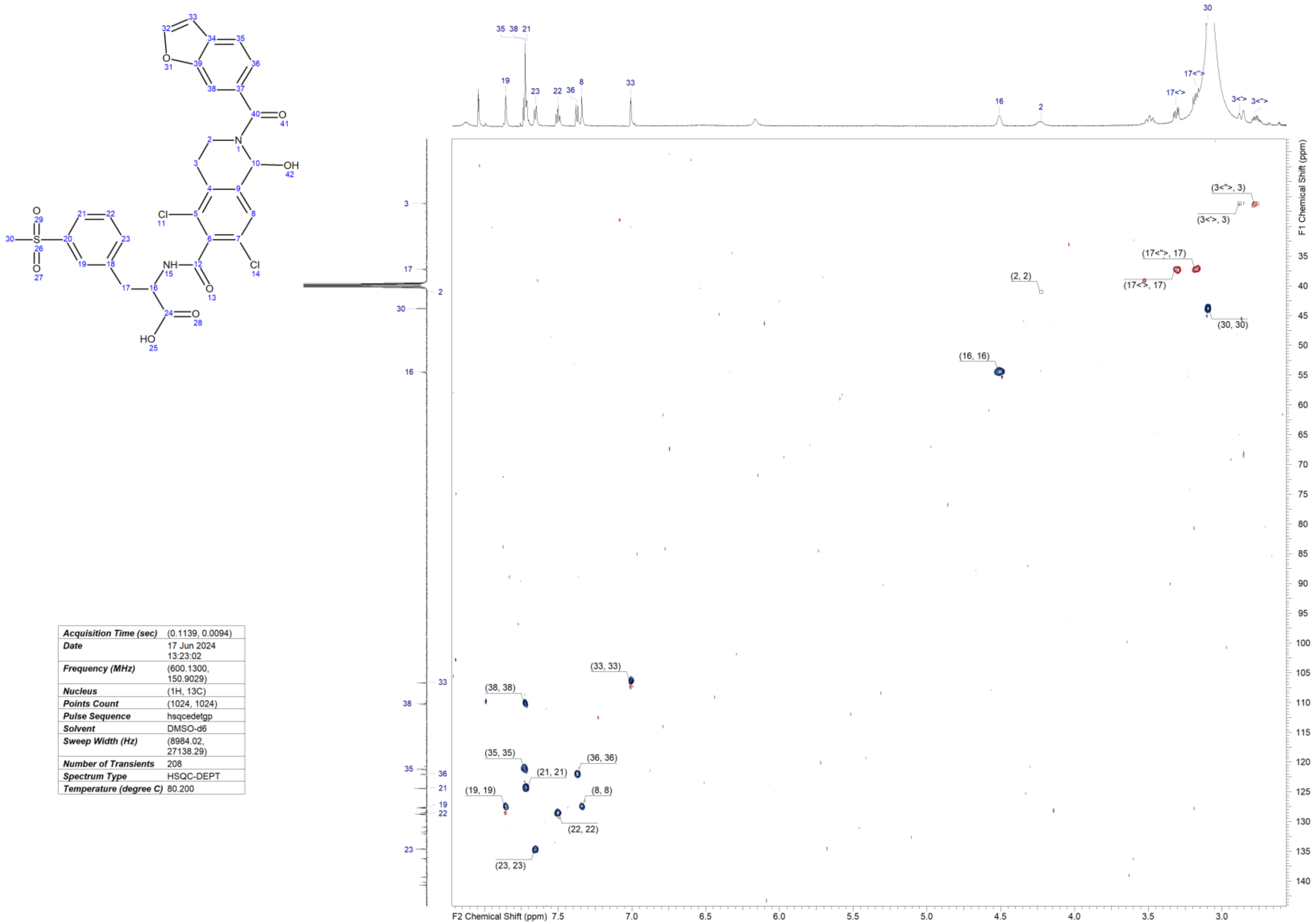

Acquisition Time (sec)	(0.1139, 0.0094)
Date	17 Jun 2024 13:23:02
Frequency (MHz)	(600.1300, 150.9029)
Nucleus	(1H, 13C)
Points Count	(1024, 1024)
Pulse Sequence	hsqcedetgp
Solvent	DMSO-d6
Sweep Width (Hz)	(8984.02, 27138.29)
Number of Transients	208
Spectrum Type	HSQC-DEPT
Temperature (degree C)	80.200

Fig. S95. Fully assigned ^1H-^{13}C HSQCe spectrum of purchased DP7 in DMSO-d_6 at 80 °C

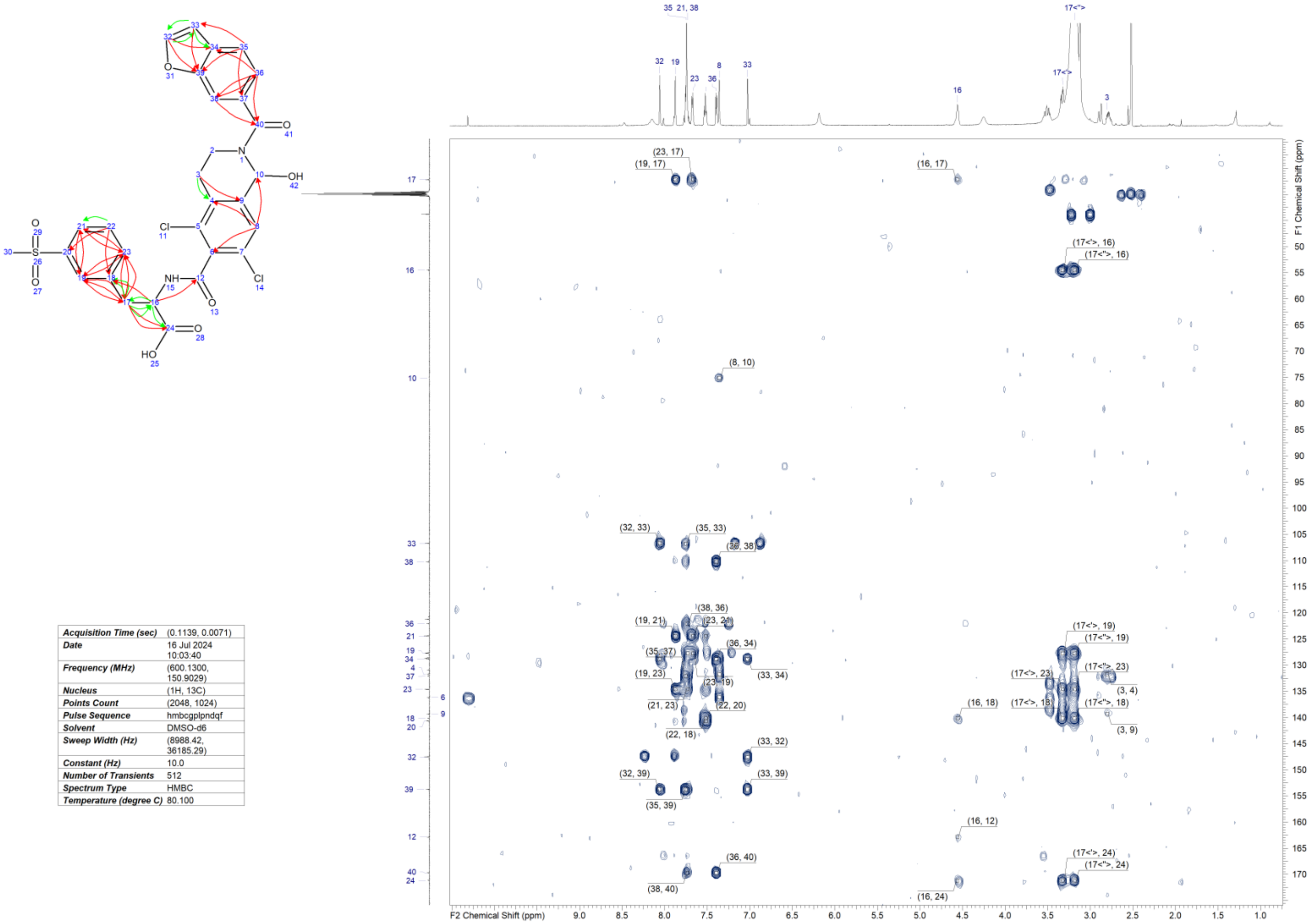

Acquisition Time (sec)	(0.1139, 0.0071)
Date	16 Jul 2024 10:03:40
Frequency (MHz)	(600.1300, 150.9029)
Nucleus	(1H, 13C)
Points Count	(2048, 1024)
Pulse Sequence	hmbcgplpndqf
Solvent	DMSO-d6
Sweep Width (Hz)	(8988.42, 36185.29)
Constant (Hz)	10.0
Number of Transients	512
Spectrum Type	HMBC
Temperature (degree C)	80.100

Fig. S96. Fully assigned ^{1}H-^{13}C HMBC spectrum of purchased DP7 in DMSO-d_6 at 80 °C

S7. Quantitative context for degradation kinetics of DP5 and DP7

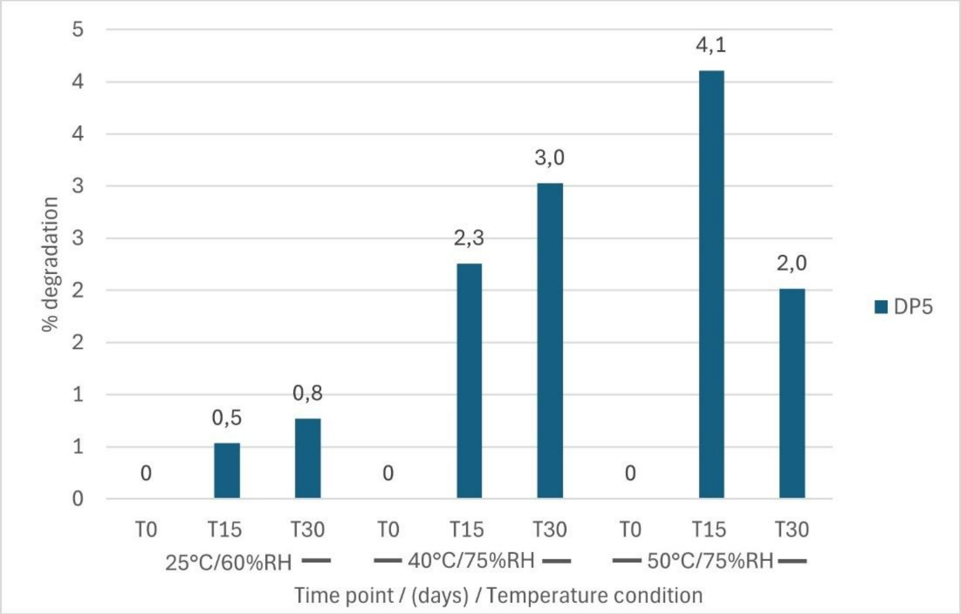

Fig. S97. DP5 content during extended degradation in 3% H2O2 at 25 °C/60%, 40 °C/75%, 50 °C/75% for 30 days (results expressed as 0 are below limit of quantification, DP7 was not detected)

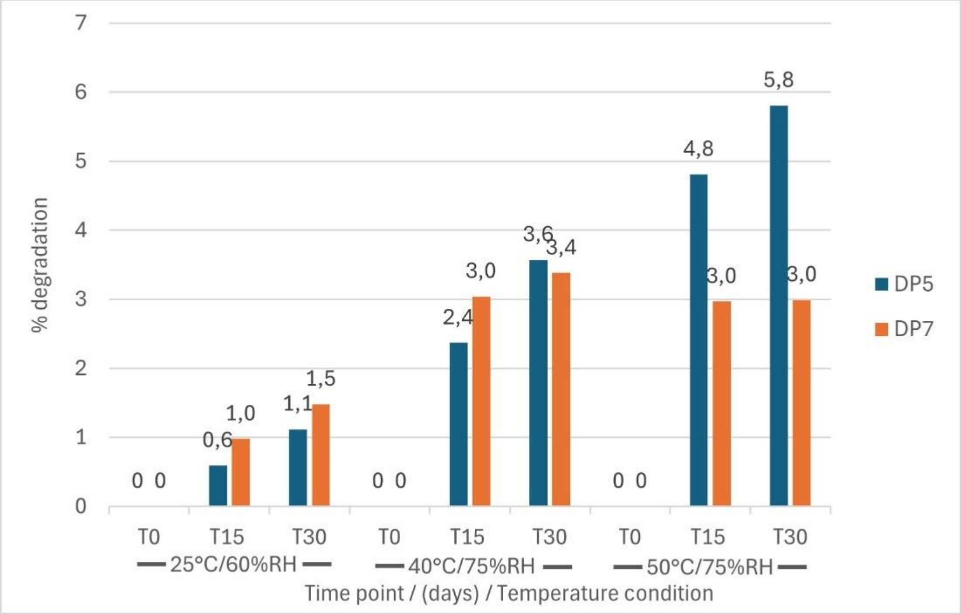

Fig. S98. DP5 (blue) and DP7 (orange) content during extended degradation with AIBN at 25 °C/60%, 40 °C/75%, 50 °C/75% for 30 days.

S8. Single-point energy calculations employing different DFT functionals

Table S12. Free energy profiles for the neutral hydrolysis of N1–C40 (blue) and N15–C12 (red) amide bonds in LIF, obtained by various DFT approaches in water compared to the (SMD)/B3LYP/Def2TZVPP model discussed in the text.

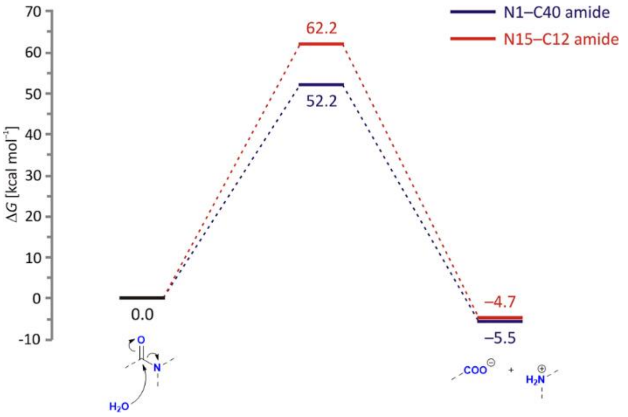

Level of theory	Neutral hydrolysis of N1–C40 (blue)	
	Activation free energy ($\Delta G^{\ddagger}$, in kcal mol^{-1})	Reaction free energy (ΔG_R, in kcal mol^{-1})
(SMD)/B3LYP/Def2TZVPP	52.2	–5.5
(SMD)/M06-2X/Def2TZVPP//(SMD)/B3LYP/Def2TZVPP	51.3	–0.5
(SMD)/ωB97XD/Def2TZVPP//(SMD)/B3LYP/Def2TZVPP	51.5	–1.0
(SMD)/PBE/Def2TZVPP//(SMD)/B3LYP/Def2TZVPP	52.6	–3.0
(SMD)/BMK/Def2TZVPP//(SMD)/B3LYP/Def2TZVPP	52.5	–6.0

Level of theory	Neutral hydrolysis of N15–C12 (red)	
	Activation free energy ($\Delta G^{\ddagger}$, in kcal mol^{-1})	Reaction free energy (ΔG_R, in kcal mol^{-1})
(SMD)/B3LYP/Def2TZVPP	62.2	–4.7
(SMD)/M06-2X/Def2TZVPP//(SMD)/B3LYP/Def2TZVPP	60.9	0.0
(SMD)/ωB97XD/Def2TZVPP//(SMD)/B3LYP/Def2TZVPP	61.8	–0.4
(SMD)/PBE/Def2TZVPP//(SMD)/B3LYP/Def2TZVPP	62.1	–2.8
(SMD)/BMK/Def2TZVPP//(SMD)/B3LYP/Def2TZVPP	62.2	–5.8

Table S13. Free energy profiles for the base-catalyzed hydrolysis of N1–C40 (blue) and N15–C12 (red) amide bonds in LIF, obtained by various DFT approaches in water compared to the (SMD)/B3LYP/Def2TZVPP model discussed in the text.

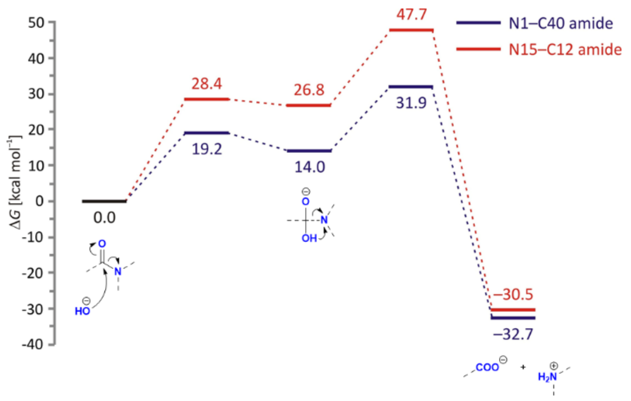

Level of theory	Base-catalyzed hydrolysis of N1–C40 (blue)	
	Activation free energy ($\Delta G^{\ddagger}$, in kcal mol^{-1})	Reaction free energy (ΔG_R, in kcal mol^{-1})
(SMD)/B3LYP/Def2TZVPP	31.9	−32.7
(SMD)/M06-2X/Def2TZVPP//(SMD)/B3LYP/Def2TZVPP	28.2	−29.3
(SMD)/ωB97XD/Def2TZVPP//(SMD)/B3LYP/Def2TZVPP	30.3	−27.9
(SMD)/PBE/Def2TZVPP//(SMD)/B3LYP/Def2TZVPP	28.2	−32.1
(SMD)/BMK/Def2TZVPP//(SMD)/B3LYP/Def2TZVPP	31.8	−33.3

Level of theory	Base-catalyzed hydrolysis of N15–C12 (red)	
	Activation free energy ($\Delta G^{\ddagger}$, in kcal mol^{-1})	Reaction free energy (ΔG_R, in kcal mol^{-1})
(SMD)/B3LYP/Def2TZVPP	47.7	−30.5
(SMD)/M06-2X/Def2TZVPP//(SMD)/B3LYP/Def2TZVPP	45.3	−26.9
(SMD)/ωB97XD/Def2TZVPP//(SMD)/B3LYP/Def2TZVPP	50.5	−25.7
(SMD)/PBE/Def2TZVPP//(SMD)/B3LYP/Def2TZVPP	44.8	−29.7
(SMD)/BMK/Def2TZVPP//(SMD)/B3LYP/Def2TZVPP	47.9	−31.3

Table S14. Free energy profiles for the acid-catalyzed hydrolysis of N1–C40 (blue) and N15–C12 (red) amide bonds in LIF, obtained by various DFT approaches in water compared to the (SMD)/B3LYP/Def2TZVPP model discussed in the text.

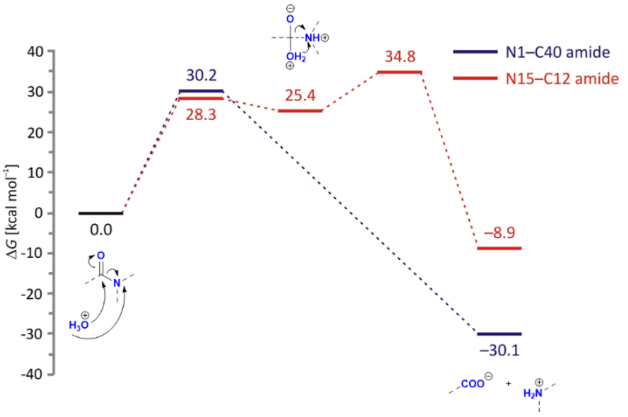

Level of theory	Acid-catalyzed hydrolysis of N1–C40 (blue)	
	Activation free energy ($\Delta G^{\ddagger}$, in kcal mol^{-1})	Reaction free energy (ΔG_R, in kcal mol^{-1})
(SMD)/B3LYP/Def2TZVPP	30.2	–30.1
(SMD)/M06-2X/Def2TZVPP//(SMD)/B3LYP/Def2TZVPP	31.6	–25.1
(SMD)/ωB97XD/Def2TZVPP//(SMD)/B3LYP/Def2TZVPP	32.0	–24.8
(SMD)/PBE/Def2TZVPP//(SMD)/B3LYP/Def2TZVPP	31.1	–28.9
(SMD)/BMK/Def2TZVPP//(SMD)/B3LYP/Def2TZVPP	32.8	–30.2

Level of theory	Acid-catalyzed hydrolysis of N15–C12 (red)	
	Activation free energy ($\Delta G^{\ddagger}$, in kcal mol^{-1})	Reaction free energy (ΔG_R, in kcal mol^{-1})
(SMD)/B3LYP/Def2TZVPP	34.8	–8.9
(SMD)/M06-2X/Def2TZVPP//(SMD)/B3LYP/Def2TZVPP	28.6	–2.2
(SMD)/ωB97XD/Def2TZVPP//(SMD)/B3LYP/Def2TZVPP	33.2	–2.1
(SMD)/PBE/Def2TZVPP//(SMD)/B3LYP/Def2TZVPP	30.0	–6.8
(SMD)/BMK/Def2TZVPP//(SMD)/B3LYP/Def2TZVPP	31.0	–9.1

S9. *In silico* toxicity scope

The applicability of the Derek Nexus, Leadscope, Toxtree, and IRFMN models primarily relates to small- and medium-sized chemicals evaluated in standard genotoxicity assays (*in vitro* and *in vivo*). While their predictions provide valuable guidance for assessing genotoxic potential, each model has specific limitations in terms of endpoint coverage, performance, and applicability domain, and the predictions may be less informative for certain exposure scenarios (*e.g.,* local or ocular administration). Therefore, these computational outcomes should be interpreted in combination with expert judgment and, where possible, complementary experimental or literature data to avoid over-interpretation.

Table S15. In silico prediction of mutagenicity, clastogenicity and aneugenicity

	**Derek *Salmonella/E. coli* mutagenicity**	**Leadscope bacterial mutagenicity**	**Derek *in vitro/in vivo* chromosome damage**	**Leadscope *in vivo* chromosomal aberration/ micronucleus**	**ToxTree *in vivo* micro-nucleus**	**IRFMN *in vivo* micro-nucleus**
**LIF**	Negative[1]	Negative[1,2]	No matching alert	Negative[5]	H-acc-path3-H-acc	Negative[3]
**DP5**	Negative[1]	Negative[1]	No matching alert	Negative[4]	H-acc-path3-H-acc	Negative[4]
**DP7**	Negative[1]	Negative[1]	No matching alert	Negative[4]	H-acc-path3-H-acc	Negative[3]

[1]The structure is within the applicability domain of the model. [2]Structure present in training/reference set. [3]Could be out of applicability domain. [4]Out of applicability domain for *in vivo* micronucleus and three models on *in vivo* chromosomal aberration. [5]Negative for *in vivo* micronucleus; out of applicability domain for all three models on *in vivo* chromosomal aberrations.

S10. List of Tables and Figures

Table S1. Sample preparation for the initial forced degradation study 4
Table S2. Experimental UPLC-UV parameters for initial forced degradation study 4
Table S3. Results of initial forced degradation study 5
Table S4. Sample preparation for extended forced degradation... 6
Table S5. Results of extended forced degradation (H_2O_2 and AIBN) 6
Table S6. Experimental parameters for UHPLC-HRMS method used for analysis of degradation products mixture after extended oxidative degradation with 3% H_2O_2 after 30 days at 50 °C/75% RH 27
Table S7. Experimental parameters for HPLC-MS method for extreme degradation study .. 60
Table S8. Experimental parameters for degradation monitoring - analytical HPLC method 63
Table S9. Experimental parameters for degradation products isolation - analytical HPLC method 63
Table S10. Experimental parameters for degradation products isolation - preparative HPLC method 64
Table S11. Experimental parameters for UHPLC-HRMS method used for structure elucidation of degradation products 65
Table S12. Free energy profiles for the neutral hydrolysis of N1–C40 (blue) and N15–C12 (red) amide bonds in LIF, obtained by various DFT approaches in water compared to the (SMD)/B3LYP/Def2TZVPP model discussed in the text. 86
Table S13. Free energy profiles for the base-catalyzed hydrolysis of N1–C40 (blue) and N15–C12 (red) amide bonds in LIF, obtained by various DFT approaches in water compared to the (SMD)/B3LYP/Def2TZVPP model discussed in the text. 87
Table S14. Free energy profiles for the acid-catalyzed hydrolysis of N1–C40 (blue) and N15–C12 (red) amide bonds in LIF, obtained by various DFT approaches in water compared to the (SMD)/B3LYP/Def2TZVPP model discussed in the text. 88
Table S15. In silico prediction of mutagenicity, clastogenicity and aneugenicity.................. 89

Fig. S1. UPLC-UV chromatograms after initial degradations of Lifitegrast in solution: (A) 3% H_2O_2; 50 °C/75% RH and (B) Water, pH=7, AIBN, 50 °C/75% RH after 7 days 5
Fig. S2. UPLC-UV chromatograms after extended degradations of Lifitegrast in solution: (A) 3% H_2O_2; 50 °C/75% RH and (B) Water, pH=7, AIBN, 50 °C/75% RH after 30 days 7
Fig. S3. Fully assigned ^{1}H spectrum of Lifitegrast in DMSO-d_6 at 80 °C 8
Fig. S4. Fully assigned ^{13}C spectrum of Lifitegrast in DMSO-d_6 at 80 °C 9
Fig. S5. Fully assigned ^{1}H-^{1}H COSY spectrum of Lifitegrast in DMSO-d_6 at 80 °C 10
Fig. S6. Fully assigned ^{1}H-^{13}C HSQCe spectrum of Lifitegrast in DMSO-d_6 at 80 °C 11
Fig. S7. Fully assigned ^{1}H-^{13}C HMBC spectrum of Lifitegrast in DMSO-d_6 at 80 °C 12
Fig. S8. Fully assigned ^{1}H spectrum of DP1 in DMSO-d_6 at 25 °C 13
Fig. S9. Fully assigned ^{13}C spectrum of DP1 in DMSO-d_6 at 25 °C.................................... 14
Fig. S10. Fully assigned ^{1}H-^{1}H COSY spectrum of DP1 in DMSO-d_6 at 25 °C.................... 15
Fig. S11. Fully assigned ^{1}H-^{13}C HSQCe spectrum of DP1 in DMSO-d_6 at 25 °C................. 16
Fig. S12. Fully assigned ^{1}H-^{13}C HMBC spectrum of DP1 in DMSO-d_6 at 25 °C................. 17
Fig. S13.. Fully assigned ^{1}H spectrum of DP2 in DMSO-d_6 at 25 °C................................. 18
Fig. S14. Fully assigned ^{13}C spectrum of DP2 in DMSO-d_6 at 25 °C 19
Fig. S15. Fully assigned ^{1}H-^{1}H COSY spectrum of DP2 in DMSO-d_6 at 25 °C.................... 20
Fig. S16. Fully assigned ^{1}H-^{13}C HSQCe spectrum of DP2 in DMSO-d_6 at 25 °C 21
Fig. S17. Fully assigned ^{1}H-^{13}C HMBC spectrum of DP2 in DMSO-d_6 at 25 °C 22
Fig. S18. Full ^{1}H-^{13}C HMBC spectrum of the oxidative degradation mixture in DMSO-d_6 at 80 °C... 23
Fig. S19. Aliphatic region of ^{1}H-^{13}C HMBC spectrum of the oxidative degradation mixture in DMSO-d_6 at 80 °C 24
Fig. S20. Aromatic region of ^{1}H-^{13}C HMBC spectrum of the oxidative degradation mixture in DMSO-d_6 at 80 °C 25
Fig. S21. List and depiction of all ^{1}H-^{13}C HMBC interactions found in oxidative degradation mixture in DMSO-d_6 at 80 °C 26
Fig. S22. ESI+ total ion chromatogram of standard solution of Lifitegrast, γ = 50 µg/ml (A) and blank (B) 28
Fig. S23. ESI- total ion chromatogram of standard solution of Lifitegrast, γ = 50 µg/ml (A) and blank (B) 29
Fig. S24. ESI+ MS spectrum of standard solution of Lifitegrast, γ = 50 µg/ml.................... 30
Fig. S25. ESI– MS spectrum of standard solution of Lifitegrast, γ = 50 µg/ml 30
Fig. S26. ESI+ MSMS spectrum of ion at m/z 615.0754, CE 20 V 31
Fig. S27. Possible fragmentation pathway of lifitegrast, ESI+ 31
Fig. S28. ESI- MSMS spectrum of ion at m/z 613.0619, CE 30 V... 32
Fig. S29. Possible fragmentation pathway of lifitegrast, ESI-... 32
Fig. S30. ESI+ total ion chromatogram of the Lifitegrast degradation products mixture after extended oxidative degradation with 3% H_2O_2 after 30 days at 50 °C/75% RH 33
Fig. S31. ESI+ MS spectrum of Lifitegrast.. 33
Fig. S32. ESI- MS spectrum of Lifitegrast... 34
Fig. S33. ESI+ MS spectrum of DP1.. 34
Fig. S34. ESI- MS spectrum of DP1 35
Fig. S35. ESI+ MS/MS spectrum of DP1, collision energy 30 V.. 35
Fig. S36. Possible fragmentation pathway of DP1 36
Fig. S37. ESI+ MS spectrum of DP3.. 37
Fig. S38. ESI- MS spectrum of DP3 37

Fig. S39. ESI+ MS/MS spectrum of DP3, collision energy 20 V 38
Fig. S40. Possible fragmentation pathway of DP3 38
Fig. S41. ESI+ MS spectrum of DP4 39
Fig. S42. ESI- MS spectrum of DP4 39
Fig. S43. ESI+ MS/MS spectrum of DP4, collision energy 20 V 40
Fig. S44. Possible fragmentation pathway of DP4 40
Fig. S45. ESI+ MS spectrum of DP5 41
Fig. S46. ESI- MS spectrum of DP5 41
Fig. S47. ESI+ MS/MS spectrum of DP5, collision energy 20 V 42
Fig. S48. Possible fragmentation pathway of DP5 42
Fig. S49. ESI+ MS spectrum of DP7 43
Fig. S50. ESI- MS spectrum of DP7 43
Fig. S51. ESI- MS/MS spectrum of DP7, collision energy 20 V 44
Fig. S52. Possible fragmentation pathway of DP7 44
Fig. S53. ESI+ MS spectrum of DP8 45
Fig. S54. ESI- MS spectrum of DP8 45
Fig. S55. ESI+ MS/MS spectrum of DP8, collision energy 20 V 46
Fig. S56. Possible fragmentation pathway of DP8 46
Fig. S57. Fully assigned ^{1}H and ^{13}C spectra of DP9 in DMSO-d_6 at 25 °C 48
Fig. S58. Fully assigned ^{1}H-^{1}H COSY spectrum of DP9 in DMSO-d_6 at 25 °C 49
Fig. S59. Fully assigned ^{1}H-^{13}C HSQCe spectrum of DP9 in DMSO-d_6 at 25 °C 50
Fig. S60. Fully assigned ^{1}H-^{13}C HMBC spectrum of DP9 in DMSO-d_6 at 25 °C 51
Fig. S61. Fully assigned ^{1}H and ^{13}C spectra, structure, numbering and full assignment of DP10 in DMSO-d_6 at 80 °C 52
Fig. S62. Fully assigned ^{1}H-^{1}H COSY spectrum of DP10 in DMSO-d_6 at 80 °C 53
Fig. S63. Fully assigned ^{1}H-^{13}C HSQCe spectrum of DP10 in DMSO-d_6 at 80 °C 54
Fig. S64. Fully assigned ^{1}H-^{13}C HMBC spectrum of DP10 in DMSO-d_6 at 80 °C 55
Fig. S65. Fully assigned ^{1}H spectrum and ^{13}C chemical shifts (extracted from ^{1}H-^{13}C HMBC) of DP11 in DMSO-d_6 at 80 °C 56
Fig. S66. Fully assigned ^{1}H-^{1}H COSY spectrum of DP11 in DMSO-d_6 at 80 °C 57
Fig. S67. Fully assigned ^{1}H-^{13}C HSQCe spectrum of DP11 in DMSO-d_6 at 80 °C 58
Fig. S68. Fully assigned ^{1}H-^{13}C HMBC spectrum of DP11 in DMSO-d_6 at 80 °C 59
Fig. S69. (A) LC-UV, (B) LC-MS chromatograms and (C) MS spectrum of fraction 1 61
Fig. S70. LC-UV chromatograms of fraction 2 62
Fig. S71. LC-UV chromatograms of fraction 3 62
Fig. S72. ESI+ total ion chromatogram of DP5 and MS spectrum of chromatographic peak at retention time 11.338 min 66
Fig. S73. ESI– total ion chromatogram of DP5 and MS spectrum of chromatographic peak at retention time 11.325 min 67
Fig. S74. ESI+ MS/MS spectrum of ion at m/z 635.0652, CE 20 V 68
Fig. S75. ESI– MS/MS spectrum of ion at m/z 633.0507, CE 30 V 68
Fig. S76. ESI+ total ion chromatogram of DP12 and MS spectrum of chromatographic peak at retention time 13.739 min 69
Fig. S77. ESI– total ion chromatogram of DP12 and MS spectrum of chromatographic peak at retention time 13.738 min 70
Fig. S78. ESI+ MS/MS spectrum of DP12, CE 20 V 71
Fig. S79. Possible fragmentation pathway of DP12, ESI+ 71

Fig. S80. ESI– MS/MS spectrum of DP12, CE 20 V 72
Fig. S81. Possible fragmentation pathway of DP12, ESI- 72
Fig. S82. ESI+ total ion chromatogram of DP7 73
Fig. S83. ESI– total ion chromatogram of DP7 and MS spectrum of chromatographic peak at retention time 14.365 min 73
Fig. S84. ESI+ MS/MS spectrum of ion at m/z 631.0703, CE 10 V 74
Fig. S85. ESI+ MS/MS spectrum of ion at m/z 631.0703, CE 20 V 74
Fig. S86. Possible fragmentation pathway of DP7 75
Fig. S87. ESI– MS/MS spectrum of ion at m/z 629.0558, CE 20 V 75
Fig. S88. ESI+ total ion chromatogram of purchased DP7 and MS spectra of chromatographic peak at 14.26 min 76
Fig. S89. ESI- total ion chromatogram of purchased DP7 and MS spectra of chromatographic peak at 14.23 min 77
Fig. S90. ESI- MS/MS spectrum of purchased DP7, CE 20 V 78
Fig. S91. ESI- total ion chromatograms of the sample of isolated DP7 (upper) and standard solution of Lifitegrast (lower). 79
Fig. S92. Fully assigned ^{1}H spectrum of purchased DP7 in DMSO-d_6 at 80 °C 80
Fig. S93. Fully assigned ^{13}C spectrum of purchased DP7 in DMSO-d_6 at 80 °C 81
Fig. S94. Fully assigned ^{1}H-^{1}H COSY spectrum of purchased DP7 in DMSO-d_6 at 80 °C ... 82
Fig. S95. Fully assigned ^{1}H-^{13}C HSQCe spectrum of purchased DP7 in DMSO-d_6 at 80 °C 83
Fig. S96. Fully assigned ^{1}H-^{13}C HMBC spectrum of purchased DP7 in DMSO-d_6 at 80 °C. 84
Fig. S97. DP5 content during extended degradation in 3% H2O2 at 25 °C/60%, 40 °C/75%, 50 °C/75% for 30 days (results expressed as 0 are below limit of quantification, DP7 was not detected) 85
Fig. S98. DP5 (blue) and DP7 (orange) content during extended degradation with AIBN at 25 °C/60%, 40 °C/75%, 50 °C/75% for 30 days. 85
